# African swine fever virus I177L induces host inflammatory responses by facilitating the TRAF6-TAK1 axis and NLRP3 inflammasome assembly

**DOI:** 10.1128/jvi.02080-24

**Published:** 2025-03-26

**Authors:** Pan-Xue Wu, Wen-Ping Yang, Tao Feng, Jing Zhang, Guo-Qiang Zhu, Xu-Guang Du, Yi Ru, Yao-Feng Zhao, Sen Wu, Dan Li, Hai-Xue Zheng

**Affiliations:** 1 State Key Laboratory of Animal Biotech Breeding College of Biological Sciences, National Engineering Laboratory for Animal Breeding, Frontiers Science Center for Molecular Design Breeding, China Agricultural University 34752 , Beijing, China; 2 State Key Laboratory for Animal Disease Control and Prevention, College of Veterinary Medicine, Lanzhou University, Lanzhou Veterinary Research Institute, Chinese Academy of Agricultural Sciences, Lanzhou, China; 3 Gansu Province Research Center for Basic Disciplines of Pathogen Biology, Lanzhou, China; Northwestern University Feinberg School of Medicine, Chicago, Illinois, USA

**Keywords:** African swine fever virus, I177L, inflammatory response, TRAF6, TAK1, NLRP3 inflammasome

## Abstract

African swine fever virus (ASFV) is the pathogen of African swine fever (ASF), and its infection causes a lethal disease in pigs, with severe pathological lesions. These changes indicate excessive inflammatory responses in infected pigs, which is the main cause of death, but the ASFV proteins worked in this physiological process and the mechanisms underlying ASFV-induced inflammation remain unclear. Here, we identify that viral I177L works in these inflammatory responses. Mechanistically, I177L facilitates TRAF6 ubiquitination that enhances its binding to TAK1, which promotes TAK1 ubiquitination and phosphorylation. These processes depend on the E3 ubiquitin ligase activity of TRAF6. The upregulation of I177L to TRAF6-TAK1 interaction and TAK1 activation is responsible for I177L’s activated effect on the NF-κB signaling pathway. Additionally, I177L promotes assembly of the NLRP3 inflammasome and ASC oligomerization, thus leading to the activation of the NLRP3 inflammasome and the production and secretion of mature IL-1β. TAK1 inhibition efficiently reverses ASFV-activated NF-κB signaling and inflammatory responses and suppresses ASFV replication. Furthermore, I177L-deficient ASFV induces milder inflammatory responses in pigs compared with parental ASFV, which still protects pigs against ASFV challenge. The finding confirms ASFV I177L as an important proinflammatory protein *in vitro* and *in vivo* and reveals a key mechanism underlying ASFV-mediated inflammatory responses for the first time, which enriches our knowledge of the complex ASFV, thus benefiting our understanding of the interplay between ASFV infection and the host’s inflammatory responses.

**IMPORTANCE:**

African swine fever (ASF) is a devastating viral disease in pigs, and excessive inflammatory responses induced by ASFV mainly cause death. Thus, the study of the proinflammatory virulent proteins and the detailed mechanisms are important to ASF control. Here, I177L was demonstrated to be an essential protein in ASFV-mediated inflammation, which performs by simultaneously activating the NF-κB signaling and the NLRP3 inflammasome. The finding elucidates the molecular mechanism underlying ASFV-activated inflammatory responses for the first time. It provides a theoretical foundation for reducing the high mortality caused by excessive inflammation and opens new avenues for small-molecule drug development and vaccine design targeting ASFV.

## INTRODUCTION

African swine fever (ASF) is a highly contagious viral disease in pigs, with mortality rates approaching 100%, as measured by virulent isolates (1, 2). Since there are no effective vaccines or treatments, ASF has caused great economic losses (3). Therefore, urgent investigations into the pathogenesis of ASFV are needed to understand the mechanisms of death and develop new vaccines. However, the extensive size of the ASFV genome, which ranges from 170 to 193 kb (4–6), makes the identification of the viral proteins responsible for ASFV pathogenesis a significant challenge.

The inflammatory response, a universal physiological reaction, is triggered when the host encounters harmful stimuli or conditions, including viral infection or tissue injury (7). Acute ASFV infection induces severe clinical symptoms and pathological changes in pigs, indicating activation of the inflammatory responses (1, 8–10). Similar to other highly infectious viruses, such as SARS-CoV-2 (11, 12), the severity and high mortality of ASFV are primarily associated with profound and sustained inflammation. Therefore, understanding the proinflammatory mechanisms and pathogenesis of ASFV is vital to reducing high mortality rates and identifying potential therapeutic targets for inflammation control. Several ASFV proteins have been implicated in inflammation. For example, ASFV MGF505-7R negatively regulates inflammation by interacting with IKKα and inhibiting NLRP3 inflammasome assembly (13, 14). H240R inhibits IL-1β production by promoting NEMO degradation and preventing NLRP3 oligomerization (15, 16). L83L demonstrates its potential immunomodulatory role by interacting with IL-1β (17). CD2v- and MGF360-505R-deficient ASFV inhibits the NF-κB signaling and IL-1β production without a well-defined mechanism (18). However, the precise mechanisms of ASFV-induced inflammation and the functional proteins involved in this process remain largely undetermined.

Our study illuminates the important role of viral I177L in ASFV-induced inflammation, both *in vitro* and *in vivo*. On one hand, I177L activates the NF-κB signaling pathway through the TRAF6-TAK1 axis, leading to increased expressions of NLRP3 and pro-IL-1β. Using a TAK1 inhibitor can effectively mitigate ASFV-mediated inflammation and viral replication. On the other hand, I177L facilitates assembly of the NLRP3 inflammasome and ASC oligomerization, which triggers the production and secretion of IL-1β. Additionally, I177L-deleted ASFV provides efficient protection against ASFV challenges in inoculated pigs. Our findings assist in comprehensively understanding the relationship between ASFV infection and host inflammatory responses. They also provide clues for suppressing viral replication and host inflammation and shed light on ASFV vaccine design.

## RESULTS

### ASFV I177L is the main proinflammatory protein

To determine ASFV’s impact on the inflammatory response, porcine bone marrow-derived macrophages (BMDMs) were infected with ASFV (ASFV CN/GS/2018, which was provided by the Lanzhou Veterinary Research Institute, Chinese Academy of Agricultural Sciences) at a different time or dose. The results showed that ASFV infection induced IL-1β and TNFα mRNA expression and IL-1β secretion in a time- and dose-dependent manner in BMDMs (Fig. S1A through F). Concurrently, IL-1β maturation (p17) and Caspase-1 cleavage (p20) in cell supernatants, and pro-IL-1β production in cell lysates were activated by ASFV (Fig. S1G and H). Taken together, we demonstrate that ASFV activates the inflammatory response in cultured cells. Conversely, inhibiting ASFV replication via ultraviolet (UV) or heat treatment or suppressing ASFV protein expression with cycloheximide (CHX) (19) mitigated these processes (Fig. S1I through K). These findings underscore the role of active ASFV infection, replication, and protein expression in inciting ASFV-induced inflammation.

**Fig 1:**
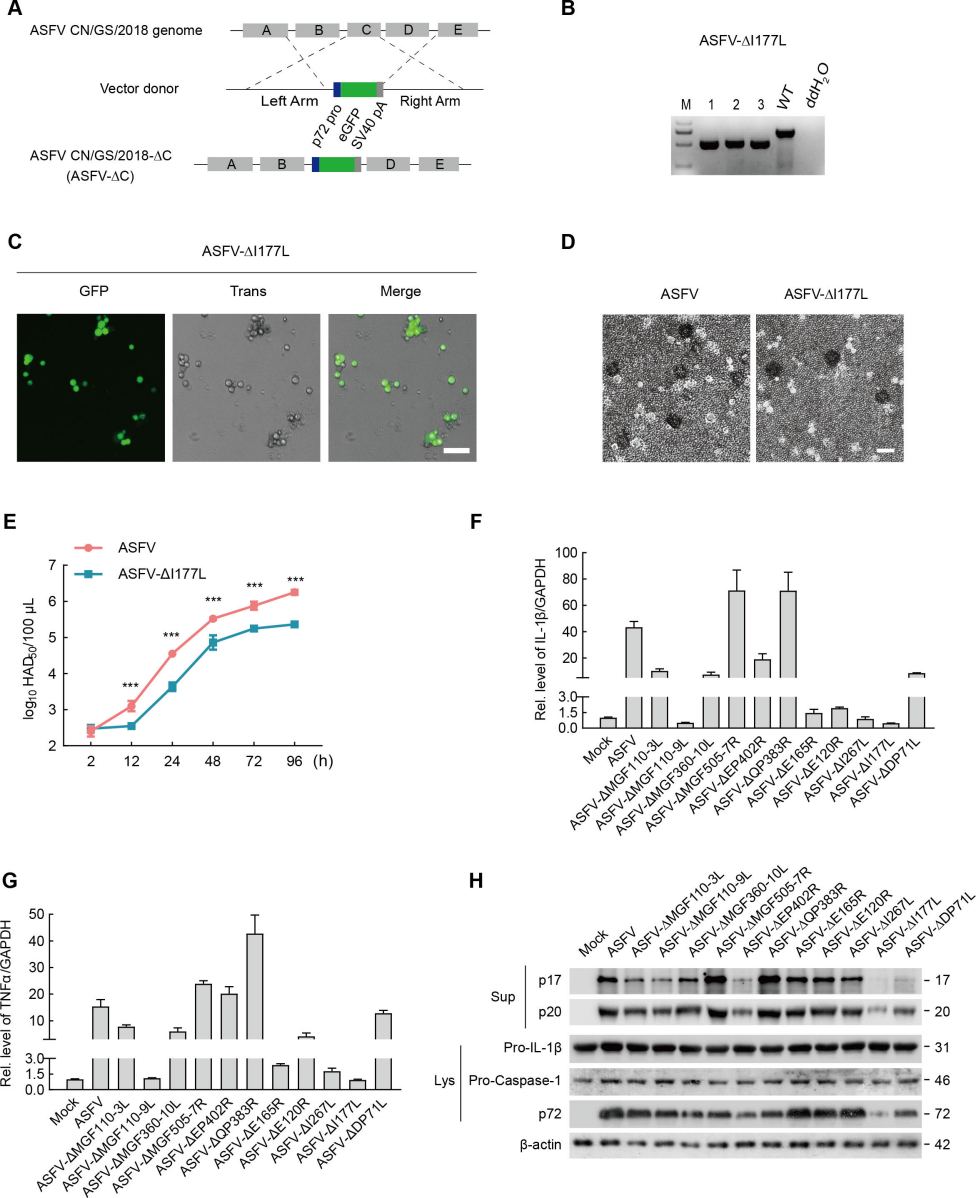
Screening assay of various single-gene-deleted ASFVs on inflammation. (A) The construction strategy of ASFV single-gene-deleted recombinant virus. (B-E) The construction of ASFV-ΔI177L recombinant virus. (B) Identification of the successful construction of ASFV-ΔI177L using Genotyping PCR. (C-E) The infection, hemadsorption, and replication of ASFV-ΔI177L compared with those of ASFV. (F-H) BMDMs were infected with ASFV or a series of ASFV single-gene-deleted recombinant viruses at 0.1 MOI for 24 h. (F, G) The expression of IL-1β and TNF-α mRNA was determined by qPCR. (H) Mature IL-1β (p17) and cleaved Caspase-1 (p20) in the supernatant of BMDMs were analyzed by Western blot. Scale bar, 50 µm. Data are mean (*n* = 3) ± s.e.m. ns, *P* > 0.05, ***P < 0.001.

As a large DNA virus, ASFV encodes 150–200 viral proteins (2). To pinpoint ASFV proteins that contribute to ASFV-activated inflammation, we implemented a screening assay by comparing the impact of parental ASFV and a series of single gene-deleted recombinant ASFVs on inflammation. Recombinant viruses were generated by replacing the target gene with the p72 promoter-GFP-polyA cassette (Fig. 1A). Successful deletion of ASFV gene was determined using Genotyping PCR (Fig. 1B; Fig. S2A). In the ASFV-ΔI177L genome obtained by next-generation sequencing (the genome sequence and read depth are shown in Supplementary materials; I177L, red font; eGFP, green font), five single-nucleotide variations (SNVs), one deletion, and one insertion in the non-coding region were detected, except for the indicated changes (Fig. S2B). These additional mutations most likely emerged during passage and exerted a minimal impact on I177L function. In ASFV-ΔI177L, its capacity for infection of porcine alveolar macrophages (PAMs) and hemadsorption remained, similar to that of parental ASFV (Fig. 1C and D). However, ASFV replication was patently attenuated when I177L was deleted (Fig. 1E). The screening assay results unveiled that the expression of IL-1β and TNF-α mRNA (Fig. 1F and G), the maturation of IL-1β (p17), and the cleavage of Caspase-1 (p20) (Fig. 1H) in ASFV-ΔI177L-infected cells were lowest compared with those in the parental ASFV-infection group, demonstrating the impact of ASFV-ΔI177L on modulating inflammatory responses and the crucial role of ASFV I177L in ASFV-mediated inflammation.

### Deficiency of I177L greatly attenuates inflammation activation and IL-1β production induced by ASFV

**Fig 2:**
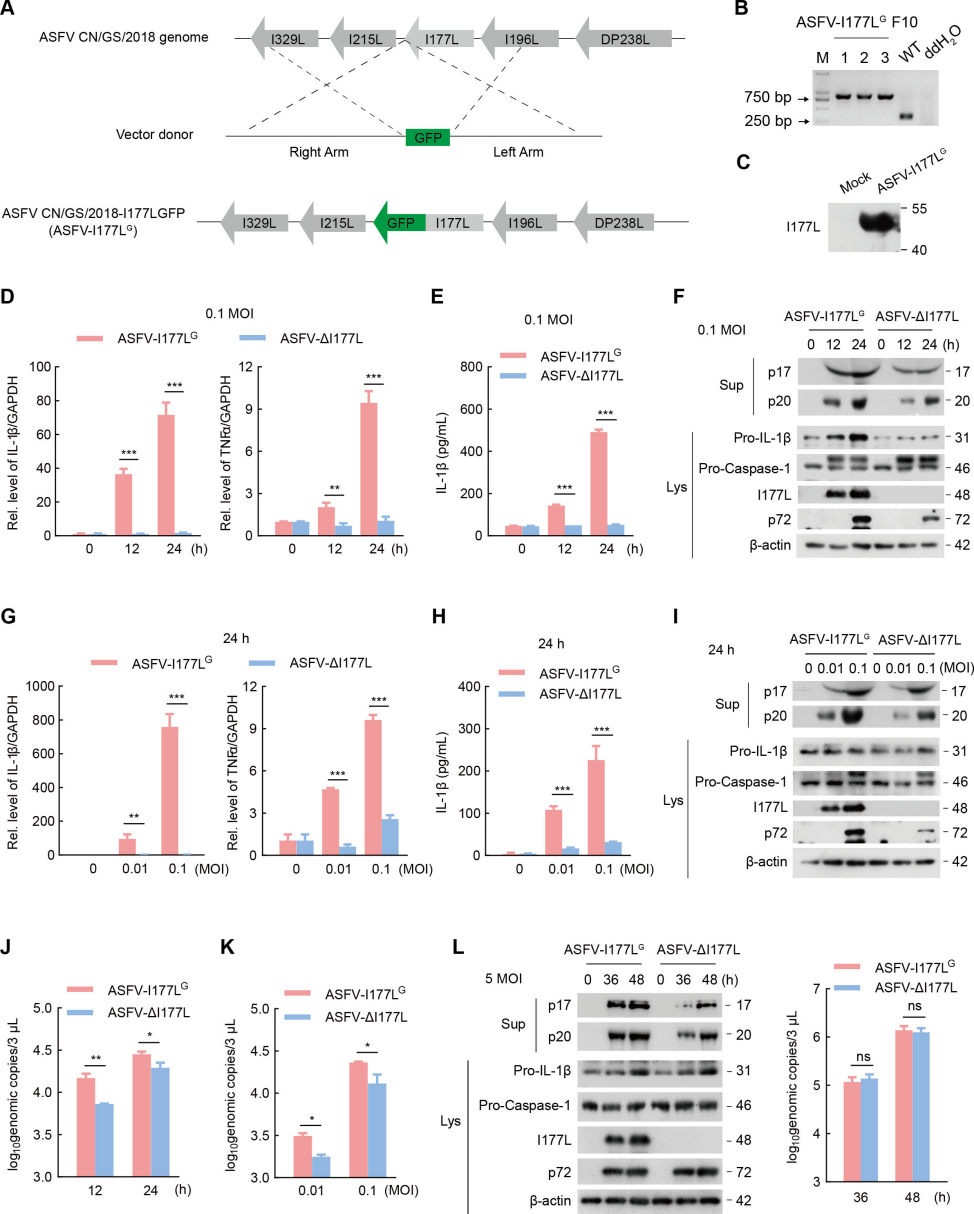
I177L promotes inflammation and IL-1β production. (A-C) The design and identification of ASFV-I177L^G^ and expression of I177L-GFP fusion protein. (D-K) BMDMs were infected with ASFV-I177L^G^ or ASFV-ΔI177L at 0.1 MOI for the indicated time or for 24 h at the indicated dose. (D, G) QPCR of IL-1β and TNF-α mRNA. (E, H) ELISA of IL-1β levels. (F, I) Western blot of Mature IL-1β (p17) and cleaved Caspase-1 (p20). (J, K) Absolute qPCR of the genomic copies of ASFV-I177L^G^ or ASFV-ΔI177L in BMDMs. (L) Western blot of Mature IL-1β (p17) and cleaved Caspase-1 (p20) in BMDMs infected with ASFV-I177L^G^ or ASFV-ΔI177L at 5 MOI for 36 or 48 h. Absolute qPCR of the genomic copies of ASFV-I177L^G^ and ASFV-ΔI177L. Data are mean (*n* = 3) ± s.e.m. *P < 0.05, **P < 0.01, ***P < 0.001.

The effect of I177L on ASFV-induced inflammation was then specifically explored by comparing the level of inflammatory responses induced by ASFV-ΔI177L and parental ASFV. To better show the expression of I177L in ASFV infection, we generated a recombinant virus, ASFV-CN/GS/2018-I177LGFP (ASFV-I177L^G^), in which the ASFV I177L gene was fused with GFP sequences to express a fusion protein (Fig. 2A and B). The successful construction of this recombinant virus not only visualized I177L expression during the infection and replication period of ASFV but also served as the antibody of I177L by targeting GFP (Fig. 2C). The capacity of ASFV-I177L^G^ for infection, hemadsorption, and replication was compared with those of the parental ASFV (Fig. S3A through C). Besides the designed changes, seven single-nucleotide variations (SNVs), one deletion, and one insertion in the non-coding region were detected in the genome sequence of ASFV-I177L^G^ using next-generation sequencing (Fig. S3D) (Genome sequence and read depth of ASFV-I177L^G^ are shown in Supplementary materials; I177L, red font; eGFP, green font). These additional mutations, produced due to passaging of the recombinant virus, had a minimal impact on I177L function. Thus, ASFV-I177L^G^ and ASFV-ΔI177L were implied in our following investigations to determine I177L function.

In BMDMs, IL-1β and TNFα mRNA were activated by ASFV-I177L^G^ but not by ASFV-ΔI177L (Fig. 2D and G), mature IL-1β secretion was largely induced by ASFV-I177L^G^ compared with ASFV-ΔI177L (Fig. 2E and H), IL-1β maturation (p17), and Caspase-1 cleavage (p20) in cell supernatants, and pro-IL-1β production in cell lysates were significantly activated by ASFV-I177L^G^ relative to ASFV-ΔI177L (Fig. 2F and I). Moreover, the genomic copies of ASFV-I177L^G^ and ASFV-ΔI177L were increased over time and dose, with the former higher than the latter (Fig. 2J and K), indicating that ASFV-I177L^G^ and ASFV-ΔI177L replicate well in BMDMs, and I177L is a virulent factor. Taken together, we demonstrate that ASFV I177L time- and dose-dependently activates the production and secretion of IL-1β in cultured cells.

Moreover, we conducted a high-dose infection experiment to ensure synchronized infection and to rule out the possibility that the higher inflammation induced by ASFV-I177L^G^ was due to its stronger replication ability. Importantly, we observed that the p17 and p20 levels in ASFV-I177L^G^-infected BMDMs remained higher than those in ASFV-ΔI177L-infected BMDMs, with the genomic copies of the two recombinant viruses equivalent (Fig. 2L). In conclusion, our findings underscore the significant role of I177L in ASFV-induced inflammation and IL-1β production.

### I177L facilitates the NF-κB signaling by enhancing the formation of the TRAF6-TAK1 complex

**Fig 3:**
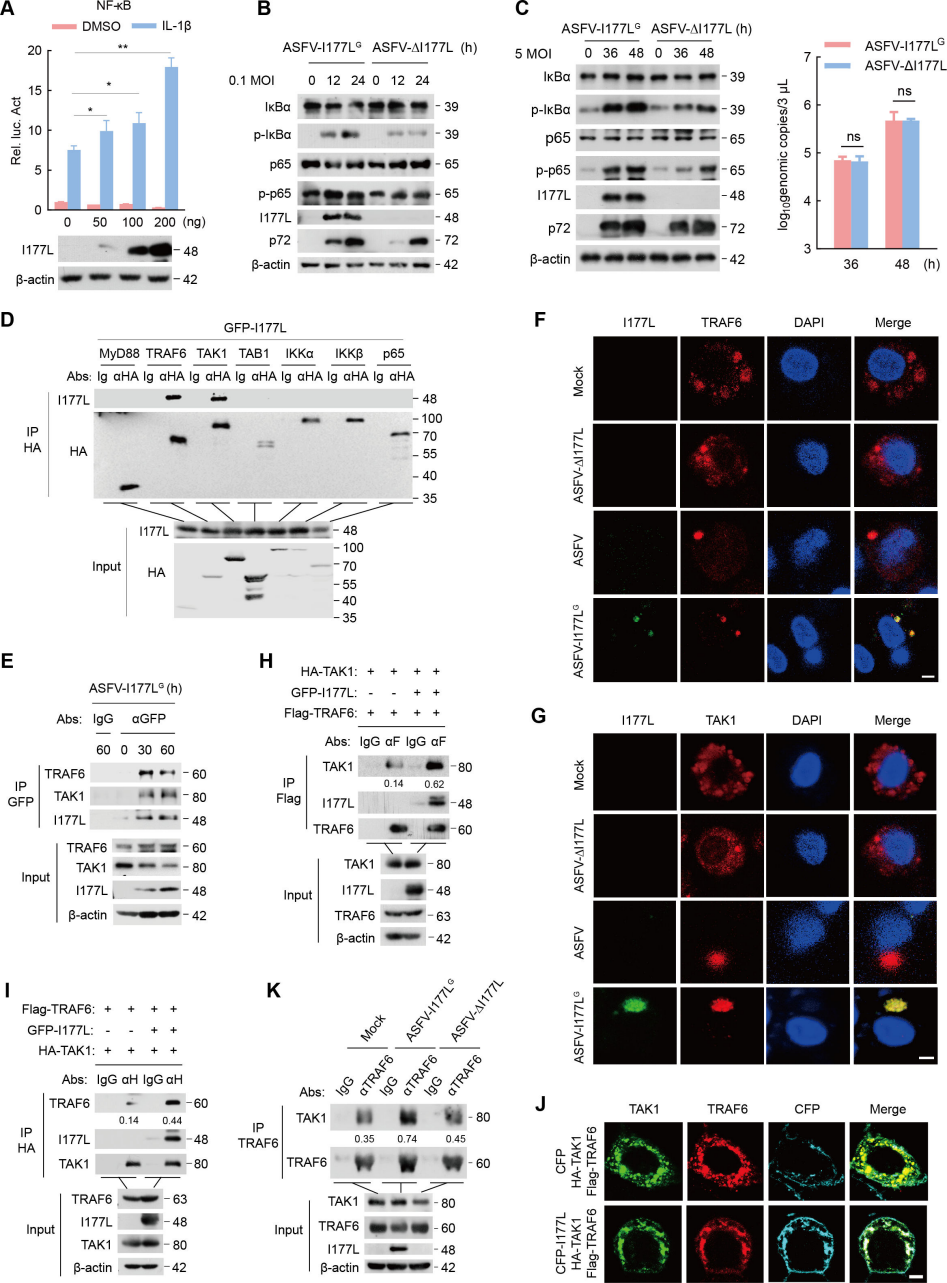
I177L activates the NF-κB signaling by enhancing TRAF6-TAK1 interaction. (A) HEK293T cells were transfected with increasing doses of I177L plasmids and NF-κB reporter plasmid, then stimulated by IL-1β (1 µg/ml) for 6 h; luciferase activity of I177L influence on NF-κB reporter was detected. (B) Western blot of extracts of PAMs infected with ASFV-I177L^G^ or ASFV-ΔI177L at 0.1 MOI as indicated. (C) Western blot of extracts of PAMs infected with ASFV-I177L^G^ or ASFV-ΔI177L at 5 MOI as indicated. Absolute qPCR of the genomic copies of ASFV-I177L^G^ and ASFV-ΔI177L. (D) The interaction between GFP-I177L and HA-tagged canonical NF-κB signaling components by immunoprecipitation with anti-HA and western blot with anti-HA or anti-GFP antibodies. (E) PAMs were infected with ASFV-I177L^G^ at 0.1 MOI for 30 or 60 h. The lysates were immunoprecipitated using anti-GFP antibodies and analyzed using anti-TRAF6 and anti-TAK1 antibodies. (F, G) Confocal microscopy of TRAF6 or TAK1 in mock PAMs, in ASFV-ΔI177L-infected PAMs, in wild-type ASFV-infected PAMs, and in ASFV-I177L^G^-infected PAMs. Scale bar, 5 µm. (H, I) HEK293T cells were co-transfected with indicated plasmids for 36 h. The lysates were immunoprecipitated using anti-Flag antibody (H) or anti-HA antibody (I). (J) Confocal microscopy of PAMs co-transfected with plasmids as indicated. Confocal images of TAK1 (green), TRAF6 (red), CFP, or I177L (cyan). Scale bar, 10 µm. (K) Co-immunoprecipitation and immunoblotting analysis of extracts of PAMs infected with ASFV-I177L^G^ or ASFV-ΔI177L as indicated.

Inflammatory response typically involves two steps: the NF-κB priming stage (signal 1) and the inflammasome activating stage (signal 2) (20, 21). The impact of ASFV on the NF-κB signaling was thus evaluated. Levels of phosphorylated-IκBα (p-IκBα) and phosphorylated-p65 (p-p65) increased over ASFV infection (Fig. S4A and B). BAY-11–7082 , an inhibitor of NF-κB by inhibiting phosphorylation of IκB-α (22), could significantly inhibit IL-1β mRNA expression, and IL-1β secretion is triggered by ASFV and LPS (Fig. S4C and D), suggesting that the NF-κB signaling is activated during ASFV-mediated inflammation. The influence of ASFV I177L on the NF-κB signaling was then explored. I177L dose-dependently enhanced IL-1β-triggered NF-κB reporter activity (Fig. 3A). Furthermore, the levels of p-IκBα and p-p65 in ASFV-I177L^G^-infected PAMs were higher than those in PAMs infected with ASFV-ΔI177L, regardless of the infection dose (Fig. 3B and C). Deficiency of I177L greatly reversed the inhibitory effect of BAY11-7082 on ASFV-triggered IL-1β mRNA expression and IL-1β production (Fig. S4E and F). These results suggest that ASFV I177L facilitates the NF-κB signaling pathway, thereby activating inflammation.

Next, we assessed which molecule of the canonical NF-κB pathway is regulated by I177L in NF-κB activating process. Co-immunoprecipitation assay confirmed that I177L could be precipitated by TRAF6 and TAK1, but not by Myd88, TAB1, IKKα, IKKβ, or p65 in HEK293T cells (Fig. 3D). Visualization by confocal microscopy revealed I177L colocalizing with TRAF6 and TAK1 in HeLa cells (Fig. S4G and H). The interaction between I177L and TRAF6/TAK1 was further determined in PAMs. Endogenous co-immunoprecipitation experiment showed that I177L was associated with TRAF6 and TAK1 in ASFV-I177L^G^-infected PAMs (Fig. 3E). Furthermore, in mock PAMs, TRAF6 and TAK1 were diffusely distributed in cytoplasm. In ASFV-I177L^G^-infected PAMs, TRAF6 and TAK1 were transformed from diffusion to aggregation to form specks in the cytoplasm and colocalized with I177L (Fig. 3F and G). Besides, we found that expression of TRAF6 and TAK1 remained basically unchanged whether in ASFV-infected PAMs, in ASFV-I177L^G^ or ASFV-ΔI177L-infected PAMs, or in LPS-stimulated PK15 cells transfected with increasing doses of GFP-I177L plasmid (Fig. S4I through K). Taken together, we reveal that ASFV I177L interacts with TRAF6 and TAK1 of NF-κB signalling without affecting their expression.

TRAF6 physically binds to TAK1, facilitating the activation of TAK1 and subsequent signal transduction (23). We further examined the effect of I177L on the formation of the TRAF6-TAK1 complex in HEK293T cells and PAMs. When I177L was presented, the interaction between TRAF6 and TAK1 was significantly enhanced (Fig. 3H and I). Meanwhile, TRAF6 and TAK1 that predictably colocalized (indicated in yellow) were co-localized with I177L in the presence of three proteins (indicated in white) (Fig. 3J). Notably, TRAF6 physically interacted with TAK1, and the binding was enhanced in ASFV-I177L^G^-infected PAMs but remained basically unchanged in ASFV-ΔI177L-infected PAMs (Fig. 3K). Collectively, we demonstrate that ASFV I177L facilitates the assembly of the TRAF6-TAK1 complex, thus stimulating the activation of the NF-κB signaling pathway.

### TRAF6 auto-ubiquitination is vital for I177L-mediated TRAF6-TAK1 complex assembly

**Fig 4:**
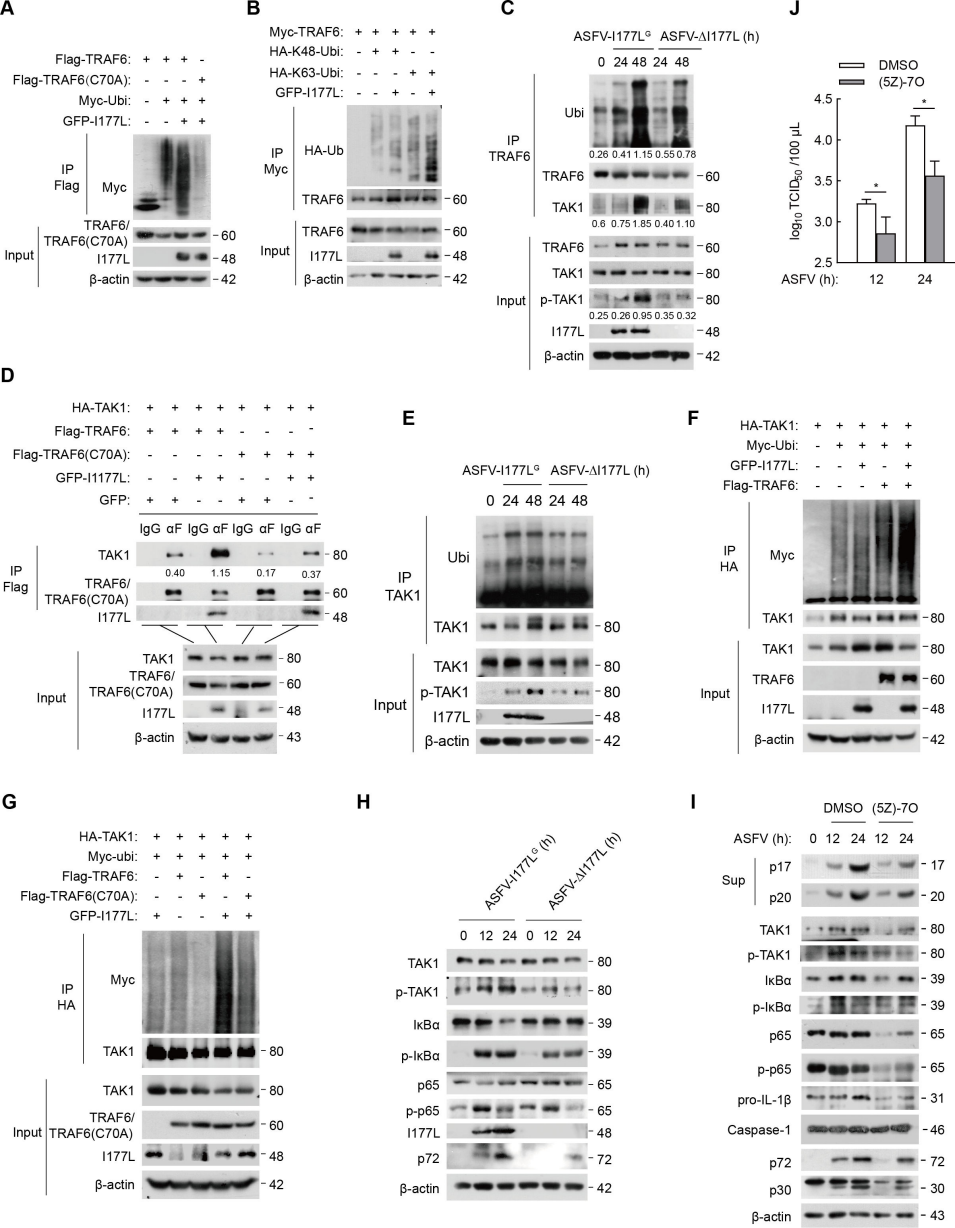
E3 ligase activity of TRAF6 is vital for I177L-mediated TRAF6/TAK1/NF-κB signaling activation. (A) Immunoprecipitation and western blot of HEK293T cells co-transfected with Flag-TRAF6/TRAF6(C70A), Myc-Ubi, and GFP-I177L plasmids for 36 h. (B) HEK293T cells were co-transfected with Myc-TRAF6, GFP-I177L and HA-K48-Ubi or HA-K63-Ubi for 36 h and then immunoprecipitated using anti-Myc antibody. (C) Immunoprecipitation of PAMs infected with ASFV-I177L^G^ or ASFV-ΔI177L at 0.1 MOI using anti-TRAF6 antibody and western blot using anti-Ubi and anti-TAK1 antibodies. (D) HEK293T cells were co-transfected with indicated plasmids. Co-immunoprecipitation and western blot were performed. (E) Immunoprecipitation of PAMs infected with ASFV-I177L^G^ or ASFV-ΔI177L using anti-TAK1 antibody and western blot analysis using anti-Ubi antibody. (F, G) HEK293T cells were co-transfected with the indicated plasmids for 36 h. The lysates were immunoprecipitated using anti-HA antibody. (H) Western blot of extracts of PAMs infected with ASFV-I177L^G^ or ASFV-ΔI177L as indicated. (I, J) PAMs were incubated with DMSO or (5Z)−7-Oxozeaenol (10 µM) for 2 h and then infected with ASFV as indicated. The lysates were detected using indicated antibodies (I), and ASFV titers were quantified by TCID_50_ (J). Data are mean (*n* = 3) ±s.e.m. *P < 0.05.

The discovery that I177L activates the NF-κB signaling pathway by enhancing TRAF6-TAK1 binding has inspired us to explore how I177L facilitates this binding. To answer this, we analyzed the role of I177L in TRAF6 activation and its interaction with TAK1. TRAF6 auto-ubiquitination contributes to its activation (24, 25). To determine whether I177L activates TRAF6, we investigate TRAF6 ubiquitination. In HEK293T cells, I177L increased TRAF6 auto-ubiquitination (Fig. 4A), and catalyzed TRAF6 ubiquitination with wild-type ubiquitin and ubiquitin-K63, but not with ubiquitin-K48 (Fig. 4B). Moreover, the level of TRAF6 ubiquitination was significantly augmented in the ASFV-I177L^G^-infected group, whereas they remained low in ASFV-ΔI177L-infected PAMs (Fig. 4C). These results suggest that I177L actively promotes TRAF6 auto-ubiquitination. Furthermore, as shown in Fig. 4C, TAK1 was more efficiently recruited by ubiquitinated TRAF6 in the ASFV-I177L^G^ group, with increased expression of p-TAK1, indicating that ubiquitinated TRAF6 fosters TAK1 recruitment and activation. Thus, our findings demonstrate that ASFV I177L enhances TRAF6-TAK1 binding by promoting TRAF6 K63-ubiquitination.

However, when TRAF6 was replaced with TRAF6(C70A), a mutant lacking E3 ligase activity due to a cysteine-to-alanine mutation, the activated effect of I177L on TRAF6 ubiquitination was suppressed (Fig. 4A). Additionally, TRAF6(C70A) still interacted with I177L and TAK1 when they presented together (Fig. S5A and B), but the basal interactions between TRAF6(C70A) and TAK1, along with the amplification of this interaction by I177L, were all diminished (Fig. 4D). Together, these observations indicate that I177L promotes TRAF6-TAK1 complex formation by enhancing TRAF6 auto-ubiquitination.

### I177L activates the TAK1/IκBα/p65 axis depending on the E3 ligase activity of TRAF6

TAK1 ubiquitination contributes to its phosphorylation and subsequent activation of downstream molecules (26, 27). To determine TAK1 activation upon ASFV infection, we detected the ubiquitination and phosphorylation of TAK1 in PAMs. We observed enhanced TAK1 ubiquitination following ASFV infection, a phenomenon that was reduced when TRAF6 expression was inhibited, with a similar trend to p-TAK1 expression (Fig. S5C and D). In addition, we found that TRAF6, but not TRAF6(C70A), enhanced TAK1 ubiquitination in HEK293T cells (Fig. S5E). These results suggest that TRAF6, specifically its E3 ubiquitin ligase activity, is essential to TAK1 activation.

We then examined the influence of I177L on TAK1 activation. In ASFV-I177L^G^-infected PAMs, we detected elevated TAK1 ubiquitination and corresponding phosphorylation compared with ASFV-ΔI177L-infected PAMs (Fig. 4E). This suggests that ASFV I177L enhances TAK1 activation. In HEK293T cells, TAK1 ubiquitination slightly increased due to GFP-I177L but was substantially boosted by Flag-TRAF6. Interestingly, when I177L and TRAF6 were co-introduced, TAK1 ubiquitination was not only increased by TRAF6 but further amplified by I177L (Fig. 4F). In contrast, the TRAF6(C70A) mutant, lacking E3 ubiquitin ligase activity, lost the ability to enhance TAK1 ubiquitination, and I177L’s promotion to TAK1 ubiquitination was further ablated (Fig. 4G). Therefore, I177L is demonstrated to facilitate TAK1 activation via TRAF6 E3 ubiquitin ligase activity.

TAK1 is involved in regulating JNK, p38 MAPK, and IKK signaling (28, 29). We next explored I177L’s impact on TAK1 downstream factors. Compared with ASFV-ΔI177L-infected PAMs, those infected with ASFV-I177L^G^ were demonstrated with increased levels of p-TAK1, p-IκBα, and p-p65 (Fig. 4H). This implies that I177L activates the NF-κB signaling pathway through cascade activation of the TAK1/IκBα/p65 axis.

To better understand the role of TAK1 in ASFV-mediated NF-κB signaling and inflammation, we used (5Z)−7-Oxozeaenol ((5Z)−7O), a TAK1 inhibitor (30). This treatment notably diminished both basal and phosphorylated expressions of TAK1, IκBα, and p65 and also reduced pro-IL-1β expression, IL-1β maturation, and Caspase-1 cleavage (Fig. 4I). Interestingly, (5Z)−7-Oxozeaenol ((5Z)−7O) also curtailed ASFV-p72 and p30 levels (the viral protein p72 is translated by ASFV B646L gene, which is the main component of ASFV icosahedron and is crucial for the formation of viral capsid; p30 protein is translated by ASFV CP204L gene, which is an early membrane protein of ASFV and is associated with virus invasion of host cells), as well as ASFV replication ability (Fig. 4J). In summary, (5Z)−7-oxozeaenol not only inhibits NF-κB signaling but also attenuates ASFV-induced inflammation and ASFV replication.

### I177L facilitates the NLRP3 inflammasome activation

**Fig 5:**
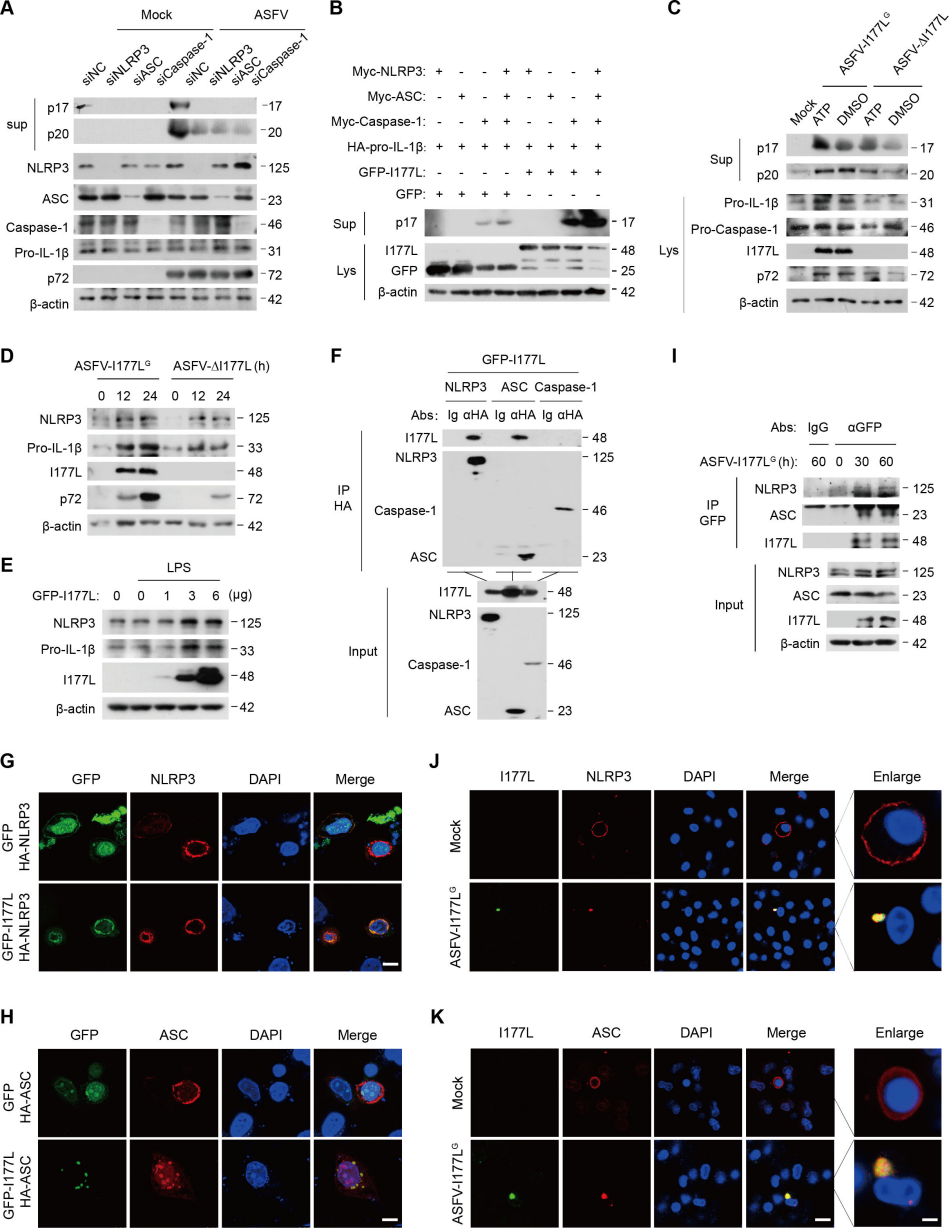
I177L interacts with NLRP3 and ASC. (A) Western blot of PAMs transfected with indicated siRNA for 48 h and then infected with ASFV at 0.1 MOI for 36 h. (B) Western blot of supernatants or extracts of HEK293T cells co-transfected with indicated plasmids. (C) Western blot of extracts of PAMs incubated with DMSO or ATP (5 mM, 1 h), then infected with ASFV-I177L^G^ or ASFV-ΔI177L at 0.1 MOI for 24 h. (D) PAMs were infected with ASFV-I177L^G^ or ASFV-ΔI177L at 0.1 MOI for 12 or 24 h. The lysates were detected using indicated antibodies. (E) Western blot of PK15 cells transfected with indicated plasmids and stimulated by LPS (0.1 µg/ml, 2 h). (F) The interaction between GFP-I177L and HA-tagged NLRP3 inflammasome members by immunoprecipitation with anti-HA antibody. (G, H) Confocal microscopy of HeLa cells transfected with indicated plasmids. I177L (green), NLRP3 (red), and ASC (red). Scale bar, 7.5 µm. (I) PAMs were infected with ASFV-I177L^G^ for 30 or 60 h. Western blot was performed using anti-NLRP3 and anti-ASC antibodies to detect co-immunoprecipitation samples. (J, K) PAMs were infected with ASFV-I177L^G^ at 0.1 MOI for 24 h. Confocal images of I177L (green) and NLRP3 (red), I177L (green), and ASC (red). Scale bar, 25 µm. Enlarge scale bar, 5 µm.

Inflammasome undergoes a functional role to induce the production and secretion of IL-1β in stimulus- or virus-induced inflammatory responses (31–33), and the NLRP3 inflammasome is the most investigated among them. Cytosolic DNA could be detected by the NLRP3 inflammasome (34). The role of NLRP3 inflammasome in ASFV-induced inflammatory responses was thus evaluated. IL-1β maturation and Caspase-1 cleavage triggered by ASFV were diminished when the expression of NLRP3, ASC, or Caspase-1 was interfered with PAMs (Fig. 5A). This suggests that NLRP3 is crucial for ASFV-mediated inflammatory responses and that ASFV induces inflammation by activating the NLRP3 inflammasome.

To further determine whether ASFV I177L promotes inflammation through the NLRP3 inflammasome, we engineered an “NLRP3 inflammasome” in HEK293T cells by co-transfecting plasmids that encoded NLRP3, ASC, pro-Caspase-1, and pro-IL-1β (35, 36). IL-1β maturation (p17) cleaved by individual Caspase-1 and NLRP3-ASC complex-enhanced Caspase-1 were significantly increased when I177L was present (Fig. 5B). ATP, a known stimulant for the NLRP3 inflammasome, initiated IL-1β maturation (p17) and Caspase-1 cleavage (p20) (37). In ATP-stimulated PAMs, ASFV-I177L^G^ led to higher levels of p17 and p20 than ASFV-ΔI177L (Fig. 5C). We also noted increased expression of NLRP3 and pro-IL-1β in PAMs infected with ASFV-I177L^G^ rather than with ASFV-ΔI177L (Fig. 5D) and in LPS-stimulated PK15 cells with overexpressing GFP-I177L plasmids (Fig. 5E). Taken together, we demonstrate that I177L facilitates inflammation activation and IL-1β production via activating the NLRP3 inflammasome.

Subsequently, we assessed which NLRP3 inflammasome component is required for I177L’s function. Co-immunoprecipitation (Co-IP) assay reveals that I177L was precipitated by NLRP3 and ASC, but not by Caspase-1 in HEK293T cells (Fig. 5F). When NLRP3 and ASC were present individually, they diffusely distributed in the cytoplasm. When NLRP3 and ASC presented with GFP-I177L, they colocalized with I177L, with ASC forming speck-like aggregates (Fig. 5G and H). Meanwhile, in ASFV-I177L^G^-infected PAMs, endogenous NLRP3 and ASC interacted with I177L (Fig. 5I). NLRP3 and ASC were dispersed in mock PAMs, whereas they formed spot-like aggregates that colocalized with I177L in ASFV-I177L^G^-infected PAMs (Fig. 5J and K). Thus, we demonstrate that ASFV I177L interacts with NLRP3 and ASC, thereby activating the NLRP3 inflammasome.

### I177L promotes NLRP3 inflammasome assembly

**Fig 6:**
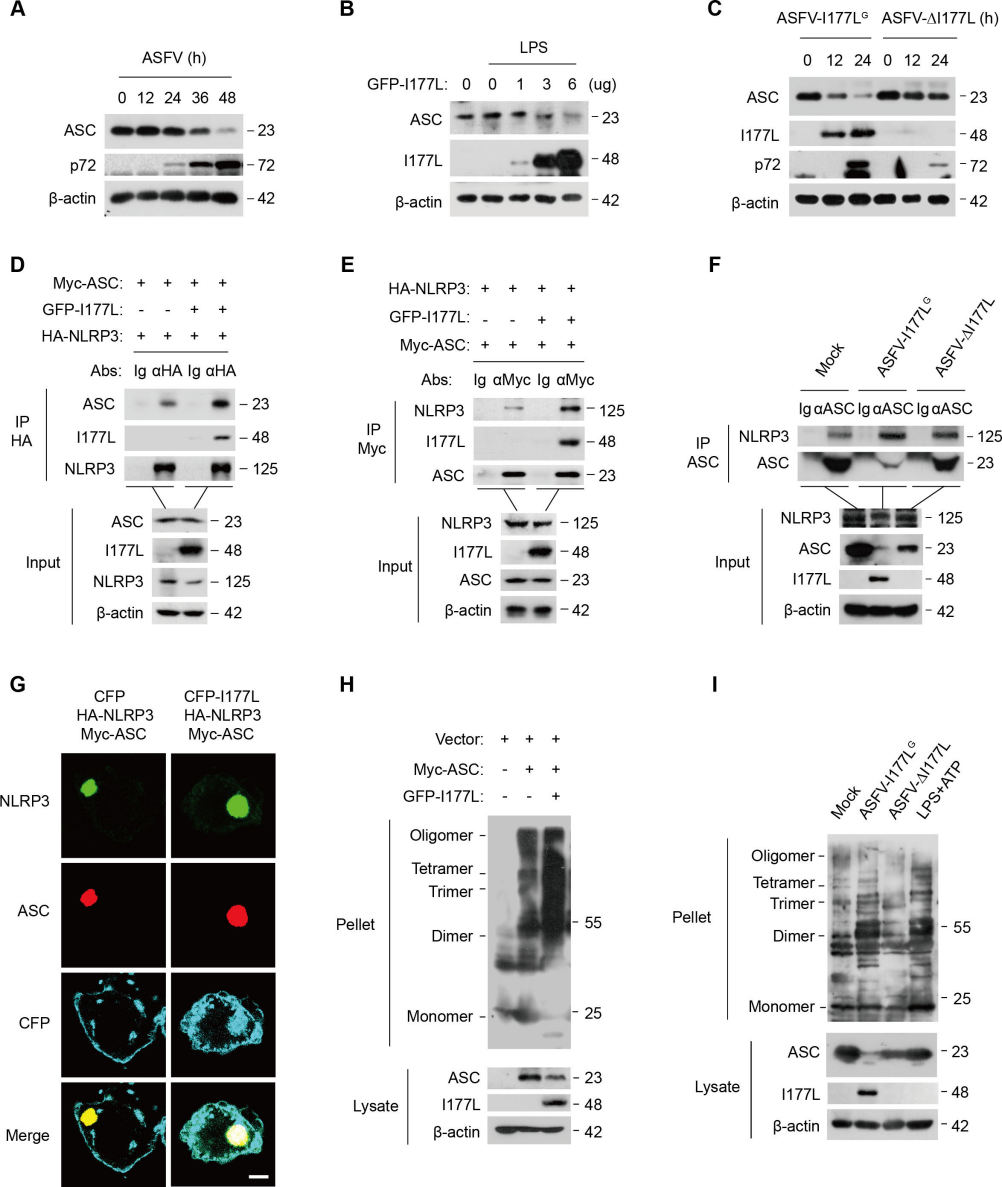
I177L enhances NLRP3 inflammasome activation. (A-C) Western blot of extracts using anti-ASC antibody. (A) PAMs were infected with ASFV at 0.1 MOI for 12, 24, 36, and 48 h. (B) PK15 cells were transfected with indicated plasmids and treated with LPS (0.1 µg/ml, 2 h). (C) PAMs were infected with ASFV-I177L^G^ or ASFV-ΔI177L as indicated. (D, E) Immunoprecipitation and western blot of HEK293T cells co-transfected with Myc-ASC, GFP-I177L, and HA-NLRP3 plasmids. (F) Immunoprecipitation of PAMs infected with ASFV-I177L^G^ or ASFV-ΔI177L at 0.1 MOI for 48 h using anti-ASC antibody. (G) Confocal microscopy of HeLa cells transfected with indicated plasmids. NLRP3 (green), ASC (red), and I177L (cyan). Scale bar, 10 µm. (H) HEK293T cells were co-transfected with indicated plasmids for 36 h. The lysates were subjected to ASC oligomerization assay (top) or western blot for ASC expression (bottom). (I) ASC oligomerization analysis of PAMs infected with ASFV-I177L^G^ or ASFV-ΔI177L at 0.1 MOI for 48 h or stimulated with LPS (0.1 µg/mL, 2 h) and ATP (5 mM, 1 h).

NLRP3 associates with ASC to form an active inflammasome complex (38). The association of I177L with the NLRP3 inflammasome was thus evaluated. To begin with, we assessed the influence of I177L on ASC expression to rule out its effect on inflammasome assembly. ASC expression decreased gradually with ASFV infection (Fig. 6A), and I177L prompted the degradation of ASC in a dose-dependent manner (Fig. 6B). Meanwhile, ASFV-I177L^G^ infection significantly inhibited ASC expression compared to ASFV-ΔI177L (Fig. 6C). These results imply that I177L inhibits ASC expression. Despite this, the basal interaction between NLRP3 and ASC was seen to be enhanced by I177L (Fig. 6D and E). The binding of ASC to NLRP3, existed in mock PAMs, was enhanced when PAMs were infected with a virus, with higher enhancement in ASFV-I177L^G^-infected PAMs than in ASFV-ΔI177L-infected PAMs (Fig. 6F). These results indicate that I177L enhances assembly of the NLRP3 inflammasome.

Next, we turned our attention to NLRP3 conformation and ASC specks within the inflammasome. In the absence of I177L, NLRP3 and ASC were co-localized to form a speck structure (indicated in yellow). In the presence of I177L, the three proteins (NLRP3, ASC, and I177L) were co-localized and aggregated as a larger spot (indicated in white) (Fig. 6G). This result suggests that I177L aids in the assembly of the NLRP3 inflammasome complex by forming a spot-like structure of “I177L-NLRP3-ASC.”

ASC oligomerization, as a distinctive sign of inflammasome activation (39), was finally investigated. ASC oligomerization formed individually was further enhanced by I177L (Fig. 6H). Moreover, ASC oligomerization was presented in mock PAMs and increased in PAMs stimulated by LPS and ATP or infected with ASFV-I177L^G^, but not affected in ASFV-ΔI177L-infected PAMs (Fig. 6I). These results indicate that ASFV I177L promotes ASC oligomerization, thereby activating the NLRP3 inflammasome.

### I177L deficiency diminishes ASFV-mediated inflammatory responses in pigs

**Fig 7:**
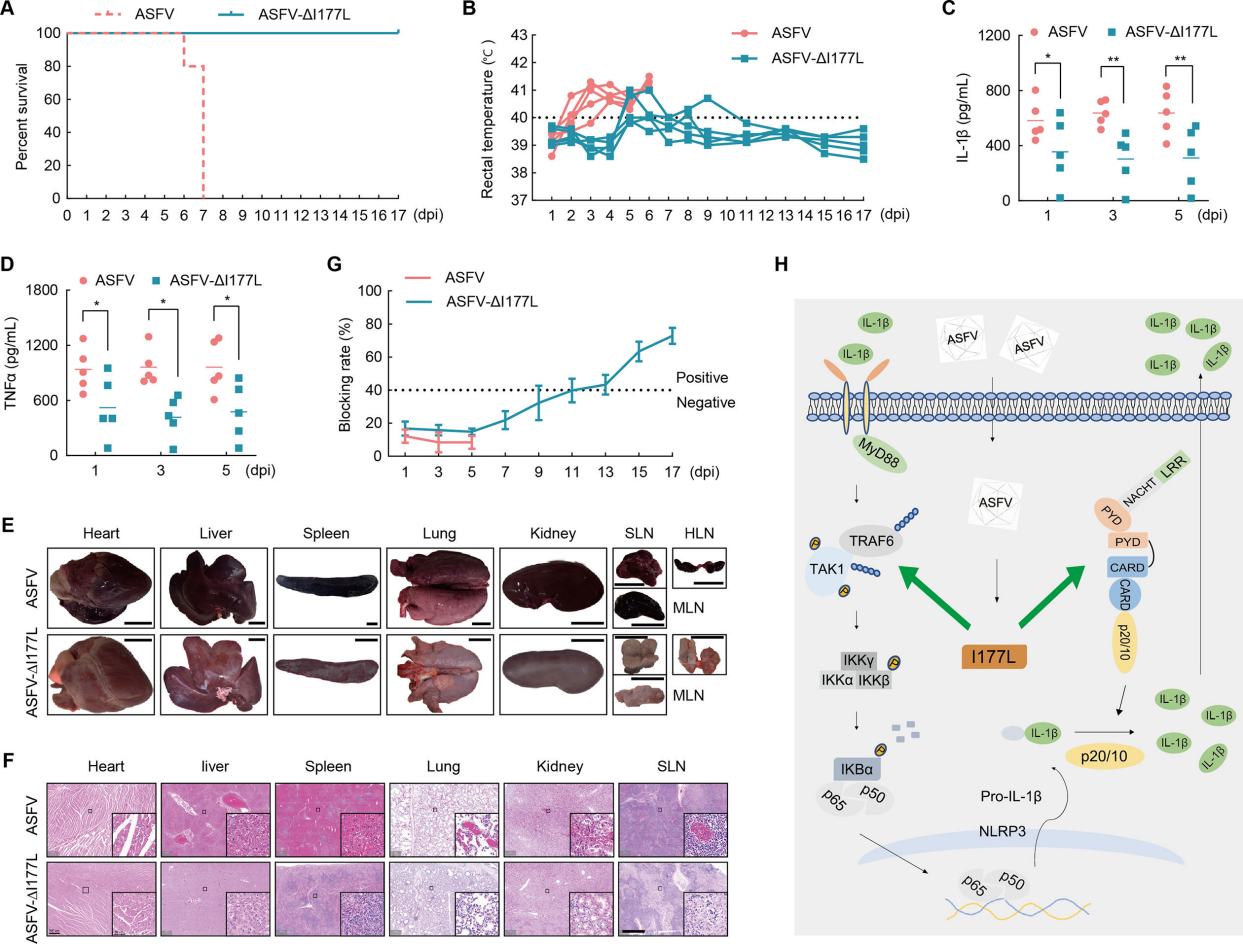
ASFV-mediated inflammatory responses in pigs were reduced after I177L deficiency. Pigs were intravenously injected with ASFV (*n* = 5, red) or ASFV-ΔI177L (*n* = 5, cyan) at 10^4^ HAD_50_ and observed the characteristics 19 days post-inoculation (dpi). (A, B) Survival rates and rectal temperatures were statistically analyzed. (C, D) ELISA analysis of IL-1β and TNFα levels in the sera of the two injected groups. (E, F) The morphology and H&E staining of the heart, liver, spleen, lung, kidney, and lymph node of two pigs respectively belong to the two groups. SLN, submandibular lymph node. HLN, gastrohepatic lymph node. MLN, mesenteric lymph node. Scale bar, 3 cm (E), 1 mm (F). (G) ELISA of ASFV p30 antibody blocking rates of the two groups. (H) Schematic diagram of the molecular mechanisms underlying ASFV-induced inflammation. Data are presented as mean  ±  s.d. of 5 pigs of each group, *P < 0.05, **P < 0.01.

To further evaluate the impact of ASFV-ΔI177L in pigs, we performed animal experiments by dividing pigs into two groups and intramuscularly injecting them with ASFV or ASFV-ΔI177L recombinant virus at a dose of 10^4^ HAD_50_. During the 19-day inoculation period, pigs injected with ASFV died or were euthanized 7 days post-inoculation (dpi) with persistent hyperpyrexia. Conversely, pigs suffering from ASFV-ΔI177L remained alive, and only one transiently suffered high fever during the observation period (Fig. 7A and B). IL-1β and TNF-α levels in serums of ASFV-ΔI177L-injected pigs were reduced compared with those in ASFV-infected pigs (Fig. 7C and D). In addition, ASFV-infected pigs displayed hyperemic organomegaly and microscopic tissue lesions marked with infiltration of red blood cells and depletion of parenchymal cells, but these phenomena were barely observed in the ASFV-ΔI177L infection group (Fig. 7E and F). The above results indicate that I177L deficiency mitigates severe inflammatory responses triggered by ASFV.

We also measured and compared viral titers in serums and swabs of the two infection groups. In ASFV-ΔI177L-injected pigs, viral titers remained low in blood, oral, nasal, and stool swabs, but in ASFV-infected pigs, viral titers continuously increased until death (Fig. S6A), suggesting that I177L is a critical virulence factor of ASFV. Pig survival is associated with the production of circulating virus-specific antibodies (40, 41). Notably, the blocking rate in ASFV-ΔI177L-infected pigs surpassed 40% from day 9 while remaining below this threshold in ASFV-infected pigs (Fig. 7G), indicating that the expression of ASFV-specific p30 antibodies in ASFV-ΔI177L-infected pigs provides protection against death.

The protective possibility of ASFV-ΔI177L against ASFV challenge was further investigated. We intramuscularly administered 10^2^ HAD_50_ ASFV into ASFV-ΔI177L-vaccinated pigs and another five healthy pigs, the latter serving as the unvaccinated control group. Unvaccinated pigs died or were euthanized 9 days post-challenge (dpc) with severe hyperpyrexia, whereas vaccinated pigs were all alive without high fever (Fig. S6B and C). Furthermore, the virus was partially eradicated in the blood, oral, nasal, and stool swabs, as well as in the heart, liver, spleen, lung, kidney, and three lymph nodes of ASFV-ΔI177L-vaccinated pigs (Fig. S6D through H). These results imply that ASFV-ΔI177L vaccination immunizes pigs against the ASFV challenge. The protective ability of ASFV-ΔI177L is probably due to the efficient production of ASFV p30 antibodies (Fig. S6I). The finding that deficiency of proinflammatory factor I177L can safeguard pigs against ASFV challenge is encouraging. It suggests that ASFV’s inflammation-related genes may be viable candidates for ASFV live-attenuated vaccines.

## DISCUSSION

African swine fever (ASF) can be categorized into five clinical forms based on symptoms: hyper-acute, acute, sub-acute, chronic, and unapparent. The virulent type II field strain is under extensive investigation, which typically causes acute infection with high mortality (42). Infections typically present with clinical symptoms such as high fever, vascular leakage, cardiomyopathy, lung injury, and an exaggerated and prolonged release of proinflammatory cytokines including TNFα, IL-1, and IL-6 (9, 10). ASFV infection prompts acute inflammatory responses.

During ASFV infection, the NF-κB signaling pathway was observed to be activated, which in turn promoted inflammation. Additionally, several reported ASFV proteins with inflammation-modulating functions were found to influence the NLRP3 inflammasome. Furthermore, there have been a few studies of I177L-deficient ASFVs (2, 43, 44), which induce low fever and slight pathology in infected pigs. In this study, an important mechanism of ASFV in activation of the inflammatory responses was demonstrated. Specifically, ASFV I177L enhances the formation of the TRAF6-TAK1 complex by boosting TRAF6 auto-ubiquitination. This enhancement initiates TAK1 activation through TRAF6, thus triggering subsequent phosphorylation of IκBα and p65. Consequently, I177L triggers activation of the NF-κB signaling, thus resulting in elevated levels of NLRP3 and pro-IL-1β, which are key components of the NLRP3 inflammasome. Furthermore, I177L increases the binding of NLRP3 to ASC and promotes ASC oligomerization, thereby driving the assembly and activation of the NLRP3 inflammasome. The truncated caspase-1 cleaves pro-IL-1β into mature IL-1β, indicating the onset of inflammation (Fig. 7H). Collective activation of the TRAF6-TAK1-NF-κB axis and the NLRP3 inflammasome substantiates the proinflammatory effect of ASFV.

TAK1 is involved in the regulation of multiple downstream signaling pathways, including NF-κB, JNK, p38, and TGF-β signaling (45, 46). Activity of these pathways is regulated by various upstream molecules, such as TRAF6, RIP1, and TAB1, and through post-translational modifications, including ubiquitination and phosphorylation (23, 47, 48). In this study, we found that ASFV I177L enhances TAK1 ubiquitination and phosphorylation, which depends on the E3 ubiquitin ligase activity of TRAF6. Phosphorylated TAK1 then facilitates the degradation of phosphorylated IκBα and the translocation of the p65/p50 heterodimer into the nucleus (49), ultimately activating the NF-κB signaling pathway. Thus, TAK1 plays a significantly important role in ASFV-activated NF-κB signaling and inflammation. Suppression of TAK1 expression using its small-molecule inhibitor, (5Z)-7-oxozeaenol, effectively dampened the NF-κB signaling and inflammation induced by ASFV and even inhibited ASFV replication. Generally, uncontrolled viral replication and excessive inflammation triggered by viral infection lead to host death (50). ASFV pathogenesis is probably due to uncontrolled ASFV replication induced by excessive activation of the NF-κB signaling and inflammasome. Therefore, TAK1 could be a novel candidate for relieving ASFV-mediated inflammation, which provides corroborating evidence that TAK1 is a potential therapeutic target in inflammatory diseases (51, 52). In addition, (5Z)-7-oxozeaenol may serve as a natural small molecule to mitigate ASFV pathopoiesis and could be considered for use in ASF control strategies.

We found that ASFV I177L activated inflammation by enhancing the NF-κB signaling and the NLRP3 inflammasome. However, several ASFV proteins, for example, MGF505-7R, H240R, and L83L, significantly inhibited activation of the NF-κB signaling and the NLRP3 inflammasome, thus suppressing ASFV-induced inflammation. These demonstrate that complex interactions among the two-type proteins probably exist, where different viral proteins have opposing effects on the regulation of the NF-κB signaling and the NLRP3 inflammasome. However, the interaction between I177L and which protein poses an inhibitory effect on ASFV-induced inflammation requires further exploration. Additionally, the specific mechanisms of the interaction are also unclear; hence, further investigation is needed to determine these inferences.

ASFV-ΔI177L induced strong protection against virulent virus challenge, which is similar to the former investigation using a 112 amino-acid I177L deleted virus from ASFV-G (ASFV derived from the 2007 Georgia isolate) (41, 53). However, the underlying mechanism behind this protection remains unclear. In this study, we have been observing a close relationship between the efficient production of ASFV-specific p30 antibodies and protection. Besides, lots of studies have shown that the cellular immune response contributes greatly to the protective immunity of the host against ASFV infection. Strong ASFV-specific IFN-γ responses and immunostaining of CD4^+^ T cells were detected in the spleens of vaccinated pigs (54). Activation of the immune cell subsets including Th1 and cytotoxic T cells, TNF-producing macrophages, CXCL10-expressing lymphocytes, and cross-presenting dendritic cells were involved in cross-protection against ASFV (55). The innate antibody responses and cellular responses collaborate in protection against ASFV.

Although this study provides essential insights, several areas could be enhanced. Including more recombinant viruses in the screening woulf benefit finding additional proinflammatory ASFV proteins. Although deleting I177L almost entirely blocks ASFV-mediated inflammation, we still detected TRAF6 and TAK1 ubiquitination levels in the ASFV-ΔI177L infection group. This suggests that there might be other cooperative mechanisms involved in ASFV-mediated inflammatory responses. Nevertheless, this study’s findings present the first and most important mechanism of ASFV-induced inflammation.

In conclusion, this study provides critical insights into the mechanism of ASFV-induced inflammation. It establishes a solid theoretical basis for managing host homeostasis and offers valuable information for developing small-molecule therapeutics targeting ASFV and advancing vaccine research.

## MATERIALS AND METHODS

### Reagents

LPS, ATP, and dextran sulfate sodium (DSS) were purchased from Sigma-Aldrich (St. Louis, MO, USA). The TAK1 inhibitor (5Z)-7-oxozeaenol was purchased from MedChem Express (Monmouth Junction, NJ, USA). Antibodies targeting the following proteins were purchased from Sigma-Aldrich (St. Louis, MO, USA): HA (H3663, 1:1000), Flag (F1804, 1:1000), Myc (M5546, 1:1000), and β-actin (A1978, 1:1000). Antibodies against TRAF6 (sc-8409, 1:500), GFP (sc-9996, 1:1000), and ubiquitin (AF0289, 1:500) were purchased from Santa Cruz Biotechnology (Dallas, TX, USA). The antibody against swine pro-IL-1β (ASC0912, 1:500) was obtained from Invitrogen (Carlsbad, CA, USA). Monoclonal antibodies against TAK1 (ab109526, 1:1000), p-TAK1 (ab109404, 1:500), and p65 (ab16502, 1:1000) were purchased from Abcam (Cambridge, UK). Antibodies against p-p65 (3033S, 1:500), IκBα (9242 s, 1:500), and p-IκBα (9246S, 1:500) were obtained from Cell Signaling Technology (Beverly, MA, USA). Rabbit anti-p72 pAb (1:2000), mouse anti-p30 pAb (1:500), rabbit anti-NLRP3 pAb, mouse anti-Caspase-1 p20 pAb, and mouse anti-ASC pAb were produced by immunizing the rabbits or cavia porcellus using corresponding recombinant proteins (56). The Swine IL-1β enzyme-linked immunosorbent assay (ELISA) kit (SEKP-0001–96T) was purchased from Solarbio (Beijing, China). HRP-conjugated secondary antibodies (1:10000) were purchased from Sigma-Aldrich (St. Louis, MO, USA) to visualize western blot bands.

### Cell cultures and viral strains

Primary PAMs were shed from swine lungs by repetitive lavage with gentle slapping and cultured in RPMI-1640 medium containing 10% fetal bovine serum (FBS) and 1% penicillin-streptomycin (Gibco). HEK293T and PK15 cells were purchased from ATCC (Manassas, VA, USA) and maintained in Dulbecco’s Modified Eagle’s medium (DMEM) with 10% FBS and 1% penicillin-streptomycin. Cells were cultured in an incubator at 37°C with 5% CO_2_. The ASFV strain (ASFV/CN/GS/2018) was isolated from the field and propagated in the laboratory; other recombinant viruses were engineered based on this parental strain.

### Plasmids

NLRP3-HA, ASC-HA, Caspase-1-HA, and IL-1β-HA were generated by cloning the swine NLRP3, ASC, Caspase-1, and IL-1β genes into the pEF1α-HA-puro vector at the *NheI* site. The pEF1α-HA-puro plasmid was generated by substituting the EGFP of pEF1α-EGFP-puro for HA tag using *NheI* and *SalI* endonucleases. ASFV I177L was subcloned into the pRK-GFP vector between *EcoRI* and *XbaI* sites to construct pRK-I177L-GFP. pRK-I177L-CFP was generated by replacing the GFP region of pRK-I177L-GFP with a CFP tag between the *XbaI and XhoI* sites. To construct pRK-CFP, I177L region of pRK-I177L-CFP was truncated at the *EcoRI* and *XbaI* sites. The pRK-GFP, Myc-TRAF6, HA-TRAF6, Flag-TRAF6, Flag-TRAF6(C70A), and Myc-Ubi plasmids were stored in the laboratory. The primers used for the construction are shown in Table S1. The accuracy of all plasmids was determined by Sanger sequencing.

### Recombinant virus construction and purification

ASFV strain used in our study was ASFV CN/GS/2018, which was provided by the Lanzhou Veterinary Research Institute, Chinese Academy of Agricultural Sciences. Recombinant viruses were constructed in this parental ASFV strain. Single-gene-deleted and GFP-fused recombinant ASFV was created by introducing foreign plasmids as templates into PAMs and then infecting these PAMs with parental ASFV. ASFV-ΔI177L transfer plasmid (ASFV I177L-p72GFP) was constructed by inserting elements of 800 bp upstream and downstream homologous arm of the corresponding gene and the middle p72GFPpA cassette into NdeI- and BglII-digested pEF1α-GFPpuro vector. The recombinant ASFV-I177L^G^ transfer plasmid named ASFV-I177LGFP, in which GFP was fused with I177L and inserted before the I177L TAA terminator codon. PAMs were transfected with these transfer plasmids using jetPRIME and were infected with ASFV at 0.5 MOI. The GFP-positive PAMs were picked and passed into fresh PAMs. After 6–8 rounds, the recombinant virus was purified, the purity was checked using PCR, and the accuracy of the modified region was amplified and sequenced. All primers used are listed in Table S2.

### Recombinant virus genome sequencing

The viral sequences of ASFV-I177L^G^ and ASFV-ΔI177L were determined through whole-genome sequencing and subsequent assembly of the extracted viral DNA. Viral DNA was extracted using MagAttract HMW DNA Kit (67563, QIAGEN) according to the manufacturer’s instructions. The purity and integrity of the extracted DNA were subsequently evaluated via agarose gel electrophoresis. Then, DNA samples were randomly sheared into fragments using an ultrasonic fragmentation system. The fragmented DNA was then processed in a series of steps including end repair, A-tailing, adapter ligation, purification, and PCR amplification to complete library preparation. The library was pooled and high-throughput sequenced on the Illumina NovaSeq PE150 platform.

### Genotyping PCR

Genotyping PCR was performed according to former instructions to identify whether the target ASFV gene was successively deleted. Among the Genotyping PCR system, the three primers were designed separately in deleted-gene, GFP cassette, and the common downstream. Genotyping PCR was performed using the PrimeSTAR Max DNA Polymerase (R045Q; Takara Bio) on T100 Thermal Cycler (Bio-Rad) using the following cycling conditions: 95°C for 3 min, 36 cycles of 95°C for 30 s, 60°C for 30 s, and 72°C for 60 s, 72°C for 5 min, 4°C for ∞. Detailed primer sequences are shown in Table S3.

### Interference experiments

The mRNA expressions of TRAF6, NLRP3, ASC, and Caspase-1 were interfered with using small-interfering RNA (siRNA); detailed siRNA sequences are shown in Table S4. siRNA was transfected in PAMs using jetPRIME of Polyplus Transfection (Illkirch, France) according to the manufacturer’s instructions. After 48 h of culture, PAMs were infected with ASFV at an MOI of 0.1 for the indicated time, followed by subsequent experiments.

### Quantitative PCR (qPCR)

Total mRNA was extracted using TRIzol reagent (15596–018, Invitrogen) according to the manufacturer’s instructions. Reverse transcription was conducted to convert mRNA into cDNA using Reverse Transcriptase M-MLV (RNase H-; 2641B; Takara Bio, Tokyo, Japan). Quantitative PCR of the indicated genes was performed using the TB Green reagent (RR820B; Takara Bio) on QuantStudio 5 applied biosystem (Thermo Fisher Scientific) using the following cycling conditions: 95°C for 2 min, 40 cycles of 95°C for 5 s, and 60°C for 30 s, with a melt curve cycle. QPCR primers used are summarized in Table S5.

For ASFV genomic copy detection, Triplex real-time PCR (rPCR) was applied. RPCR reactions were prepared with Pro Taq HS Premix Probe qPCR Kit (AG11704, ACCURATE BIOLOGY AG), along with heat-treated ASFV strain as the direct amplification template and primers and probe targeting ASFV B646L gene (encoding p72 protein). Reactions performed under the following conditions: 95°C for 2 min, 3 cycles of 95°C for 7 s and 60°C for 12 s, 40 cycles of 95°C for 6 s, and 58°C for 11 s. The primers and probe used in absolute qPCR were ASFV-F: ATAGAGATACAGCTCTTCCAG; ASFV-R: GTATGTAAGAGCTGCAGAAC; ASFV-probe: FAM-TATCGATAAGATTGAT-BHQ2.

### Western blot

Total protein of cultured cells was extracted using SDS-lysis buffer (10% 1M Tris-HCl, pH 6.8, 4% SDS, 20% glycerin, 2% mercaptoethanol-β, and suitable bromophenol blue for visualization). The protein concentration was determined using the BCA Protein Assay Kit (P0012S, Beyotime). Proteins in cell lysates were subjected to SDS-PAGE, transferred to a nitrocellulose filter membrane, blocked, and incubated with the indicated primary antibodies and the corresponding secondary antibodies. Protein bands were visualized using ChemiDoc XRS + Imaging system (Bio-Rad). Protein expression levels were standardized to parallel β-actin levels.

### ELISA analysis

IL-1β levels in animal sera and cultured cell supernatants and TNFα levels in animal sera were evaluated using the Porcine IL-1β ELISA kit (SEKP-0001, Solarbio) and the Porcine TNFα ELISA kit (SEKG-0001, Solarbio) following the manufacturer’s instructions. Absorbance was read using the SYNERGY multi-mode reader (BioTek). IL-1β and TNFα levels were proportional to Absorbance at 450 nm.

### Co-immunoprecipitation assay

Co-immunoprecipitation was performed to confirm protein-protein interactions and explore protein modifications, such as ubiquitination. HEK293T cells transfected with indicated plasmids or PAMs infected with the virus were collected and then lysed in lysis buffer (50 mM Tris at pH 7.5, 150 mM NaCl, 1% NP-40, and complete protease inhibitor cocktail tablets) at 4°C for 30 min followed by centrifugation at 12,000 g for 10 min. The lysates were incubated with Protein G-agarose and primary antibodies at 4°C overnight with gentle shaking. The beads were further washed with lysis buffer and centrifuged at 8,000 g for 30 s. Proteins were dissociated in 2 × SDS loading buffer with 100°C boiling. The indicated proteins and the modified forms were separated by SDS-PAGE, followed by immunoblotting analysis with indicated antibodies.

### Immunoprecipitation-mass spectrometry (IP-MS) assay

PAMs were infected with ASFV-I177L^G^ for 48 h, subsequently collected and lysed with NP40 lysis buffer. After incubation with protein G beads and GFP antibody, the immunoprecipitated proteins were separated using SDS-PAGE electrophoresis and visualized through silver staining (Protein Stains K, C500021, Sangon Biotech, Shanghai). The indicated protein bands were sliced and sent for mass spectrometry analysis.

### Confocal microscopy

The transfected HeLa cells or infected PAMs were washed three times using PBS and fixed with 4% paraformaldehyde for 1 h at 20-25°C or overnight at 4°C. After washing, the fixed cells were permeabilized with 0.1% Triton X-100 for 30 min and blocked with 5% bovine serum albumin in PBS for 1 h. The cells were then incubated with the indicated primary antibodies at 1:200 dilution overnight at 4°C, followed by incubation with mouse or rabbit special Dylight for 1 h at 20-25°C, preventing light. After three washes, the cells were incubated with a 4',6-diamidino-2-phenylindole (DAPI) solution for 10 min. Finally, the cells were visualized under a laser confocal microscope (TCS SP8; Leica, Germany).

### ASC oligomerization

ASC oligomerization was performed according to former instructions. PAMs or HEK293T cells were lysed by buffer (50 mM Tris, pH 7.5, 150 mM NaCl, 1% NP40, 5 mM EDTA, and 10% glycerol), then centrifuged at 6,000 rpm for 15 min, and the supernatants were separated to determine ASC expression. Then, the pellets were cross-linked with DSS (2 mM) at 37°C for 1 h. The cross-linked pellets were subjected to western blot to evaluate ASC oligomerization.

### Hematoxylin and eosin staining

The organs (heart, liver, spleen, lung, kidney, and submandibular lymph nodes) from virus-infected and control-healthy pigs were fixed by soaking in 4% paraformaldehyde at room temperature for at least 12 h, then embedded into paraffin wax, and cut into thin slices. The sections were finally stained with hematoxylin and eosin for better observation. The results were photographed and scanned using a Panoramic Imaging Scanner in Gansu RUIDE Biotechnology Co., LTD according to the manufacturer’s instructions. Images were scanned and processed using ZYFViewer software.

### Animal experiments

Large White-Duroc crossbred pigs at 4- to 5-week-old were obtained from a local farm with high biosecurity standards. Pigs were diagnosed to confirm the absence of ASFV and clinically common porcine viruses by serological testing. After a 3-day acclimatization period, pigs were divided into ASFV-infection and ASFV-ΔI177L-infection groups with five pigs per group. The two groups of pigs were intramuscularly injected with ASFV-CN/GS/2018 or ASFV-ΔI177L at a dose of 10^4^ HAD_50_. After 19 days post-inoculation (dpi), one survival pig of the ASFV-ΔI177L infection group was euthanized to collect tissue samples (heart, liver, spleen, lung, kidney, and lymph nodes) that were fixed in 4% paraformaldehyde for histology, and other survival pigs serving as the vaccinated group were intramuscularly challenged with 10^2^ HAD_50_ of parental ASFV, along with another five healthy pigs administered with the same dose of ASFV as unvaccinated control. At an extra 17 days post-challenge (dpc), pigs died or were euthanized using pentobarbital.

During the entire observed period, pigs’ survival and rectal temperature were recorded daily. Meanwhile, blood samples with EDTA and oral, rectal, and nasal swabs were collected at 2-day intervals to assess inflammatory response and viremia. In addition, tissue samples, including heart, liver, spleen, lung, kidney, and lymph nodes, were obtained and sampled by snap-freezing for virus detection.

### P30 antibody detection

P30 antibody levels in the sera of infected pigs were determined using a Blocking ELISA Kit (Lanzhou Shouyan Biotechnology) according to the manufacturer’s instructions. Absorbance at 450 nm of tested sera and negative control were read using a SYNERGY multi-mode reader (BioTek). The blocking rate was equal to 100 minus the ratio of OD_450 nm_ of tested sera to OD_450 nm_ of negative control. The higher blocking rate indicated a higher amount of p30 antibodies in the tested sera.

### Statistical analysis

The data of all in vitro experiments are represented as mean  ±  standard error of the mean (s.e.m.) of three independent experiments; data of in vivo experiments are represented as mean  ±  standard deviation (s.d.) of two independent experiments. Unpaired two-tailed Student’s *t*-test was used to determine significant differences, and statistical significance was set at 0.05. The mean values of the groups are illustrated using histograms in GraphPad Prism. Image J software was used to quantify western blot bands. Whenever possible, randomization and blinding strategies were used.

## Data Availability

All data supporting the findings in the paper are available within the paper and its supporting information. All relevant data are available from the authors.

## SUPPLEMENTAL MATERIAL

Text S1 jvi.02080-24-s0001.docx Genome sequence of recombinant ASFV with I177L fusing with GFP.

Text S2 jvi.02080-24-s0002.docx Genome sequence of ASFV-ΔI177L.

Supplemental material jvi.02080-24-s0003.pdf Figures S1 to S6; Tables S1 to S5.

Supplemental Excel file jvi.02080-24-s0004.xls Quality control results of raw data, including read death, GC(%), etc.

ASM does not own the copyrights to Supplemental Material that may be linked to, or accessed through, an article. The authors have granted ASM a non-exclusive, world-wide license to publish the Supplemental Material files. Please contact the corresponding author directly for reuse.
