# Supplementary material for: African swine fever virus I177L induces host inflammatory responses by facilitating the TRAF6-TAK1 axis and NLRP3 inflammasome assembly
Source: J Virol. 2025 Mar 26;99(4):e02080-24. doi: 10.1128/jvi.02080-24 (PMC11998506; doi:10.1128/jvi.02080-24)
Supplement: Text S1 — Genome sequence of recombinant ASFV with I177L fusing with GFP. [file jvi.02080-24-s0001.docx]

CAAATAGCTGTTTATCAGCAAATAGCTGTTTATCAGCAACAACTAATTATCAGCAAATGCTGCTTGTGGGTAAGCCAATAAATAGGCCATACCCTTGAAAGGAGAATTCAGTTTGATAAAAAAAATAACGAGTTTTCTAATAACCCGGTCAAGCATTTAATAAATGAATAGCATCACACGTCTGCATCGTGCATTCTGCCTGGAAAATGGGCCCATCTCTAATATATTTACACTGACGGTGAATCATACAGTGTTCCATGGGATAGCTATGCTCCTGTACAGGAGGCATATCTTTTAGAACTTTATTCTTACAAAGACCATCTTGACAAGCCCAGCAAAACCGACAATTTTTCACATATTGACACCAGTATCTAAGCTCCTCTTCCAGGGGATTGTCGGTCGAAAACCCCTGTAGACTAGCTAGGCCAGCTAGCAGCAAGCCGAGGTAACTAAAGAACCTCATTGTAGTGTTATATTACGAAAAAACATGTTAAAATTTGGAAAAAAAAGCCCTTTTTATAGATCTGGAAAAAAATTTTCACAAATCTAATTAAAAGCCTTACAGATCATCCTTTTCATAAATTTTCATTAACAATTGGTGGGGGCGGTTGTGAGGTACTGGATCAGAACAATCCATAACATGGTAATGTCCATTTCCTTCACCATATGTACACTGGTTATACCAGCGAGAAACCTCACAAGATGTCAAATAACTGTTCTCAACAATCAATGGCATGCTCTTATTCACCTTGTTCTTGCAAATTCCATGTGCACATTCCCAGCAAAACTTGCAGTTTTCCATGTAAGTACACCAGTATCCAAGTTCTTCTTGTGGAGGATTATCCGTTGAACGAAGATGCCCTCCTGCCTGAGTAGGTAGTCCTAAGACCTGATTGGCCAGCAGGCCAAGAATTTCCAAGAAGATCACCAACATTGCTACGGCTGGCTGAACAGCTGACTGAATAGCTAGCCAATTAGCAATCCACTGTACTTTTCATAAGATCATTTAAGATTCGGTCGGCATTTTTTCAATAGTTTGCTAGGAAAAAATTTTTAATTTTATAGATTCACACTACTTCATTCTCATGCTTAGGAAAAAAACAAACTAAATCTTACAATGTATCTGGATCTAATGAGAAGCTAGAATTCATCTTTTTTCAAATCCTTTCTGGGATGTTCATTCTTTTTCCACTCCTTCCTTGCAATTTTATAAGGATTCCAGGGCTTTGGGTCAGAACAGTTCATGCTATGGTAAATGTGCTCCTCCACATCATATCTACATAGGTCACCCCAGCGGGAAACCTCACAATATTTTACATAGTCATTCTCAATAATACTTGTGGAGTTGTTTCCCCAAACCCTGCTGGTACAAATCCCATCTTCACAATCCCAGCAGAACCGACAGCTTTCCACATAAGTGCACCAGTATCCAAGTTCATTCTCTGGGGGTTCAAATGTTAGAGGAAGATGTCCACCTACCCGAGTAGAAGTGGAGGATGAAACCAGGTTGCTACTGGCAGCAGGCCAATAATTCCCAGGATAATCACCAGCATTGTGCTCAACCAGCAACGGCTAGCAACGACTAGCAACTGACTAGCAATAGCTAGAAATGGCTAGCAATCAGTAGTAGCTAACGCTCTACTCTTTATAAGAAAATTTAAAATTCGATCAGATTTTTTTAGAATTGAGAATGAGTAAAACGCTTATATTCTTTTTCTAGCTAGAAAAAATAAGCTAGTTTAAGATAGGATTTCCCTTACTAACGGTTTAATTTTTAGCAAAGGTATAGGTAAAATACACTTGTACTTAGCTGCAAAAAAATAAGCTTATGGCGTATAAGCCGCCATAAGTTTATTTAATTAAAATGTTAAACTCTGTGATAAGACTGGAATCTTAGGCAGGTTTGATGTGGAGAACAGCATGAAATACAAGAGTGCCTGTTACACGAATAAGTTCTCTCAAACCGGGGATGGTCATACTCACATCTATGAAATCCTGGTCTAGGAGATTCATTTGATGCATGATGGCCGCACCCACACTTATGAGACACTGAAGAACTAAAGGGTTTAATTTTGATCTGAATGGTACTATATAGGATGATGGCAATCCATATCAAGATTAGAGCAATCAAAATCACCTCCTCAAGAAGCATGATGTAGCCTTAAATCTTAGACTGCTTTAAACCTTAGGCCCTCACTATCTTTAATGAAGGAGTTTAAATTTTGATCCCTTTTTCAAGACCCATTTAGAAGAAAAAAATAAAGTTTATATCAATCTAATTCATAAGTCATCTCTTTCATAAATCTTCATGTATTCTCTATGTGGATAAGTATGGGATGTTGGATTTGCGCAGTCCATTTGATGATCTGTATGGTTTTTGGGTCCTTCATAATAACTACATATACCATTCCAGCGGGAAACCGTGCAATTTATAATCCAGTCATTTTGATGAATAACTGGCCAATCTGTTTGAATCCTGTTTCGGCAGATACCGTGGACGCATTCCCAGCAAAAGTCACATTGGTTTGCGTAAGTGCACCAATAAACTAGCTCATGTTCAGGAGGATAACGGGTTGGTAGTAAATCTTCTAATTTACGTATAGGAGCGGCTTGAAGGACAACCACCCCCAGTAGTACTAGAATCAGTACCTTTATAGTGGCCACCCTACACTAGACCTCTAAGTTGAAGACAAAGAACTAAAATTTAGAGCCGTTTAATTACTACTAATAATTATATTTTTTATTGTCTACAATAGGATTCTATTAAAAAATAATGATTTTTACCAAGAAATATTTTTATAAAAAATTAATATATTTTGTAATAAACTTTATTTCCAATGACTGTTAAAATAAGGAAACTATCCTTAGTTAGTCGAGGAAGATGGTTAGGTTATTTCGCAATCCGATAAAATGTTTATTTTATCGTAGGTCTCGTAAAATCCAGGAAAAAAAATTACGGAAGAGTTTAAAAAAGCTAAATTTTTACCACCCTCCAGAAGATTGTTGTCAAATATATCGTTTGCTAGAAAATGTTCCTGGAGGAACTTACTTTATTACAGAAAATATGACGAATGATTTAATTATGGTCGTAAAGGATTCGGTGGATAAAAAAATTAAAAGCATTAAATTATATCTTCATGGAAGTTATATTAAGATTCATCAGCACTATTATATTAATATTTATATGTATCTTATGAGATATACCCAAATTTATAAATATCCCTTAATTTGTTTTAACAAATATTATAACATCTAAGTAAATATTCTTGGAATGGATTTTCTTATAGAATGGTTACAGGATATGTCAGCGACAGGCTTAATAACAAATTTGTTAATATTTTTTTGTTAAATAAATGAACAGGCCACCATTTAATATTACCCGTTGCAAAATAAGAAAAAAAAACAAACTTATAGTTACAAATCATCTTGATTAATCACATGTCGTTTTAACTCAATGAACCATTCTAAATCTTTGGGTTGTGAACAATTCATGTTATGTTGATAGTGTATCCTAAAGTGAGCTTCATACATACACCGGTCATGCCACCGGGAAACTGTACAATTAACAATATAATCATTTTGCGTAATAATAGGGTGGTCACTAAACACTTTATTTTTACACATTCCATCTTTACAGGTCCAGCAGAAGTCACAGTGTTTTGCATAGGTGCACCAGAACTTGAGATCCCTTTCAGGAGGCCTACGCATTTGCATCGGATTATCTGTGGAAAGAGGTAGGTTCATTATTATGTTCGTCATCAAAATTCCTAAAAGAACATAGAAGCCAAGAAAGATAAGCAGTCTTGTAGCGGCTTGCATTCGCATTCGTGAGTATTGTTTGCGAACATAGCTTATGAGAGCAATGGTAGCTATCATACAAAGACAAGTATGTTTGATATTCTCAGTGTCAATGACCCTATCCTCCTTTATTTGCATTAACTCATCAAACCAATCATAATATGTGGGATTTGTACAGCTCATGATGTGAAAGCGGCGTATCCTAGAGTCTGTAAAGTAGCTACATCTTTCATTATAGCGAGAAACCCTACATATTTGTATGTAATCATTTTTTTTGATGAGAGGGTGTTTTTCAAAAACCTTATTTTTACAAACCCCGTGTCGACAATTCCAGCAGAAGTCACACGATTTTGCATAGGTGCACCAATACTCAAGCTCTCTCTTTGGAGGTCTCCGGGTCATTGGTAACTCTCCTGTTCCTGGAAAAGATTGGCTTTGAATGACCGGCTGCATGACCGCCAGTACCAAAAGGAACACAATCACCTTCATGGCTGCAACTTATAAGTTGCAACTTATGGGTTGCAATACTGCAACGTATAGGTTGCACCTTATAGATCGCGACTCAAAAGGTATGAAAACCTTACCCTCAATACAGAATTTAAGTTTTAATCCTGATAATGTATCTGTTTATGAAAAAAAATTTTTTTTACTCATGTATGAATTCTTATACGAATCATAATATGTAGGCTGAGAATAATAATTCATATACGGTGTTGCGGGCTCAATAAAAATTTTGTTACCACAAAAAATAAATGCTGGATTTTTAAGATATATATCTATTAATGACTAAACCCTTTATACGCTGTAGGCTGAAAACAATCCATATAATGAATATACGGTGATTTGGGTTTAATAAAATACATACAACGGTCAAAATAGCGGGCAATACTACATTGACTAATATAATCATTTTGTTTAATAAGAGGCATATCATCCCACACTTTATTTTTACAAATACCGTTCCTACATTCCCAGCAGAAATCACAGTGTTTTCCATACGTGCACCAGTATTCAAGCTCTCTTATAGGAGGCGTATAAGTCCTTGGTAAATTTTGTTTCATATAAAAGATGGAAAGGGGTCGATTTAAACCCGGCTGAGATAGCCAAATCAAAATACATAAAAGAGCAAGTAGTTTCATAGTGGTATTTAGATGTAAATTTTTATAGTATGCAAATACAATGTAACCTACAAATACAATACTAAATACAAGGTAAAAACAACAATGTCTTATAATGATTGGCCAATAATCACCCCCCCCCCATTTTTCCATGAATATTTCATTTCCTGTATAGGGTCTAGGATGTGAACACTCCATGTTATGATGATTAGGCATTTTAACTGATATTTCATAAAAACACCCCCAGGAATTGCGATTAACTATACAGTTTACAATCGAATTCATCGAATTAGACTCATTTGTTATCTTATTTTTACAAATGCCATTTTGACAATCCCAGCAGAAGTCACAATTCTTTACATACGTACACCAATATGGAAGCTCCTCCTTAGGAGGATGCTGGGTTCTTGGTAATTCTGGTAATTCATGTGCAAGAATGAGGACTGAGTAGCCCAACAAAAGTCCTAGAACCTTCATGTTGTGTCCAAATGGCACCTGTCATTTTAAAAAAGATTTAAATTTTGCTACCGCAAAAAAAAATCCAGTATGTATTTTTTTAATACATATAATTATTGAAGTCTTATAAGATAAAGCCGAGAACACTATATTTTGTATAGATGATGTATCCGGTATTCAAACTCTCTTATAAGTACATGTAGGAAATGGTCAATTATTCAAGATTGGCTGAGATAACAACAAAACCAAAATACTCAAAAGCATAAGTAATTTCATGGTTGTACTCAGTCGTAGATTTTTGCAGATCGCAAATGCAACGCAACCAGCAAATACAAAGCTAAATACAAGGTAAAAACAATAATACCTTATAATGATTGGCCAATTCTTATCCCTCCATTTTTCCATGAACATTTCATGTTCATAAAGTCTAGGATACGAACAACATTTCATGCTATGATGATTAGGTATTTTAAGTGATATTTCATAAAAACACCACGGGGTTGTTGGTGATTGATAGGTAAGAATAAGGATGGTTGAATAACCTAGTAAAAGTCCTAGAAAAACCTTCATATTGCGTTCATACCACAGATGTTATTTAAAAAAAATATAAATTTTACAGTATGTGATATACACATACCACAAAAATGTTCTTATATTAACTAAAATATGTGGGCAGAGAGCAATTCATATAATGAATATATGGTATTTTAGGCTCAATAAAGTACATACAACGATCAATAAAACGGGTAATACTACATTTACTGATGTAATCATTTTGAACAATAAGAGGCATATCATCCAAAACCTTATTTTTACAAATACCATTCTTACAATCCCAGCAGAAATCACAGTGTTTTCCATACGTACACCAATATTCAAGTTCTCTCATAGGAGGCGTATAGGTCCTTGGTAAAATTTGTTTCGTATAAAAGATGGAAAGGGGTCGATTTAAAACTGGCTGTGCTAACCAAACCAAAATACTCAAAAGAACGAAAAGTTTCATGGTTGTACTCAGACGCAGATTCTTACAAAGCGCACATACAAAGCAGCCTGTATATGCAATACCAATGATGAAATAGAGACAGTATTGCTTTATAGATAATTGTTGATGGTCACCCCCCCCCCCCCCATGTTTGCATGAATATTTCATTTCCTGTATAGGGTCTAGGATGTAAACATTCCATGCTAAAGTGATTAGGCATTTTAGATGAAATTTCATATAAACAGGATTGAGTCTTGGAATCACGGAAAACTCTACAGTTTACAATAGAATGATTGGAGTCAATGAAACGAGATTCCGTTATCTTATTTTTGCAAATGCCATCTTGACAGTCCCAACAGAAATCGCATTGTGGTACATACGTACACCAATATGAAAGCTCACTCTTGGGAGGATGCTGGGTTCTTGGTAAGTCTGGTAATTCATGTGCGAGAATGAGGACTGAGTAGCCCAACAAAAGTCCCAGAAGAACCTTCATGTTGCGTCTAAATGACACCTGCACTTACAAAAAAAAATTTAAATTTTGAATATAACACAAAAAAACCACCTTAAAATTTCTTATATTATTTCTTGGATCTGCCCCGACGTCATACAATGTATTAAAATTATAGACCAATCATCTTTTTGTATATAGGCTAATCATCTTTATATATAGATTTTAGATGTTTGCTTGTTGTATCAACTTAACTGCTAGCGAAGAAAATGGATAAAAACTTTCTGTATTTTTATAGGTTGAAATCATTTTATGCACATCGCTAGGATCTAATATTTTATTTTGAAGAACCGAATGTGGGCTTAAAATTTTTTTCTTAGAAAAAAGTAGAATCATAATATTGCTATGTTTTTGTTTAATGATTTCTTGTATCTTTTTTGTATACGGGTTGGCACCCAAACCTATACAAAAATATACATTACTCAAATAACTACCTTCTATACATAATCTTTTTTCCCCACGTATTTTCCTATTTATTTCCCTATTTATGGAATTAAAGGATATCAATCTCTCTAAGGCACGGTCAAGGTCTGCGCCTAAGGCAAAACAATAATATATACCTAATTTATTCCCAGGGCGTGCACAGGCAAGAAACATCATGACGTTTAGCCCTAAACGTATATTTTCCTGAAAATACGCATGATGAACTTCATCAATATTACCTAAGTATATGGCCGTTTGTAAACGCCAAAGATCTAAATGAGGAAATTTTTTACTAAGATAATGAATAGGTTTTGTGAGATTAAAATCTATGGCGAACTTATACCAAAATTTTAATACAAGTGTATTTCTCGTCATTTCTTCTTCTTTTTCATCTAAATATAAGATAAAACGATTGTAAACAAAGTCTATCAATAGGTGAAAATCATTGCTATTAAAGCTGTCGAGAATCAAAATATTGTCATAATAAATTTCGATCGCCAGTAAAACCTTTTTTCGTTTGACGAGATAAACAAACATATTATACAACCCTACATCTAAAAATTCTGGATTGGCTCCTAGTTGGATACACAGGTCTTTAGTCTGCTTCGTTTTGGCACACATGATGCCAAAATTAATATCAGCACCCCATAAAACAAATAACTTGATTAGATCAGTCTGGTTTTCCTTCACAGCTTTTACTAAGGCTCTGTCAAGCTCATAGCTGTCGACATCAGAGCATGACATAGAGCCACCGGTTACCATTTTACATTGCTTACAAAAACCTATGGGTCCGTTTTCCCACCATAGTCCAAGCTGTTGTAGAATAAAAATATCATCCTCATGATAATTTGAAAAAGCCTTGGTTTCTATCAAGACTTTTTTTGTAAGAACCTGTAAAGAGTTCATCGTATTATTATGAATAACAGGAGTAAACGTAATCAATTATAAAAGTGATTTTTTCGAAAAAAACTTTAGATGGTTGAAAATGATAATGTACATGTTCATACAAAAAATAGATGCAGTGATGTCTAAAATCAAAATTTAATTTTCTATGTAAAAAGTACAGACTTACTTATTTGGGTTAAATTGTTTATTTTAAACTTTAATTAACCGTTTGAGTTAGCGATGTTTGATTTATCTTCCATACTCATCCGGGGGGGGTCCTTATAGCTCTGACATTATTGTGGATTATTGAATATAATGAATACTTCATAGATGCTAAACATTTTAATAGTAGTTCTGAGGCTTAATTGTACTCTATAAATTTATAAAAACTTTTTGATCAAAATTTAATTTCTTATAAAAAGAGTACAGACGTCGCTTGTTTAAGCTTCATCATGTTTCATTCATTACTTTCTACAATTACGGGGGGGGGGAGTCCCCTCATAGCTTTAGTATTGCTATGGTTTACTAATTATTATGTAGAATTTATAGAAGCATATGTACCTGAAAGTATACCTACTCTATAAAATTAAATAATTTCAGTATATTTTTTTTATGAATAGAACGGAAATGATATAAAAATAATTTAATATTGCAAAAAAAATTCATAATGTTGGTATGTATTATAAACATAATAGCATGTGTAATTTATAAACTGACTCCTCTATATAATTATTAGATGAGGTACCAACCTACTTATGATATGCCGATGATAGATATTGTATACTATAAAACAAAATTATTTTAAATGTATTCATGGATACATTATAACATTTTTACCGCAAATTGTCTCTCAGCGAAGAAAATGAATGAAACGTTTCTGTATATTCATAGGTTGAAATTATTTTACGCACTTCACTAGGTTCTAATATTTTCTTATGAAGTATTGAATGGGGGCTTAAAAGTCCTTTCTTAAAAAGAAGTTTCATCATAACATTCTTTTCTTGTCTAAGAAGAGTTTCTTGTATTTTTTTTGTATAAGGATTGGCACCCAAACTTATACAAAAATGTACATTACTCCAAATACCATAATTTGAAAAGAAAGTTATTTCCCTATTTACTTCATGATTAATGAAACCTATCAACGTCTCTAAGGCCGTATTGATATTTGCGCCTAAGGCAAAACAATAGTATATACCCAATTTATTTTGAGGGTACATACAAGCAAGCGACATCATGTCATTTGGATCTAAACGTATATTTTCCTGAAAATATGCATGATGGATTTCATCAACATTACCTAAGTATACAGCCGTTTTTAAACGCCAATAATCTAGGTGAGGAAATTTCTTACTAAGAAAACGAATAGGTTTTATAAGATTAAACTCTATGGCGATCTTAAACCAAAATTTTAATACATATGTATTTTTTATCATTTTTTCTTTTTCATCTAAATTTAAGATAAAACGATTGTAAATAAAGTCTATCAACACGTAAAAATCATGGCTATCAAAACTGTCGAGAATCGAAATATTGTCATAATAAATATCTATAGCTAATAAGACCTTTTGTTGTTTAATTAGATCAACAAACATATTATACAACCCTACATCTAAAAATTTTGGATCAGCTCCTAGTTGAATACACAGAACTTTCGTCCTTTCCGTCTTGGCACATATGATGCCATAATTAATGTTGGCACCCCATAAAACAAATAACTTGATTAGATCAGTCTGGTTTTTCTTCACAGCCCTCACCAAGGCTCTGTCAAGCTCATAGCTGTCAACATCAGAACATGACATAGAGCCACTGGTTACCATTTTACATTGTTTACAAAAACCTATGGGTCCGTTTTCCCACCATAATCCAAGCTGCTGTAAAATAAAAATATCATCCTCATGATAATTTGAAAAAGCCTTGTTTTCTATCAAGACTTTTTTTGTAAGAACCTGTAAAGAATTCATCGTATTATCATGAATGAAAGCAGTAAATGTAATCAATTATAAAATTGACTTATTGAAGAGAAATGTTAAATGAGTGAAATCGGTGTTTATGATGATGTACATGATCATACGAAGAAACACGTTCACTGGTGTCCATGATCAAAATTTAATGTTTTACGTAAAAAGTACAGATGTTAACTGTTTAGTTTAAACATAAATTTAACCTTTAGTTTAAACCCTAGTTAATGATGTTTAATATTTCTTCTATACTCATTCAGGGAAGTGTAATGATTCTAATACTGTTGTTATGGATTATTAATGAAAACTTTACAGATGCTGGAGGGAATAATTTTAATCATACTGTTTTAATGTAGCTATATAAGCTTTCATCAAAATTTAATTTTTTTTTATAAAAATACACGAATTAAACTAAAGTCTAAACTTTAGTTTGACTATTTGAGTTAATGATGCTTAACTTATCTTCCATGCTTATCAAGGGGGGGTCCTAATAGTTTTGATACTATTGTTGTGGATTGTTGAATATAATAAATACTTTATAGATGCTGAAATGTTTGAAAATAATAGTACATCAATGTTGTAAGTTTGATCAAAATTTAATTTCTCATAAAAAAGGTACACATCAACATTGCTCATTTAAGTTTCATGATGTTTGATTCATTACTTCCTACAATTACTGGGGGGGGGTCTTTAATAGCTTTAGCATTGTTATGGTTTGCTGACTATTATGTAGAATTCATAGAAGCACGTTTAGATAGTAATATCACTGCAGTGTAGATTATGAAATACATACTAAACTAATTTCAGTATATTTTTTTTGTTCATATAAGTTAAGGTACAAAAATGATTAAACATTGCAAAAAAAGAAAATCACAATGCTATTATACATAGTGATCATAGTGGCTTGTATCATTTCTAAACTAGTTCCAAATGAATATTGGGCAATACATCTATTTTTTATCATTATGATTTTTATGGTATATATGTATGAAAAGTTAGATATACATCAAAAATCTCAGTTCTGGAATTATACCATGTCAGGCTTATCTGGACATAACGTACAGGTAACATGTAAGTGTTACTAAATACTATGAAGTATCTATTTTTTTTTGTTGTAAAAAAAAGAACTTGATAGTATTTTTTAAAAAATAAAATAATTAATTGTACGTCAACTTCCTTATTTTATTCTTTAAAAATAACTCGTAAGTATTATTTATCTATTTTTTGAAAAAATAGATGTAATCGGTTTCATCATTTAGGTGTGTATTTCTTTTTAGCATCTATCAAGAATTCATTGTTTAGTGATATGAAAACAATGAATGATCATTATCTTCTATTTAACAACCACCTAAATAAATGAACGTCTTTTTCATCTTAACTGATTACCAAAAGTTATTTTGCGAAAAGGCATACATATGATCAATATCAGACCTACAATGAATATTTCCATAATATCCCTTTATTGTAATAATTCTATTTTTGCATTCCGATATCTCATCATCTGTGCTATTATATGTTTCCATAACTGTTTCATCATCAAACATAAATCCTGTTAAATAGGCAAAAGACTTTAATCCCGGATAGATTTTTACCATTTTCCTGAGAGCCGTGTATAGCTTGTAATAAATGGCCAAAAATATGCAATAAAGCGTAGAAAGAGAGTAATTTTTGGCATAAAAGATTTTGAAGGTTTGATGAATGGCTAAATCGCATATAATATAAGATACGATTTTAAAGCGCACCTGTTCACGCAGATTTGTTGAAAAATTCGTGGAAAGATTTAACAAATAAAAGGTTATTAATAGTTGCTCATCATTCCCCTTATACGACATCGTCAGACGCTCTAATATTTTACTACTAGGCACATCTGCCACATGTTGAACATTTAAAGCCTGTTCTTCTTCTGTGTTACGGCAAAAGAGCCGTGCGTATTCAGGTGAAGCTCCCCAGGATAACAACGTCCTTGCTACGGCTAAATTTTTTTTGACGATGACTTTTATCAGAAATAAGTCTTTATTTTTGCATTGATCACTATGCGAATTTGTATAGTTGACGCCGTTGCATTGAGTACATTGATATAATGTTTTACAATTCCAGCGTAGCCCTAAATGGTATAAAAGAACTGTATTTTCGACATAAGCATGCTGATTAACGATGTTTTTGAGACAACACGTCGTTAAGGACACCATATTGTCTCCAATTTGTTAGATAAAAGTCTTTACTAAAAAAATAGATTTTTAGTTTTAACAATCGAGATTTTATTATTTGGATGCATCATCAAAAAGATTTATAAGTATAAGAGGTTGTATAAGAAAAAAAATGATGTTATACTATTTATGTTAAAATTTAATTTATCATATAAAAAGTACAGATTTAATCAGTTGGTTAAACTATTTAGTTAATTAAACTAAATAGTTTAACCATTTAGTCAGACTACTTGGTTAGCAATGTTTGAGCTTTCTTCCATTCTTATCCGGGGGGGGGGTCCTAATCGTTCTAATACTATTGTGGATAGTTGAATATAATGAAGACTTTATAGATGCTATAATGATGAATTCTAGTATGCCTGTATAAAATAATTAACCTTTTTGATCAAAATTTAATTTTTTTATAAAAAGCTACAGAGTAGTGTTTTATTAAACGTGGCTTATTTAAAAGTTACACAATGTTAAAATCTCTACTTACTTTAATTCTTTGTGGGGTTTTATTAACTTTATCCATATTATGGCTTACTACTTACCATGTAGAACTTATAGAGGCAATAGATGATTTCTACGACTGAAATATAGAATAGTCCATTTTCTATTTGTAAAATAATGATTTATATTCTTTCCTAAAAATGATACTTTATATGGTTTGAAAACAAATATTAACAACTTGATTTTTTTTTCTATAAATAAACTATAAATGAAAATAGTAAAACTCATAGAGTCTTATAAGTGAACATCTTCATAATGTTACTCAAACGTTGGACTATTAAAAAATATTCCGTGTGCATTATTGCTTTTAATCAGTATGATTACTTTATACGAAGCCGCTATTAAAACGCTTATCACACACCGAAAACAAATTTTAAAACACCCCGATAGCCGTGAAATTTTACTAGCTTTGGGGTTGTACTGGGATAAAACTCATATTCTTGTTAAATGTCGTGAATGTGGGAATATGAGTCTTACCGGAAAACACAGTACAAAATGTATTAACATTAATTGTCTACTTATTCTTGCCATAAAAAAAAAGAATAAGCGTATTGTTGATACCTTGATAGGAATGGGCGCGGATGTAACATATATACATCTTTTAAAGAATAAGATAAAACTGTCATACAACCAGCTGTCTATGCTTAAAAGCAACTCGCAGATTTCATTGAAGGAGCTTCATGCTATATGCTATCTTTTATATGGTCGGCTTCCCAAAAAAATTAAACAAGGGATGCGACTGTGTAAAACAATGGCGGGACTATGTGGTGAACTTTTATGTGCATTTTTAGCTCCGTAAATGATAATATGTATTTAAAACAAACAGATATTACCAAAATATATTCTATGTACATAATATCTGGGAAATTATTTTTTTTTCTCATACCCTTAAATATAAAAATATTGGGTTTCTTCACTAAACTTTAGAGGTAAAAATTTTTCTTTGTTTTGCACCATCATGTATGGGTTTAGGCTGTCCCAGGGATTGTTTATTTGAATATTTCCTAAATAGGAACACAACGCCATGATCATATATCTTTCATTCTGGTAAGCTTTTTGATACATCTTCAAAGATGCCGTACCTCCGAGTGTGTAACAGCAAACAAACGTCCGTACTTTTCCATGGGTCGCAGCCCATTCCATTCCGTAGCTCAGCATCTTTTGCTGTATTTTTTTATTCGCTTTATAAAAAAAGTTTTTCATCCATTCCACGTTCTCATAAAAACAGGCACTTAAAAAGAGCACTAGGGGTAGTGTAGTCTTATTATAGAATGTAGGAATGTATGTTTTAGTTATTTTTTTCAACGCGTGTTCCATACTATGTTTTACCGCCATAAAAATACAAAACCAATACCAACTTTTTCTATAAAAGGTTTTGCTGTACACATATAAACGAGCAAAATATATTTCAAACTCTATATTCTTTTTATAAAAAAACTCGAGACAGTCGTTTATGTTACGACTTTTTCTAAATACCTCAAAAACAGTAATTAATTCACTGTCGCTGTGGAAATGTTCGTAAGCTAACTGTTTAATGTCTTTAGGGGTCAATTCTTTTTTTGGGAGCAGTGGTTTGAGATTCGGCAAAGGTCGTCTAAAGTAGTGAGCGAACTTTTCATTCGCTCCCCAACACAAAAGCCGATAAGCCAGCATGTAGTTATCACGTTTTACCGCGTAAATAAGCAAATAGTTTATATTGATACATGTACCATGTTGCTGCCCGTTTGGACATATGTTGCCGCATTCTGAACACTTATGAATGAGATCATAGTTCTTACAACATAACCCCAAACGGGTTAGTACTTCTTTGTCACGTTTTAAAAACTCGACATGATTCTTTAATGTTAATGCTTTGAGCGCAATGTTAAATAAACTCTGCATTTTATTAAAATGAGGTTAGTATCATGTTTTAGTATAAAATTTAGCGGCTGTTTACATAATGCTAAATAAACTTAACGTTCCTACTAAACCAAAAAAAAATCAAATTGACTAAGTCATAGAGAATTTGACGATGTTGGTAGGTAATTTTTTAACATGGTATATATTTTTTTAGGGTCGGTTATATTAGGTAATAAAAGAGGACGTGCCGTTAAAGTATTTTGCTTAAGATCCTTTAGATCCTTACAAAAATATAGATTGTTCGTCTGATGATGCCACTGTGTTGCAGTGATGGCTTGATCAATATCACCTCCCAAGACAAAACAGTAGTATATCGTTAAAAAGTTGTAATCTTTCATACAAGCCAACTGCATCATTTTATCGATGTCCATATGAACGATCTTTTGCTCGTATATTTCATGAAGGTCAAATACATTGTTGAAGTAAATGGCGCACATGAGTCGCCACATACTAAGGTGCCCATATGTTTGATAGAAAAAGGAGATAGCTCTTTTAAGCTTATATTTTACTGCTATGGCATAGCAGTATTTAACGAATACGTTCATGGGTACATTATCTAAGATATAAAATATGAAAAACTTTAACTCTCGATGAATCTCTTCCCCCATTTCCTGTACATTTAGAGCTTCCAACATAGGATTTTTATCAAATATTTCATGACATAAAATAATGTTATTGCTCGTTTTATGACGCATTAAACCGGTGAAAATTTCCTTATTATTTAAACTATCTTTAGCTCCTAACTTTCGACACAGCTCCTGAGTTTGTTCCGTCCTAGCACAGGTCAGCCCATAATAAATGTTTGCTCCCCACTCGGTGAACAGCCTTATTACGTCATAGTTATTTTCTTTTATGGCCATGATTAATGCCACATCAAGATGAAGAAGTTCCCCCTTAAAGGGGGTTGAGCTTAAAATAACGTAATTACAGTAGTGACATAAGCTAATGGGCTTGTTTTGCCACCATAAGCCACAATATTTTAAAATATAATGATACTCCTCAGGCACGCTCTGTTTGGCCACAGCCTTTTTGGCCAGGGTTTGCAAGGAGAGCATGATAACTTCTTGAAAAAAAAACTCAAATTAAGTTCCTACTTTTTTAAAATATTAGTATGGACAGATCTACCATCATATGAAGGAATTCTTTCATCGTTAAACACTGAAGAGATAATACTTTCATCGTATAGAGAATATCATGTCAATCCATATATTGAATGTTATATATCATTAAACCCATCATTAATATAGTGTTTATGTGCTATGGACAGGTTTTTTGAATGATAATCTTTTAACATACGTTTTATAACTTCGGGATCAGTTTCTTTTAAAGATAAAGAATCATTCATGTTATAACAATTTAATGATAACATGCTGGCAATGAACGAGTTGTCTTTTTGATGCGCTAGAGTCTTTCCCTCCTCAAAGGCATTGGCGCCTAAGTCTATACAAAAGAATATGTTTCCGATATTATAGAACTGAATAGAATGAAACATGGCCTGATTGATATCAGCCCCTAAGACGACGCAACAGTAATAAATCGTTAAATAGTTATAGTTCTTGCGACAGGCCCACTTTAGCATTTCATTCATGTCTATGCGAATCCTCTCCTTTTCGTACACTTCGTGAAGTTCAAACACATTATTGTAAAAAAGGGCGCACATAAGCCGCCACCGATGTAGATGAGCATATCTCTGATAAAAATAGCAAATCGCCTCCTTAAGGTTACATTCTATTGCCATCGCGTACCAATATTTAGTAAACATCTCGCTTAATATATCGGTTTCTACCATTAATCCCTCCAGTTGTTCATAAATCATTCCCTTTACTTCAAAACGATTTATGGTATCTAAAATGGGATTATTAGAAAATACCTCATGGCAGAAAATGATGTTACTGCTAGTTAGATCACGTTTCAATGTGTAAAAAAATCGTAAAATTTCCTGGTCATTTAACTGTTCTTTGGCACCTAGCTGCCTGCACAGGTCTCGGGTGTGCTCCGTGTTGACAGAAAGCAAACCGTAGTTGATGTTTGCACCCCACTCGGTGAACAATTCTATTAGATCGTGATTGTTTTCCTCCACAGCTTTCACCAAGGCCGCGTTAAGATTTGTGCCGTTCTTAAAATACGGCGTCCATATTTTCTTTTGATGATACATGATAGGGCCATTATGCCACCATAGACCGCAGCACTTCAAAAAATGAGGATGGCATTTGGCCGGATACTGGCTGGCCAGCACCTTTTTGGTGAGAGTCTGCAGAGAGAGGACCATATTTCTTTTTTTTGAAAAAATCAAATTAAAAAAATCATGCTTGTTTAGCATACATGTAATATTGTTATAATTACGTTATAATTACGTTATAATTACGTTATAACTATATTATAACAATGGTATAACAATGGTATAACAATGTTATAACAATGTTATAACGATGTATCATTGATGTCATCATTCAACTAGGCCAACATACTTTTTAATTTATAGTTTTTTAATAGATGATATATTTTGCTAGGATCTGCTTCTTTTAACGTTAATAGCGAGGAGTCTGCACTATAAATGTCTAATGATAAATGATGAGATATCAAATAGTAATTCCGTTGCTCTGCTAGGGCCTTTGCCTCTTCAAAGGCGTCGGCTCCCAGATCTATACAAAAGAACAAGTTATCCATATTATAAAATCGTACGCAGGCAAGCATAGCTGAATTAATATTAGCTCCTAAGAGAAAACAATAATATATGGTTAAAAAATTGTTATCTTTTGTGCAGGCCATCCGCATCATTTCATCCACGTCCATGCGGATCTTTTCCTTTTCATACAAATTATGTAGGTCAAACAGCTTATTAAAACAAAGAGCACAGATTAACCACCACGTATTTAGATACTTAAAATGTTGGTAAACATAAGAAATGGCCTCCCTAAGATTATCCTGCAATGCCACTATAAAACAGTATATCGTTAACATATCACCATCCGACATATTACTTAATATGTCGGTGTCTTCTACTAACCTTTTCAACTTCCAATATATGGATGACCTTATTTCCCTTATAATGACATAGGCTGGAAAGGGATTATCATTAAAAAGTTTAAGACATAAGATAATATTACTGCTAGTAGTGCCAGGGTGTATTAATTTAAAGAACATGTGCATAATCTTCTTTTTATCCACGCGGTACTTGGCTCCTAATTCCCAGCAAAATTCTCGAACAGGCGGCGTATTGGCGCAAATTAACCCATAGTTGATGTCTGCGCCCCATTCTGTAAACAGTTTTATTAACTGATAGTTGTTTTCCTTTGTAGCCAACATTAGTGCCGTATTAAGGTCCAAGCCGTCTGCAAAGCTTGGCAGCTTTATCAGCATATGTTTGCAATCAAGGGAAATTGGGGCCTTATACCACCATAGTCCGCAGCGTTCTAAGATAACATGGTACTCAATAGATACTTGCTGTCTGGCTAGTACCTTTTTGGCGAAGGATTGTAAGGAAGGAAACATCCTGTTTCTTTTTTTTTTAAAAATCAATTATCTTTGTTCATAATCAAGAAAAATCCCCATATTTATTGAGTGATAATTTTTTAACATGCAATTTATTTTTTCAGGGTCCGTAACGATCGACAACAGAGAAATAACCGGATTGTAATGCTTTAATGATAAGGCATGGGCTATCAGATAATTTTCCTTTTGTTCTGCCAAAGCTTTGCCCTCCTCAAAGGCATCGGCACCCAGGTCTATACAAAAGAACAGGTTTCCAAGATTATAGTTTTGTATGGAAACAAGCATGGCTTGATTGATGTTGGCTCCCATGATAAAACAGTAGTAAATGGCCGAATAGCTATAATCTTGGATGCAGGCTATGTGCATCATTTCATCAATATCCATGCGGACCCTTTCTATTTCGTACAGCTCGTGAAGGTCGAACACGTTGTTGTAAAAAAGGGCGCACATGAGCCGCCACCTATGTAGACGCGGGTATTTCTGGTAAAAGTAGCGGATAGCATCTTTGAGGTCATAGTCCACCGCTATCGCGTACCAGTATTTGGTTAAAACAGTGCTAAAGCTATCATCATGGTCCAGCATGAAGGTTATCTCCATGAGCCCTCTTAACTCCCACATGATTTCCCCCCTCAGATCCAGATTATCTATAATCCTTAAATTGGGGTTATTGGAAAACACCTCGTGGCAAAAGATAATATTGCTACTGGTTTTATCGCGCGTTGTATCAAAGAAAATTTTTAAAATATACTCTCTTTCTAAATATTCTTTGGCTCCCAGCTCTTTGCACAGATCACGGGTATTTTCCGTGAGAGCACAAATCATTCCATAGTTAATATCTGCACCCCATTCAGTAAACAGCTTTATCAAGTCATGATTATTCTCCTTCACGGCTTTCATCAGTCCTATGTTTAACTCGATACCTTGACTAAAACAGGTTGACCTTATAAATAATTTATTGCGTCGAATATGAAGCATAATGGGGCCATTATGCCACCACAGGCCACAACACTTCAGGACATGATATTGATCTACCGGTATACACTGCCCGGCCAGTACTTTCTTCGTGAGGGATTGCAGGGAAGGCAACATGCCTTTCCATCCTTTGACGGAAATCAAATTATCTACTAATAACTATCAGTGTTTATATTAAGTATTTAGATATTATCCCGGGCTGGATACGTAGTATCGCTATTCACATGTACTTCCAACTCTAGCCGGAGCCTGCAGGGTCATTTATTTTTAATATTGATTCTTTTTTGTATTTAATCATTTAGAGAAGGTCATCATAGGAGCCAGATGTTCTCTCTCCAGAACTTATGTCGAAAAACATTACCTAACCGTAAACTTCCTGAATTTTTTGACGAATATATATTACAACTGCTGGGATTATACTGGGAAAACCATGGAACTATTCAACGAGCAGGAAACAACTGTGTGCTTATACAGCAACATACCCTCATTCCCGTAAATGAAGCCCTGAGAACAGCAGCATCTGAAGAAAATTATGAGATCGTGAGCCTTTTATTAGCGTGGGAGGGGAACCTTTACTATGCTATTATAGGGGCTCTAGAGGGCAACCGCCACGACTTAATTCGTAAATATGATGACCAAATCAAGGACCATCATGAAATTCTGCCATTCATTGACGATCCAGTCATATTTCACAAATGCCATATCATGCGGCAATGCTTTTTTGATTGTATTTTATATCAAGCTGTAAAATATAGTAAGTTTCGCGTTCTTCTTTACTTTAAACATAGATTAGAGGATGATTTGCCCTTCACTCATTTACTTATTGAAAAGGCATGTAAAGATCATAATTATGAAGTTATTAAATGGATATATGAAAACCTACATATCTACAATATGATAGATACCTTTGAATGTGCTATTGCCCATAAGGATCTACATCTATATTGTTTGGGGTATAGATTTATATATAACAGAATCGTACCCGATAAGTATCATCATTTAGATATTCGCATGCTTTCAAGCCTACAACTCCTACATAAGGTGGCAGCCAAAGGATACTTAGATTTTATCCTAGAAACCTTAAAGTATGATCATAATAAAGATAATATAAATATTATTCTAACACAAGCTGCAACCTATAACCATAGAAAAATTTTAATCTATTTCATTCCTCAATCAACCCACGCACAGATAGAACAATGTTTACTAGTGGCGATAAAAGCAAAATCTTCCAGGAAAACCTTGAACTTACTACTGTCTCACCTAAACCTTTCCATCAACCTCATCAAAAAAATAAGCCATTATGTTGCCACTTACAATTCAACAAATATAATAGGCATTCTGAGTATGCGGCGGAAAAAGAAGATATATTTAGATATCATATTGACAAAATTTGTAAAAAAAGCTATTTTTAATAAGTTTGTCGTTCGATGTATGGATACATTTTCTATAAACCCGGAAAGAATCCTTAAAATAGCCGCGCGAATAAATAGGATGATGTTAGTGAAAAAAATATCTGAACATGTTTGGAAAAATCATGCGGTTAGACTTAAATACCTTAAACATGCGGTACACACGATGAAGCATAAAGATGGGAAAAATAGACTCATGAACTTTATCTATGATCGCTGTTATTACCATATGCAAGGGGAAGAAATCTTTAGCCTCGCAAGATTTTATGCAATCCATCATGCACCAAAGTTGTTTGACGTTTTTTATGATTGTTGTATCCTAGATACGATACGATTCAAAAGCCTTCTTTTAGATTGTTCACATATCATAGGTAAAAACGCTCATGATGCTACCAATATCAACATCGTGAACAAGTATATCGGCAACCTGTTTGTTATGGGAGTTCTTAGCAAAAAAGAAATCTTACAGGACTATCCATCCATTTATTCTAAACAATACATGCCTTAGTTTATTTTTTTTGCGGCCGAAACATTATTCTTACCCTAGAAAACGCTTATAGTCATCTTAAATCATAGGTAAGGAAGATCATCATATTTTTTGAAACGTAATTTTTTAACGCATGATCTATGATTTCAGGGTCCGTGCTTTTAGGCAACGGGGTGGTGGCCGGACTATAAATCTTTAGGGATAAAATGTTCTTTATAAGCTCATACCCTTCCCCTAAAGCTGTAGTACCCTCTTCGAAAACATCAGCCCCCAGATCTATACAAAAGAACATGTTTTCTATATTATAGTACTGTATTGAGCTAAGCATGGCTTGATTGATGTTGGCGCCCAGGACATAGCAGTAGTACATGGTTGAAAGGTTGTGGTCTTTGATGCAGGCGATCCGCATCATCTCTTCTATGTCCATATGGATCTTGTCCTTTTCATACGCCTCATGAAGGTCAAACACATTATTAAAACAAAGAGCACATGTTAACCGCCACGTATTCAGGTGTGTATATTTTTGGTAAAAATACTGTATGGCCTCTTTCAGGTTATAGCGTATGGCTATAGCGTACCAGTATTTGAGTAGTAATGTACTGAGCGAAAACTCATTATTTAGCAGATCGGTTTTTACTATTAACTCCCTTAACTCCCAGAAAATTTCTATCCTCATTTTTATATTATTTACTTTTTGTAATATCGGATTGTTGGAAAACACCTCATGGCATAAAATAATGTTACTACTAGTTTTATGAAACTTTAGATCTATAAAAATTTGTAAAATTTCTTCTTCATTCAAGGTTTCCTTGGCACCTAGCTCTCGACAGAGGTCCCAGGTGTGCTCCGTGTTGACAGATACCAGCCCGTAGTTGATGTCCGCCCCCCACTCTGCAAACAGTTTTATAAGGTTGTAGTTGTTTTCCCTTACAGCCTTCACTAACGCCGTATTTAGGTTTAAGCCCTCTTTAATACCTGCTGATTTTATGAGCCTTAGGTTATGATCAAACGTGATCGGAGCATCATGCCACCATAGGTCATAACACTTTAAAAGATAATGTTGGTTCGTGGGCACGCATTGTCCAGCCAACACCTTTTTGGTCAGAGATTGCAGGGAAGGCAACATGTCTCTTCATCTTTTAAAAAAAAATCAAATTAATTAGCCGAATAAATTTTTCTTTCGAGGGCTTTTTAAAAGAGCTCTTTAAGAGCTCTTTAAGAGCTTTTTAAGAGATTAAAAAATTATTCTTGCTGGCATTCTGCCAAGTATGCGGCATTCCTATCATCTATAGTATATTATGAGAATATTCCCAAATGATGGATAAGTTTTTTGATTTATAATCTTTTAATAAACTGCTTATTTCTTCGGGGTCCTTTAAGTTTAGTGGCAAGGAAGCATCTGAGCTGTAAATATCCAAAGCCAAACTATGGCTCAGAAAATTATAACCTTTTTGTTCCGCTATGGCACGACCCTCTTCAAAGGCATTACCACCCAAATCTATACAGAAAAATATATTACCGATGTTATAATATTGTACTGAAGTAAGCATAGCTTGGTTGATGTTGCCCCCCAGCGCGTAACAGTAATATATTGTTAATGGATTGTTATCCTTGGTAGAAGCCAGACATATCATGTCATGGACGTCTATTTGGATGTTTTCCTTGTGGTACATCTCATGAAGCTCATATATTTTGTTATAATACAGGAGACATTTTAATCGCCATTCATTAAGATCCGTATATTTCTCATCTAGAAAACAAATGGCGTCCTTACAATCGTATTGTACTGCTTTGGCGTACCAATACTTCACTAGTAAACCATTTAACTCGTCCGTTTCTTTTATTTCTATGAGCCCCCATAGTCTTTTATAAATTAAGCCCCTTAATTGTATAACAAATTTGTTTTCTAAAATAGGATTATTCATAAAAATTTCATGGCACAAAATAATACTGCCGCTGGTTTTATTGTGCATTATCCTGGTAAAAATACGGAAAATATCGTTGTCCTCTAGAGTTTCTTTGGCGCCTAGCTGTCTACACAACTCTCGGATGTGCTTCGTATTGATAGAAAGCAAACCATAGTTGATATTTGCGCCCCACTCTGTAAAGAGCTTTATCAGACTATAGTTGTTTTCCTTAACAGCTATTATTAATGCCACACGAAGGTCTATATCTTCTCCTAAAAATCCTGATTTTATTTGTATTCGGCCACGATCCATACAAAGCTTGAGAGGAGCATCATGCCACCATAGGCCACAATATTTCAAAATGCAGTGTTCATCTATTGACAAACACTGGCTGGCTATCGTCTTTTTGACGAGGGTCTGCAGAGAGAGCGGCAACGACATGTTTCTTTTTCACCAAAAAAAAATCAAATGTTCTCGTCTTTAAAGGTTAATTCATGTTCTTAAAATGTTCATTTCATGATAGTGATTAATAATATGGTTTAATAACGCTAGAAGGCTTGTTTATAAGACAGTCATAAGCAGTCTATAAGACAGTCTATAAGCAGTCTATAAGACAGTCTATGACTTAGTCTATAACTATAATTTCTGGATGGGCTGTAAGATACTCTTCGGCTCGTTTCAGATTTTTTGAAGTATATGTCTTTAGCATATCATATATTTCCTGGGGTTCGGTTACATCTAATACCAAGGTCACATCACGGCTGAAAAGCTGCTTTACTAAGAAAATGTTGCTCAAGTTATACATATAAGCTTTGTGCGCAATGAGTTGTGCCCTATCAAAATCGGCAGCCCCCAAATCAATACAGAAAAACATGTTTAAAGTATTATTGTTATAGATAGAAAGATTCATGCCATAATCGAGACTAGCCCCCAACCTATGACAGTAATAAATGGCCGCGTAATTTTTTTCCCGCAAGCAAGCAAATTTCATCATCAGATTAGGGCTGATGCAAATCTCTTTTTCACGACACAACTCGTGTATGTCAAAAATGTTATTAAAATAAAGGCTACAAGCTACCCGCCAATAGAGGTGATTTTTATGCCTTTTATAGAAATAGTGAATAGCCTTTGTAAAATTATGTCGTAATGCCAGGGCAAACCAAAACTTTGTTAATAGGTGGTGCGCCGTATCCCCCGTCAACGGAATGTTTGAACAGGTGTACGTAACTGTGTCTAAAGTGGTTCTAGTTACGGTTTCCAAGAGTGGATTATGACAAAACATGTCATAACCCAGCAGAACTCCTGCACAGGATTTTAGCCTGGCCACTTCTTTTAAAATTTCCAGAAGACGGGGTTCGGATACAGGCGTTAAGCCTCCCAGTTCCGCACACAGCCGCTTTAGATACACGGCAGGAACACGTATAAGCCCATATTCAGGATTTGCGCCCCAATCCACAAATAAACGTATAAGTTCAAGATTATCGCTCTTCACGGCCTTTACTAGCGCCGCTTCGAGACAAAGATCATCCTCAGAAAAACACTGTAAATGTTTATACGAAAAAACTTGCTTACAATTGTTACATAGGTGAATAGGACCTAAATCCCACCACAAACCAAAACGCTGCAACGTATAATCATAGTCACTTGAAAGATAATTGCATGCCACAACTTTTTTGGCCAACGTTTGTAAAGACAACATACTAAGTTTAAAACATCTTAAATCTAAGCTAGCTAACTTTCAAGAAAACCCTCTATCCCTAAGAATATATCTTATAACTAGACTTATAGCAGTAAAAATCAACTTTGGTTATTCTTTTTAATATAAAACGTCTAATTACTTGCAAAGGACTATAAAGCCCATTTTCCTCAGCTAGAATTTTTATTTTTTAATGAAGTAGGGGGATATGTTTTCCCTTCAAGACCTTTGCCGAAAGCATCTTTTTATTCTTCCCGATGTTTTTGGCGAGCATGTACTACAACGATTAGGACTGTATTGGAGATGTCACGGCTCCCTTCAACGCATAGGAGACGACCACATACTCATACGACGGGATCTCATCCTTTCCACCAACGAGGCCTTAAGAATGGCGGGAGAGGAAGGAAACAATGAAGTAGTAAAGCTCTTGTTACTGTGGAAGGGAAATCTTCATTACGCCGTCATAGGAGCCTTGCAGGGTGATCAATATGACCTGATCCATAAGTATGAAAACCAAATCGGCGACTTTCATTTTATCTTACCATTGATTCAAGACGCGAATACGTTTGAAAAATGCCACGCTTTAGAACGTTTTTGTGGTGTTTCATGTCTGCTAAAACATGCTACAAAATACAACATGCTCCCTATTCTCCAAAAATACCAAGAAGAGCTGTCTATGAGAGCGTATCTTCACGAAACCCTATTTGAACTAGCATGCCTATGGCAGAGGTATGATGTCCTTAAATGGATAGAGCAAACCATACATGTTTACGACCTAAAGATTATGTTTAATATTGCCATCTCCAAGAGGGATCTGACTATGTACTCCTTAGGATATATTTTCCTTTTTGATAGAGGGAACACCGAAGCTACGTTGCTAACGCAACATCTCAAGAAGACAGCGGCCAAAGGGCTCCTCCACTTTGTGCTAGAAACGTTAAAATACGGCGGCAACATAGATACCGTCCTGACCCAAGCCGTAAAGTACAATCATAGAAAACTTTTAGATTATTTTCTGCGTCAACTACCTCGTAAACATATTGAAAAACTTTTGTTGCTGGCCGTGCAGGAAAAGGCTTCTAAAAAAACATTGAACTTACTGTTGTCACATTTAAACTACTCCGTGAAACGCATCAAAAAACTACCGCGCTATGTGATAGAGTACGAGTCCACCTTGGTGATAAAGATTTTATTAAAAAAAAGAGTGAACCTGATAGATGCCATGTTGGAAAAGATGGTAAGATATTTTTCTGCGACGAAAGTGAGGACGATCATGGATGAGCTTTCGATTAGTCCGGAAAGAGTCATTAAGATGGCTATACAGAAAATGAGAACGGATATCGTAATCCATACTTCTTATGTTTGGGAGGATGATCTAGAACGTCTTACTCGTCTTAAAAATATGGTATACACCATAAAGTACGAACATGGGAAAAAAATGTTAATTAAAGTCATGCACGGCATATACAAAAACTTATTATACGGCGAAAGGGAAAAAGTCATGTTTTATTTAGCCAAGCTCTATGTTGCTCAAAACGCGGCCACCCAATTCAGAGACATTTGTAAGGACTGTTACAAACTGGATGTGGCACGGTTTAAACCGCGGTTTAAGCAACTAATATTAGACTGTTTAGAAATTATTACTAAAAAATCTTGCTATAGTATCCTGGAAATCTTAGAAAAACATATTATTTCCCTGTTTACTATGAAAGTTATGACTGAAGAAGAAAAAAACCTATGTTTAGAAATATTATATAAAGTAATTCATTATAAAACAATACAATGTTAAAATTCAATAGATATCCATCATTAATATTGATTATATTTTCGAATATTATCTTCTATGGTGCAAGATAATCATCTAGCGCGTGAAACATGTCCTCTTCTCTTCAGGAACTTTGTCGAAAAAAGCTGCCTGACTGCATACTTCCAGAGTTTTTTGACGACTATGTATTGCAACTGTTAGGACTGCACTGGCAAGATCATGGTTCCCTTCAGCGTATCGAGAAGAACCAGATACTTGTTCAACAGGAACCCATCCATATCAATGAAGCACTCAAAGTAGCAGCATCGGAAGGGAACTATGAAATCGTAGAGCTGTTGTTGTCATGGGAGGCAGATCCCCGCTACGCCGTCGTAGGAGCCCTAGAAAGCAAATACTATGACCTGGTTTACAAATACTATGACCAAGTTAAAGACTGCCATGATATCTTGCCGCTGATTCAAAATCCGGAAACATTCGAAAGATGTCATGAGTTAAACAGCACCTGTTCACTGAAATGCTTATTCAAGCATGCTGTGATAAATGACATGCTGCCGATTCTTCAAAAATATACAGACTATCTGGATAGGTGGGAGTATTGCAGCCAGATGCTGTTCGAACTGGCATGTAGTAAAAAAAAATATGAGATGGTTGTGTGGATAGAGGGAGTTCTAGGCGTCGGCAAAGTTACATCTCTTTTCACCATTGCGATTAGCAACAGAGACCTACAGCTGTATTCTCTGGGCTACTCAATTATCCTTGAGAATTTGTACTCCTGTGGACAGGACCCCAAGTTTTTACTAAATCATTTCCTGCGAGACGTTTCAATAAAAGGGCTTCTACCCTTTGTAATCAAAACCATAGAATATGGTGGAAGCAAGGAGATAGCCATAACTCTGGCTAAAAAATATCAGCATAAACATATTTTGAAATACTTCGAAACCTGGGAAAGCTAGGTTCAGTATGGTGTACTCACTATTGTAGTGAATCGTATCCTGTAAATTTTGTAAAAAAGCTTAAACTTTTGACCACATCATATTGTTTTAGAAATCTCAAACCAGTGAACAACAGTCTTATCATACATTAAAATTCCAGTAAAATTTATATTTTTTTTGGTAAACAAATGTTTTCTCTTCAAGACATCTGTCGGAAACATCTTTTTCAACTTCCTGACGCTTTTGATGAATATATATTACAAGCGCTAGGACTATACTGGGAAAAACACGGATCTCTTCAACGAATAAGAAAGGACGCTGTGTTTGTACAGCGAAACATCGTCCTTTCTACCAATGAGGCCCTGAGAATCGCAGCCTCAGAGGGAAACGAAAGGGTAATAAAACTTCTGTTATCATGGGAGGGAAATTTTCATTATGTGATCATAGGAGCTCTAGAGGGTGACCAATATGACCTAATTCATAAGTATGATAGTCAAATTAAAGACTACCACATGATTTTATCATTGATCCAAAATGCAAATACCTTTGAAAAGTGTCATCAGTTATCCAATAGTAATATGTGGTGTCTTATACAGAATGCTATAAAATATAATATGCTCCCTATTCTCCAAAAACACAGAAATATTCTGACACATGAGGGAGAGAATCAGGAATTGTTTGAGATGGCATGTGAGGAACAGAAATATGACATAGTTTTATGGATAGGACAAACCCTAATGTTAAATGAGCCGGAGTTTATTTTTGATATCGCCTTCGAACGGATAGATTTTTCTTTATTAACAATGGGTTATAGCCTTCTTTTTGATAACAAGATGAGTAGTATAGACATTCATGATGAAGAAGATCTTACTTCATTACCAACAGAACACCTCGAAAAAGCAGCCACTAAGGGATGTTTCTTCTTTATGCTAGAAACTTTAAAACATGGTGGAAATGTAAATATGGCAGTCTTATCTAAAGCTGTTGAGTATAATCATAGAAAAATTTTAGACCATTTTATTCGGCGGCAAAAATGTTTATCACGTGAAGAGATTGAAAACCTATTATTAACCGCCATAACCAATTGTGCATCCATAAAAACGTTAAACTTACTCTTGTCTTACCTAAACTATTCCGTAAAAAATATCATTGGAAAAATAGTACAACATGTCATAAAAGATGGTGATTATACCATCATATTACTTTTAAAAAAAAAGAAAATAAACCTAGTGGAACCTGTTTTAACAGGTTTTATAGATTATTACTATAGCTATTGTTTTATAAAACATTTTATCCAAGAGTTTGCTATTCGTCCGGAAAAACTGATTAAAATGGCCGCGCGAAAAGGTAAACTAAATATGATTATCGAATTCCTTAACGAAAAATATGTTCATAAAGATGATCTTGGAACTATATTTAAATATCTCAAAACCCTAGTATGTACCATGAAACATAAAAAAGGAAAAGAGACATTAATTGTTCTTATTCATAAAATATATCAAGATATTCATCTGGAGACTAAAGAAAAATTTAAATTATTAAGATTTTATGTCATGCATGATGCAACTATCCAATTTCTATCTATGTGCAAAGACTGTTTTAATTTAGCCGGTTTTAAACCATTTGTTTTAGAATGTTTGGATATTGCTATTAAAAAAAATTACCCTGATATGATACAATATATAGAAATTCTATCGAAATCTGAGTAAAATTTATTTTTTTGATCAGAGTAAGAAAATGTTCTCCCTCCAGGAGATCTGTCGAAAGAACATCTACTTTCTACCTGACTGGCTCGGTGAGCATGTGATTCAGCGACTAGGTCTGTACTGGGAAAAACATGGTTCTCTTCAGCGAATCGGAGACAACTATGTACTTATACAACAGGACCTCATCATCCCCATCAATGAAGCCCTAAGAATGGCAGGGGAGGAGGGGAATGATGAGGTGGTACAACTCCTATTACTATGGGAGGGAAACATTCATTATGCCATCATAGGAGCTTTGGAGAGTGACCATTATAGCCTAATACGTAAGCTCTATGACCAAATCGAAGACTGTCACGACATCCTTCCCTTGATTCAAGACCCAAAACTCTTTGAAAAATGCCATGAATTAGATAAATCTTGTAACATTTTATGTCTCGTATTACACGCCGTAAAAAACGATATGCTTTGCATTCTTCAAGAGTATAAAATGCATCTAAGTGGAGAGGATATTCAAGTGGTGTTTGAAACAGCATGCCGTTCACAAAAAAACGATATTGTGTCATGGATGGGACAAAATATTGCAATATACAACCCCGAAGTTATTTTTGATATTGCCTTTGATAAGATGAATGTGTCCTTATTATCTATAGGGTATACGCTTCTTTTCAATCATCATATAAATAATACGAACGAAAATATTAATTCTTTATTGACACAACATCTTGAATGGGCTGCCGGCATGGGCCTTCTTCATTTTATGCTGGAAACTTTAAAGTATGGCGGGGATGTAACGATAATAGTCTTGTCTGAGGCCGTAAAATATGACCACAGAAAGATTTTAGATTATTTTCTCCGTCGAAAAAACTTGTACCAAGAAGATCTTGAAGAACTATTATTGTTGGCGATACGTGCAGATTGTTCTAAAAAGACCTTAAACTTGTTATTATCTTACTTAAACTATTCCATAAACAATATCCGTAAAAAAATATTACAATGTGTAAAAGAATATGAAACGACCGTTATTATAAAAATTTTACGGAAAAGAAAGATAAATCTGATAGAGCCCATTTTGGCAGACTTTATAGGATATCATAGCTATACCTATATGGTAGATTTTATGCGTGAGTTTTCCATCCATCCGGAAAAAATGATCAAAATGGCTGCACGAGAATCGAGGGAGGACTTGATCATAAAATTTTCCAAAAAAGTTTGCAAAGAGCCTAAAGATAGACTTCACTATCTCAAAAGCTTAGTGTATACTATGCGACATAAAGAAGGCAAACAACTGTTAATTTATACAATCCATAACTTATACAAAGCTTGTCATCTAGAGAGTAAAGAAATGTTTAATTTGGCACGATTTTATGCACGGCATAATGCAGTGATCCAGTTCAAATCGATTTGCCACGATCTCTCCAAGCTCAATATTAATATCAAAAACTTGTTGTTAGAATGTTTAGGTATTGCTATTAAAAAAAATTACTTTCAACTTATCAAAACAATAGAAACGGATATGCGTTATGAGTAACATTTTTAGATGAGGGAAGATTCTACCAAACTAACTAAGACCTTTCGCTAGAATGTATCTTATTGTTAATATAGATGAGATATGTCATTGTGAAAAAATAGATTAGGTAGGTTGTGAAAAACAGATTAAACTTAAAATTATGTGTATTATGTAAAATTTTAGAAATAAAAATTTATTTTTTTTTATTGAGGGTACGGAAAATGTTCTCCCTACAGGACCTCTGTCGGAAGAACATTTTCTTCCTTCCAAATGATTTTAGCAAGCATACCCTACAATGGCTGGGATTATATTGGAAAGAGCATGGATCCGTCCATCGAGCAGAAAAAGACAGCATAATGATACAGAATGAATTGGTTCTTTCTATCAATGATGCTTTACAGCTTGCAGGAGAGGAGGGGGACACAGATGTAGTACAGCTCTTGTTATTATGGGAGGGAAATCTGCATTATGCCATCATAGGAGCCTTGAAGACTGAAAAATATAACCTAATATGTGAGTATCATAGCCAAATTCAGGACTGGCATATTCTCCTACCCATGATTCAAGATCCAGAAACATTCGAAAAATGTCATGATTTAAGCCTTGGATGTGACTTTATTTGCCTTCTCCAACATGCTGTAAAATACAACATGCTTTCTATTCTTGTCAAATATAAGGAGGATCTACTAAATGCAAGGATTAGGCATCGTATCCAATCCCTGTTTGTTTTGGCATGCGAAAATCGGAGAATTGAAATTATTGATTGGATAGGCCAAAATCTGCCAATTCCTGAACCTGATGCCATTTTTAGCATTGCTGTTGCTACAAGAGATTTAGAACTGTTTTCCTTAGGGTACAAGATTATTTTTGATTACATGCAAAGACAGGGAATCATTCAATTAACCAATGGAGTTCGCATGGTTGTGCTAAATCGTCACATTAGCATGGCAATAGATAATGGTCTTTTACCTTTTGTTCTGGAAACTTTAAAACATGGTGGGAATATACATAGAGCCTTATCTTATGCAGTAACACACAATAGAAGAAAAATTCTGGATTATCTTATTCGCCAGAAAAATATAGCCCCTAATACAATTGAAAGACTTTTATATCTGGCCGTGAAAAATCAATCTTCCAGGAAAACTTTGAACTTGTTGCTATCTTACATAAATTACAAGGTGAAAAATGTTAAAAAGCTGGTAGAGCATGTAGTAAATGAGAAATCCACTCTTGTGTTAAAAATTTTATTAGAAAAAAAGGAAAATCTAGTGGATGCTGTTTTAACAAGACTTGTAAAACATTCTACATATTTCCAGGTGAGAGAATTTATCCAGGAGTTTTCCATCAGCCCAGAAAAATTCATTAAAATAGCTGTGCGGGAAAAGAAAAATGTGTTAATCGAGGCTATTTCTGAAGATATTTGGGAAAATCCCACAGAAAGAATTACTTATCTCAAACAGATAGTGCACACCATAAAATATGAAAGTGGAAGGCGATTTTTGGTAGACATCATTCACAGCATTTACCAAAGTTACTCACTAAAACACGAAGATATTCTTAAACTGGCAACATTTTATGTCAAACACAATGCAATCACCCATTTTAAAGACCTCTGCAAATATCTTTGGCTGAACAGAGGAACAGAAAGTAAGAAACTGTTTTTAGAGTGTTTAGAAATTGCTGATGAGAAGGAGTTTCCTGATATTAAAAGTATTGTGAGTGAATATATTAACTACTTGTTTACTGCAGGAGCTATTACCAAGGAAGAAATCATGCAAGCCTATGATGCTTTAGAGTAGCCATGTATTAACATTCTGAAAGTAGAATAAAATATACTATATACTAAAAACCAAATTAGCCATTTTTAACTATCTTCTTCTTAAAAACTCTGGATAAAAATTTATTTTTTTTTAATTTGGGTAGGGAAAATGTTCTCCCTTCAGGACCTCTGTCGGAAGAACACCTTCTTCCTTCCAAGTGATTTTAGCAAGCATACCCTGCATTTGCTGGGGTTATACTGGAAGGGGCATGGATCTATCCAAAGGATAAAGAATGATGGTGTGCTTATAGAGCATGATCTTACTCTTTCCATCAATGAAGCCTTAATTCTTGCAGGAGAAGAGGGAAACAATGAAGTAGTAAAGCTCTTGTTACTATGGGAAGGAAATCTTCATTATGCCATCATAGGAGCTTTGAGGACTGAGAACTATAACCTAGTATGTGAGTACCATAGTCAAATTCAGGACTGGCATGTTCTCCTCCCTTTGATTCAAGATCCAGAAACATTCGAAAAATGTCATGATTTAAGCCTTGAATGTGATCTTTCATGCCTTCTCCAACATGCTGTAAAATATAACATGCTTTCGATTCTTGTTAAATATAAAGAGGATCTACTAAATGTACTATTTAGGCAACAAATTCAAGGACTATTTATTTTAGCATGTGAAAATCGGAAGCTTGAGATTCTTACGTGGATGGGTCAAAATCTGCCAATTCCTGATCCTGAGCCTATTTTTAGCATTGCTGTTGTCACAAAAGATTTAGAAATGTTTTCCTTAGGGTACAAGATTGTTTTTGAATACATGGAAAACCAAGGACTTCATTTAACCCAGGTAGTTCGTATGGTTATGCTAAATCATCACTTTGGCATGGTAATAAATAAAGGACTTTTACCCTTTGTGCTGGAAATTTTAAATTATGGTGGGAATGTAAATAGAGCCTTATCTTATGCTGTCACACAAAATAAAAGAAAGATTTTAGACCATGTTGTTCGCCAAAAGAATATACCCCATAAAACCATTGAAAGAATGTTGCATCTGGCTGTAAAAAAGCATGCTCCCAGGAAAACTCTGAACTTGTTACTATCTTACATAAATTACAAGGTGAAAAATGTTAAAAAGTTGTTAGAACATGTAGTGAAATACAACTCTACTCTTGTGATAAGACTCTTGTTAGAAAAAAAGAAAAACCTGCTGGATGCTACTTTGACAAGATATGTCAAAGATTCTACATACTTTCAGGTGAAAGAATTTATGCAAGACTTCTCCATCAGCCCAGAAAAATTCATTAAAATAGCTGTGCGGGAAAAGAGAAATGTGTTGATCAAGGGTATTTCTGAAGATATTTGGGAAAATCCCGCGGAAAGAATCAGGAATCTTAAGCAGATAGTGTGTACCATAAAATATGAAAGTGGAAGACAATTCCTGATAAATATCATTCACACCATTTACCAGAGTTATTCTTTGAAACCTGAAGAAATTCTTAAATTGGCAACATTTTATGTCAAACACAATGCAACCACCCATTTTAAAGATCTCTGCAAATATCTTTGGCTGAACAGAAGAACAGAAAGTAAGAAACTGTTTTTAGAGTGCTTGGAAATTGCTGATAAGAAGGAGTTTCCTGATATTAAAAGTATTGTGAGTGAATACATTAACTATTTGTTTACTGCAGGAGCTATTACCAAGGAAGAAATCATGCAAGCCTATGCTTTGGAGTATGCCATGTATTAAATTTCTGAATCAGTAAGCAATAGATAGATTTTAGAATATGCTGTATTAAGTTAGTTTCTGAATAAGTAATTAATAGATAGATTTTAGTTTATGTAAAAATGTTAACATTTGTTCATAAGTTTTAGATACCATTTTAGAGTTACTTTTTTAGATATTACTATTTTAGCCATTATTATCTTAAATAATCACTATTTTAGATAGGTCCCCGTATTAAAAACCAAATTAACCATTATCTATGTTTTTAATAATACTTTTTAAAAACCCTCCATAAAAATTTATTTTTTTTTCATAAAAGTAGAGAAAATGTTCTCCCTACAGGATCTCTGTCGGAAGAACCTTTTTCTTCCACTTGAGCCCTTAGGCAAGCATGTGGTTCAACGGCTGGGATTATACTGGGAAGGCCATGGTTCAGTTAAACGAGTGGGTGATTGCTTTATATGTGTAGACCAGATTTGGATGCTATCAATCCATAAGGCTATACAAATTGCAGCCTCGGAAGGAAATGAGAACATTGTCAAGCTTTTCTTACTATGGAAGGGGAGTCTACAATATGCCATCATAGGAGCCTTAGAGGGCAGGCAATATGATCTGATTCAAAAATATTACAACCAAATTGGGGACTGCCATCAGATTCTACCACTGATTCAAGATCCAGAAATTTACGAAAGATGTCATGAATTAAATGTTACATGTACCTTTCAATGCTTATTTCAACATGCTATAAGAGATAACATGCTGCCCATTTTCCAAAAATATGGAGAAGATCTGAATGGAAACAGGAGAATGGTTCAACTTCTGTATGAGATGGCATGCCGATTACAAAATTATGATATCATCAAATGGATAGGATCTAACCTGCATGTTTATAACTTGGAAGCCATTTTTAGCATTGCTTTTGTTAGAAAGGATTTAACTTTGTATTCTTTAGGCTACATGCTTCTTCTGGGTAGAATGAGTACTGAAGATAGAAACTTTATCTCAATCATAACACGCCATCTTGAATACGCATCAAAAAAGGGACTTTTTGACTTTGTACTAGAATCTTTGAAATATGGAGGTCAAGTGGATACAGTGTTGTTTCAGGCTGTAAAATACAACCATAGGAAAATTTTGGCCCATTTTATTCATGAAATTCCCCGTGAAACGGTTGAAAAGCTGATACTCCATGCTGTGGAGTCACGGGCCTCCAGAAAAACATTCAACCTGCTTTTATCTTCCATAAACTACTGTGTGAACCCTTTTGTCAAAAAACTACTGCACGCTGTGGTGGAACACAAGTACATGCTTATCATAAAGCTTTTGCTCGAGCGGCCCAAAAAGAAGATAAACCTGGTAGATGCTGCTCTATTCAAACTTGTAAAATACTCTACTTATACAGAAATAGTAAAATACATGGGTGAGTTTTCTGTGGACCCAAAAAGGGTGGTCAAAATGGCAGCACGACTCATGAGAGTGGACCTGATTAAAAAGATTTCTAATGATGCATGGGAAGATAAACTAGAGAGAATCAAGCACCTTAAACAGATGGTAAATACCATGAACCACAGAAATGGAAAAAATCTATTGATGTACAATATTCACAATATTACTGGATATACCTATCTGAACACCAAAGAAGCATTTAACTTAACAAGATTTTATGCTGTCCACAATGCAACATGTTTGTTTAAAGAAATGTGTAAAAGCTGTTTTGTACATGATAAAATACAGCTCAGAGAATTGCTTGAAGATTGTTTACATATTGCTAATAGGCATGATTATATCCAGATTGCAGAAACCGCAGATGAATGTATCAAATATATAGATCTTATTACATTTAAGTAAACCATGTATATATCAAGTAAATCCAGATTAAATCAGGCTAATTGTAAATAGTTGTAGATACCATATAATGAATGTTTTATTAGGATAGTAGTTCAGTTAAGATAGTAGTTTAGTTAAGATAGTAGTTTAGTTAAGATAGTAGTTATGTTAAGATAGTAGTTCTGTTAAGATAATAGTTTAGTTAAAACTAGTTCATGTTAAGTTAATAGTTTTGTTAAGACAATAGTTCATTTAAGTCAATAGTTCAGTTAAGTCAATAGTTTTGTTAAGTCAATAGTTTAGTTAAGTCAATAGTTTAGTTAAGTCAATAGTTTAGTTAAGTCAATAGTTATATTAAGACATTAGTTCTGCTAATACATTAGTTTTGTTAAGATAATAAAAATTTATTTTTTTTTCATCAGGGTAGAGAAAATGTTCTCCCTACAGGAGCTCTGCCGGAAGAACATTTACATTCTTCCTTACCCCTTGGCTAAGCATGTACTTCAACAACTAGGGCTGTACTGGAAGGGACATGGATCTCTTCAACGAATCGGAGATGACCATGTACTCTTACAGCAGGACCTGATCTTTTCCATCAACGAGGCCTTAAGAATGGCAGGAGAGGAAGGAAACAATGAAGTAGTAAAGCTCTTGTTACTATGGGAGGGAAACCTTCATTATGCCATCATAGGAGCTTTAGAGGGCGACCGATATGACCTTATCCATAAATATTATGATCAAATTGGGGACTGCCACAAGATTCTTCCTTTAATCCAAGACCCGCAAATCTTTGAAAAATGCCATGAATTGAGTAACTCCTGTAATATTCGATGCCTTTTAGAACATGCAGTAAAACACGACATGCTTTCTATTCTTCAAAAACACAAGGAGCAAATAAGATTACACATGGCATTAACCCAAATACTATTTGAATTGGCGTGTCATGAACGTAAAAATGACATCATTAGATGGATCGGTTATTCCCTGCACATACACCATCTAGAGACTATTTTTGATGTTGCATTCGCCCATAAAAATTTATCCTTATACGTTTTAGGGTATGAACTTCTCATGCACAAAGTAAATACAGAGGCTGCATATATAGAATTACCCAATTTGCTATCATATCACCTTCGAACTGCGGCGGCAGGAGGTCTTCTTAACTTTATGTTAGAAACAATAAAGCATGGTGGATATCTGGATAAAACGGTTTTATCCGCGGCTATCAGGTACAAGCATAGGAAAATTGTGGCTCATTTTATTCATCAGGTTCCCCGTAAAACCGTTAAAAAACTGTTACTCTATGCTGTGCAGGCTCGGGCCCCCAAAAAAACACTGAACCTACTTTTATCTTCCTTAAACTACTCCGTGCACACCATCACCAAACAACTCGTACACAATGTCGTCATCTACAGTTCCACGCTTATCGTAAAGCTTTTACTCATGCGGCGAAAAAACAAGTTAAACCTAGTAGATGCCGTTTTAGCCAGACTTGTAAAATATTCCACCTATACAGACATTGTACAATTCATGGGTGAGTTTTCTGTGAGCCCAGAAAGGGTGATCAAAATGGCTGCACGGGAATCCAGGACCTTTCTGATTGAAATGATCTCCAAAGCTGCTTGGGGAAATCACCCACAGACGTTGATTCATCATCTCAAACAACTAACCAATACCATGAAGCCTCAATCTGGAAAAGACCACATCATATATACCATCCACTATATTTATCTAAACTCTAATATGCTGGTAGCGGAGGAGGAAAAAAATATTTTTAAATTAGCAAAATTTTATGCGAATCATAATGCGGTAAACAGGTTTAAACAAATTTGTGAAGACTATTATATATTAGATGCACGATTTAAAACACTTATTTTAGAATGTTTTGAAATTGCCGTCCAGAAAAACTATCCTAGAATTGCAAATATTGTGGATGACTATATTCGATTCCTTTTTTACAGGGGAAATATAACCGAGGAAGAAATTCGTGAAGCCTATTCTTTAAAAGATGCTGAGGTTTATGTAGATTTAAAATGGTTACAACAAGGAGAAATGGTTTAAACCAAATCCGGTTTAAACTAAATCCAATTTAAACTACATTTGGTTTATCATTAGTCATTGAAACCATCGAAAAAAAAGCTATTTGTTTATCCCCATAAACTCATCTTTTTTTTGTCTCAAAGTTTGACACTAAAATTCAGTGTTTTATAGTGTTTATAATTAAGTGTTTTGCATGCATTGCAGAAATTTTCATCTTTTTTAATTGGTTCAATACCACATGTCATACAATATGTTGTTTGATTATCAAGATTAACTTTATGAAAGGAAAGTAAGTGAGCCGCAAATTTAAAAGTAAAATATCTTTCATTTAAAATGATCTTATGAATGTATTTTCGATAAGGAGGAATGAAAGCATTTGCCAAAATAAATCGCATAAAAGGCTTGGAAAAACCCATATCTTCTAATCTTTTGTGGGTATAAACCCTATTTTGGTGTTTTACAAAAACTTCATTGTTATAATAGTCGTTATAGCTATCAATCATTTTTTTAAGTCCTATAATGCCCAAGGTTGCACGCATAAAGCCACAGTTTCTGCTCCAAAAAGCATGCACCTGTAAAGGGTGCTTTTCATATAACCAATTACAAAATTTCATTCCGCAACAGTAGCATGTTATTTCAGTGGGGGATGTATAGAATAATCCGGCATTCGAAAATTTTTCATAATTTTTTATGTCATGGATTGCGAAGCTTTGATTTCGTGCATCTATGGAGCTATAGCCTACATATTTAGGTTTTACTTCAAATAATCGCAAAGAGATGTATGGATCTATCGTATTTATTTTAGGAAACATTTCATAATTTTAAATTCTTATATATAATATAAAAAAAATTACAAACATTTGTAATGATCATCCTCAATTGAAGGCTGAGTTGTAGGCTTTATTTTTCTAATTATACGAAGAAGGTAGGTTCTCATAAAGCCTTCAAGATGACTATTGATGTTTCCAATACATTTTCTCAATGAGTTCATAAACCCAGACATTTTGCTAATGGCTTGGCAAAGTGCCAACAAGTTGTCCACAAAGTACTGGTAGATTGCCACTAGCTATAGCTAGCTATAGTGAGCCAACCTCTCTGTATGTATTTTATATATTTCATTTTTTAATAGATTTAATATTTTTATAAAAAAATATTTAGTTTTTTATACAAGAATGTCGACAAAAAAAAGCCCACAATTACCAAGCAAGAGCTTTACTCCTTAGTAGCGGCAGATACCCAGTTAAATAAAGCATTGATTGAAAGAATCTTTACAAGTCAGCAAAAAATAATACAAAATGCTTTAAAGCACAATCAAGAAGTTATTATACCACCCGGAATCAAGTTCACCGTCGTTACGGTGAAAGCTAAACCTGCTCGCCAGGGCCATAATCCCGCCACAGGAGAGCCTATTCAAATTAAAGCTAAACCTGAACATAAAGCCGTAAAGATACGAGCATTGAAACCTGTCCATGATATGTTAAACTAAACTATAAAGTCATATTCTTCTTTATCGTTATTATCTTCAATATATTTTTGCCAATCGAAATCGAATAAATTCAGATCCTGGACATTTAAATACTTATCATCGTACATTTTAATATAATTTAAACATGAGTTGTTGTCAAAAACTTTTAGCGTTTTTGTTAAAATTATCATATGAATAATTTCCTTATTAAGAGTTGCCGGAATAATACAAAACCTATTTTTAGGTACATCATCCATGATAATAGTAAAATTAGTAAAAATTGTTTCTTGTTTTTCTTTTGTTTCAAATAAACGTTGTAAGGTTAAAGGTTTCTCGTTCAATGGTTTCTTTGAAGATAAAAAGAATGTATAATCTGGTTTAAAGGTATTTTTGGTTTCAATCGTGATTCCATCTGCTTGAGCATATACTAAACCAGACCAAATATAACGGTCCACTATTACAATATAATTTAGCTTAAGTAGCACTGCAATTTCTGCGATAAATTCACTACGATGTTTTGTAAATAATTTATGTAATTGTTCCGATGACATTTCTATGGTTTTATTTAACACCTGCAATATAAGATCACCGGTGGTCGTGTCTGGATTAGGAAAATGTATACATATAGCATTATAATCCATGCATTCCAATGTTTCTTTTAATTTCATTGCCTGTGTGCTTTTTCCCACACCATTGATTCCCTCGATGGCAATGAGTATTCCACGCATGATTAATAAAAGGAAAAAAAGAATTCAGTTTTTAACATTTCTTACAAATCTTTTTTTATACAACATTGTACAACACTGCATTAGCGGTATATGATGTTATAGCTTCATTAAATATTTGCTTTTATATAATCTTTACCAACCTATATTTGGTAGATCACTGCAGATGGTCATAAATAGGCCATAACTAAGATAAAAATTATTTCAGACGCTACTACGGTAGTATTATTAAAATCATGTGTGGCAATGTATGACGTCTTAATAGATAAAACATTTAAGGAAAACAAATTTGAATAAAAAAAATAATTGTTATGATGGCGTTGTTACACAAAGAAAAGCTTATAGAGTGCATCTATCATGAGCTAGAAAATGGCGGGACAATATTGCTTCTAACAAAAAATATTGTTGTGTCAGAAATTTCATACATTGGCAATACTTATAAATATTTTACCTTTAATGACAATCATGATCTGATAAGCAAAGAAGATCTTAAAGGAGCAACATCCAAAAACATTGCTAAAATGATTTATAATTGGATTATAAAAAATCCTCAAAATAATAAGATTTGGAGTGGTGAGCCGCGTACTCAAATTTATTTTGAAAATGATTTATATCATACAAATTACAATCATAAATGTATAAAAGATTTTTGGAATGTTTCAACTTCAGTCGGTCCTCATATCTTTAATGATCGTAGCATTTGGTGTACTAAATGCACATCCTTTTACCCATTTACCAACATTATGTCGCCCAATATATTCCAATAAATTAGATATCTTTGCTATTAAAATAGTTAAAAACCTTATAGGATAATTAGGTACTTTATTACGATAAATTATGATATTTTATAATTAGTTACTTTATTATAATTAATCTCTTTATTAATGAATTATCATAAGATAACTAATTATTTTTTTCCATATATCAGATAATAAATCTGATATGGGCTAAAAGTATGTTTCAAACTATTTACAATAGAATTTCTGTTAAGAAAACATACATAATTTGAATAAAATTTTTTTAAATATCACCGAAACAATCAACATGGTGTTAATAGAGTTTTTAACAGGTTTCTTCTATTTATATGGAAAGAGACTGTTTTCCATTAGTAAAGTCATGGACATGATATGTCTAGACTATTATACCATTATTCCTGCTCCTCTGGCGATGATGTTAGCGGCAAGACTAAAAAACTATGACCTCATGAAACGACTGCACGAATGGGAAATCTCTATTGACTACGCTCTACTTGTAGTAGATGATGTGCCGTCTATTGACTATTGCTTAAGTCTTGGCGCTAGATCCCCGACTAGAGCACAAAAAAGAGAACTGCTGAGGGACAACACGTTTAATCCCGTGTATAAGTATCTTATGAACTGTTCCGGCTTCCCAACAAAGAGAGAAAAAAACATTCCTTGTGATGTTCAATGCGAAAGACTGCAAAAAAACATTATAAAAGAACTGGTATTTAACTGCTCTGTACTGCTTGAAATGGTACTGCACACAGAAAGAGAATATGCATACGCCCTACACTGTGCTGCAAAACATAACCAATTGCCCATCCTCATGTATTGTTGGCAACAATCCACAGACGCGGAATCTATTTTGTTGAAAACCTGCTGTTCTGATAAGAACATCAATTGTTTTAACTATTGTATTCTATATGGCGGCGCCCAAAATTTGGATGCTGCAATGGTGGAAGCGGCAAAGCACGATGCCCGGATGCTGATAAACTACTGTGTCATGCTTGGTGGAAGATCCTTAAACGAAGCAAAAGAAACGGCTGCCATGTTTGGACACATTGAATGCGCACAACACTGTTTTAAACTGCAGTCTTACGTCGTGGACACATCGAATACAGACGACACTGATTAAAGCGACAATCTTACGTCATGAACGACTGTCTTTTGAGTATCTATACTTACATTATATTTTTTTATGAAAAAAATATAAAGGTTGTATACAAACCTTTGTATACAAGAAATTTGGATCATTAAACAATAATTAATTTGGACACAGGAAACGATCTAGATCGATCAAAAAGCTATTTTTTTTGCACACAGAACATTTAGATAATTGAGAGATTACTTTCCATACTTGTTAAGCTTTTTTACACACAGGAACTTTGGATTCTGTTCAGGAAGTTTTTCATAGACATTATGTTTACAGCCAGTAATAATAATTTTGGGCTTTTTCTTAAACCACCGGTGGAAAACATCCAGCTTGTAAAGAGGGAAATGCATGTAGAGAGGTTTTGGTAGTCATGGTTAAGAGATTTGACTAACTCCATGTTTCCTGTAAAGACTGCCCAGTCCCAAGCAGTAAAACCTCTATGATAGTCTTTTTGAGTCGGATCTGCTCCAAATTTTATGAGAGAAAGCATATTTAAAGAACGGCCCCGTATTGCGGCCTTCATCACAGGAGTCATCCCATTAAAATTCGGTAAACAAATTCTGGTCCCATTTTTTCCGAAATAGCCCAACACCCCTTCCAGGATTAAATGATTTTTTTTCTCAGCTAAATAATGTAAAGCAGAGTTTCCATCTTTATCCCTCCTATGAGGGTTAATTATTTCTCCAGGATAAGATTCTTGTTCAAAAAGAAATTTTAAAAAGTCTATACGTCCGTAGATGCATATCCACATGAATACCGAGGATCCATTTTTATCGCATCTATTGACAATCCACGGATCTGTTTTAAAAAATTCCTCAAATAGTGTAAGATTCCCATTTCTAATATGTTTTTTAATCCATTTAACAAACAAGTTTTCTATCTCCCTTTCTGGAAACATGTGTTCCATTTTGAATGTCGCCCCTACTCCACTATATGATTTTACTCCTTTAATTTTTAATGTCCTTTTTTTTCGGACTTCTTTGGATAAGCTGTTTATTACCATCTTTAAATGCCTTATAGCGGGGAGGAGCCAGGCCCTTTTCCCATATGTGCGGTAATTCTTGGTGTTTATGCTTGCCTTTGGCATAACCAGGCCAGTATTTTTCGATATATTCAGGGTTTGTTTTTACGTATTCTTTAAAGGTCCGATAGGCTTCTTGAATACAGGTAGGCTCACCGGTATAATTTCCATGTTCATCTTCCTTTAAAAAGCCATTAACCCTGTCCTTTCTCCACTTAAGATTGTGCTTTCCAAAAATGCGATCAAGATCTTGCGCCTGCTGGGGTGGAATCATAAATCCCTTTTTAGGTCGAAGCTTTTTATTTTTTCCATAGCTTCGGCCATCGCGTTGCGAAACAGTGGTTAGGACGCCTGATAGTCTTTCCATGGGCGTCGCATCTAATCCTATCCATCCACCCTGATGAATATCAATGGCAACAAGCTCTCCTTTATTTTGGGCAAGCCAAGTTTCCAAGAATGCCATGCTTTCTTCCCAGGGATAAGGCCCGCCAACACCACGGGTTGTCCAATCTTGCAAGGACTCCAGGTCCGACACCTGGTAAGGCTCTAAAGAAGACGGTTCCTTGTTTTTGTACTGCAAATAAGATTTAATGACCCATTTATACCATGTGTCGAACCGCAGCGTGGCGCCTCCAAAGTGAAAGCCGTCGTTGATTTTAGGATATCTGCAACATATTTCAACCGTACGTTTGAGTTCTGCAAAAGCGGCCTTCCAAGGAAGTCTTTCGCTGCGGGTAAGACGGTCTATTTTGCCCTGCGTGCCATAGCGTATGGCATGTCGTGCCAATTGCAACAATTCTGACACCGATCCGTGGGCCCCGATCCAGTTTATCGGATAGGCAACCTCCGAAGGGTTTAAAAGATGCTCGTAAAAGCGTGGATCTTCAGATGCCAAGGCGTCTGCAAAGGGGATAATGCTAGAAAACCTGTCTAGACATACGTTTTCTGTGTTTACTTCTAAAGGTAGAAAAATGGTTGCGTGAGGCTTTTGAACCTGCTTGTTCAGCGGTCTGCATATGCTTTGAATAATGTCTCTAGGACTATGTCGCGGCGCTGCAAAAAATACCGCGTTTAGTTCTGGAACCTCTACGCCCTCTTGAAAGAGTCGACAGTTTAATAAAATAACGGGTTCCTTTGAGGAACAAAATTCTGTAAATGTTTTGAGGATAACCTGTCGCGGCAGGGTTGAGTGAGCTATCAGGGCATAGACCCCTTGGTCTACCAACGCCGCGTATAGCTCCTTGGCCTGTTTAATATCACGGGTAAATACCAGCATTTTAGGAGCCGGTATATTGGTTTTTAAATAGGCTAAGGCCATTATAATTTGCTTTACTATGATCTGTTTCGTGGTCTCCTCTTTGGTACTCGGTTGGTGGGCCAATTTAGGCGCGGCTACCATCTGCAATTCAAAATCATTTACATAGCCGGCCTCTATGCCTTCTCGCAGATAGTAGCGAAAGGCAACGCCGCCAAAAAGTTCACGATTTTTCATGGAAAGCGGGGTGTCGTACCTGGGCGTTGCCGTTAAAAAAAGTCGGTGCCCTTTTTTAAAGTTGAGCAACACGTGGGTAAAGGGCCGTGTCTCCCATTCGCCGCAAATCCGGTGACATTCATCGCTAATAATAAGATCGAAATCATCCACCAGTAGCGTGGAGGATTGGTAGGTGGCAATCACAAGAAGAGAAGGGGCCTCCCGTATCCGTTTTGCAATAAAGACAGGATTGGTGGTCATTTCTATATTGTCGTGATTTAGCACAATGCGGGTCTGGTCAGACCCCACAAGCAAAACGTTCTTCAAAGAAATTCCATACTGATAGAGTTTTTCCAGAGTCTGCCGTAGTAGGGACAGGCCCGGCACCAGGTACAAAACTTTTCCTTGAAGATAATTGGAGAGGATAAGATAGGCGACGCGAGTTTTGCCGCATCGGCAGGCCATCTGCAGAATGGCCCTCCCACTTCGCCGCAGCTCCTGATAGCCCATATTGGCCGCCTCCTTCTGATAAAGTCGATCCTCGATTGCAGTCCGTGTCTCATCTGTAGAAAAAAATAATACGTCATCTGCGAAATGTTCATCTTCCACAGGAGTTATCACCAGGTGTCTCAGTTTCTCCTTGCTTATCAGCGGATCAGAGGGCAAAGATGGCTCAACCACTATCGTGGAATCATTCATCTCATAGGCGGGAGAATCACACAAAGTATAGCTTATGTCCAGACAGTTTGCAACATCCTCAGCCAATTGTTTTATTTTTTCGGGTAAAAGACATACGAGTTCTTTGTTTTTGACGCGAAAAAACTGTGCACAATATAACACCCCTGCTTCAATTTTTTGCGCATCCTTCTTTGTAGATGTTTCCAATGTGAAACAATACTTCCATTCATCCGTAAAACAGGTTGTATAAGATCCATCATGAAGCCTAGCGGCCAAGTTTCCTGTGTGCCCAACTTTATGTAAGGATTGGGCCTCCAGCCAGGGATGAACCGCCACGTAAAATCCTGCGCACATGCTATATCAAATTGCAGTTTCTTAATAACTGTACACAGGATCTGAAAAACATGTGATTACAAAATTTAGATAAGAAATATTTAATATTAAAAATCACAGAATACATGTCACTGTGTAGAGAGAAAGCCAAAAACTCCTCTTGACCGCCGTGGGAAATCATCCAGGGTAGTAGGTTGTGTTTCATAAAGTTGTATGCCGTAGTGATCACCGTGGACTCCAGATGGTTATTGGCATCTTTGCAATACTTTGCCATCTTGGCAGAAAAGACGATAAATCCACAAATTCTACCCCAGTTGATAAGATCCTTAAACAGCTCAGTCACAACCCCAGTAAACTGGGTTTTAATTTCTTGAACACTCGTAAGAGAAAAGGTAATTGTAACCTGTTTGTTCAAACACTCATCATAATAGGTTAAAATTTTTTTTATTTGTTGTTGATATGGGCTAAGCTCATGCTCTGAAATATCATTAATGTAATATTTAATATATCCCACTAGTATTTCATTAATGATATTATGATATATTAACTCTTCTCCCTCCATAGCGGCACCCTATATTTTTTTATTTAGGTTTCAATGTTATCACAATTGCGATACAATTGTGATACAATTGTGACACAACTGTGTTGTATACAACAAATGTTAGGCCACGTATAGCAACCTATATGTTAAGAAATATTTTTATCCCAACATTAGTTGGAAACGAGCAGCCGCAAAGAAGTCATTTAAAATAAGCCATTTAAAGATTTAGAATTTATATGTATACAACTGTACAATGGAAGCAGTTCTTACCAAACTCGACCAGGAGGAAAAAAAGGCTCTCCAAAATTTTCATCGTTGTGCTTGGGAAGAAACTAAAAATATTATAAACGATTTTCTTGAAATCCCTGAGGAACGATGCACCTATAAATTCAACTCATACACAAAAAAAATGGAGCTTTTATTTACCCCTGAATTCCACACCGCCTGGCATGAAGTTCCTGAGTGCAGAGAGTTCATATTAAACTTTTTGAGACTCATTTCGGGACATCGAGTGGTATTAAAAGGCCCTACATTTGTTTTTACAAAAGAGATCAAGAATCTGGGCATTCCTAGTACCATCAATGTTGACTTTCAGGCCAACATTGAAAATATGGATGATCTACAGAAGGGAAATCTCATCGGCAAGATGAATATCAAAGAAGGCTAAATAAAACAACTAACATCAAAAAACATTAAAGGCTATGTTGTGGACGATGCCTTTGTCTCAATAGTTTCGAGGTCATCCAATAACTCATGTAACGTAAAAAAGTTGGTCCATTTTTTTGAAAACATTAAAAGACGTTCGTCTTCATAAATAAAAAAGTCATTCGAAGGAAAAATGATATACTCAATACCATAGTCTTGTAATATTTTTTTTAGGTCTCTCAGGGTCCAGGGATTTACCAGGCTTCTACGCGAAGTGAGCATCATAAAAATATCTAATATTTTTTGCGCCATAAGCCAGCGCGGATTCTCATTGGCCCACAAATCAACAATAATTCTCTTATCAACCGTGAGCATTCCTACTTGATTCGAAGAAATGATTAGATGCCCAGCAGTCCACCCCATGAGTAGATAACGCAGCGTTGTAGAAATGTCACATATGGAAGGCATTCCTCCACAACATGAACCCAAATTAGGATGCGTGTGAAACACAAACATAGCAGGCTTGTTGGCCACCCTGCTATAAATATCAGCAGGCATCATAGCCTCGCTGCCAAAATAAATGTTCTCTCCTGCCCTATAGGGGCTTGGAATGATTTCCACTATCTCGGGTACACCGTTTATCATATTAATGCGGCCGCACCATTCACGGTCATCGTCCAAAAATTTTTTGATGGCACCCCGAACATTGTCCCAGTTAAGCAACAGAGTATTCACAATCTCATTACGCTCCGCCCAGTATTCCTTAAAACTTCTTTTAGACTTGCTGAGCTGTTCCCAGGATTCGAACTCAGTCCAATGTTTTTTTTCTTTTGGGGAAGACTTCCCTTTTGAAACATTTTTTGCGGCTCCACCATCTACACTATGATTTTCCAAAATAATCTCCTTCATCGTTTGAGTTATATGGGCATTGCTAAGCACCTTAGTGGTAACCTGTTTACCTATGTGATTTAGCAGAAAACCAAGTTTGTCCATTTGTGTCTCAACCATTTATTCTTAACAAAACAAAAAAAAATTAAAAATCATCGTCGTTTAAAAAGAGTTTGAAGGCAAACGCATCATCCTTAACACAGTTCTGATACTGCGTAGGTCTTAACTCGAAAAAGTTGGTTTTTTCTACTTCATTAAGAAAGAATTTAGTCATCTGAGGAAAAGGGTTTCCCACCTTATAAATGCTTTTGCACTGCATCATGAAGCACAAATTATCTGTAAAGTAGCGTATATATTGAAATAGCATTTCTTTTGAAAAACCGGGAACTCTTCCTCTTGCCTTGTCAAAGGCATAGTTAATAAACTCATCCACCAACTCCACAGCCTCCTTCAAAATTTTGTGAATGATCTTTTCCTCGGGAATGTTATACACGTAATTTGAGATAAGAAAACACGCAAAACTACAGTGCATCCCTTCATCACGTGAGATAAACTCATTATAGCTTACAAGCCCCGGCATAATATTCTGTTCCTTAAGAAACTGGATCGCCACAAAGTGGTTTTGAAATAAAATGCCTTCTACGGCGGCGAAGCCCACCAGCCGCTCACCTAGAGTGTTCCTGTCGGGGTCCATCCACTGCCGCACCCACTGCGCCATTTTTTTTATGATAGGGTGTTTTTCAATGCCGCTAAAGATGCGCTGTTGTTCCTTCTCATCCGGGATCAGCGTTTTTACCTGTATTGAGTAGGCTTCGCTATGAACGCACTCTTGGGCAGCCTGCATTGTATAAAAGTATAACACTTCCTTTACTTTAATTTCGCGCATAAAATTGGTTAAAAGGTTTTCGATAACAATTTCGTCGGCAACAACAAAGAAGGCTAAAATTTGTTTATAAAATTCGCGCTGTGGCTTTGGCATGGCTTCCCAATCATCAATGTCCTTACACATGTCCACCTCCTGCGCCGTCCACGTCAAACTTTCTAATTTTTTATACCAGTTCCAACATTCGGGGTGCTGAATAGGAAAAATAGTGAAACGTTGGGAATTTTCAATTAGTAATTCCTCCATATTTGAAATAAATATTAACATCTTCAAATTTATTGGCTGCCATGGAGACGTTTTTTATTGAGACGTTGGCATCTGATGTGTATGGAAAGGCGTTAAATGTTGATTTAGATAGACTATCGCAGGCGCAGGTTAAATATACCCTTCAAGAGCTTATTTCCTACTGCAGCGCTCTAACCATTTTACATTATGACTATTCAACCCTTGCGGCGCGTCTTTCGGTGTACCAGCTGCACCAGTCAACGGCCTCCTCCTTCTCAAAGGCGGTGAGGCTGCAGGCCGCACAATCCTGCTCACGCCTGTCCCCCCAGTTTGTGGACGTCGTTTACAAGTACAAAGCCATTTTTGACAGCTACATTGACTATAGCAGAGATTACAAGCTGTCCCTCCTGGGGATAGAAACCATGAAAAATTCTTATTTGTTAAAAAATAAAGATGGGGTCATCATGGAACGCCCGCAGGATGCTTATATGCGGGTTGCCATCATGATCTATGGGATGGGAAGAGTGGTCAATATGAAAATGATTCTGCTAACCTATGACCTGCTTTCCCAGCACGTCATCACACACGCGTCGCCCACCATGTTCAATGCAGGCACCAAAAAGCCACAACTCTCCAGCTGTTTCCTGCTAAATGTAAATGATAATTTAGAAAATTTATATGATATGGTCAAAACGGCCGGCATCATTTCAGGCGGCGGCGGTGGAATAGGGCTGTGCTTGTCAGGAATACGGGCAAAGAATAGTTTTATTTCTGGTAGTGGTCTTAAAAGTAACGGCATACAGAATTATATTGTGCTGCAAAATGCTTCACAATGCTACGCGAACCAGGGAGGCCTACGTCCCGGAGCCTACGCCGTCTACTTAGAGCTGTGGCACCAAGACATCTTTACATTTTTACAAATGCCTCGCCTAAAAGGACAAATGGCTGAACAACGGCTTAATGCCCCTAATCTCAAGTACGGCCTATGGGTCCCCGACCTATTCATGGAAATACTTGAAGACCAAATACACAACAGAGGCGACGGCAAATGGTACCTCTTTTCGCCGGATCAGGCCCCCAATCTACATAAGGTCTTTGATTTGGAACGGTCGCAGCACGAAAACGCACACCGCGAATTTAAAAAGCTTTACTATCAGTATGTTGCTGAAAAAAGGTACACCGGCGTCACAACGGCCAAAGAGATTATCAAAGAGTGGTTCAAAACAGTTGTTCAAGTAGGGAATCCCTATATCGGGTTTAAAGATGCCATAAATCGTAAAAGTAATCTTTCACATGTAGGCACTATCACGAACTCCAATCTTTGTATTGAAGTCACAATCCCCTGCTGGGAGGGTGATAAGGCTGAACAAGGTGTTTGTAATCTGGCCGCAGTAAATCTAGCCGCCTTTATACGTGAAAATGGCTACGACTACCGTGGGCTCATAGAAGCATCAGGCAATGTCACAGAAAATTTAGATAATATTATAGATAATGGCTACTACCCCACAGAAGCCACGCGGAGAAGCAATATGCGTCACCGACCTATTGGCATCGGGGTCTTTGGCCTAGCCGACGTGTTTGCGTCTTTAAAAATGAAATTTGGTTCACCCGAGGCCATTGCCATGGATGAGGCCATCCATGCGGCCCTATACTACGGGGCCATGCGACGATCCATAGAACTTGCAAAAGAAAAAGGAAGTCATCCCAGCTTTCCGGGGTCTGCGGCCTCAAAGGGTCTACTGCAGCCCGACCTATGGGTTCGCTGTGGTGATTTAGTTTCCTCCTGGGAAGAACGCGTGGCACAGACGACGCAGGGTGTGTTGACGCCGAAAAGGTGGTCGCAGCTACGCCTGGCGGCTATGCAGGGACTTCGAAATGGATATGTCACAGCTCTTATGCCCACCGCAACCTCCTCAAATTCTACAGGAAAAAACGAATGTTTTGAGCCCTTTACATCCAATCTATATACACGTAGAACGTTAAGCGGGGAGTTTATTGTTTTAAATAAGTATTTAATAGACGATTTAAAAGAAATTAATCTTTGGACAGAAGCCATTCAACAGCAGCTACTAAATGCGGGAGGTAGCATTCAGCACATTTTGGATATACCGGCCGAGATCCGCGATCGGTATAAAACCTCCAGGGAAATGAATCAAAAAATTTTAACAAAACACGCGGCCGCACGAAACCCCTTTGTATCCCAAAGTATGTCCTTGAACTATTACTTTTATGAACCTGAACTAAGCCAGGTACTTACAGTGCTCGTCCTAGGCTGGAAAAAAGGTTTAACTACCGGTTCCTATTACTGTCATTTTAGCCCTGGAGCGGGTACCCAAAAAAAGATTATAAGAAACTCTGAGAAAGCGTGTAATGCGGACTGCGAGGCGTGTCTTCTGTAGGTGTCTCGCGGTAAAAGAGCAGCGGGGACCATATGGTAAACCCCAACAAGAGGATAATGAATAAAAAAAGTAAACAGGCATCCATTAGTTCCATATTAAATTTTTTTTTCTTCTATATAATGGAATATTTTGTTGCGGTAGACAATGAAACCTCCTTGGGGGTTTTTACTTCTATAGAGCAATGTGAAGAAACGATGAAACAATACCCCGGCCTCCATTATGTCGTTTTTAAGTATATGTGTCCGGCGGATGCAGAAAATACAGATGTTGTATATTTAATACCCTCGTTAACCTTGCATACCCCCATGTTTGTAGACCACTGTCCAAATCGTACCAAACAAGCACGACACGTATTGAAAAAAATAAACTTAGTGTTCGAGGAAGAGTCTATTGAAAATTGGAAGGTTTCAGTAAATACTGTGTTCCCCCATGTTCACAACAGATTATCTGCGCCGAAACTTTCCATCGACGAGGCTAATGAAGCCGTAGAAAAGTTTTTGATACAAGCAGGACGACTCATGTCTCTGTAAATGTCTCTTCCTTTATGGGTGACGTCTCTTCCTTTGCCGAGGAAGTCTCTGTTATGGGCAAGAGGTTTGAAACAACGCAAGGACTCTGCTTAATCTGCTGTCTCACAAAGGGAATCAAACTACCTGCTTTCGTATTTTTAATGTAGTAATTACCCTTGTTGTGATGAATTTTAAGACCATAGCGTAGTCCCAGTACTTTATTAATGAATTTTAAAATTGTTTGAGGGTCCGTTTTATTGGGCTTTTTAAGCTTAAACTCAAAGCTGATCGCGCTTAAATCATACTGAACAAATTCATCAACGAGTTTCGTCATTAATTGTTCATTGGTCAATATATTAGGGTCCTGAACGCATTTAAAGCCGCACTTAGTTAATAGCATAATAGCGTACATATGAGATTGAAAACTATAATTAAATTGTAGATCATGATGCTCTGCGTGTTGCATGGCCCATTGATGAAAGTTTAATTCCTGAGTTTGTAACATAGTGAGCGACTCGTATACTGTCTTTCCGCGGCTTATTTGGACACGGCCAGTATAGTTCTGTTTTGTCATAAAACTATTGTATTGTTCAACAAATTTGGGAGTAATTTTATGACCGTGCCATGCATAAAATTCGAGTAGTTTATACTTTTCATACGCAAATAGGTCTTGCTGGTCTACTGTGATGCCTTCCTTTAAGTTTTGTTTAATTTGTAAAGCTTTATTGGCATCAATGGTTTCAGCCGAGGCAATGTTTACATAGTCCTGGTGTTTAATTTCCATTTTAATGCTTGTATATTGTTTGACTGTCTCCAGCTTTTCACCCGTCAGTATAAACACCTTAGCGCCGGTGTCGGCGATCTGGTTAATAAATCGGGTTATAAAGTGATTTTTTGATAGATGTTGTATCCGCATTGTTTCGAGCCATAGATGGTAGTATGGAGTTTTATAATATATCGGCCTACCTGTTTCCTTACTATACGTGAAGGAAAGCTGGTGATTGCTTATGGTCTGAAAAAGGGTGTCACGTTTTTGTAACGTAAACATTTCAATGTCTTCGATGGTTTCTGGATAGTAATTTTGTTTCCCCTGTAAGCAGATTTTATAACACTTACTTTTTAATTCACGCACGCGGCCCAACATTTGGCAACATGTTTCTACGTCACACGACATATTGTTAAAAAAGCCGTATAAAACATCAAATCTCTTATCTTCGTATGAAACACCCGCTGAAATCGTGGGCGTATAGATAAGGATATCAACGAGCCCCCAATAATACGATACATTATTAAAATGGGATTCCCGTTCATGAGCAGTGCTTTTAGAACTATAAAACCCAATTTTTTTTTCCGGAAACTTTTTTTGGATAAATGATTGCAACAGCCGGGCCTCCATTAATGAATTTGTAGGGATAACAATTTTTTTGTCTTCTAGCAAATCCTTTAAAAGGTTATTTAACCAAGTTTCTCGTGAAGAGGTAAAATAATACGTGTCATGCTGGGCCCTTTTATATTGATTCCAGTGAAAGAAGATAGGGACATCCCCGCGAAAACGCTGTAGAATATTATACGTTCGATTTCCTAGGTTTGCGTCCAAGCATATAACATAATTTGCCGTTTCGAGCATCCACATGAAAATGGCAAAAGAGGGAGCAAAGTATTTGTGCAGGCCGCTATTGAATTGATTAAAAATCGATTCTACCTCATCCAAAATAAGTAGGTCTACAGGCTCGGCTGTGGAGGTTAGCCGGAAAAGTGATTCTACCTGAATGATGACTCTTTCGTAGCTGTCCAAATCTCCAGTTACTTCGCTGTACAATGTGAAATTCGGTAGCCGGGATTGTATATTTTTTGAGAAGATCTGTCGAAACGTCACAAACCGTATGGTTTGTTGTTTTGAAATAGAATTATTGCCGTAGTATTTTTGCAAATAGTTGCGCAGTTGGACGGTTTTACCTATTTTCATTTGAGCCTTTACAACAAGCGTAGGGACTCGTTCATATTCTCGCATACTACTTTCATCATAGATGTGTTTTTGAGTATCAGGCAGTTCTTCAAAGAGAATGGACTCATGAACCTCTATGCTCTTTGTCATCACTTGGTCCACATATGTTTCCACAAAATTATTTGTGCCGGAAAGGCTGCCCATGAGAAGGCTATGTTTATTGTCATGGCGACAGTGTTGATACACTTTGTTTCCCGTGACTCTTAAAATTAGGGTATTGTCCTTATCATGCATACGCTTACATATTTCGCAGTAACTTGGACTTGTACGTTTAAACAATACTAAATTTTTATGAACACGGAGGAAGCAATGATTTTTACATAGTGTTCCTGCAAATTTTAATACCTCTTCAAGTTCACTTTGTTGGATAGTATCGCAGGAACTCGGTGTTGTTTCTTTTACATTTGTGAAGATACAAGGTAAACACGTCGTTTCAAAGGGGGTTGCTATAAGGGTATCACTCTTTTTCGTGGTTGTACTGGTCTCAAACACCTCTGCAAGCTCCTCATTAAACATTTTAACACGCATGCTACCTTTTTTATGAGACCCTATGATGCGAAAATTTTGAATACTTTTGTTGACCTGGGGGTCAACAAAAGGATAAACGTGTTTGGGAAGATTTTCTAACACTTTGGATGTAAAGACTTTGGCCTCATTATTGTTTAATACTGAGTATGTATAAAGTATGATATGAAAGGAGTATTTAAGTTCTCGCTTTTTATTTAATCCGATAGAATCTGTTAGCAAAATTTGTTCACGCGTTAGATTGATGTTATAAGGTAAAGAATATGTCTCGTAAAATACATCCATGATGACGTTAATTATCATGTCAAGGATGTCATAGACATTGTCTTCGACATTATCATTGTCATCAACATTGTCATCAGAGTATGACTTATTTACCGGAAAGTCGATGTCAAATTTTAAGCGCTGAGGCAAAAACCCAAATACCACTTCGTGGAAACACTTCTGCTCAAAGGGCTGAGCCGCCTCCCACTCCCAAAAGTCATCACGACTTGAAAAAACTCTAAAAAGATTATTATATTCATCTCGCACCACGAAGTGATTCTTTAAGGTTTCGAGAGAATATTTATCCTCTACGGCTTCTCCTTGGGAGTTACAGCGAAGAAACTTGAATGTTTCTTGCATTTTGATATTTAAAATTAAATCAATTATGATGCGGCCGCTAATGCGGCGGTTGACGCGGCCGCGCCGCTGACGCAGCCATCATACATAAAGCGGCATGGCCGTTTTATAACGACTAGTCGGCCGTTATATGACGAACTATATAAAAATGAATTCTTTTAATTAGAGTTAAGTATTGTTGATTGTATAATCCATCATGGTTGAGCCACGCGAACAGTTTTTTCAAGATCTGCTTTCAGCAGTGGATCAACAAATGGACACTGTAAAAAATGACATAAAAGACATTATGAAAGAAAAAACGTCTTTTATGGTATCATTCGAAAACTTTATAGAACGTTACGATACCATGGAAAAAAATATTCAAGACCTTCAGAATAAGTACGAAGAAATGGCGGCCAACCTTATGACCGTCATGACGGATACAAAAATTCAGCTTGGAGCCATTATCGCCCAACTTGAGATTCTAATGATAAATGGCACTCCACTTCCGGCAAAAAAGACAACAATTAAGGAGGCTATGCCCTTACCTTCATCAAACACGAATAATGAACAAACGAGTCCTCCCGCCTCAGGCAAAACAAGTGAAACACCTAAAAAAAATCCCACGAATGCGATGTTCTTCACGCGTAGCGAATGGGCATCCTCGAATACTTTTCGAGAAAAGTTTTTAACACCAGAAATTCAAGCCATATTGGATGAGCAGTTTGCAAACAAGACCGGGATCGAAAGATTGCATGCCGAGGGTCTTTACATGTGGAGAACCCAATTCTCTGACGAACAGAAGAAAATGGTCAAAGAGATGATGAAGAAGTAATATTTTTGGTAAAAATATTTTTATCAAAATTTTTTTACCAAATAATAAAAAATATTTTTTACTTTTTTTTCTTCATAATATACATAGAATGCCTACAAAAGCTGGCACAAAAAGTACCGCAAATAAAAAAACAACGAAGGGCTCCTCCAAATCTGGTTCTTCCAGAGGCCACACCGGCAAAACCCATGCTTCTTCGTCCATGCATTCCGGGATGCTCTATAAAGATATGGTAAATATTGCTAGATCTAGAGGCATTCCGATTTACCAGAATGGATCGCGTCTTACTAAAAGTGAATTGGAGAAAAAAATTAAACGGTCAAAATGAATATAATCAGGAAACTTAAGCCTGGAACAATTAGCCTTGTGCTGGGACCCATGTTTGCCGGCAAAACTACGTTTCTTATTCATTGCATTTACATGCTCGAACGTTTGGAAAAAAAAGTAGTCTTCATAAAATCTACCAAAAACACCCGAGACAAAACTATTAAAACACACTCCGGTATACAGCTACGACCCAAACAATGTAAAATCATAGAAAGCACACAGTTATCTGACGTGGGTTCTCTCACCGATATCCATGCAGTTGTCGTAGATGAAGCGCATTTTTTTGACGATTTAATCACATGCCGCACTTGGGCAGAGGAAGAAAAAATTATTATTCTTGCGGGACTCAATGCTTCCTTCGAGCAGAAAATGTTTCCGCCCATCGTTCGTATTTTTCCTTACTGCAGCTGGGTTAAGTATATTGGCCGCACCTGTATGAAATGTAACCAACATAATGCATGCTTTAATGTGCGTAAGAACGCAGACAAGACGCTTATCCTTGCGGGAGGAAGTGAACTGTACGTAACATGTTGTAACAACTGTCTAAAAAATACATTTATTAAGCAGTTGCAACCTATTAAATATTAAAAATCTTATACAATAATGGATCATTATCTTAAAAAATTACAAGATATTTATACGAAGCTCGAGGGTCATCCCTTTCTTTTTAGCCCGTCGAAAACCAATGAAAAAGAGTTTATTACTCTGCTAAACCAGGCCTTGGCCTCAACGCAGCTTTACCGCAGCATACAACAGCTGTTTTTAACGATGTATAAGCTAGATCCCATTGGGTTTATTAACTATATTAAAACGAGTAAACAAGAGTATTTATGCCTGTTAATTAATCCTAAACTCGTTACTAAGTTTTTAAAAATAACGAGCTTTAAAATTTACATTAATTTCAGGCTGAAAACTTTTTATATAAGTCCTAATAAGTATAATAATTTTTACACCGCTCCCTCTGAAGAAAAGACTAACCATCTTCTAAAAGAAGAAAAAACTTGGGCAAAGATTGTTGAAGAAGGAGGAGAAGAATCCTAAGTCGCTTACATTTTTTTTTGCTATTTTTATAGAATGTACACGCATGTTGATGTTGTCGGAATAGCTGAAGCCTCAGCGGCCCTCTACGTGCAAAAAGATAGGGATCGCTACTTAGACGTGCTAACAACCATTGAAAACTTTATTTACCAACACAAATGCATCATAACAGGGGAAAGCGCCCACCTACTCTTTTTAAAAAAAAATATTTATCTTTACGAATTTTACTCCAACAATGTGGCGGAGCACAGCAAGGCTTTGGCGACCCTGCTTTATAAACTTGATCCGGAATACCTCACTCGTTACACAGTACTCATTACCAAAATTCCCAACCATTGGTATGTGATTAACGTAGATCAGCGAGAATTTGTGCGCCTATATGCCATCCCGGCAGTTAAACAACACTTACCGATTCCCATTTTACCCTTCTATTGCACCAGCGCACTCACCCAGCAAGAATTGTTTTGTTTAGGACCTGAACTGCAGTTAATACAAATATATTCCAAGCTCTGTAACCCCAACTTTGTCGAGGAATGGCCTACGTTGCTCGACTACGAAAAAAGCATGCGGATGTTATTTTTAGAACAGTTTCCGCAAAGATTGGAAATGACGGGCGGGAAGAAGGAGGAGAAGGAAAAGCATGAAAGTATCATTAAAAAAATAATACTAGAAATGGTCTCTACCCGTCAGCGAATCGTTGTTGGGGGTTACATACAAAAAAACCTGTACAACCATGTACTCAAGAATAGAAATCGTTTACAGCTTATTACGAGCTTAAATATTTATGAAGAAAAAGATATCATCCAGCAATTTTGTGATTCAAATGGACTGAAGATCAAAATACGTATCAACAATCCGCTCTTGCCTACAAATCCGGAATTACGGCGTTTGACTATTTATTTTAATCATAATAATGATGATGATCAGTCATATCTAATAGTAGATATGTACAACACGGGAAGCTATGAGCTAGTGCCTACAAATCAGATAAACACGCTTGATGGCAGCTTTTTAATAGGAACACCCTTCGTGCAAGCGCGATTTTTGTTGGTAGAGATCTGGGTGCTTATGCTTATTGCGCAGCAAACTAAAAAGGACACCAAAAAAATAATACAATTTTTTATAAATCAATATGAAATGCTTATGAATAGTCCTTGGCCCAGTATGGAGGCCCTTTTTCCCTCAAGCAGTAAAAGATATTTAGGCAACTATGTAGACCCTAACGCGCTCATAAAGTGGGCACAACTCAAATTAAAAAGAATACCGCCTTTTTATCCTGGAAAGCCGGATGAAGAATCATGTTAAGCCGATTAAAAAATCATGTTAAGCTGGTTGAAAAATCATGTTAAGCTGGTTGAAAAACTCTTGGTGAAAGCACGGATGTAATATTAACATTGGCCGCTCGCATTTCGTGTTGAAATACGATGGAAGAGCGACGGCTATCTACCATGCCGATATCGGCCTGGACATCACAGTTCATGCACTTGTAGATGGGATGACTCGCGTTATAGATGGCAGGCTCGCCACAGTTTCTACAGATGTAGGAGATGCAGCCATCCGAGTCGTCGTGCGATTTTTCTATGATGGTTTGCATGGCGCCCTGCGCCGTAAGCACCCAATGCTCCATTTCTCCCAGACGAAGACCTCCGTGCGATCGTTTGCCGTCCAACGGCTGGCCTGTGAGGGCATCCGTGGGCCCATAGCTTGCAACGGCGTATCGGTCATCCAGCACAAATTTTTGCAGGCGCTGGTGATAGGTCGGTCCTATGAAGATGGCCGCATCAAAGTACTCGCCGGTCTGGCCGTTGAACATTTTTTGGCATCCATTGAAGCGTAGACCTTCTTGCGCCAGTCTTTCTGAAAGAAGCTGCACATTAATAGGCAGGAATGCGGTGCCGTCTGTTACCACCCCCTGTAGGGCATTTGCTAGACCAACCGTGGTTTCTATCATTTGACCGTTGGTCATTCGGGAGGGATGTGAGTGGGGGTTTACAATGAGGTCGGGCTGCAATCCGTCCTCTGTGAAGGGCATGTCTGAAGTGGGCAGGGCCAGCGCCGCAATGCCCTTGTTCCCGCTGCGAGAACTCATTTTGTCGCCTATATTGAGATTTCTTTCATAGCGCAGGCGCATGAGGCCAAAGATCTCGTCATTAGGCCCATGGGGACGCATCACAGCATCCACGACGGCCGGCTCATCGAAGCCGTACATGACAGACCGGTCGATGTATTTGTTGAGTTCGTCTTTTTCGCCCCGTATTTTGGCCACTTTTCCTATAATGATGTCGCCCTTTTTGACCACCGTTCCTACGGGCACGAATCCATCTACAAGCTTTTCGTAATTAGCACCAGGCTTAAGATTTTTGGTGATTAAAGGGTCGGGCTTCCCAAACGACTCTATATCGCTTTCTAATTCTACTTTTTCTTCTCGGTAGAAGGTGCCGGCAAAGCCGCCCCTGTCAATAAAGGACTGCGACACGATCACAGAGTCCTCCTGATTGTAGCCGCCGTAGATCATATAAGCCACAATGGTATTAAGCCCGTTGGGTATGACATAGTTATGTGCTATGGTCTTTACAAGCGGCATTTCATTGTAAAACTGGAAGAAGCGGTTCATGTCGACACGATATGGCCAGCTAAAGCAATACCAGCCCCCCGTTTGCCGGCCTTGGTTTGTTTCATAGGTAACACGCGCAGGTTGGGTACAGTTTGCGTAGGGGGACACTAGGGCGGCAAGGCCCAAAATAGCTTGGGGCACGTCCACGTGTGTGAAACGACGCGTTACATCATGTTTATGTTTGCGTAGCTCGATGATGGAGAAGGCAACAAGACAGTTTTCCGCCTCCTCGGGGGTAATGAACTCACAGATGCCCTGTGCTACGAGATCTTCAAGTGTAAGCGTTCCGGCTAAAATGTCTTTTGCCATTTGAGGCGTAAATCGCGTATTTTGAATGAAAGGGATTTTATGTTTTTCCCAGTCTTTATCGCCTTTTTTTCTGGCCTCTGCGGCCTTGTAGCAGGCTTGATTGTATTTTTCAATATTATTATCTACAATGAGTAGGGGGCGGGTCAGCCTACCGACGTCCAACCAAAATTCTACTTCGTCTACCATGCTATCCCAGTAGATGGTGGTATGGGGATGCACAACCTTGCCCTCACGGCGAAGCATTCTATACCGCTGAGCAAGCTCAAAGGCATTGGTGCAGCAGCCGATCCATTCTCCGTTGATAAATACGCGCGCTAGGCCCTTTCGTACAATGTCCTTGTTGGAAACATCGGCTAACTGTTGAATGGCCGGATCTGATAGAAGGCGTTGTTTTAACGAAAGTACTTCTCCGGCGGTGCAGACATTGGCAGTGATGGCTAACTGTTTAGACATGCCTACTTTTTCACCAGTATCGGCTGACTGGGCTACGCAGATGTATCCAGGATAGGATGCGTGCACGCGACGCATCATGTCAGCCCTTTCTGTTTGTTTGGATGCGTTGGTGGTGTTATGAGTATTTACCGTACGCAATGCTGAAATGGTATTTAATAAATTTTTTCTTTCCAAACTTTGAGTAGATACTCTGTTTACAATGGGGCGCTGTCGCACCATGATGGTTTTATTTCCTGAAATGATAGACTGTTCCATACTGCGATTAAGATCGGAGGCGGTATTTTTTGATAAAGCGGCAGAAAATGCCTCGATAATGTTTCGCTGAGTAAGCTCCTCAAAGGCTGTTTGTTTAAGAAGTTCTTTGAACCCATTGATGATGGGTGCTATCACGGAAGTATTAAAAATAGCCTTAAAGGCCTTGGCGAGTGAGACCCCTGAGCCGTGCACCCGCTTGGTGCGGTAGCTATCACGGTCCGTGGGTGGAAACACATTCATAATGACAAGAAGTATTTTATGAATAAGCAGGCCTAAAAAGCGCAGCTTTCGTACACGTGTATCTGCGGTTTGGCCCATGTGTGGCAGCAATATTTTGTCTAAAATAGTAAGTTGTCTTTCATTTAAGTATTGTACCGCATTTTCATCGCTTTTGTAAGCAGATGGGTTTGAGACAAATTTGGAAACCTTCTCGGATAAAAACTGGATAATTTTTTCTCGGTTCAGCTCGTGTTGGACCGGTTGAAATATGGGGTCTAAAACATGAATGGATTTTTCCAGAATTTCTATCATGAAGGTATTCACAAGGGAGTTGGATTCTAGATCAAATACCACTTGCTCAATGATGCTGTCATCGCCTGTCATTCCAAACATGCGAAAGATGAGATACCAAGGTATGCGAAGTTTTGAGAACTTGGTGCTATTGATTTCAATGGTAATGGCGCCGGTGGTCATGTAGCGTATAATAATTTGAGAGCTATTTTCGAAGGCACCTCCCGGTTGGGAGATAAACTCGCCGCGAATGATTTCATTATTCCCTTGTTGCATGGTATGGTAATGGATGTGAAGCGTGTTAAAGCGGATGTTTTCTAAGAGGTCTACGACCCATTCCCCGCCTCGGGCTATAAAGTAGCCGCCGGGTTCATTAGGGTCTTCTCCTATTTCTTTTTTTGCGGTTTTTGATAGGTGATGAGTGTGGCAGCGGTTGCTGCCCCGCATGATGGGAAATGTAGATACCTGAAAAGGAGGAATACTTGCTCGTTTTACCTCCTGCCGACCATTGCTGTAGTGCGCCGTTAAAATAACCTCGGCGGCTAGATTAACCGGGCCCGAATAGGAAAGGCCACACAGGCGTGCCTTATTGGGTAGTAAATTTATCTTGTTTCCCTGTGAATAGTTTCGATGTTGCGGGCGTTCAATGTTCACATCTGTAAAGTTAAATTGGATCTGAACTGATTCCCGAAGCTTATCTATTTCAGTATGGTCGCGTTGGTCTTTATAAGTAATATCCACGTTAAACATTTGTTTTACAATTTGCGGAATTCCATTGTCCATAAGATCGTCGAAGCTTTTGATGTTATACCCTATCAATCCTGTAGAGTTTACTGCAGCGGAGATAAAGCTCAGCATATCAGCCTCTGTAAGCTCCTCATTATCCACGGTTTCAATGGGGCCGTAGGTTATTTGCGGCCGCAAGGGTTCCATGATTATGAAGTACTACATTAATATTCAGTTATTCTTTAAAATAAATCTTTATTTATAAATCTTATTTATAATATAAGAATGCCTTATGCAAGAGACATCACAAAGTTTATTACGGCAACGGAACCAGAGGTGGGTCTTCCCCTGTTGGCGCTGCAGCGCTCCAAATCCATCATAGGGGTTATTCTTCTTGTAATAAGTTTGTTATTTATTTTCATTGGCATTATTATATTATCAGTGAGTAGTGGTCATACCACAGCAGCCTCTATATTTATCGTATTGAGTCTTATCCTAGGTGGCGGTGGTTTTTTTCTTATTTATAAAGATAATTCTTAACCCACATAAAATTTGAAAAAATATAGAGTAAGAAAATGTCCAATTACTATTATTACTATGGCGGGGGGAGATATGATTGGTTAAAAACAGTAGAACCCACTAATTTTTTAAAAATCGGGTTGCCTTACCAGGCACACCCATTACATCTTCAACATCAGGCAACTACTCCCCCATCTATCTTAGAAAAATTTAAACGAGCAGACATTCTTCTTAATGAGGTGAAGGCCGAAATGGACCCACTCATGTTACAACCAGAAACCGAAAAAAAACTATTCCAGATATTGAGTAGTATTGATATGTTCAAAGGTCTGCGAAAAAAAGTAGAATTCACGTACAATGCTCAAATTGTTACGAATGCTTGGCTTAAAATGTATGAGCTGCTAAATACCATGAATTTTAATAATACATCTCAGGCATTTTGCAATTGTGAGCTTCCAGGAGGGTTTATAAGTGCAATTAACCATTTTAATTATACAATGATGCATTACCCTACTTTTAACTGGGTAGCTTCCTCCCTTTACCCCAGTTCGGAAACAGATGCCCTGGAAGATCACTATGGTCTTTATCAGTGCAATCCGGATAACTGGTTGATGCAATCTCCTTTACTGAAAAAAAATATAGATTATAATAACGGGGACGTAACCATCGCTAGCAATGTAAAAAACCTAGCGCTTAGAGCCACACAAAGGCTGACGCCCATCCATCTATATACGGCTGATGGGGGTATTAATGTAGGACATGACTACAATAAACAGGAAGAATTAAATCTTAAGCTTCACTTTGGTCAAGCCCTTACGGGTTTGTTGAGTCTTAGCAAAGGCGGAAACATGATACTCAAACACTATACCTTAAATCATGCATTTACTCTTTCTTTAATATGTGTATTTTCTCACTTTTTTGAGGAACTATACATTACCAAACCTACCTCCTCTCGGCCCACAAACTCTGAAACCTATATTGTGGGTAAAAACAGATTACGCTTATTTACCCCCAAGGAAGAACAAGTCCTTCTAAAACGGCTAGAATTTTTTAATGATACGCCCCTCGTAGACCTAAGTCTTTACCAAAATTTACTTGAAAGCGTTTACTTTGCCGTAGAAACAATACATCTAAAACAACAAATAGAATTTCTAAACTTCGGAATGAAATGTTATCGACATTTTTATAACAAGATTAAACTACTTAACGATTATTTAGCTCCGAAAAAAAAGATTTTTCAGGATAGGTGGCGTGTGCTTAATAAGCTTTATGTTCTTGAAAAAAAGCATAAACTTAAGCTTTGTGCCTCCTAGGGATCTGTTGCTTAATTTAACAGATGCAATCTTAACAGATGTAAACTAAAAAGTGTGTTCATACAAGGATTGTATTTATGAATATTTATTAACATATAAGGTTGTGATGTAACACTGTATAACCTATATAACTACACTATGAAGCACGGCGTATAATAATTTATATTGAACACGATGTTGACTCATTTATTTGCAAACAAATATTTGTTTGCAAGACGTTTGCATGCATTTACTAATATGTTGTTGACTAGTTTATTTGCAAACTAGATGTTTGATTGCAAACTAGATGTTTGCACGTATTTATTTGAACTAATATACACTCCTTGTTTTATTTGTTATATACACAGCATACATAAGTGTATATTGTTTACACTTATGTTTATAACTCGACGTAATAACATTTTACACGCTTTTTTTTTGCAAATCTTAATAATATTGTATGATAAATCAAACAATGTCTTATATATGTGGTTTATTATTTTAGGCGCCGCAAGATGTACTCCATTCTCATTGCATGCTTGGTGTTATTACTCTGTCTAGTTATATATGTCGGTCATCGTGCCGATCATGCACGAAAATATTTAGAAGGAATGTGGCATGGAGATCCGGTTTTTCTAAAACAGTCGGGGCTACAATCCTTTTATCTCTACATACAACCTGACCATACATGTTTTTTTAGCATTGTGAATAAAAATGGTGAAAAGCTGATGGAAACCAAAATACCTTGTACGATAACAAATAAAATATATATGTTTTTTAAACCTATTTTTGAATTTCATGTTGTGATGGAAGACATACATAGCTACTTCCCTAAGCAGTTTAACTTTCTGTTAGATAGTACAGAAGGTAAACTTATTTTAGAAAACAATCACGTTATTTATGCTGTATTGTATAAGGATAATTTCGCCACCGCACTAGGAAAAACGGTTGAAAAATATATAACACAAAATTAATCATGTTTTCTAACAAAAAGTACATCGGTCTTATCAATAAGAAGGAGGGTTTGAAAAAAAAAATAGATGATTATAGTATATTAATAATTGGAATATTAATTGGAACTAACATCTTAAGCCTTATTATAAATATAATAGGAGAGATTAATAAACCAATATGTTACCAAAATGATGATAAGATATTTTATTGCCCTAAAGATTGGGTTGGATATAATAATGTTTGTTATTATTTTGGCAATGAAGAAAAAAATTATAATAATGCAAGTAATTATTGTAAGCAATTAAATAGTACGCTTACTAATAATAATACTATTTTAGTAAATCTTACTAAAACATTAAATCTTACTAAAACATATAATCACGAATCTAATTATTGGGTTAATTATTCTTTAATTAAAAATGAGTCAGTACTATTACGTGATAGTGGATATTACAAAAAACAAAAACATGTAAGTTTATTATATATTTGTAGTAAATAATATTTTTAATTACTTAAAATTTTTATATATAAGTTTTTGATACTATATTATAAAACATATGTTCATAAAATGATAATACTTATTTTTTTAATATTTTCTAACATAGTTTTAAGTATTGATTATTGGGTTAGTTTTAATAAAACAATAATTTTAGATAGTAATATTACTAATGATAATAATGATATAAATGGAGTATCATGGAATTTTTTTAATAATTCTTTTAATACACTAGCTACATGTGGAAAAGCAGGTAACTTTTGTGAATGTTCTAATTATAGTACATCAATATATAATATAACAAATAATTGTAGCTTAACTATTTTTCCTCATAATGATGTATTTGATACAACATATCAAGTAGTATGGAATCAAATAATTAATTATACAATAAAATTATTAACACCTGCTACTCCCCCAAATATCACATATAATTGTACTAATTTTTTAATAACATGTAAAAAAAATAATGGAACAAACACTAATATATATTTAAATATAAATGATACTTTTGTTAAATATACTAATGAAAGTATACTTGAATATAACTGGAATAATAGTAACATTAACAATTTTACAGCTACATGTATAATTAATAATACAATTAGTACATCTAATGAAACAACACTTATAAATTGTACTTATTTAACATTGTCATCTAACTATTTTTATACTTTTTTTAAATTATATTATATTCCATTAAGCATCATAATTGGGATAACAATAAGTATTCTTCTTATATCCATCATAACTTTTTTATCTTTACGAAAAAGAAAAAAACATGTTGAAGAAATAGAAAGTCCACCACCTGAATCTAATGAAGAAGAACAATGTCAGCATGATGACACCACTTCCATACATGAACCATCTCCCAGAGAACCATTACTTCCTAAGCCTTACAGTCGTTATCAGTATAATACACCTATTTACTACATGCGTCCCTCAACACAACCACTCAACCCATTTCCCTTACCTAAACCGTGTCCTCCACCCAAACCATGTCCGCCACCCAAACCATGTCCTCCACCTAAACCATGTCCTTCAGCTGAATCCTATTCTCCACCCAAACCACTACCTAGTATCCCGCTACTACCCAATATCCCGCCATTATCTACCCAAAATATTTCGCTTATTCACGTAGATAGAATTATTTAATATGTACTATATATTAATTATTTAACCTTTCAAGCTGGTCTTCATTTAAATTTAAAATCCACTAATAAAATGTATTTTCTAGTAGCAGATCATCGAGAACATCATGTGATTCCTTTTCTTAAAACCGATTTCCATCACATGCATCAAAATCCTATACAAAAAAATCAAGCTCTCCTAGAAATCAAACAGCTTTTTACTGGAGATTATCTCATCTGCAAAAGCCCTTCTACCATTCTGGCCTGTATTGAACGAAAAACCTACAAAGACTTTGCGGCTTCTTTGAAAGATGGACGTTATAAAAATCGCCAAAAAATGCTGTCGCTGCGAGAACAAACCAACTGTCAACTTTATTTTTTTGTAGAAGGCCCGGCATTTCCTAACCCTCAAAAAAAAATTAATCACGTTGCCTATGCAAGCATTATTACTGCTATGACGCATCTTATGGTTAGAGATCATATTTTTGTCATTCAAACGAAAAATGAGGCCCACAGTTCCCAAAAGCTTGTGCAGCTTTTTTATGCCTTTTCTAAGGAAATGGTGTGCGTCGTTCCCACCTCCCTCACCCCCACGGATGAAGAGCTATGCATCAAGCTATGGTCTTCTCTTTCTGGTATTTCAGGCGTGATAGGTAAAATCTTGGCAAACACTTGTTCCGTAGCTCATTTGGTTCATGGAAAGCTTTCATCGCAGAATATTGATCAGTTAAAAACTCCCTCCAACCGACCATTCCCCAAAAAAGTAAAACGTATGCTTATAAGCATTAGCAAAGGAAATAAGGAGTTAGAAATAAAATTGCTCTCGGGGGTTCCCAATATCGGGAAAAAATTAGCTGCCGAAATTTTAAAAGATCATGCGCTTCTTTTTTTTCTAAATCAGCCCGTAGAATGCTTGGCAAATATACAAATCGTTCAAAAAACCCGTACGATTAAGTTGGGAATGAAGCGAGCCGAAGCGATTCATTATTTTTTAAACTGGTGTGGCTCTGCCCATGTAACCGATGATAGCCAAAATATCACAGAGGCGTCGCGGTCCACAATGCAGGTCGCGACGCAGTCCGCCGCAATACAGCCCGCTGCAACGCAGCCATTGCACGAAGTATCAGATGATGCATCATCAGATGCTTCATCACCCGTAGGGTATCAAACATTATCTAAAGAAATGTTATTGAACACAGCCTGATGTTAATAATTCACTACATCTAAAGAAATGTTAACCTCGATACTAAAAAGTCATTGAACACAACTACTGGGGCGCTAAGTTGTCCAACACATCTAAAGAAATGTCAACATCCTCGATGCTAAAAGGGTCATCGAGCCGGTCAATAATGTCTTCCCCAAAAAGTCCGGGAGAACTGTAGGCCGAGATGTCGTCCATGGAGCTATCTTCCCCAGAGCACACAAAGTCCTCTCCAAAAATCATAAAGTTAAATGCACCGGGCTTACTTAACAGCTTTTCGCTTTGAATAATAGTGTTGAGTTCTGTCAGCGCAAACTCTCTCACAATATTCACAACCCAGGAGGGCTCTTTAATTTCATACAGCGTTAAGAAACTTATACATAAAAATTCTATAGAGTAAAGCAAGGCGCTGGCAGGATCTGTTACCCGTAGGTGTTTAAATGTAGTGTGATATTCATTCACAACGTTAGGCAGCACCTTTTCCAAATCCTCCTTTTCCTCGTACGACAGGTGCTTTACAAGCCTTTCAACATGTATAGGAGGCTTGTTAAATGTACTAACGTGCCGCAAACAGTTATAATTATATAAGAAAATACGTACGGCAGAGTCGACCGCCATGAGCCTTGGATCACCCATTGAGGTAGGTGGTGGCGGGGCACCCTGGCCTTCCCTGATGTCTGCGTAGGAGCGCCCCTCCATGGCCCCTATGGCCTCTATCACAGCAGGACTGATATCCAAAATCTTGGCCGTCTTGATTATTTTTCCGTAATCGAAAGTCCATGGCTCCTGTGGAGGCTTGGGTTGTGTTTCGGTGGAGGGCGTGGTCATATCTTTCTTTATTTGAATAGAACGGATCGACATCTTTTCCTTATCGTACTGGTCTTTATAATTATTATAATAGTCATGAACTAATTCGGGTTGAGAAAGATGATCGTATATAATATAGGTAAAAAGTCCGCACTTGACACATTTTTTATCCTGGAAGTCGTGTAATCCTCCCTTGGGGCAGCGTGACTCGTAGAAGGCATAAAAGGTGTTAAATTCTAAGCTCGCCTTTAGGGCTGTTTGGACCTTTTTTATGTTTAATTGCCCCACCTCATGTTGTAGCACGTGGCATACAGAACAGCGTAGATCGGCAAGTGCATAATGGTTGTCAATTTTTTTTATGACGTCTTTGCGTGTTACTTCAATCTCGGCGGGTTTCTGCGAACTGTCTACGGCCTTGTAAACGTAAATGGTCCACTTATGAGGAAGCCCCCTTTCATCGTATAGGGTTGAAATGGGAAGCCTTTTATACTCAAACAGCCGAGTCCGTTGGTCGGCTCTTCCTGTGTTAGGATCAAATATGTTATAAAATCCTTGCTGAGCAAGCAGGGCCTTTTGCTCGCCATAAGCATTTTCGTACGTTTTGAATTCTGCAAGTTCGGAGTTAAAATTAGGTGCATTTTGTAAATACTTAAGAAATAATTCATAGGCTCTAAGGTAAATGAGAGTTGAGGTTTTTTCCTCATCCCGTCCTCCCCACCACACCCGCAGGCTTTCTTCTTGAAAATAGATGTCATTCAGACGCGTCAACTGCGTAAAATCAGGCCGATATTTAGAGGTATAAATTTTATCATAAAATTCTTTTTGCGATAATAGCTCGGCCGGGGTACGTCCTATCACGGTTTTAAACTCATATTCAGCCTCCTTGGGAGTCCGTGGTTTGTGCATAGGGATGCTGCCGTCAATACGGGCCACTGTGGCAGCATAATCATACATGGGGTCCAGCAGAATCTCTGTCAAAAGTACCTTGGTGTCGTCCTGCACGCTAAGCCCTTGTAGCCCATTTTGGTGGATAATTTTTTTGAAAGCCTCCCGAAAATTATTAGCAATCCACTGATCCGTAATCTCAGATAGCTGATTTATTATACCGCTATATTGCTGCATCATTTTCTCCAAAAGAAAGGTCACGTATGCATTCAAAGAGCTATCCGCCTTCATTCCATGAATGGTAATCGTAAGAAATTCTTTATTTTTTTGCGAGCTATAAATGAGATTCAAAATATAGGCATAGATGTAGATCACAGCATACAGCTGCGTTAAAGGATCGTAATCCTCTTCCTTTTTAATATTTTCGATGCTATACACGAGCGGCAGGCAGACATTTACGGCTATATTGGCAAACTGTTTCACGTCTACAAGCTTTCCAAAGTGGATAAACGTGCAGGCCTTCATGGTTTCCTGCCAAATAAAAACACGGAGCTTACTATTAAGATCGCCGATGATGCCCACATCTGCCGTACGATCCTCTTGAATAAAATGGGCCAGCTCTTCGCCACAAATTTTGCAAAAGTAGGAGTAAATAAGCCCCTGGTTGTTTTCTTTCTCCTTGTTTATTCCTGAAAATTTCATTAGCTTGGTTCGCATGGTGTCGTAGGACGCTTCTGCCGCTTGAAGCTGTATAAGCATGTCCACATGGGGACAAAGCAGCTTAAACCCGCAGGCTTTGCATAGATTCCAATTGGTGGTATTGTTTTTTTCCTTGTAGAGTACACGAATACTTTCTAATACTTTTAATAACTCCGCGTATTGAAGACCCGAACGCAACTGTTTTACCAGCTTGAGATGAGCACATGCATTTTTTTCTTGGAGTTCCCACTGTTTTTTAATGTTTAGGTATTCTGTTGTAATAAGTTCTGCCTCCTGTTTCCCACAGGCTTTAATGACTTCTTGAAGGATGCTGTTAGGGTCATCCACTTTACCCTCCATTGTAAGAATTTCACGTATAGCATCCGACTGCACCCTACCTATTTTTTCTTCCATAATTTTAAAATACTGTCTCGCCTGGGTAATGACCTCTGTGAGCTTCATGTCCACCTGCTGCAGAATCATTTGCTCCTTTTCACGCTGTTCAGCATGTTGTAAAAACTTTTGTTCTACAGGGTTCCAAAGCACCTCCAAATAGCCTGCTCTATATAGGTCATAAAGCAAGGGCATGTATCCCGATGTAAAAACCGGGGACACCGAGTACATCGTAGACAACTCTTTTAAAAAAAATATCACGCGCTTAATGTTCTCCTCCGGTTCAATCTCCTCGGTTTCAACGATATTAGATATATGACTGCCCTGATCCTCACGGTCTAGCTTTCGGTGTACCATCTCCTCTGCTAGCCGATTAATGAGCCAGCTATGCCCGCCGCTCCGCAAAAACTTATAAAGTTCGATATACTGGTGCGTAAACTGGATGATGTTTTCCTTGGTGGTTACGACAACCCCTTCTCCGTTTTTTTTCCAGGTTTCTTGATCCACGCATTTCATAAATACTCGAATAAAATTGGTCAAATTGGCTCCTGAGGCGACGTAGCCCAAGGTTTCAGGCGAGAAGGAGCCTATCTCAGCCATACGCATAAAACACTGCGGGGAAAAAGTTTTTAGCCGCAACTTAAGTCCATAGATTTCAATGGGGGCTTCTGCGGGAACGGCCAGGTGCGTCCCATTAATTAAAAAAATTTCTTTGCGTGTGCTAGGGCGAACACGTAATTCCTTTTTTTTTTCACTCACGATGGGGACCACATCGGGGTCTACCAGCAGTTGACGTATGTAGGCCTCTATGGGCATGGATAGATCGGGCAGCTTTGACTGCTCGGCGCGAACATGGTTCACAAAATCTTTTAGAGTGAAAAGAAAGTCTATTAAACGTATGTTTTTTATATCATTAGACCCTTTAAGGGTAGAGTAGATTTCATCCACTAGTGCCTCGATTTCCTCATTATTGAGCGATAAGATATCTGTGCCACGGTGGACTATTTGCGCGATCGTAATTACTTCCTCCATTAGATAGAAACTGAATATTATATTTAAAATAAATACAAAATGTCAAATGAAAGTTTTCCCGAAACGTTGGAAAACTTACTTTCAATGTTACAGACCAAACAGCAAAACGCAATTCAGTCAGAGGTGATTGAATGGCTGCACAGCTTTTGTGAAACCTTTCACTTAAAAATACACTGCCATAAACAGTTTATTCCTAGCGGGGAAAAAAAACGAGCTAAAATACCCGCTCAAGAAACACAGGGAAACACGCAGCCCTCCCACCATGTGTACCGGGTTGTTCTCTCCAGAGCACAGCCAGTCAAAGCACAGGAATCTCTGCTAACAACCATGTGCAACGGACTGGTGCTAGATGCAAACACATGGACATGCCTAGCCATTCCTCCGCCTGCGCCCTTTCAACAGGCGACCCGCCAGGTCCAACACTTTTACCGTAACAATTTCTACGAAGTGGTTCCCATCCAGGATGGCACCCTTCTCACAATCTACCACTGGGATGACCCTGAATATGGCCCCTCCTGGTGCCTAGCAAGTACCCACGGATATGATGTGAGTAACTACTGTTGGATAGGCGACAAAACCTTCGCCGAGCTTGTATACGAATTGCTGCAGCAGCACTCTACCTGCGACGTCACCCTGGAAAAAAATAAAACGCGGGGAACGCGTCTTTTCTTTGATAACTTAAATCCCGATTACTGCTATACGATTGGAATCCGGCACCATAATTTACAGCCGCTCATCTATGACCCTCAAAATATTTGGGCGATTCAATCTACAAACCTAAAAACGCTTAAAACGGTATATCCAGAATACTACGGCTATATAGGCATTCCAGGAATTCAGAGTCAAGTTCCTGAGCTTCCCCAGTATGATTTACCTTATCTAATACGATCTTATAAAACTGCTATGAATCAAGCCAAAAATGCTATAAAAAATGGCAAAAAAGACAAGGGATACTTTAATTATGGCTATTTACTCATTTCGCGAGCGCCTGCCATTACTAAAAGTACTTCTAATGTTTTGTTAAAATCGCCTCTGCTGGTATTTTTACAAAAAAGTGTGTACCAGAAAAAACACAATATCTCTAACAGCCAGCGACTAGAATTTATTATACTGCAAAACTACTTGATGCAGCATTTTCGAGATCATTTCATTGCTCTATTTCCGCAGTACATATCCTATTATACGAAATACCAAAACATGTTGAATATGATTATCCATAGTATTGCAACTAAAGATAAAGATCATCCCTTTGCAGGAGCCGTGGTAAAAAAAGTGTTGGAAGATATTGAAAACGCCGAAAACATTATTGATCATACAACCATTCAAAACTATGCCCATCAAAGCAAGTACGCCATGCTTTACTTGTCAATTATTTCCCATTTTTAATCTAATACGGCCAAAGCCGCGGGTTTTTTAATAAACTAACATTTAAAAAAACTGTTTTATTAAAAATTATAATACTTTTATTATATATGGAACATCCATCTACAAACTATACTCCCGAACAGCAACACGAAAAATTAAAACATTATGTTTTAATCCCTAAACACCTTTGGTCTTATATTAAATACGGAACGCATGTCCGGTACTACACCACACAAAATGTTTTCCGAGTCGGTGGCTTTGTGCTTCAAAATCCCTACGAAGCCGTTATAAAAAATGAGGTAAAAACAGCAATAAGACTGCAAAATAGTTTTAACACAAAAGCGAAAGGGCATGTAACGTGGGCCGTCCCATATGATAATATTAGCAAGCTATATGCCAAACCAGATGCAATTATGCTTACCATACAAGAAAATGTTGAAAAAGCTCTTCATGCTTTAAACCAAAACGTACTGACGCTCGCATCAAAAATACGTTAAATATAATTTTTGTAGAGGATAAAAAGCTATTTTAGCTAAAAAATAATTCATATACGTTTATGCAGAGGAAGAACGGTGGCTTTCAAATTCAGATTGCATCCACGTAGACCGTAGCGTTTTTTTTGCTTCTGGTTTATATCGTAAACCGTAATAAACATCATCATTTGTATCCGTTGGATCTTTTTCCCACTCCGGATAAAAAATCGGTTTTCTTTTTTTTTGGTCGTTTTTTGCAGTAAGCTGTAAATTAAGGGAATATAGCTTATCGAAAAGTTGTTCCTGATCCATATAAATAGCAGCATATATTAAAAAAAAATAAAAAAAGACGCTTCAACGAGTCAGTACCACTGCTTGCCAACGATTTACGTTGGTTGGTGCATTATGGTGATATAGTAATGAGTGCCTGCACAAGTGCTTGCACAAGTGCCTGCACAAGTGCTTGCACAAGTGCTTGCACAAGTGCTTACACAAGTGCTTGCACAAGTGCCTGTACACATTACTGCATCGCCAAAGCACCTGCAATGCCTACTTCCTCAACAGAGTACGATAACTAAATGCTTTTAAGCACCGCTTGCGTCGATGTGTCCTTCGGGGCAATCGGGTTCAATTGGATCCAATATTATTAGTCATAATTACCTAATACTTATTCAATTTTATCTTTTTTACCTTGTAAGATTTAAACAGCGTTTTAGCTTGTTTAAAGCAACGTTTAAAACAAGCTAAAATGCTGTTTAAAACAACGTTTTAAACAAGTTAAAACAAATAAGCTTATAAATATACCATGACAAAATTAGCCCAATGGATGTTTGAGCAGTATGTCAAAGATTTAAACCTAAAAAATCGAGGGTCCCCCTCGTTCCGCAAATGGCTCACATTGCAACCCTCACTGCTGCGCTATTCGGGTGTGATGCGTGCTAACGCCTTTGACATCCTAAAATATGGCTATCCTATGCAGCAGTCAGGTTATACGGTTGCTACGCTTGAAATCCACTTTAAAAATATTAGGTCTTCCTTTGCCAACATTTACTGGAACCGTGATAGCGAGGAGCCTGAGTACGTCTGCTGTTGTGCCACCTATCAATCGCACGATGGCGAATACCGGTATCGATTTGTTTGGTACCAACCCTTCATAGAGGCTTATAATGCCATAGAGGCGGCCCTGGATCCCCTGGAAACCATTATCCTGAACCTCATTGCGGCACGAGATCTAGACTTCGTTGTTCACATATTTCCTTATAATAAGGGCCATGAAGACTATTTGGCCTCCACGCAACTTATTCTCAAAATCTTTATTGCGACGCTTTTAATGGACATTTTAAGAATTAAAGACAACACGTTGGACGTTCACTTAAATTCCGACTATATTATTGTGATGGAGCGGCTTTGGCCTCACATAAAGGATGCCATAGAACACTTTTTTGAAGCCCATAAGGACTTACTAGGGTACTTAATTGCCTTTCGCAATGGGGGGAACTTTGCAGGAAGTCTTAGACCCTCCTGTGGGCAAAAGATTGTTCCCCTAACGATTCGAGAGGTCCTACAAATGAATGATATTAATTTAGCCGTATGGCGGGAGGTGTTTATTATGCAGGAATGTTCCGACTTAGTCATCAATGGGATAGCGCCCTGTTTCCCCATTTTTAACACGTGGACGTATTTGCAAGGTATTAACCAGATTTTTTTTGAAAACACGTCTTTGCAGGAGAAATTTAAAAAAGATTTTATTGCCCGAGAGCTTTCCAAAGAAATTATCAAGGGCCAAAAAACGTTGAATGACAAGGAGTTTAAAAAGTTAAGCCTACATCAAATCCAGTACATGGAATCCTTTCTACTTATGTCGGATGTTGCCATTATGATTACCACAGAGTATGTTGGCTATACCCTTCAATCCCTGCCGGGTATTATTTCGCGATCCAGCTATTTATCCCCCATCGTGAAAAACATTTTGATGGACAAAGACTCTTTTATGTCCCTACTATTTGACCTATGCTATGGCGCCTACGTGTTGCATAAAAAAGAAAATGTGATTCACGCGGATTTGCACCTGAATAACATGACCTACTACCATTTCAACCCAACCAGTTTTACAGATCGCAACAAACCAGGAAAATACACCTTAAAGGTCAAGAATCCTGTGATTGCCTTTATAACCGGGCCCAAAGTCGAAACCGAAACGTACGTGTTCAAGCACATAGATGGGTTCGGCTGCATCATTGACTTTAGCAGAGCCATTATGGGGCCAAACCATGCAATCAAGCTTGAGCGGCAGTACGGCCTCGCTTTTGTAAACACCTTTTACCGCAATCAAAGTGAGCATATTTTAAAGGTATTACGGTACTATTTTCCTGAAATGCTAACCAATCGCGAAAACGAAATACAGGGGGTGATTTTATCAAACTTTAATTTCTTTTTCAATAGCATTACTGCCATTGATTTTTACGCCATTGCTAGAAACCTACGTAGTATGCTTTCTTTGGACTATTTACACACCTCTGAGGTGAAACGAAACGTAGAAATTTCGCAAACATTTTTGGATACATGTCAATTTTTGGAGGAAAAGGCCGTGGAATTTTTGTTTAAAAATCTTCATACTGTCTTATCTGGCAAGCCGGTCGAAAAAACGGCCGGGGATGTGCTTTTACCCATCGTATTTAAAAAATTTTTATACCCAAATATTCCTAAAAATATATTACGGTCTTTTACCGTAATAGATGTATACAATTATAATAATATAAAGCGTTATTCTGGGAAAGCTATACAAACGTTTCCACCCTGGGCTCAAACCAAAGAAATCTTGACGCACGCCGAGGGTCGTACATTTGAAGATATTTTTCCTAGAGGAGAATTAGTTTTTAAAAAGGCTTACGCAGAAAACAACCATTTGGACAAAATTTTACAGCGTATTCGTGAGCAGCTTGCTAATGAAAATTTGTAAGGCTTGCAGTTCTTGTATGGTCAGAACCTATGTCGATGGAAACATTATTTTTCGCTGCAGCTGCGGCGAAAGCGTTCAAGGGGATAGTCAGAACTTGCTCGTCTCTAGCAAGGTGTACCACACCGGGGAAATGGAAGATAAGTACAAGATTTTTATTAAAAATGCACCCTTTGACCCCACGAATTGCCAAATAAAAAAGGATTGCCCAAATTGTCATTTAGACTATTTGACACAAATCTGTATTGGAAGCCAAAAAATCATTATATTGGTGTGCCGCTGTGGCTATATGAGCAACAGAGGATAAACCATATCATCCCACCGAATTATGACATTCCTTTAAAACCGTCCGCCTAAATAGTTTTCACACCTTTGGTGGCAGACTATTTTATAAAAAGTAATGTTGGTTCATGAAGATAAAGTGTGCCAAAGAAACTTTTATAAACAAATGATTAATGTAGGTGCTAGTCGTGTGTACTTAAACAGGGTATTCTATAGCCAAGTATTTTCTATAGCCAAGTATTTTCTATAGCCAGTATTAGTCAAGTATTTAGATGTCAGGGTATTTTTATAGCCAGTATTTTTCTATATGTACAAACTATTCCAGTAAACATATGTGTGTTCTTTATTGAGCAGCATCATGGCATTAACAAGTTTATTAAACTGCTCTAATGGGCATTAAATGACAACTCGGTGCTTAGCAAAAGTGCCTATACCTTTTAACAATTAGGGCCGGGAGGCATTCCCAGCTTTTTTCTATAATCAGCCATACAGTACCCCTGAGCCTCATACACGGGAATAAGGTCCTTCCATTCCTTGTTGGGATCGGCGGGCCAGCTCTCAAATGAGGTGTGAATGTAAGGGTCCTGTTCTTTTTCCTTAATGAAGCGTTTAATCTCCATTTGATGTTGTTTACTTTTTTGTTTGCGGCGGAGCGTGTTCCGCACCAATACGTAAAAAATACCAAGAATCACACATAAAAGAATTATTAAAAAAAATATCATCATCGCGGGGTTTAAAAAACGATCCCATGCAACAGGAATCGTTCTTAAAACCTTGTCTGGCAGGGCTGTAAACATGAAGTCTCCTCCTATAATCGGGGTGGGACTGTAGCCTAACAGTTCAAGGTCCTGTCGTTCTAGATACTTATTGGCGAACTGCCCACCCTTTGCCCCCGTTTTTTTATTAATCAAGCAGCGCTGCATTTTCCACCATTCTAAATCTTCAGGAGAAAGCTCAATGCCATATATCAACTTTAACGTTATTGCATCTTTTTCAATATCCTTATCAATTTGGCTGAGCTTTTGAGCTTTAAGCGGGTCTAGTGTGTACTTCCATTTAAACTTAGTGTCCTGTAGTTTGGCTACATGAAATACGGAACATTTCGGCGGGGCCTTTGTGACGCCCTTACACTGCGGAAGTTTATCATTAGGACAGGCGCATAGATGAGACTGCGCCACAGCATCGCGAACTACATCGCAGACGGAGTACATTTTCCTCCTATGTTAAACAATAAATTTTTTTCATAGCTGAAATTTGTGGGCCTATCTTTTCCCTTGCCCGGATAATAATTATAAGGGAGTGTTGAAACATCTGGGAGAGAATTGCTTAAAAAATGGGTTTTTGGGAGGGGTAACTGCGACTGTTGTACGTCGTTGGCCAGGGAGATTCTATATGCCGGGCTAAAGGTGCAACGTTCCTGTGAACAACTTAGTACGCGCGTTGTTAATACAAATGGACTGGTATTAGCAAACCTCGTAAACTCTTCCGGACTTGTTTGTTTTTGTATGATGTTTAGCAGGGAGTCTGCCTTTTCGAGAATCCAAAGCGTCGCATTGTAGTAAAATAAAAATAGCGACTTATCGGCAGGCGTTGCAAAAGCGCCGTATAGAAAATAAAGCAGTAAGTACTGGGGAGACACCACAATAAGGTTATCTTGAATGATAGATATCGCTAGCTCTTTAAACATAGTGCTAAAAAAATGTATGTCGTTCGTCTTGAATATAGGGGGACTATAGTCCATGTAGGGCTCACATATCTCAGTCAGGTGAAGGCCCATTTCTTTTATGACTTCTTCCGGGTTGTACGTCGCTAACACCAGCGCGGGATAGGCTTTGGGCATATCCACGGTAAGTGTTATGTTTTTATCATTCTTATGGTAGGAGTAAGATGGTTGTGGAAATTCTGTTTTCCACTCCGGGACTTTGCAGGTAATTCTCAGCTCATTTAGAGTCTGGTACAGGAGGGCGTATGCCGCAAAGCCGTGTATGGCCACTTGTTTAAAGGGAATTGAAAACGTTTTACTTTCGTATGTCGACTTCACAGGAACAACGGGAATGGGGTAATATTTTTCTATGAGGTTATACCGCTGCAAATCCTTTTTAAACCTGCTAAAAACATCTTCCCTTGGTGGGTTATCAAAAGGAAAGCAAAATGCTAGGTGTAGCCCGGCCCGCTGGTAATCGGGGTGAATGATTTTAAGGTTTTTATACGTTAATGTGGGTATGGTGTTAAAGATATTGGGGGGCATATATGAAAGATCAGCAACCCACACAAAGTCCGTGCGCACCCGCATGGTCTGCACATGGATGGCGCGCACCGTGCCCACCTGCTTGAAGCCCTTTTCATACAAAATGTCAGCAAGTTCGTAGGCGTCCTCAACGTGGTTGGGGGAAAACATATCAAAGTCGGGTCTTTCTCCCTCGGGATAAATTGAGCTGCCTTTAAGATGCAGGGCATAATCAATGGCAATCCCCCCGTACAAAATAAGCTTTTTCTTTATGATAAATTCGCGGACCACCTCCAAAGCCGCCTCAATCTCCACGGCATTTGCCTCACGTTTTTGAGCAATGAGCCGGTACTTAGAAACATTAAAATCAGTCTTTAGTAAAGACGTCATAAATAGTGTTTAATATATATTAAAGGTTTGAATAAAATACTAAATAGTAAAAATGGATGCCCTATTAAAGGAAATAGAAAAGTTATCGCAGCCATCCTTGCAGAAAGAAAACAATGATGTATGCGATCTCTGTTTTATGCAAATGAAAAAAATTTCTAACTATCAGCTTTTATGCGAAGAGTGCGGTCAGCTGAAGGACTGGTTTGAACCTGAATATAATGAAAAATTCACGGTATATTCTCGTCTAAAGATCGTGGGTGCCAATAGTTCCTATCACCAGCGCGATTTGGACAAGGCCAACTCAAGTGACTATAGCTCCTTGCAATTTCATCACATTTTAGAGGAGCTCAAATCCCTAAATGTTAAGTATATGGATGCGGGGCAAAAGCCCTTTCCTATTCAGGTGTTAAAAGAAACTGCTCACAGTTATAACCAAGTACAACAACATCGGGTCATACGCAGCATTACAAAGCTTCAGATCTTAGCCAGTATTCTACGTAGCATTTGTTTAAAATTAAACATTGCTTGTACGGTGGCAGACGCCGCGAGGTTTACTCAACTTAATACCAAAGGGATCTCAAGGGGCATGGATCTTCTGCGCTCCCTATTTGTAGACAATAAAATTACTTTAAACGTTGATTTAAACCCTATAGACAGCTTTATTAATAGTACCTACAGTGCCTTACAAATTAAACAAATCCACCAAGAACTGCAGGAGGAAAATGTTTATAATTTAAAAGAAATTGTTAAGAGCTTTATATTATACGCGGATGAGAAGAACATCGGCGTCGATCTTAACAGGAGAACCGTTGTGATTGCTACGATGTATAATGTTTTACGCCGTGCCTACTACCCCATAGAAATTGATACGGTGGTGTATCAATGTAAAATACGAAAAAATACAATTACACGTGCTCTTAAAATGTATGAGGATTACTACTCCCACTTTAAGTCTCTTTATGAGCAGTATCATTTAAACGCGGCAAAAAAATTAATTTAAACTAAACGTTTAAACTAAATGTTTAAACTAAACGTTAAAACTAAACATTTCGACTAAAGTTTAAAACCTAGTCTAACAGCGGGATGCCCATTTCCCTGGGGTTCCATATTTCAACAATTTTTTGACCTTCGGGTGTTACCTTGATGCAGCGCATGACGAGCAGTGGAATTTTCCTATTAAAGAGTTCTTGCTTAGCTATATCAATAGGACTGCTATATTTTTTTTTAAGCATTGTAGATCCATTAATTGCCAATTGTTGCGCTCTAACGGCGACCAACCTTGTGGCCTCAAAGGTGGTTAAAACGTTGGAGGTAATGCGCTCGTTATCGGGTATAATGACCAATGTTTGCGACGAGGCCTGCACAAAGCCCTCGCAGATGGACGGAGACTCCACGATCTCGTCCTTGTCCTCGGACTCCTCCTCACTGTCGACGAGGTTCTCCTCTTCCGTTTCCACATATTCCTCCACGAGGTCATCCATGATAAGATCCTCGTTGTCATTATCAGCCATATTACACTGTTATCAAATGTACTGTTTAATACGCAAATGGATTTACTACGTTTTAATTGTATGTCTTCATGTGCAGGCTCTAGTGGAAAGTAATTTTCTCACAATTTTTGGCACCGTTACACTTGTGCCCACAAAAACCCGCGATTTTTTTATTTTATATTACTTTTGGAAGTACGAGTTTAACCAGTCGCTTTCAAACCTTATGCGTCTATCTCGCCAAAAAACGCTCACAGCGGTGTTGGATATTACCTTTAAAAAAATAACATTAATTTTTACCACAGAGGGCGTATTGCGTATGGATTCTACGAATAAGCCAGGCGTGCCACTCGATATAGACCCCCAGTTCATTGACCTTGATAGTATTTTAATGGAACTGGATCATTAGGACCTCTCCCGCCCATTTAAATTTTTAGTTTCTACAATAATAAAATGCGCGAGGAATCATGGGAAGACCACGATACCATTCAGCTCACCGCTCAGCGCAAATACCTCGCCGAGGTGCAAGCTCTAGAGACCCTTTTGACTCGAGAGCTTTCAGTCTTTCTCACAGAGCCAGGCAGCAAAAAAACAAATATTATTAATAGAATCACAGGAAAAACCTACGCACTTCCCAGCACAGAGCTACTAAGACTCTACGAGCATCTCGAGCAATGTCGCAAGCAAGGCGCCCTCATGTATTTTTTGGAAAGACAGGGGACCTACTCGGGTCTCATGTTGGACTATGACCTTAAACTCAATACAAATGCTGTTCCCCCGCTGGAACCCCCCGCGCTATCACGGCTTTGCCATCGAATATTTGTGCATATAAAAAACAGCAGTGTGCTGCCTGAGGGCAGCCATAAAATCCACTTCTTTTTTACATTAAAACCTGAAGTGGTTCAGGGCAAATATGGGTTCCATGTGCTCATTCCTGGTCTCAAGCTGGCGGCTTCTACCAAAAAAAGCATTATAGGATCCCTACAGCACGATGCCACCGTACAAAAAATTCTACACGAGCAGGGCGTTACAAATCCTGAGTCCTGTCTGGACCCCCACTCCGCCTCCGTTCCCTCGCTCCTCTACGGCTCCTCCAAACTAAACCACAAGCCCTACCAACTGAAAACCGGCTTTGAGTTAGTCTTTGATAGCTCTGATCCCGACTACATTCCCATTCATCAAATAAAAAATTTAGAATCTTATAATTTAGTTTCTGAGTTGAGCCTTACGAATGAACAGGGAAGCCTTGTAAGACCTGTCTATTGCGCGGCAGACATTGCCGCTGAGAAGGAGGAAGAGATCCCGACCGAGGATCACTCGCTCTCCATATTAATGCTACATGATCCCGAAGCCCGGTATTTACATAAAATTTTAAATCTGCTTCCTCCGGAGTATTATGTAGAGTACCCCCTATGGAGCAACGTCGTATTCGCTTTGGCCAATACATCCGCTAACTATCGGCCCCTCGCCGAATGGTTTTCGCAAAAATGCCCTGAAAAATGGAATACGGGAGGAAAAGAGAAACTAGAAAAACTTTGGAATGATGCCTCGCACCACACTGAAAAGAAAATCACCAAGCGGTCCATTATGTACTGGGCCCACAAACATGCCCCCCAGCAATACAAAGAAATTGTAGAACAAGGCTACTTTTCCATTCTCGCTGAATATGTGTATAGCTATAACGGCATGCTTGAGCACTACATGATCGCCAAAGTCATCTATGCTATGATGGGCAACAAGTTTGTAGTGGACGTGGATTCAAACGGGAAGTACGTTTGGTTCGAATTTGTGCTACCGGGCCAGCCAATGAATCAGGGAGAAATATGGAAGTGGCGCAAGGAGGTAAACCCGGATGAGCTGCACATCTATATTTCCGAAAACTTTTCAAGGGTGATGGACCGAATCACGGAGCACATCAAATACCACCTCAGTCAACCCCATGAAAGCAATATTTTAAATTATTATAAAAAACTATTAAAAGCCTTTGAACGCTCTAAAAGTAAAATCTTTAATGACAGCTTTAAAAAGGGAGTTATCAGGCAAGCTGAGTTTTTATTTCGCCAAAGAAGCTTTATTCAAACTCTGGATACCAATCCCCACCTACTGGGGGTTGGCAACGGGGTTCTCTCCATTGAGACCATCCCGGCTAAGCTCATTAATCATTTTCACGAGCATCCCATTCATCAGTACACACACATATGTTATGTGCCCTTTAATCCCGAAAACCCCTGGACAAAACTATTATTGAATGCACTCCAAGACATCATCCCAGAACTTGATGCTAGGCTGTGGATCATGTTCTACCTAAGCACGGCCATATTTCGCGGCCTGAAGGAGGCTCTGATGCTTTTGTGGCTTGGAGGCGGCTGCAATGGAAAAACTTTTCTAATGCGACTTGTGGCCATGGTATTGGGCGATCACTATGCCTCCAAGCTCAACATCAGCCTTCTTACAAGCTGCAGAGAAACCGCGGAAAAACCCAACAGTGCCTTTATGCGGCTTAAGGGGCGGGGATATGGGTACTTTGAGGAAACCAACAAAAGCGAGGTTCTAAATACGTCGCGGCTGAAGGAAATGGTAAATCCGGGCGATGTCACCGCTCGAGAGCTTAATCAAAAACAGGAAAGCTTTCAGATGACGGCCACCATGGTCGCCGCGTCCAACTATAACTTCATCATTGACACGACGGACCACGGCACATGGAGAAGACTGCGGCATTATCGGTCAAAGGTGAAATTCTGCCATAACCCCGACCCCAGTAACCCCTACGAGAAAAAGGAAGATCCTCGCTTTATTCACGAGTACATCATGGATCCAGACTGCCAAAACGCATTCTTCAGCATACTCGTCTATTTTTGGGAGAAGCTACAGAAGGAATACAACGGGCAGATTAAAAAAGTGTTTTGTCCCACCATTGAGAGCGAAACGGAGGCGTACAGAAAGTCACAAGATACGCTACATAGGTTTATCACAGAAAGAGTCGTGGAGTCGCCCTCCGCAGAAACTGTGTACAACCTATCCGAGGTCGTGACGGCCTACGCGGAATGGTACAACACCAACATTAACGTAAAGCGCCATATTGCCCTCGAGCTATCCCAGGAGTTAGAAAACTCTGTGCTAGAAAAATACCTTCAGTGGTCTCCCAACAAAACGCGAATTCTAAAGGGTTGCCGTATTTTGCATAAATTTGAAACGCTGCAGCCCGGCGAATCCTACATTGGGGTGTCCACGGCCGGCACACTCCTAAACACACCCATATGCGAGCCAAAAAATAAATGGTGGGAATGGTCCCCTAATCCCTCTGCCCCTCCTGAGAAAGAAGCGTCTGCACCAACTCCTTAGGGAATATCCTTAGAAGCATGTCTTTCGGCAGAGCCATTACCGGTAGCAAAAAAGCAACATTGAGTATATTATATGCCTTAGCCTGCTCATAAGCGTCCTTTTTTTTCATGGTATTTTATGTTTTTAAATATTTTTAATTATTTTTTAAATACGATGAACAGTTCGTGCTCCGAAGGCTGTTTACTAAAAATCGGTGTGAATCCGCATTCTTTAAATATGGTTTCCCATTCGGGGATGGTATGGAAATCCATGTCTCTACGAATAGTATGGTGCCCAAGTGCGTCCTGCAGGCTGTGAAGCCAGAAGGCCTCCTGACCTTGATGAAGGTCGTACATGATAAGAAAACCATCAGGTTTCAACAGATGGTAAAGCTTGTTAAAATCGTTTATCGTAAGATGATGCGCCGCCATAGGTAACCCTATGAGCTCCACAGAGTTTTCATGCTGGACATCGTCCATATCGGTATAAAACGTTTCACAGTAAATGAGACGCTTAAACGAGTATCGATGACAAACATTTATTTCCAAGTAGGTTTGCACTACGTTTTTAGGTATATCGGGAATCATGTTGATTAAGGTTGTTTCGGGAAACTTAATCATCTGACTAGGCTTCATTTTCAACTCTTTAAAGGATTTCCCGGAGAAGTGAAAATGGGTCTTTACGTATTTATGTAAAAATACCTGAATGGGCAGAGGGGGCTCCTCCTCTTCGTTCTCGACGCCTCCCAAAATATTTGGAATTTCCTGACGTGGCAAAAGAAAGTTTATGTCCACGTTTACGAATCCATCGAGGACGGACACAAAGCTTGGCTCTAATCTCCATTCCATATACTGTTTAGAAACGGGAGATAGCATAATCCTAGGCGTCACAATGCACGAAGGGTTTTTAATCACCGCATCGTGGTAAGAAAAGTGTATTCCATTTCTTCCAGTATAAAGAAGCCTATGTTCGTCGTAGCAGAAACAATTAAGGCGGTATGCCTCATACATACACTGTTTCAAAGTACAAACACGTTTTAAAAAGGTTTCTGCATTGGCGGAGGCCAAGCGGTTTTGCCATTGGTGGAAGGGGTTCAATCCTACAATGGCCAGCTCGTTTAAAATATCTTCGCGGCGCGCTAAAATCTGCACCATAGAAGAATACTTTAGCATTTTTTTTTCGCACCATTCGCGAAGATGTTTAGCTACATTATTAACCTTATTATTGATAAAGTATACGATGGCATGTTGGAAGCCTTCAAAAATAAAGAGCCCCTCCAAAAGATCATCTGCCAATAGAAGATGGATGTTGGTGTAAGCATTGTCAATATTTTGTAGAAACGGCGGAATGCCTGCCAAAACCGCTTCAGCAAGCATAGCTCCGTTCCGTTGTTTACTGTCCAATAGATTCGTAAGTTTTTTGTCCGCAACAGACACGACGGCTAGGATGGTTGCAATGTCAGAAATGGCGGCTTGCCAGAAATAACCCGAAAAGCACATGCGCGCTTCTTCTATAGATAAAAACGAAAAGCGAGAGGCAATGTCTCCGAGCTGCGTGAGTTGAAGACCTTTTTCTCCTCTGGTTAAAAGGCCTGCCACAATGGCCCGCTCAATGGCTGATGCCAGCGCATCCGTGGGGGGAGGATCCAGCATATCAATCTCCTCTGCCTTAAACACGCCTTCCTTATTTTTTTTAATCGTTTCTACGACAATGCTAAGAAAAATGGCCCCAGGGCCTTCCGTAATGATTTCAGGATACTGCTGCACTGGTATTTGCTCAAAGACGTGTTTTGTGTAAAGCGGGTAAAAGTGCCCAGGAAATACTCTCCCTACACGCCCCTTTCTTTGCTCGATACGGCTTTGAGCCGCGGGGCGCGTAATAAGCCCTCCCGCCCATTCGGGATAGTAGGTTTCAATGCTTCTGTTCCACCCGGGATCTATGACGTACTTCAGCGTTTCAATGGTAAGGCCCGTTTCCGCAACAACCGTGGAAACAATGACCCTTCTTAAAGGTTTTTCCACTTTAGCGGTTAAGGGATTTTTCACCCACAGATTCTTAATTTCCGCTTTCAGGCCAAGGTAGGCCTCATTTTCCTGCGCAATCGCCTCACTATCGATCGGCAAAATCAACATTAACGGCAGCTTTTCTTTGGCAAGGTCCATATTTGCATTATTCAGCAACATCGAAAGGAAGCGTATTTCAGCCATACCGGGCATGAAAATTAAAATATCTGCTTCCGTGGGACGATCATGAATGTTTTCTTTATGAATAGTGAGAGCCGTTTCGCAGGCGGTCTTAATGTAGTTGTTGGTGTTATACAGCGGCCAGTGGGTTTCCACACCGTACTGTCGTCCTTCCACCAAAATAATGTTTTCTTTTCCGATACCAAAATAGGTTGAGTATTTATGGGTATCAATGGTGGCGGAGGTTAAAATTACAAAGGGAATACGCAGCGCCCCTATGCTTCCTCTTTGCAACATGCGCTGAAGCATACTTTTAATATACATGAGCATAAGGTCGATGCCTAGGGCTCGCTCATGGGCCTCATCTATAATCATAAAGGCATAGCGGGAAGCTATCTCATCATCCGTCATTGTATGTAGCTGCGCCAACAGAACCCCCGCGGTTGCATAAATAAGGCCCCGATTGGGTTTTTCCGTCAGAGGCTTCGTTTGGTAGCCCACTGTTTGGCCTAATATCATGTCGGGGTAGTGGGTTGAGGCGCCGATGTCTTTGGCGAGGGTCACCGCGGTTAGGACTCTTGGCTGGGTACAAATAACCGAGCGTCCCAAGTATTTTTGGAAAGAATGCGTGTTTTCATTTCTCAGAATTCTGAACACGTGTACGGGTAAGGCCGTGGATTTTCCGGAACCAGTGCGTGACTTTATAATGAGCACCCGGTCTGCGAGGGAGGTTGGAATGGCCCCTCCAAACTCCGGGAGACGTTGTTTTATCCAAGTGATGATGTAATGAATAGGAACATCATTCTTGTGCTCAGCGGGCACGTTATAGAGATGACCAGGCTCCAATAAAGTCGGTTTTCCCATATTCTATTGTTTTAAGGATTGATTGTTCATAAATATTTTTATACTCTGACCAAGAAATTATTTTTTTATTAAGCCGGTTATTTACGTTGTTATGGAACGCGAAGGTCCAGTACTGAAAGTCCTCCGAGTTGTTTAATGTCAAGGGATTTTTTGTAAGATACGAAAAGGCGTGGTGCTGGCACCTGGTGCATGGCAGAGACTCGATAAAGTTCAGTATCCATTGGATGGCTTCATATTTTTCTTTCCAGCTAGGAGCGTCTGAAAAAAAGATAGCATATAGATGCAAGGATCGCCAGTATTTAGGTCCCCAATGCAACATTTATAACCTTTTGAAAAATCTCATTCCATATAGAGGTAAATATTTTTTTTCCATGGAGAATTTTTTTGCACTCTTGAAGGGATTGCGCCACATCGTCAAATGTTTTTTGTTTTCCATGTATTTTGGCGTAATTCCAGCCAGTATCTGTGTCATGGTCCTTAATGTCATCCGCTAACTGAAAGGCATGTCCAAAACAATGGGCAGCCCTTTCAATCATCCCAATGTCTTCAACGGATCCAGTTCCTAAAACCCAGCCCATAATAAACGCGATCTTAAAAAAGGGAATGGTTTTTTCTGGAGTGTCTACTAACTGACCGGAACCCGCGCTGTTTAGAGAGTGGCTTACAAAGGTACACAGCAGCGCTCCCAGTTGGTTGGGATCCGGAAACCTTGGACAGTGTTCCTTAATCCAGTCGATTTGCCGGCAAATATTTTGAAATCCTTGCATGGTTAGCGCCAGAGCGCTCATCTGCGCCTTGGCTACGCCAAAGCGGGCCCACACTGTATCTTTATTTCGCCGCTTCACATCGTTGTCAAAGGAGGGCATATCATCGATAATCAAAGAAGCTACGTGAAAGTACTCCGCTGCTAGGGCGGCCTCTGCCGGATAAATAGGCGCCCCAAAGGAATGTTGCAACTGACAGGCCCGAACAATTTCCATCAGGATAATGGGACGGATATACTTCCCACCTCTTAGAGCGTAAGAGCAAGGCTCTGTTAGTTGTCCCTTAAAGTCCCCATCTTCAATAGCATTATTTAAGATGGTCTCAAACTCTTCACTAAAGGTTTTATAATTTTTAGGATTCAGTGGATGTATTCCATGAAAAAGCGCGACACTACGCGGTGCTGTGATTCTAAAATACTTAGGTTTGCGCGTATAGGATATTAAAATAATAATAAGAACTACAATGATGGAGATATAGATGAGATGCAACATGCTGAGTTGTCTCCCCGCAGGGAATGGTCCTTTTCCGCGCTTGTTAACGGTACCGAGGAGGCGTTGAAATCTTTAGGAAAGGTGCTGTCTAGTTTGGAATCTCCAATTCCTCCCGTATATTTAGGTATATAATTATTGTGTCTAGAAATTGTTTGCTTTGAGGTATCAAAATATTCAGCCTGACCGCTATTTCTTTTAGAATAATTCGGTATAGGGCTTGAGTAGTTGGCAATACTCTTAAACCGGGGCACCAAGGTAACAATATTTTCCATATAATGGGTTTGATACGCTTTGTTTAAAAATGGGCTTACCGGCTTTATGCTTGTTAGTTGTGCATTGAGTACCGGTATGTCTTCTAGGATTTGTGGCTTTATAGAATGATTAGCAAACACAGAATGTAGTATATTAGATACTTGTAGCATATGTCTATTTGCGGAAAATTCCTGGTATTCTCTGCCGTGTTGCGAATCTTTGGGCGGAAGGGGACCAAGCATCGGCACGTCCGTGTAGGTACTGGTGGATTTTATGAGTTCCTGCTCTATGTTCGGTTTGACATGTGGATTTCCTAAAGGAATACCTCTACCTGCAATCCCTTTTTCTACCGACGCAGGTAGATTGTGCGCTAAACACAAAATATTGTACACGTCTTTGTGCGGAATATATCCGTTATAGTGCTGGCCCGGCATCTGATCGCCAAGGTGCTGCTCATGCTTAATGGTACCCTTTGTTCTGAGTTTAGGAAGATCCTCGTACGAAAAAAATTTTGTGTGCTCGCTGAACCTCGTAGAAGGAACCGAACTATTTTTTGGGTTTTTTAAGGAAGGCAATGAGGAAGGCTGGGTCAGACAATTTTTCTGTGTGCCCTTTAAGCTAGCCACCTGCGGAAATGTTTTTTTTTCCGTACGAACAACATTGCGCCTAATTAGGTTTTCCGTATGGGTTGAAAAAGCAGGACGATGATTTTTAAAATGATTAAAAAGTTTATTTTTTGGAATGGAGCTGTACGGCTCCAGATCTTGCGCATCGCCGTAACCAATGTTTTTGTGCTGAGGGTTCAGCATAAAAGAAAAGTTACGTAGATCACTGAGTTGCAATCCCTTTTCAGCCTTTTCAGGACTATTAGTGTATTCATTGTATACAGGCGCAGCTCCATTTTTGTTGCCGCAGTACCGGGAATTTAGTATATTATCAGAATACCGGTTATGACGCGGCAAATCGCTTTCCCAAAGAGGTGGATCTGACCTATAATCGGCTAACAGCTTTGAAGCATAATCATGATACATTGTATATAAAAGTTAATTATTATATTGAGAAGGCATAATTACTTCTTGTAGGGGTACAAGAGGCTTTGAATCAGGCAAACTGACGGGTTTTGAATCGGCCGGCTTTGGACCGGCAGGTATCTTTTTAGGTTGATCTTCTTCTAGCTCATTAGACACGGATGGGGGAGAAATAGGAGGAATAATTTCATCTCCGCCCTTATATTTGTCATGGATAGAAGAAACAATTACATCCATGTTTGATTTATTATAAATGTCGTTTAACTGGTGATTTAAAACATAATAATGCAAAAATAATAGGGCTACAATGCATATATATACGTAAATAGCCGTCTTCGTTTTTCGTTTTTTATCCACCGGCGGATTACAAATTGCAAAAAATACAACTAATACCACCGCTGTAATGATTAAGGCCACAATGAAAGGATTTTGAAAGGATGTTTTGAACGGTTCGCACGTATAAATTTTTTCTCCTAAATTATTGATACCCGCAATAAAATCTACATTCATTTTATATATTTATAAATTATGAAAAATTTAGAGTTACATCTCCGCCGGACCAATCATTGCTAAAATTTGAAGATTCTTCAAAAAGGCCCGACTGGTTGAATGTCTTCTGCTCAGGTTTCCAAAAATTTTCCAAGAATGGATTTTGAACAATAGGCTCATCTTGATTTTCTTCTTCAAGGATATTTTCTTTGATATCAAGAACAGCTTCTTTAAACTCAGGTGTATCTTGATTAAACTCAGGTTTATCCTGATCAATCGCAAAAATATTATCTTCTTCAGATATATCCTGTTTAATCGCAAGAATAGTTTCTTCCTCAGGTTTATCCTGATCAATCGCAAGAATATTTTCTTCTTCAGGTTTATCCTGACCAAACTCAACAATATCTTTCTCGCTAAATCCGTTTTTAGTGTGAAGCTCTTGGTTTTGAAGAGAATTATCAAAATCTATTTTAGTTGTTGTCCTAGACCGTGGCACGGGATAGTTATCTAATGGTTTACTTACTATAGTCCTCGAATGTGGCACGGGATAATTGTTTGGTGACTTGCTGGTTAGCTCTTGGCTTGTTAATAGTTCTTGTTTTCTCAATAATTCCATCTCTACTACTTCTTTTTGATCCGCTGGTGTCTCTTTTTGGTATTCTTCATTAGAAAAATGTTCAGAGGGTAATGTTTCAATAAACTTTGTGAGTGGATAGCTGCTCTTTGATGTAGAAGAGCGTTGAATTTGCTGATAAAGGAGTTGAACAAGTCGCCGGTATTCACTCTGTCTTTTTTCATATTTTTTACGTAGCGTGGAGAGATCTGCTAAGAGCGACTTGTTTTCAGATGTTAATTCTTCAATTTGATGAAGAAGGCTGCGATTGTATGAACTAAGTCTTGCATACGTTTCTTCTAATTCTGTCTCCGGCTCCACATAGGCCTGTTTTCGCAGAAATTTATTGTATAGTTCCATTCTTTTTTTGAGCAGAAAGGTAAGACTATAATCTTGCATTTCTTTCGTAACTTTATGGTAGTTTTCTTTCCGGTTTTTGATAATAAAGGGCAGCATTTTTTCTGTTGTGATAAAGGTGCCCAGATTGCTAATGTAGTCGCACAGTAGCAATTCCAAGATAGATTCTTTCTTTTCAAGGCTTATAGATTGGCTGTATTCTTTAGGTATGAAAGAATCAACAATCGTTGTTACGAAGTTTGAAAAGTTTAATGTTTTGCTGTTAATTTGGGTAATGTTACAAAAATATTTGTAAAAACTATCTAGCATTTTTTCATAAAGTTTTTTATTTTGTTTAACCCCTAAAATATAGCCCTTTACTTGATACTGATATTCCGTAACAATGGAATGTTTTTTGTATAGTGCATTTTTGTATAAAAAGTTATAAAAAATGTTGATAAAATACGCACCAAGGGTTTCAAAAATACTTATAACGTGGGATTCTTCCTGATCCATTATATCATATGTAATATTATTTTAATAAAAAATTACTGACGAATAACATGCAAAAAAAATATGTTTAAACTTATTTTAAGCTAGCACTTATTTAAAAGTGTTTTAAACACGTTTTAAATTGTATGTTAATACACTTAAAAATTAAGCCGAAATTTGCTCCAATAAGGATTACTTTTATCAATGACCACCTCTTTACTATAAACGGCTTTACATAATTTTAATAATGCTTTAGAGCCAAAGCTGAAGGCAGTGGGAAGCGGCACTGTACTATGGTAAAAATGTTGCCGATGTTCATCCTCGCGGATGTACACAAGTTTCCTATATCCTTTAAACACAATATGGCTAATTTCTTCCACATACTCCTTATCCTGTTTGGAATAGCGGTTGCTTTGACGGGAAAAATTCGACATACAAATAGAGGCATTTGTAAAAATGGAAACAAATGCGTTTTTACGAAGATTGGCGGGTAAATCGGTATCATCTTGGCAGCAAATAATCATCGAAATAAAACAGTGACGATTTTGGTAAAAAAACTTTTTAAAAATTTCTTTTGTAAATAATGGGTGCAGTTCGGCCGCGCAGTCGTCTAATATTAAAAGTAAACGAGGATTAAGATTGATATAGTTTAACGTAAACTTTTCATCCTCTGTAAGGCATAAGTTTTTATACATATGAATGTTCTGTATAATAATTTTTTTTAAAAGTTGCTGATAAAGCGATGTAATCTTTTCTTCTTTTTTTTGGTCCGTTTGTTCAGCCTTTAAGCACTCCACTTTTGCAATATTTTTGTTTTCCTTTTGCTGTATATCGATCGGAAGTTTATGATACAATGTTTTTAGCATATCGATGTTGTTTACTCGACTGTAGATGGAGGACATCATAGTTTGCCGCTGCCAGATGGCCTCCAAAAAGCGTTCAGCGCCCTTGTTGTCATTTTTTTTTTGCTTATCGGCGAGCCACAAGCGGTAGTGTATTAGAGTTGGATGTACAAAACCCTCATATGAACGATTTGAGGGTTCCGAGGGGGCAACCACTAAAATTTGTTCAATATGGGGTTGCAGGATTTTCATAATATGTTTAACGTACACGGTTTTGCCTGTTTTTGAGGGGCCATATAGCACAGTTGTTTTATCTATAAAATGATGTGCTTTGAACTGTAGTTCAGGAATTAGCTTCCCTGAATGGGTCGTTAGGGCCATCTCTATATTATTACAATTCTGCTTTTGTATATAAAATTTCTTTTTCGAGTTTATTATTATTGTTGACCCACATATCTACCCGTATCGTATCATCAGGCACATTGAGCATTTCAAGCGCATTATCTAACTGTTTTTTTGTTTTTATCAGCTCGCTTTCTTCATCGGGGGTTAAATTTTCTTTACTAAGCAGTTGCTTAATTTTTTCTTCGCAGTCGTCTATAAAATCATACTCTCGAGCTTTTTTGATATTTCCAGATGCTTTTTCTAGGTTTTTTAGCTCCTTAAAGGAAAGCAGTCCCTTAATCCCGCTATCCGTGTGAAAGGTTGAATTATAGATGGAGAGCCCCGGAGCATCCGGGCCAGTTTCTTGTATATTTTTTGCTTTTTTGTGGTAAATAGTATTTCGTAAAATCTCTTTTCCTATCTTTAGGTCTTCCTCATGACGGTCCAAAATCCGTTTTATTATTTCATTATTTTGATTAAAATAATTGTAGCGCTCTCTGTTGGCCTTAAAGCTTCCCAGGAGTGTCCAGTTGCCTAATTGAATGGATGAAACCTCTGAGAAAATCTGGTCTTTATATTTATAATAAAATTCATCAACCTTTTGTTGGTTGCTGCTATCCACCACATCATAAATAATGAAGGCAAACTCTAGGTCGGGTTTTTCTGGGTAGATGCTTTCCGTAGCGGCCCGCAACTCTTCGTAATTATCCTCAATGTAATAATTCCACTTATAAAAAGTATCCTGAGGTGGAATATGCTGCGAAAGATATCTAGTAATTTTTGTGTTAAAGAGAATGGGTTTAAACGCCCTCGGATTTTCAAGCATATGTTTAATGCTTTGGTGAAGTTCTATATTTTGTAATATGTGGGCTGCTGCCCTATAGCCCTGTGGGGTTTGGGTGATTGCATCAATATCGGCCTGAAGCTCATTAGGCACATTTAATGTTTTTTGCATGATGTGTAAAGGGATGCGCTCAGGATCTGCTAAATCGGTGTATTCTGTGCTTGTACAAGTGCTTGCACAGGTATCTACATTGGTATCTGCACACATGCTTGCACAGGTGTCTACATTGGTATCTGCACACATGCTTGCACAAGTGTCTACATTGGTATCTGCACAAGTATACGCACTTTGAGCATGAAGATTAGGATCAAACACAAAATGTTCTCGTAAAAAGCTATCGATCGTTGTTTTAGCTTCCTTGCTTTTCTGCGTCTGGGTTTTGCAGCTATCTGCTATAGATAAAATTGTATTTACTACCGATTCAGAGGGAACATCATTAGTTTCCTGTTTCAAAGTATCAACTAACGTTATTAGCTCACTGAGAAGAGTTTTGGTCGTGTGGGTAGGTTTTGAATAGGAAGGCATCCATTCCTGCAGAGCTTTGAAGACATATCCAATAAAGCTAGTCATTATAAGACGTCGAATATACTGCTCCCGCAAATTTGTAAAAGAGCAAAAGGCCACCCTGCTATCATTTTTGAACTGTTTGTAAGGGTTCGTCCTTTGGTAAAGCTGTTTAAGCGTTTCTTCGGATATTTCAGTAGAGGGATCCTCCAATACGTTTTTGAGAAGCTCATCAATATTAAATTCTGCCATATCTTAGAGTTTATTATATACATATTAAAGCTTTAATATAAGGGGGGGTATAACAATGGACGAAATCATCAATAAATACCAAGCTGTTGAAAAACTTTTTAAGGAAATTCAGCAAGGATTGGCCGCGTATGATCAATACAAGACCTTAATTAGTGAAATGATGCACTATAATAATCATATCAAGCAGGAGTATTTTAACTTTTTAATGATTATTTCACCTTATCTTATTAGGGCGCATAGCGGAGAAACGCTGCGAAACAAAGTAAATAATGAAATTAAACGTCTTATTTTGGTTGAAAATATCAATACCAAAATATCTAAAACGCTGGTAAGTGTTAATTTTTTACTACAGAAAAAACTTTCAACGGACGGGGTGAAAACGAAAAACATGTGGTGCACCAATAATCCCATGCTGCAGGTAAAAACAGCCCACAACCTTTTTAAGCAACTATGCGACACACAGTCCAAAACTCAATGGGTACAAACTTTAAAATATAAGGAATGCAAGTATTGTCATACCGACATGGTGTTTAACACCACGCAGTTTGGGCTGCAATGTCCTAACTGCGGTTGTATTCAAGAATTGATGGGAACCATTTTTGATGAAACACATTTTTACAACCATGATGGGCAGAAAGCAAAGTCAGGTATCTTTAACCCTAACCGTCACTATCGGTTTTGGATAGAACATATTCTTGGTAGAAATCCAGAACAAGAGTTGGGGACCAAACAAGATCCCTGCGGAACCAAGGTGTTGCAACAACTAAAAAAAATTATTAAGCGCGATAATAAATGCATCGCGCTTTTGACGGTCGAAAATATTCGAAAAATGTTAAAAGAGATAAACCGCACAGACTTAAATAATTGTGTTTCTCTTATATTGCGTAAACTTACCGGAGTAGGGCCGCCTCAAATATCAGAGTCGATTTTACTACGAGGCGAATACATATTTACAGAGGCAATTAAGATACGGGAAAAAGTGTGTAAAAAAGGGCGTATTAATAGGAATTATTATCCGTATTATATATATAAAATTTTTGACGCCATTTTGCCTCCAAATGATACCACGAATCGACGCATTTTACAATATATTCATTTGCAAGGAAATGATACGCTAGCTAATAATGATAGTGAGTGGGAATCTATCTGTATGGAGCTCCCTGAAATAAAATGGAAGCCCACAGATCGAACCCATTGTGTTCATTTTTTTTAAAGATGAAGATTTTTTAGATGATTTTTTTTAGTTTTTTAAAAGACGAAAAAATTTTTTAAAAGATGAATATTCTTAAACCCCGCAAATTACTTTTTTTTAGGTACTGTAACGCAGCACAGCTGAACCGTTCTGAAGAAGAAGAAAGTTAATAGCAGATGCCGATACCACAAGATCAGCCGTAGTGATAGACCCCACGTAATCCGTGTCCCAACTAATATAAAATTCTCTTGCTCTGGATACGTTAATATGACCACTGGGTTGGTATTCCTCCCGTGGCTTCAAAGCAAAGGTAATCATCATCGCACCCGGATCATCGGGGGTTTTAATCGCATTGCCTCCGTAGTGGAAGGGTATGTAAGAGCTGCAGAACTTTGATGGAAATTTATCGATAAGATTGATACCATGAGCAGTTACGGAAATGTTTTTAATAATAGGTAATGTGATCGGATACGTAACGGGGCTAATATCAGATATAGATGAACATGCGTCTGGAAGAGCTGTATCTCTATCCTGAAAGCTTATCTCTGCGTGGTGAGTGGGCTGCATAATGGCGTTAACAACATGTCCGAACTTGTGCCAATCTCGGTGTTGATGAGGATTTTGATCGGAGATGTTCCAGGTAGGTTTTAATCCTATAAACATATATTCAATGGGCCATTTAAGAGCAGACATTAGTTTTTCATCGTGGTGGTTATTGTTGGTGTGGGTCACCTGCGTTTTATGGACACGTATCAGCGAAAAGCGAACGCGTTTTACAAAAAGGTTGTGTATTTCAGGGGTTACAAACAGGTTATTGATGTAAAGTTCATTATTCGTGAGCGAGATTTCATTAATGACTCCTGGGATAAACCATGGTTTAAAGCGTATATTGCGTCTACTGGGGCGTCCAGCTATAAAACGTGACTGGCGTACAAAAAGTCCAGGAAATTCATTCACCAAATCCTTTTGCGATGCAAGCTTTATGGTGATAAAGCGCTCGCCGAAGGGAATGGATACTGAGGGAATAGCAAGGTTCACGTTCTCATTAAACCAAAAGCGCAACTTAATCCAGAGCGCAAGAGGGGGCTGATAGTATTTAGGGGTTTGAGGTCCATTACAGCTGTAATGAACATTACGTCTTATGTCCAGATACGTTGCGTCCGTGATAGGAGTAATATCTTGTTTACCTGCTGTTTGGATATTGTGAGAGTTCTCGGGAAAATGCTGTGAAAGAAATTTCGGGTTGGTATGGCTACACGTTCGCTGCGTATCATTTTCATCGGTAAGAATAGGTTTGCTTTGGTGCGGCTTGTGCAAATCATGAATGTTGCATAGGAGAGGGCCACTGGTTCCCTCCACCGATACCTCCTGGCCAACCAAGTGCTTATATCCAGTCATTTTATCCCCTGGGATGCAAAATTTGCGCACAAGCGTTGTGACATCCGAACTATATTCGTCTAGGGAATTTCCATTTACATCGAATCTTACGTTTTCATAAAGTCGTTCTCCGGGGTATTCGCAGTAGTAAACCAAGTTTCGGTACGCATTCTTTGTGCCGGGTACAATGGGTCTTCCAAAAGGATCTACAAGCGTGTAAACGGCGCCCTCTAAGGGTGTTTGGTTGTCCCAGTCATATCCGTTGCGAGGAAACGTTTGAAGCTGCCCATGGGCCCCCATCTGGGACGTGCCCTGAATCGGAGCATCCTGCCAGGATGAATGACATGCACCCAATATATGATGGCCCACCATATCATGGAAAAAGTCTCCGTACTGGGGAATACCAAAGGTAAGCTTGTTTCCCAAGGTGGGGGTACCCGTATGCGGGCGTACTTTATTGTATTCAAACCCTACTGGAACATAAGGCTTAAAATGCGCATTAAAATGCACCAAATGTGTTTCTTCGATTTGACTCAAAGTGGGTTCGGGATCGGGTTTCCCATAACTTTTGTTCACATTTTTAATGTTAGAGATCCTGCTATTCAGCAAGTCTTGGGCCAATATAATCTTGTCGGCCTTCCCATCGTTAGCAATAAGACAAAAAGCTCCTCCTGATGCCATATATAATGTTATAAAAATAATTTATTGTTTTTATTAAATATGGCGGTTTATGCGAAGGATCTTGATAATAACAAAGAGTTAAACCAAAAATTAATTAACGATCAGCTTAAAATTATTGACACGCTCTTGCTGGCAGAAAAAAAAAACTTTTTGGTGTATGAACTACCTGCCCCTTTTGACTTTTCCTCCGGCGACCCTTTGGCCAGTCAGCGCGACATATACTATGCCATCATAAAAAGCCTCGAGGAGCGCGGGTTTACTGTCAAAATATGTATGAAAGGGGATCGTGCCCTCCTTTTCATCACCTGGAAAAAAATACAATCCATTGAGATAAACAAAAAAGAAGAATATCTGCGCATGCACTTCATACAAGACGAAGAGAAAGCATTTTATTGTAAATTTTTAGAGTCTAGATGAGCTTTTACGCAATGTTGTACAGTGTTGTATATATGTCTTGTAAGCATTTGTTGTAGAGTAATAAGTAAAAGATAAATAAAAATGACTATTAAAATAAAGCCCAAACCATTAAAAATATTTTTATCTGTTAGATTTAATTTAATAAATGGCTCATGGAATGTGTGGTGCGCCGCTGCATGAGGTGTGGCCGCATGGGATGTGGTCGCATAAGATGTAGCTACATGGGATGTGGCATTTGCTTGCATGTAAGGATCATGATGTGTTGGGTCTTCATCCCAGCAATAATCGCCATCTTTATCTAGCTGAATTGTATACCCCATTATATATCACTTATTATTTTTTTTTAATGTTTCATGAATTTCATTATAGGCGGTGAAAGGGTCCTCAGGCCCCTTCTGTAAAAGATTATAGAGATCTTCGGACGCTTTATGTTTCGTGCGAATTAAGGCGGGATATAACAAAAGAGAGGGCCCCAGTTCCAAACAAATTTTACTTAGCGGGCTCATATTTTGCACCAAGTTTCCCACTACTTGCGATGTTTCATAACGCATTTTAAAGAGCTTTATCATAAAAGTGTTATGCAGGCCGGTGTAGTCTGGCCTATAGTTAAGGAAGGGGATTTCTCTGGTACCGTCAAACACGATCTCAAGTCCTCTAGCAAGCCCGATCAAAATTTCTTCAGCAATGGATGAGTATCTAATTCCTACATTACGAAGCGTAAGCATTTCTATAACATCATCTATTTCCTGCATAGAGGAATCTATTGTAGGAATTTTAATATCATCTGTGCTGATTTGTTCATTCCCAAGATAGGTAAGCAGCATATTAATTTTTTCTAGCTTTACTAGCTTAGTCTTACGCTCATAATCATGATCTTTTTTATAAAAAGAGTTGGGATCACCGTTGGACCGTAGATGATTAATAAGGCGGTCTACTTGCTTTGTACTAGGTTTAATACTTTTTTCACTATACTCGCTTTCAGCATAGTGGTTTTTACGATCTCTTTTAGAAATAGCTGTTTTTTGAGATGCCTCAGACTCTGCATATTTTTTTCTATGCGTAGAAAGAGAATAACCGCGGTCATTACGTGAACTACTGTTGCATGCAAGGCCTCGGCGCGTCTTACCGCTGCGCACACTGCCATTGCGTATACTGCCATCGCGCACACTGCCGCTGCGTATACTGCCATTGCGTATACTGCCGCTGCGTATGCTGCCGCTGCGTATGCTGCCGCTACATACACTATCACTACATATGCTGTCAGTACATACGCTATCGCGGCGTATGCCGCCGTGTACCTTATCGCCGCCCCTACCCGAGGGTTTTTTAGATATAATACTGTGTGGGGAGTCAAGCGAAAATTCAGGGTCATTAAAGTTAATGCCCAATGACTTTGCCAATCCATTAAGCTCTTCATCAAAATGATCGGTAGGAAAACTTTGTTGCTTGCCCATGACCTGTTTTTCAAGTTCCTCCAAATTGGCTTGCTCATTTATATGGAGATTATTCATAAGCGTCGTAATTCCAGCAAGATTTGCTCCTTCTAAAAATGTGGTGTCCTCCATCGGATATACTATACTATTTAAAAGCTTTTAAATAAAAATGTGTTTGGAAGAAATGCTCTCTTCAAGCGTGTGTAGCTCAGATATAAATGCCTCCTCAGAAAGCTTTCCACCATACTCCTTTCTCATCGTATAGGAGGGCGCCGGTTTAATGTAGGAAATCCACTGGGAGGTAAAAAACCGGTACAACATATTTAGCAGCTCGCGGGCCTCCCACCTTTTGGGCTCCGTATAGTGCACATCAACATAAGAGGCGGCGCATGAAAAGCTGCAAAAGTTGCCGAGAACGCCCATCTCAATCTCTCCTCGCTCATTTTCACGCATATAGGTGGGCACGAATTTTGGGACAGTCTTGAAATAGAGATGACATGTCCAGCATTTAAAGCTAGAATGGGTAACCCATTTGGAAACAGTGGTGAATACGGAGGGTAGCTTTTTTTCGACCTCGGCTTCATCGTCATTCGTATTTAACGTATCGGTGGCAGTTTTTTTGGATTGCAAGCATTCTTCAATGGTAATCCCGGATAAGTATAAAATATTAGGACAATTAGTTTCCATAATTTTGATAGTTATTTTTATACAACATGGATTTAATTAAAGATAAATGGAGGACGAAACGGAACTGTGTTTTCGGTCAAACAAGGTGACGAGGCTTGAAATGTTTGTCTGCACATACGGGGGAAAAATTACCAGCCTTGCATGTTCGCATATGGAGTTAATTAAAATGTTGCAAATTGCTGAGCCGGTGAAGGCATTGAACTGCAACTTTGGCCACCAGTGCCTACCGGGCTACGAATCTTTAATAAAGACTCCGAAAAAAACTAAAAACATGTTGCGCCGTCCGCGCAAAACAGAAGGCGATGGGACTTGCTTCAATAGTGCCATTGAAGCCTCCATTTTGTTTAAGGACAAGATGTATAAATTAAAATGTTTTCCTAGTACCGGGGAAATTCAGGTCCCGGGCGTCATTTTTCCGGATTTTGAAGACGGAAAAAACATTATACAGCAGTGGGTAGACTTCTTGCAACATCAACCCATTGAAAAAAAAATCCAGATTATTGAATTTAAAACGATTATGATTAATTTTAAGTTTCAAATAAACCCAGTGTCTCCCCGCGTCATCATTCATTTAAAAAAATTTGCAGCTTTGTTGGAACACATCCCTACTCCATATCCCATACGTGAAATAAAGCCTCCATTAGAAGACTCAAAAGTATCCGCAAAATTTATGGTCAGTCCGGGAAAAAAAGTACGCATTAATGTTTTTCTTAAAGGTAAGATAAATATTTTAGGCTGCAACACAAAGGAATCCGCGGAGACCATTTATACGTTTTTGAAAGATCTTATCAGCGTACATTGGCAAGAAATTTTGTGCGTGTTACCGGTACCCGATTAAAGAATGTTTTCATTAATAAGGTAATCGACTATGCTAAAAAGAATAACAAGAAAAATACCTTGAAGAACTATACCAAAGTAGGTAGGTTTTCTGCATGTCACGGCATGGTTAAAATTGCTAATAATGTAGTCCACAAAAGCATTGCTCAATACGACTAAAAATAGTAAAAAAAGGATAAGTGCTCTTTTTATATCCATATACTTTAAAACTTATTTTTTACACTAATAATTTCCTGCGGCCGCAATATAAACTGTAGGTCATCTATAACGCCCAGACCTGTTAAAAGTAGAGTACTATGTTTTAAGGGATTTAAAATATCCGCCGCAAGAATGTGAATATAATTTTCAAAGTGGTTTACAGGAATGCGTAAGCGTTTTTTTTTGCACTGCGGTTGGTTTAGGGTCGAATACTGGCAGGAGGTATATATATTAATAAGACCGCGGTCGATGGTTTCAATATCTTCATAGAATTCAATGCGCGGCGTCAAAAGTTTTTTAAGATGTTGACATAACTCATCATACGTGTAGGACTGGAGGGGGGAAAGAAGGGTGTAGTCAAAGTTAAAAATGTTTTTTTGAAGAACCTTTAAAGCATGTTCCGCGTCCGTGGTTTCCAAAATATGTTTTATGGTATGAATGTCATTTAAATCTACAAAGTCTGACAGCTTTGTGTAGAACTCGGTGACGGAGGTTATTTTCTGGAAATCGGTTTTTTGAAAAAGATTTTCAATGTGTTTGCGGGTTGAGTTGCTTTGCAGTCCATACAAGACATCAAAAAATTCAATCAGCAAAAACTTATACAAATGGTTAATATAAAAAGCTTTGTTGGCCTTATTCTGCTGAGGATATGGTTCCTCTAGGGGATATAGAATGGCTTGGTCTATATCCCTAGGATCAATAGTCAATGTTGCGATGGGAAGCTTTTCCAGCGTAGCGGGAAGAGTTTGGGTTGGAGCGTAGTAAAAGTATAGCCCGGTTTTTCCCTCTGAAAGAAAGCCCACAAATTCTTTTTTTATATTTTGCAGCACCGCTGAGGGTACGATTTCGTACTGTTTATACTGTTTGTTGAAAAGGGTAATAAATTTCCAGGTTTCTTCAAAGCTTGCAATCTGGGTGGGCCGCAGATCAAAGTCGATGGGAATGTCGTCATGAATGTAGGATGATAGTCTTATAGGAAAATAAATAGGGCGATCGGTGTCTGAATCGATAAGTAAAGCATAACAAAAGTTATGCCTGTTGATAAGTTTTTTACCAACCGTGTAGCCGGGAATGTTTTTCACGTCATGGATATCCCACCAGTTATCCTTGCACATAAACTCGCTCATAGACTGGATGACCTCCATCACAGGGTCATCTTCGGTAAAAATATACTGGGCCTCACTGTTTTTCAGAAATCTTTTTTGCTGGGTGATGGCCATTGGGTAGATCCCTTCGTCCGTGTCAAAGATAATGGCTATCTTCTTCGATGGGCTAAGAATTTTTTGTATTGTGCTGGGGGACACCTCAAACCCGATGTCGCCCTGTTTATCTTTAAAAAAGACACAGTGAAGGTCGTAGCATATGGCAACAAGGTCCAGAAAGATGTCCTGCCATGTGGTGTCCCATTGAAGCAGTTGGTTTTTTTGTTCAACAAAGGTTTGTAAGATAAGGTTTGCCAGCTCCGCGCCGCTGGAAAACATGTTGCCGGCCCCATTCCCCAAAATATAGTACTGCGGTGTGTTGGCCGCCTTTGCAATTTCAATGGCAAGGGCCTTGGGGGCAAGATCCAAAATTCGAGCAAGGGAATAAAAAAGCCCGGCATTGCTAATTCCAAGCATGGTTTGCTCCACCCCCACAATGCAAAAAATGTCGGGCTCTTTTATCGTATTTAAAAACAGTTCATCTGCTATCTGGTGGGGTAGAAAGGCAATCCGGTTCACCGGTATTTTTTTTCCATAGGACAAGGTATGACGCGATGTTTGTGTATTAAGATCCTCCAGGTCTTGTTCTACAAACGTGTGCTTGGTGAGGCAGGTATTGTTAATATAGAACCGCTTTGTGCCCAGCAGGGCCTTCGTCTTTTGGCAGCACGGCAGACAGTAATTTAGGGGGTGGCGGCCTTCTAGTAGGCTTAGATGAGGGTAGTCAGGATGCGGGCAGCTATAGTAGGCAGGTACCCCCTCCGTGAAATTCCAATACTTTACTAGCTCCTTGCGCTTGGCTGGCGGCATGGACTTCACCTCGGCCTCTGAGTAAATGACGGGTGGCCGTGGGTGCTGGCATAGGACGGAGTAAACCGTTGCCTGCGTGTCGTACTTGCGCAGGTCATACAGGTCGGGGTCCTGTTCTTGAAGCGCACGTAGCTGAGAGGCTCCCTTTCCTTGTTGTTTATCGTGCAGTTGAGAGAGTTTATTAACCAAAATTTTGTCAGGCCCGGTGATCAAGTTATCTAAAAACACAAATAGGTAAACCCAAAGATAGTTAAACTCTTCCTGGGTAATGTTAAACATTTCTATTTTGATATCTGTAACCCTATGGTAGATGCGAATGTTGCGGCCGCCGTAGATTGTTTCCCACCGGGCCGCAACATTTGTGTCAAAGAGGTACGCATACGTGTTTTGGAGCAACGCAACATTGATGTCCATTTTGCGCCCCGGACCGGAGGAAATAATGATCATCCGTTCGATTTCGTGGGGATCATACGAATAAATCCCCTTTTTAAATAAAAAATTGTAGACCCCGGTTTGCTGGAGGCCCCGCACGGAAATAATCCCTGCTTGCTCGTATTCCCGCCAACGACTTTTGAGCTCGGTAAATCCCTTGCTAGAAAGCGTATAGGGCCAAAAGGTGGACACCGACATGGAGCTGATAGAAATTTGGATGTCCTCGTTGGAGGGAAGGGGCAGACTCCCTCCACGAGGAAACGCGGCAGGCCCCATATCATTAATTGTATGAATAATAGGATTTATGAAATTATTTAGGGTGGACACCACGGAGTTAAAGTCGTGGCGCTCGTTTTCTGACCAATTGCTTTCGATAAAGTAGTGCCCATTATTTTGTATGGTAAGAATAAAGGCCTTTTTATTGATAAAGCGTATTAAAATAATAGTGGGTACACGGAATGTTTTATTGCTGAATTTTTCAGGCTCCGTGGAAGTTATGTGGTGTTTGGAAACCACGGTGGGACCTGTTTTACTATAAAAGAACACCACCAGCTGAGGAATATCGGGAGTAGCTGGAAATAGGTCGAAAACATTGCGCACATTAATTTGAATATTTACGAGGGGTGAAATTTTAATCATTGCCGAGGTGACGGCCAACGTGCCGCGTGTTAGTCTATTCCCCTCGTACTTGGCAATGACTTGTTGTGCTCTGGCATACGTAAAGTTTATTAGTTTTTGCTCTAGGAGAAGCCTCTTTTTAAGACTGGTCAAGGATGGAGAAAGAGCAGGATACTGTTTTTCCATTTGTAAGGGAGATTGTACCAATAGTTTAAAGGCATCGGGGGAAAGAAGAGGCCAATACTTCATAATAAGGCCGTAATAGAGTAAGTCAAATTGGTAATTATCCTCTATGGCAATGGAGATTTGGCGCCGCATGGGGGCCACTAGCGTGTTGAGGTCTGCTACAAAGATGTGATGAATGTTTTTTATGAGCTGGAAGCTGTCGAGCGCTTCCACATAGAGCTCATCTTTTTGACTTTCCATAGATGCGTCGATGTTCACCCCACCCACCTGTTGAAACTCCTTTTTGTAGTCGCGAATGTCTAACGCCACCCCGCTACCGCTTAACAATAGGCGATACGTTACCTGAAGCGCATTGTTTTGAAAAAAGAAAATGTGTTGTCTATAAGGGGGGATCCCTGTGGCAACGTAAATTTTTTCTCGAATGTCTTTAAAAGTGTCTTCAGGGAAAATACTATACTCGCTATACATCGTCTCAATTTCTGGCATCATCACGTTTGTCTCCTCGCCACGATCCTCCACAAAAAGTTTTTCAAACTCATCTAAATCATCGCTATCTCCACCCACCACGTATTGGGAAAGCTTTTTCTCCCAATCCTCGCCGTAAAAATTTTGTAAAATTTCTTTGTCCTTAGGGGTTCGCTGCAGGTCTTTGCGGCAGGCCTGTAACACGTTTGCAGGAACGGATCCCAAAAAAATAAACGTCTTCGTGTACTCATTTTCCACAGGATTATAAAGAGTAACTCGTAGAGGATTTGTTAAAAAGTCATTTTGGAAATCCATTATACCCGGTATAGAAAATAAAATTTAAAATAAAAAACGGATGATATCTATCATGGACCGTTCTGAGATTGTTGCACGGGAGAACCCGGTGATTACCCAACGAGTTACAAATCTCCTACAAACCAATGCTCCTCTACTATTCATGCCCATTGATATCCATGAAGTACGATATGGAGCCTACACACTTTTCATGTATGGTTCCCTCGAAAACGGTTACAAAGCAGAAGTAAGGATTGAAAACATCCCAGTTTTCTTTGACGTACAGATTGAGTTCAATGATACAAACCAGCTTTTTTTAAAGTCGCTACTGACGGCTGAAAATATTGTGTATGAACGGCTGGAGACGCTCACCCAGCGTCCTGTAATGGGGTACCGCGAGAAGGAAAAAGAGTTTGCACCATACATTCGAATATTTTTTAAAAGCCTGTATGAGCGACGAAAAGCCATTACTTACTTAAATAATATGGGCTACAACACGGCCGCGGACGACACAACCTGTTATTACCGAATGGTTTCCCGAGAATTAAAACTACCTCTTACAAGTTGGATACAGCTTCAGCACTATTCCTACGAGCCTCGCGGCTTGGTACACAGGTTTTCCGTAACCCCCGAGGATCTTGTTTCCTATCAGAATGATGGCCCCACAGACCACAGCATCGTTATGGCCTACGATATAGAGACCTATAGCCCTGTTAAGGGAACCGTTCCGGACCCAAATCAGGCAAACGACGTGGTGTTCATGATATGCATGCGCATTTTTTGGATTCACTCCACAGAGCCTCTAGCGAGCACGTGCATCACCATGGCACCCTGCAAAAAGTCCTCAGAGTGGACCACCATTCTATGCTCCTCTGAAAAAAATTTGTTGTTAAGCTTTGCTGAACAGTTTAGCCGCTGGGCTCCTGATATATGCACAGGGTTCAATGATTCTCGGTACGACTGGCCCTTTATCGTTGAAAAATCTATGCAGCACGGTATTCTAGAAGAAATCTTTAACAAAATGAGCCTTTTCTGGCACCAAAAGCTGGATACCATTCTAAAATGCTATTACGTAAAGGAAAAGAGAGTCAAAATCTCGGCCGAAAAATCGATCATTTCCTCCTTTTTGCATACCCCTGGATGCCTACCCATTGATGTCCGCAACATGTGTATGCAGCTTTACCCTAAAGCCGAAAAAACAAGCTTGAAAGCGTTTTTAGAAAATTGTGGGTTAGATTCGAAGGTAGACCTGCCGTACCATCTCATGTGGAAGTATTATGAAACACGAGACAGCGAAAAAATAGCCGACGTGGCCTATTACTGCATTATAGATGCCCAGCGCTGTCAGGACCTTCTGGTGCGCCACAATGTTATCCCCGATCGCAGAGAGGTAGGAATTCTGTCATACACCTCGCTGTATGACTGTATCTACTACGCGGGAGGACACAAGGTATGCAATATGCTCATTGCCTATGCCATCCATGATGAATACGGCCGTATTGCTTGCAGTACCATTGCCCGAGGTAAGCGGGAACACGGAAAATATCCCGGCGCCTTTGTGATAGACCCCGTTAAAGGGCTTGAACAGGATAAACCCACCACAGGTCTCGACTTTGCGTCGCTGTACCCCTCACTCATCATGGCCTACAACTTTTCGCCAGAAAAATTTGTAGCCTCTCGGGATGAGGCAAATAGCCTCATGGCCAAGGGTGAATCTCTTCACTACGTCTCCTTTCACTTTAACAATCGTCTCGTGGAAGGATGGTTTGTGCGGCATAATAACGTTCCTGATAAAATGGGATTGTACCCAAAAGTACTCATCGATCTACTTAACAAACGGACCGCCCTTAAACAAGAGCTTAAAAAACTAGGTGAGAAAAAAGAATGTATCCATGAATCCCATCCTGGGTTTAAGGAACTACAGTTTCGCCATGCCATGGTAGACGCGAAGCAAAAGGCGTTGAAAATTTTCATGAACACGTTTTACGGCGAGGCAGGTAACAATTTGTCGCCCTTCTTTCTGCTTCCTCTAGCCGGAGGAGTCACCAGTTCGGGTCAATATAATCTTAAACTTGTCTATAACTTTGTTATCAATAAAGGTTACGGCATCAAGTACGGTGACACCGACTCATTATACATTACATGCCCAGATAGTCTTTATACAGAGGTAACAGACGCATATTTAAACAGCCAAAAAACGATAAAACATTATGAGCAACTCTGCCACGAAAAAGTGCTTCTGTCTATGAAAGCCATGTCTACACTATGCGCCGAGGTGAATGAATACCTGCGACAAGATAATGGCACCAGTTACCTACGTATGGCCTACGAGGAAGTACTCTTTCCTGTGTGCTTTACAGGCAAGAAAAAGTATTATGGTATTGCTCATGTAAACACACCCAATTTTAATACAAAAGAATTATTCATCCGCGGAATAGATATCATTAAGCAGGGTCAAACAAAACTCACCAAAACGATAGGAACGCGAATTATGGAAGAATCCATGAAACTACGCCGCCCTGAGGACCATCGCCCCCCTCTTATTGAAATCGTTAAAACGGTTTTGAAGGATGCTGTGGTTAACATGAAGCAGTGGAATTTTGAAGACTTCATCCAAACAGATGCGTGGAGACCGGACAAAGACAACAAAGCAGTCCAAATCTTTATGTCTCGCATGCACGCTCGGCGTGAGCAACTAAAAAAACACGGCGCTGCAGCATCGCAATTTGCTGAGCCCGAGCCGGGAGAACGCTTCTCCTACGTTATCGTGGAAAAACAGGTACAGTTTGATATCCAGGGCCACCGCACAGATTCCTCCAGAAAGGGGGACAAGATGGAATACGTCTCTGAAGCAAAGGCTAAAAATCTTCCTATTGATATATTGTTTTATATCAACAACTATGTTCTAGGCTTGTGCGCGAGATTCATTAATGAAAATGAAGAATTTCAACCCCCTGACAACGTCAGCAATAAGGATGAATACGCTCAGCGCCGAGCTAAATCCTACCTACAAAAATTCGTGCAATCCATTCACCCTAAAGACAAGTCTGTCATTAAGCAAGGCAATGTTCATCGACAGTGCTACAAATACATTCACCAAGAAATTAAAAAAAAAATAGGCATCTTTGCCGACCTTTATAAGGAATTTTTTAACAACACCACAAACCCCATCGAAAGCTTTATTCAAAGCACTCAGTTTATGATACAATACTTTGATGGAGAACAAAAAGTAAACCATTCTATGAAAAAAATGGTTGAACAGCATGCTACGGCTAGTAATCGAGCTGGTAAGCCCGCTGGTAATCCAGCCGGCAATGCGCTGATGCGGGCTATATTTACGCAGCTGATTACGGAAGAAAAAAAAATTGTACAAGCCTTATACAATAAGGGGGATGCAATACACGATCTTCTCACCTATATCATTAACAATATAAATTACAAAATTGCCACGTTTCAGACGAAACAGATGTTGACGTTCGAGTTTTCCAGTACTCATGTAGAACTGCTATTAAAGCTGAATAAAACGTGGCTTATTTTGGCTGGAATTCATGTGGCAAAAAAACATCTGCAAGCTTTTTTGGATTCATATAACAATGAATCGCCGTCTAGAACATTCATTCAGCAGGCTATAGAGGAAGAATGTGGCAGTATTAAACCATCTTGCTACGACTTTATTTCCTAATACTTCTTAAGAAACTCTTTAAACAAGGACTTCGCATGGTCAAAGGTTCTAAACCCATGGCCCTTATGATTCGCCAAAAAAGCGGTTTCATCAAGATTTTCTAACCCTTTCACGGATGAAGAAATAAGGTGTTCGGCCTCGTTTGCCCATTTTCTATGATTTTTTTTCACCTCGGGTTCTAGATCTGTTTTCTCCATATACTCATTGTGGTCATATTTTTTTTTGGGAGGAGGCGTGGGTGGAGGAATGGGTGGAGGAAGTACACCCGACTTTCCCGCTTCAACCGTTTTATAAAAAAATAGAAGCATAATACAAAGAATAAGGACTATCGCAAATATGATAACCAGTGTCCCAGTCGAGGGCATTTTGTTATATAAGTAACGTTTTTTTTTATTTTTTATAATTCGAATGAAGAACCATGTTGAATAGTCTTCTACTCAAAGACATTTTGTTATACGGTAAATGAGAATTTATAAAATCCGAATATCACTATCATACTGTTTATCTGAGAAGGTCTCACTGGGTCCTGTGATGGAGAACCCATACTCTGTAATGCTGGGGTTTATAATGTGGTCAGGACTGACAAGCACATTTCTGAACTGCGAGAGTTCTAGGTTTAGACGCAGTCGTAATAGTCGCTGTATATTTGTAATAAATATTAGATTGCGTATGAGGCGAGTGTCAAAGCGATCCTTTCCAATTTGTACTAAGGTGGGCTTTTGTATTCCAACTCCCACTTGTTTAACGATGGACCAGGGTCCTTCTTCCCGATTTTGTTCCGTGATATAGGTCAGCACACTATTTTCTGTATATGAGGTATGATGTCGCATATTAATACCTGGTGCCATTCCAACTGGCGGTTGTGCAATTCGGGCTGTACCGGGACCCAACCATCGTGGAGTTTTATAAACATATCGTTCTAGCGTATTTAAAAATTCCTTAAGGTTATTTACGAGTAGCATGAAGGGTGCTATTAAAACAGGTGGATGGTTTATAACCATTGTCATAAACCATTGCATTGCTTCAATATCATTTTGTAATGCTTGACGGGGAGGCGGGGCAGGTAATCCACGTATGTTGAATAAAGCGGTTAATTGTGCACCGGCTGTTTGGGGCGTAATATTTTGTATTAAATTTATCATCGAATTGGCTTGCCCGGCATTTCCTATAAGATCGATTAAATTGGTTATTTGACCTCGATATTGTTGTACCCAGTTTTGAATGGCAGCGATGATCTCAGGGGTTGGATTGTTTTGAATTTCAGGTGTTTGTATTAGATTATTCACTTCTCTTCGTGTATCTTCAAGCTGAGTCCTAAATGCATTTAACTCACCTATAATTTGGTTTCTATCAATAACATTTCTTAAACCTCGAACTGTTTCAGCCAATCGTATAGTACGCACAATTTCATGTAAGGCCTGGTTTATGTATATTGACATGGGATGGCCCCACCGCTCACGTCCACGTTGAATACCTGCGGCCAAACTAGGACCTGCCTCGTCATAATCAAATTGTGTAGGATAAAGGCTTCCAAATAGCACTTTATTGAAAATTTGGTCAGAAAGAAATTTAGGGCGGCCCATATTTAGCGCGTTGTCCCCTCTAAAGATGCGTGACATGTATCCGGCGTTGCCTTTGGATAGTAACTCATTCCCATATTGAGTAATAGAGACCGAGACATAGGGGTTTATAAGAAGTTTTAGCATAAATTCTCGAGTATTTATGGGGGGACGATTCGGAATGTTTAATACCTCTGCAACATCTGGTTGAGGAGCCGTGGTGTCCAGAGATCGTACTTTTTCAGCCGAAATGCCGTACATAAGACAAGCAATTTCTTCAAAACTATAGTCATAGTTGTAAATATTGGCAAGTGGTATAGATCGCATCAGCGCATTTACATTGATAGGTATAATATTCATATCAAACAAGTTAAATATGCGCTCGCGCTCTCTATTAGAGCCAAGAGTGCGTGTTTGACCTTTCGGCGACACTATTTTGTGAATATGATTGATTTGCTCCTCTTGGTAAGAGCTTTCCACGAAGGAAATTACGTCTTGCAATGTTTTACGAAGCGAATACACTGCATTCATCCCTATTCCCGCTGTTATAATGGGTTTATCGTCTCTGTTCTCGCTAATAAGATTAACTCCACCAAAAGTATTTTCATTGTACATCATCACTGTTTTAAAACTACGGATATTTATGATAAATCGGAGAGCCTGAATGGCGTGGGTATAAAAGTGTTCAAATCGCGTGGGAGTAATTTGTTCGCGAGCAACTACCGTTTCATTATAGTTTTTCATGATAAGCTGTACTCCGGGCATATCTGAGAGCTGTACCGGATCATTTCCCAGTAATTTTCTTGTGCCGTATAGTAGTTTAAACTCGGGGGAGCCGCTTTCAAGGTTCGGGTAAAGAAGAGGATCATATACCTCATTATTTTCTATTCTTAGGTCATGTAAATAATAGAGCGAAAGTGAAAATGGCATAAGAGGCTCCTTATTGTACCGGGACATATAGTTTTGAATGAAGTGTTCTTCTGTTTCAAGATAGATGGGATGATCGGTAAGCTCGTGCAGGACCTCCATGGCAGAATCTGCCAGAGTGTGAGAGCCTCTAATGATCCCGTCGATCACTGCGACCAGTCGCTTTCGCACAACATCGCTCGTATTATTTTGTGCGTCTCCTAGGGGCATAAGCGTAACATTGGGACGAAATACGCCGCCAATTCCCCGCAGGGCCGCCTGACCGACGGATAGTCCTGTCGCAGGAACATTGTTATTATTATAATAAATAACGGAATCATTATTGGCTCCCAAGAGTGCCGTCAGATTAGGGCGAGCTAGTTGGACATTTGTGTATTGTATAAATTGTTTTAGAAGCTCTCCCTGGCTAATAAGAATATTAAACATTTTGTTAAATAGTGGAAGATTGGCTCTATAATTTTCTTTAAGGTAAATGGGAATTTCTGTTAAAGTAGAAATAAGATGCTGACTCAGGCCCTGGCGATTGGTATCCTTAATAAGCCGCTGAAGTATAAGTCCCAAAGACAGAAGAAGCACCGACTGCTCTGTGGGGTCGCCTCTATGACCAAAGACGTTGTTATTGCGTGCTAAGTCAGGGTGAGCATATCCCATCTCCATCACTGCTTGGCTAAAGTTCCCATTAGCGAATGCATTAATAAGATTTAGATATATTTTTCCGCTGGGAGCATCATAAAATCGGGTAATATATGAAGCTATGAGCTGGTTAAACACCATCATCATACTACGATTATTTTGAATACCATAGTCTGATCCGTATAGGCGATAACGTCGAAGGTTGTTTGCGGCATCATTGACATTGGCATAGGTTCTGAGCGCTATGTTGTCCCAGTAGCTAAGAGTATTTTCCTCCTGGGCGTTGTTGGTACGAATAAGATTGGAGAGTCTAAAGTCTCCTAGTGCCACCTGCTCTACACGAAGTCCAGAGTTATTCTCCAAAGCATCGTAAAATACGAGTCTACTGAATACTCTTCCGTATTGTTCAAAGCGTTCAGAGGATTGGGGATTGTTATTTATTTGAATATTAGCCGCGTCCCTTCTTTGCGCCCCACCTCGAAGTTGCAGTACATTATAAGGCTTTGTAAGCAAGGTGTAGGTTTTATTAATGATTTGGTTAACCCCCTCCAGGCCCAATTCACCGCCAGGAAGCGGCCTTCCTCCGGCATCGGTAGGTGGTTTAATAAGTTTGTCAATTAAATGTTCTTCCAACCAGTAAAATGAGCCAGGATTAGATCTATTTTCATAGTATTGAATAATGTTTTTATCAATATGCGGGCGTAGAAGATCAAGAAAATACTTCGTGTCGGCCATCAAAGAATCAATTAAGGAAATAAGACCTGTAAAATCTAAATGCACTTGAGCGGTGCTGGTTTCAGGGAAGCGAACTTGAACCATTTTGTTAAAACTGGAGGTCATTTCGAAGATATTGGTCAACAGGAGCTGCATGATTCGCTGATTATCTACTAAATACCTTGCGGCCAACTCTTGCTCCGGACGAACTCCTCCACCAGCAGGAATACCCACATATGGTACAATCCAAGCAAAAAGAGTTTCTGTGGTTAAATTTCGGTCTTGGGCTGCTGCAGCCGCTTCGGTAGTGGGATCAGGGTACACCATAGAAAGCCGCATATTGATTTCTTTAATGACTAATCCTGGATTTCTAATCTCAGAGATGGCCCCGTGTTTTCTTCCGAGCCAGTCAATAAGATTGGCGCGGTTCACGTTGGCAGCTTGTGTCTCTCGTAACCATTCGATAATGCTTTTTTGAATCGTATCTAGGTCTAAACCTTTAATGTTATTACGAAAGTTATTAAGAAGTACGTAAATAGCACTCAATAAGTTAAGACCTGTAATAACGGTTTCATGAAACAGAAATATTTTGTTAACATCTGTATCTGCCAGTGACTCAGAGCCTTGAATAAGTTTTGAAACGATTTGAATTTTATCGGTATGCTCCTTTTTGAGTTCATTGATAGCCTGGCGAATGAGTTCTTGGTAGGAAATTTTGCCCAATTCTTGTTGCAGACTGGGATCTTCAAACATCTCACTAAGCTGTTTCCTAAATTTTTGTACCAAATCCCACTGGGAGTTGGGCTGCAGCATTCCTGTTTGGACATCCACAGAGTCTATATTGTATAGTGCCGGGCGCCACTTGGGGGTAGGCTGGGTTGAAGGACTAATAAACCTATCGGAGGGAAGTAATTGTGAGGATTGTGTATAGCCATCCTCATCAGGAAGAATGGAGTAGTTGGTTTGATTCATCATTCCAAAATCATTCATAGTTCGCGCTTCCTGAACAATGCGTTGAAATTTTTCCCATTCGGTGCGTGTAATGACACCGAATCTGCGGTTTATTTCATTTACAAAATGGATAAGCGCTTTTTTGGTTGCTTCTTGTTCACCATACTCTAAGTTAAAGTGTTGGTAAATGACGTTTATTTCTTTGATAAGCTGACGAATTTCGGTTTCTGAGTAGTCACCAATGTTAATAAGCTCAATAGGACGCATAAAGATAATGCGAATAAGTCCTGAGAAGATTCCTTCCAGCTCAGGAAGCATCGAGATCTGTACATTTTCATCTCTAAAGGAAAACAACTTTTGATAAAATTCGGCGAGGCGGGGAAGGCGGAAGTAAAGCTCTGCTGCCTCGGGAATTACCTCGGGCTCTAGCTCATCGGCACCCCCCAATATCATACGCGTGGGTATAAGTTTGTACACGGGCTCAGGCCGTTCAAACATGTCGTAAATCCCTAATACAATAAAAATCTTGGCGGCCATACTTTTCAGCATGAAGGTGAAGAAGACGTCCTCGGTTTCCCAGCGGGTTGATAGGGCGTCGTTAACTCTCACAGTAGAGAGGTAGACCCGCTGAGCCGCTTCCTCGGCAGTCTGTGCAAGCGCCATCCTTTGTCCTCCAATTTCTGATTGATTTAGATTTTTAAGTCCCACGGAAAGCGCAGAATGTTGAAGATATTCAAGCAAGGTTTTATAGATTTGCAGGGGCGACATGGGCACCATTTGCCGCAGCTCCTCTCCCCCAAGCATGTCCCCAATCCGGGCAAAGGCATTGATGATATTTTTAAGCGCCTGAAAGTTAGAAAGAGAGCGCCCGATAAGGTCGCGAATGTTTTTAGCCTGGCTTGCTCTGACGGGACGGAGGGTACCAACGCTTCGGCCTTGTTGGATTTCAGCCGCAACTTTTTCGTAGTAGTGGCCCGCAGGAGCATTATCCGTAAAGACGTTGGAGTCGTTGCCTGTGGAGGTGGGAAAACTTTCAAAGACTTGTGCAAGCGTGTCCCCTGTTGTCTCGGTGAACCATCGTCCTATAATGCGCACGCCATCCAGCATCTGTTGGACTGTTTGAATAGAATCTATGTTGTTTACAAACGTTTTGGTAATGTTTTTAAGATAAAGATCTAGCCCTTCCAGAGCTCGATAGAATCGGCGTTTTACATCATACTCCAGCTCGATGGCGCTTACGGTTGCCTTCCAGTCTACTTCCTGGGCACCTCCAGGATTTGGGCCCACGTGTCCTCTGGCAAGATCTACAGCCGGAGAATTAATGCGCGCATTTTTTTCCGTATCCAACTGCATGAGGCGTCCCGCAATAGCATCTCCGAGAATAGTGGCATAGTTTTCCTCGTAGGATTGAAACTCCTGTTTGTTATGCGTTAAATTGGAGTAAATCTGGGCCACATAATAGTAATACATAAAGGTGTTAATTGCCTGGTTGAGGTCAACCTGCGATCGCGCGGCCTTGCTGAGCCCAAGCTCTTCAACTGTTAGGGCAGCACCGCCTACCCTTGTACACTCGCAGTCCTCCTCGCCTCCATACTTTTTTTGCACAATATCGGTATAAAAATCAATAATCTGTAGCAAGCGAGAGCAGGAGTCATAAAGATTTTTAAAATTAGGGTCGGTTTTAGATATCTCCTCCAAAACATTTTTAACAAGCGTAAGCTGTGTTAAGAAGGTTTCGCGTTCTTCTCGTGCGGCCGCATTGGTGTAAAAGCCGATAAGACTTAGATCAAGTGCGATGGTGCCCATATCATTAATGCGCGAAAGAGCATCTCGAAGCCTCGTTATGTTCGGCGTCAAGGCAATTTCTTTAACAAGTTTGATGCCTATTTTTTTCACATTTTCCAAAAAGTCGTTATAGGCTTGTGTGCTTTTATTCAAAAATTCCATGAGGATGTGCTTTCTATCCAGTCTTTGCGCTTCAATCCTCCTATCTAGTGGCGTTTTCTCCTCATCGCCCCCCTTTTTGGCACAACTGTTCTCAAGGATTTTGTGGCGTTCATTAAAGGTCTGTCGCAACAGGTTCACGGCTTTTTCAAACTCAGCAATGTTTTCTGCGGAGACAAGACCACTAAACCTTTTGAGGTCAAGCTCCTTGTCAAACTCCGCCCAGTTTTTGCTTTGAAGGTACTGTTCAACCTTGAGTCCTACTTTCTGGAGAGCCTTATTAATTTTATTCGCAACAGACGCAGCAATACCTAGATTACAAAGTGTGTACGAAAGTACTTTTCCAAAATTTTTGGTTCCCAAGACACTATTTGTATCATTTAAAAGTTTAATAATATCCACCTCATCCGTCTGCAGTTTATCAAGTTCCTTTTGGGTGGGAGTTAAAATATTGTCAATAAAATTCGTTAAAATGTTGATTTGCAGGTTTTGTTCATTTAAAAGTCGACGATATACTGCTTCAATCATGGTGACTGCATTAATGACTTCCTCATTGGGGGCTGCTTTGGTTACCTCCGTCACCATGCGCTCGTGAAGTTGCTTAATGGCGTCGTTTAACAGCTTGATATTTTCAAGTGTATTTTCTATACTGCCGTGTACATCAAGATACTCTGCGCGCAGTCCATGAGTTAGGGAGTTAATGTACAGAACTATTTGTCGACATATACTGGCGGCCCCTTCGGTGGTATCTATAAGCTTATCCTGACCTAAATCAATAAATTCCTGGTTAATGGCGTCTGCAATCATTTTACAGACGGTCTCCTGTTTTTCCGCATTTTTTACAAAGGTGGAACCGGCTCGAGGATCGGGCAGTTGTTTTTTGATATCTTTAAGAATATCTTCGATGGGCTGCTTTGTGTCTACTTTGAACCCTATTTTGGCAATCGCCCTGATAATTCCTTCTATAATCCGCAGCTTTGCTTTACTCGATACGGAGTCTATGTGATAATCTTTAATGTGTTGTACAGGATTTTTGTCCCCCCCGCCATTAAAATATCCTCCCCCTGAAAAAGGACGAGTTTGTCTTTGTATATGATCCTGTAACTTCGCATATATATTTGCTTCTGATGAAGGCAGTGGTCTACTAGAGGTTGAAGATCCACGGTTACCCATTATAATAAAAAAAAATAAAGATTTAAAACTACAAATATTTTGCTGTTTATAAACCCAATCATATAAGACTAACTAAAACATTAAATGTAGGTGAGATAAAAGCTTATTTTTTTTTTAAAAGTTTAATAACCATGAGTCTTACCACCTCTTTTTCTTCTTCCTTTAGAGGGGTTCCATAAATGGTTTGAATAAAATTATGTGCTCTAATAACCTTGTTAAAATCAGGTGCCTTTCCATATTGTTCAATATGTTGCACAGTCTTTTGTGCAAGCATATACAGCTTGGAGTCTTTAGGTACCTCCGATGAGGGCTCTTGCTCAAACAACGTTTCAAAGGAGGATGTGCATTCATTGGTTTCATTATCATTTTTTTCATGAATGTTCTCCGAAGATGCTGAGGATTCCGTCTCCTCTTCAAACAGCACATGCAGAATCATATTCCATTCTTCTTGAGCCTGATGTTCAGTATACCCTTGCCCTGCATATATACGAGCAGATTTCACAATATCATACTTAACAGTACTAAGCAATGTTTTTATAGCGGTCGTAACAATTCTACCGCTATTGATAATCTCAACAGAAAACCAATTATACAGGCTACCCGCATGAAACACAACTTGTGAAGATGATCTTAAATCCGTTTTGAAGATGACCTCCATTTTCATGGATATATTTAAAATAAAATCCATTCAATTTTAAAATTATAAAATAATAAGAAGATGCCCTCTAATATGAAACAGTTTTGCAAGATTTCTGTATGGCTACAGCAGCACGATCCAGATTTATTAGAAATTATCAACAACTTATGTATGCTTGGCAATTTATCCGCGGCAAAGTACAAACACGGAGTTACCTTCATTTACCCCAAACAGGCAAAGATCCGCGATGAAATAAAAAAACATGCCTACTCCAATGACCCTTCACAAGCCATAAAGACCTTAGAATCACTCATCCTTCCATTTTACATTCCCACTCCAGCGGAGTTCACCGGGGAAATCGGCTCCTACACCGGAGTGAAATTAGAGGTTGAAAAAACGGAGGCGAATAAAGTTATTTTAAAAAATGGAGAAGCGGTCCTAGTACCGGCGGCCGATTTTAAGCCCTTTCCTGATCGCCGACTAGCGGTCTGGATCATGGAGTCAGGCTCTATGCCCCTGGAGGGTCCCCCCTATAAGCGGAAAAAGGAGGGTGGGGGGAATGACCCGCCGGTTCCTAAGCATATCTCGCCGTATACTCCGCGCACGCGTATTGCCATTGAGGTGGAAAAGGCCTTTGATGACTGTATGCGTCAAAACTGGTGTAGTGTCAATAATCCCTATCTTGCCAAGTCGGTCTCCTTGCTGTCTTTCTTGTCGCTCAACCATCCCACCGAGTTTATTAAGGTACTGCCGCTTATAGACTTTGACCCCTTGGTGACCTTTTATCTACTTCTTGAGCCCTATAAAACGCATGGGGATGACTTTTTAATTCCGGAAACCATTTTATTCGGCCCTACCGGATGGAATGGTACAGATCTGTATCAAAGTGCCATGCTGGAGTTTAAAAAGTTTTTTACCCAGATTACTCGCCAAACCTTTATGGACATAGCCGATTCGGCTACTAAGGAGGTAGATGTTCCCATATGTTACTCGGATCCCGAAACCGTACATTCCTATGCCAATCACGTGCGTACTGAAATTTTGCATCACAATGCCGTCAATAAGGTTACAACACCTAACCTCGTCGTGCAGGCCTATAATGAGCTCGAGCAAACCAATACCATACGACATTACGGCCCTATTTTCCCGGAAAGTACCATCAACGCACTGCGTTTTTGGAAAAAGCTGTGGCAGGATGAACAGCGATTTGTTATCCACGGCCTGCACCGCACGTTGATGGATCAACCCACCTATGAAACCTCTGAGTTTGCAGAGATCGTTAGAAATTTACGGTTTTCGCGTCCCGGCAATAACTATATAAACGAGCTTAATATTACAAGTCCCGCTATGTACGGCGACAAGCATACCACCGGAGATATTGCGCCCAATGATAGATTTGCCATGTTGGTGGCCTTTATCAACAGTACTGACTTTTTATACACCGCGATTCCCGAGGAAAAGGTAGGGGGGAATGAAACCCAAACCAGTAGCCTTACAGACCTAGTTCCAACACGGCTACACTCTTTTTTAAATCATAATCTAAGCAAACTTAAAATCTTAAACCGCGCGCAGCAAACGGTTAGAAATATTCTTTCAAATGATTGTCTTAATCAACTGAAACATTATGTTAAACACACGGGAAAAAATGAAATACTAAAGTTACTTCAAGAATAAGTATGTTGATACCTGTGGTGTGTTTTACCTGTGGGTTTCCTATTGGAACCTACGCGGCAATTTTTGACAAGGCTCGTACCGAGTATATTAAAACCAAAATGGGCGGAACATTGCCGCAAAATATCCCATTAGATGCTTCTCTCCAGATTGAGTTAAAAGACCTCATTACAGCTCTGGGAATCCCAATGCGGGTGTGTTGTCGCACTCATTTAATTACTACGTTGGATTATCGTAAATATTATTAATATCTAAAATTGAAAAAATATTTTTAATGTTACTAGTAAAAATGACTACACACATCTTTCACGCAGATGATCTCCTACAAGCATTGCAACAAGCAAAAGCAGAAAAAAATTTTTCATCTGTATTTTCTTTAGATTGGGATAAATTACGCACAGCGAAGCGTAATACAACGGTTAAATATGTTACGGTCAATGTCATAGTAAAAGGCAAAAAAGCTCCGCTAATGTTTAACTTTCAAAATGAAAAACATGTAGGAACCATTCCTCCCAGTACCGATGAAGAGGTTATACGGATGAATGCTGAAAATCCAAAGTTTTTGGTGAAAAAACGTGACAGGGATCCCTGTTTGCAGTTCAACAAATACAAAATCTCGCCGCCATTGGAAGATGATGGTCTCACTGTTAAAAAGAATGAGCAGGGTGAAGAAATATACCCCGGCGACGAAGAAAAATCTAAGTTGTTTCAAATTATTGAACTGTTAGAAGAAGCCTTTGAAGACGCTGTGCAAAAAGGTCCTGAAGCCATGAAAACGAAACATGTTATAAAATTAATTCAAAGAAAAATTTCTAATAGCGCGGTTAAAAACGCAGACAAACCTTTGCCGAATCCTATCGCACGCATTCGTATTAAAATCAATCCCGCTACAAGTATACTAACACCAATATTGCTTGATAAAAATAAGCCCATTACTTTACAGAATGGTAAAACAAGCTTTGAAGAGTTAAAAGATGAAGACGGCGTTAAGGCCAATCCGGATAATATTCATAAGCTTATAGAATCGCATTCTATACATGATGGCATCATTAATGCTAGATCTATTTGCATCAGCAATATGGGCATTTCATTTCCGCTTTGCTTGGAAATGGGAGTTGTAAAAGTTTTTGAAAAAAATAATGGGATTGATGTGAACTCCATTTATGGCTCAGACGATATTTCAACTCTTGTTAATCAGATTGCTATTGCTTAAACAATTTGCTCAAAACAAGCTTATAAACGTTTCTTAGGTATGCGATACGTAAATCCTAATTCTTTAATAAGTTCTTTTTCAGTAGTGATTTTTAGAGGTACTAAAGTTTGATTTTTAAATAATCCATACTGATTTAGCTTATAATTCTTTTTTTTTAACGCAGCTCGAATTCTTATTAAATAAGAAACGGGACCCGTAAAATGAAGTACTGCGTATGGCTTTTCCTCGGCTAAGGCCGTAAAAAGATCAAGTTGATATGTGTTTTTTTTCCATTCAATAAAAAGTACACACTTTCGTTCTCCGCAGACTTTTACAGAAAAAGAAAGATCCTTTATGCGAATGTTGGGCAGGACGTGTTTTAAAAGTTTTTTTTCTGGAACAATAATAAGAAGATCCACGTCATTAAGCATTTTCTCTTCGCGTCTTAAGCTACCAACAGCAACGATGTTTTTTGATAAAATTTTTATAAGTTGTCCATTATATTCAAACGCAAGTCGGGAGCGTAAGTCATTTACAATTTTTTTTCCTTGAATAAGCGTTAACATTTTATATTTAATATTAAAATCTTTTCATTTTATATATTATATACGCAAAATGGCACTTGATGGTTCAAGTGGTGGAGGCTCTAATGTAGAAACATTACTTATAGTAGCAATCATTGTGGTTATTATGGCAATCATGCTTTACTATTTTTGGTGGATGCCCCGCCAGCAAAAAAAATGTAGCAAGGCTGAAGAATGCACATGTAATAACGGAAGCTGTTCCCTAAAAACAAGTTAAAACATGCAATTATATGCATGCATATAAACGCATGCATATAAACGCATACATATAAAATGCGTAAATACTATATAAAAAACTATAACATATCAATCAAGGAATCAACACTTTTATAATTTTCCGTAATATATTTTTCATCCATAATGATGTCAGAGTACATGGTCCCTATGCGAGGAACAGAGCCCATAAGGGTAGGCGCGGCAATACCGTAAATGGGATTCACGGCGGAGTCAACCGCAGCATCTGTCAAGACCTGGACTGGAGACGACAAGGCCATTCGCAACAACACGTTGGAAGGCTCTCTTGCATTAAGCCCTGCCTTTTCTAGAGAGGTAACCTGTCCCGTTCTTGTCATGAGATCTGCGTACATGAGTAAATGACGATGGTTGGGACCCTTGTCCCCCATAACCGTTCTAATTTCACTAATAATTTTTTGCCGTGCCGCTTCTATGCCGTAAAGCTCCATGGTGTCTCCTATAGAGGACGATACGATGGTGTATGGGTCGATGTTATCATCAAGCATTGCGCCAAAAATATTAGTCCCGTTTGTTTTGATGGCGTAGATATTGTCTAGTCTTACCAGTTTCCCCTGGGCATCCACACGGTGGCGCATAAGCTTAACAACATTCGCATTTTTGATGCCTGGTATTCCTCTAATCGTGCTATTTAATAGTTTATCCACCACATTTACGGCAATTTTTTCATCCGTAGCCATTCGGGTATTGGTACTGCGTCTAAAGGCGCTTTCCCGTAGGTATATGCGAATAATGATGGGAATCCCTGAGGCCGTGTTTTCCACAGAATGCATGATGTAGGTGTTGGGGTGTTTAGCTCTTAGACTATTAATAATACTTTCTAGACTAATGCTTTTTAATATCATGGTTGTTTTGTTTAATTCCAAGCGGATACACCAGTTTGCAATATCCTCTGGGGGCTGTAGTAGAGGATGGTTTTCCAGAAAATCCGTCATCCATTCCACATCACTTGCAAAATCGGGGTACATCACATTTTTTTTTGTGCTTGAATACGTTTCGTACAATAGGTGCCACTGCAATATCAACCGTTCGAACGTTATAAGCTCTATGCTGTTAGCAATTTCTTGCGCATATGTTTTATTTGTTTCCACTTCCGGGTTCTTTAGACGTAAAAGCATTTCAGAGGATTGTTCAGCCTCTACGGGCTTCGCGCTAAAGATCTCCTGGGGCCGCACAATTCCCGACTTGTTGGTTCCCCCGGCCACGGACCGGTGGTGGGAGTCCAGCATATATTGTGTCAAGGGCTCTGATACGGACTGCGCCGCCAGGATTCCCACTGCCTCACCGTAGTTAATAAGACTTTGAGTATATTGTAGCCTTATGAGGTCCAGGATGGCACTCATCTGCTCGCAGGTAATGTTTAATGTTTTAACGGTTGCCAGTTCGATGCGAATAAGCATGCGCATCAGAGAGGCAGCCCGTTTAAGATAAACGGGTATGGGCGTTTGTAGTCGTTCCTGAATGTTGTTAATAAACACGTATGGAAGATTTTTGCAAAACGTTTTGACCATCGCGTATTTTTGTAGAATACTTTTTTCGTCGAAGGGAAGCACGCCACTGGTGGAGCTCAGTAGAATGTTTTTTACGATGCTGGCCACGTTTACCGGCACCTGTCTAACATCTGTAAGCAGCTGACTGAAATTAAAATTTTCGACGTTTAGGAAGATCTGTCGATATTTATCTCTATCCTTTTTAAGGCGTGAAAATTCTTCTTCAAACAAGGGCGATTGTATCCCGGTGTACTTGAATTTGTCTTCAAGTTCCTGGTCCGACAGCATGATGGTTTCAAACCGTACGGTTTCAAGCTGGCGCGCATCAAGGCCGTCCTCTCCGTACAACTGCTGCACAAGACGCGTATCGATGGAAACCCGTCGGTAATAATCCACAATACAGGATTGAAGGCCAAAGATGGCTTTACGGTTGGCATAGCCTGTGGATGATGTCGATAATGCTTTGTTGATCAAGTCGAATCTTCCATTCATTTCCCCAAAGATAAATTCAGGGGAGGTAAGGCCCGCAATATAGCTGTTGCAGATGAACCCGTAGGCCTGCGCCTCCAGGGCAAACCTGGGGTAGTACACCAGGGTCCTACCGAAGGAAAACTGGGGTTGAATGCGTTGTGTATTAATTTCAATTTGGCCGATGCCCGCCATGATGTGAATCATATTGGGGTTTGAGCCCTTGGCGCCAGTGGCCACCATCTGAAAAAGCCCATTGGTTTCCGGATTAATGGAATTCATAATCGGCTTTAAAATTCTATCGGGAAATTTAAGCGCATTCAGCTGCAATTTTTCGTAGAAGTCATGCGTTGTCAGGCCTATAGGCGGCATGATGTCTCCATGAAGCAGCCGGTTGTTTATTTCCTCCGACTCAAGCAGCAGTTCATTGATAATTTCTTGGACCTCCTGATGTGCCTCCGGGGTTAGGAGCATGTCGGCCGTGGACACTGTGAATCCGGCGTTGCGCACGTAGTTTAGGGCGAGCTGCTGGGTCGCAAATATCATTTTCAAGGCCTGCTGCGGCCCATACCTACGCGAAATAAGGTGATAGATTCCACCGGAGGAACCCGCTCCGACGGCCTTTTTGTCAAGGACGCCTTCAATGAGTTCGCCGTTGCGTATTTGTGTAGAGATGTCCTGCTTGTTATAATGCATGTAGGGTGCATACACTTCTGAGTACCATGTGGGGGCTCGTTGATAATTGATGGGGGTCTGCCTCAGTAGCATAGATACAACCGATTTGCCATCCAGCAGGTCAGTTGGGGAGTAGTTGGCAAAACAAGGTGGGTCGGTTTGGGTTGTTTGAAACAACCCCATGGCGTGCAGCTTGTTCATCACATTTTTCCCCATGGGGGTGTTCGTGCGTGTAAGCAAAAAGCTTCCCACCGTGGAGTCCTGCACCTGCCCATTAACGGGACCCGAGCTCTTTGTGGAAATGAACCAGTTTCGCACAGAACAAAGTAGTTCGGCCTCAACGCGGCTCATGACGCTCCAGGGAACCCAGAGATTCATCTGATCCCCGTCAAAGTCCGCATTATACCAGGCACATGCGCTGACATTCATTTGAAACGTAGAAATTTTTGGGTTTTCAAGAACGACAATCCGGTGAACCCCTATGCTGCTTCGTTCGAGAGAAGGCTGGCGATTAAAAAACGCGACGTCGCCAGTGACGACGTCACGGTAAAGGATGTCTCCTACCTCCAGCCTAAAGTCTTGTTTGAGACCCTCAATGTCGTGAACGGATTGTGTTATTTGCTTATACACTCTTGAACAACCAGGGTACTGGCGCTTTCCATTTAAAAAATAGGGCATTAATCTATTAATATTATAATGTTGCACTGTTTCCGCAACTTGCAGCGTTCGTGCAAAGGAAATGGGATAGCCAACCTCGTCCAGGTGAAGGTCTGAGTTCCCGCAGATGGTGGACCGGCTGATCGACCATACCTGGCTGCCCAGTAGGGATTTACGAATTCTTCCCTCCTTGCGAGGAAGTCTTCGCATGATGGAGGGAGCAGGGCGTGCCCCCATGACGATCCCACGCTTTCCCGTGCCTCCCTGGGTTGCGGTGGTGGAAACGGAATCCAACAAAAAGTTATAGTAAAGTTGCTGTATGGTTTGCAAATTGCGGTCAATATTTAAAGGTATTTTTTGGCCGCGCACGATTTGTAGGTCCTTCGGGATCAGCAGATTCTTTCGAACCAGATACTGAATCACGTTGTTAATGTCGTGAAAGCTTTGGGGGCCTGACCCGATTCCCAATCTGATGCCAGGTCGTATGCTGATGGGGGGGATCTGAATGGCCTTAAGCACAAGTTTTTCGGGATGGGAGTTTTTACTTCGCCCCAGTTTTACAACGGTGTCGTAGGTTACGCGCGAAAAAATCTCTCTGATGATCTGCGGGTACAGTTTGTCAATCTTGCCCTGCTGATCCGCCCAAAAGGTAAAATAATCTTCCGAGTCCTTAACAATTTTGGGGTGTACTGCCTTACAGACGTAGCACTGCTTTCCTTCGGTTTGGCTTGAAGCCGCTTCAATAAGACGCTTAGGCCTAATAAGGTGCTCGTACCTCTTTAGGTCAACGATGGGAGCCCCGCAGTTGAGACATATAACCCTTAACCATCGTCGTATTTCGGCGATGAAGAGCGGCTGAAGCACCGGAGCATGCATCTGCAGTATCCCAGGGTGTCCCATACATTGCTTGCGCTGGTGTGAGCAAGTGATGCATTTATAATGGTGATCGGTGGTTCCCATTCGCGCATCATAGATACCCCCTTCGGCGGGAAGGGTGCCCTCAAATAAATTAGAAATGGTAACCTCCATAACGCCTTGCCTCTTATGATCATTGTCACCGGCAATATTGAACTGAACGGCGGCTATTTCGGCATATCCAGCCTCCATATTTTTGCTAAATACATAATAAAACTTCAAATGTTAAAAAAAAATAACATCGGTTGGCATATTTTTTTGTTAAAACCAAGTGTTAAATGATTTCTAAAACACTTATCGGTTCACGAAAACCTACCGCACGGGCCTGAAGAGGAATGCCAGTTTTGGGGGAAAGCTCGGCATATTCCACGGTAAGCTCTTTTCCATAAAGATGTTTTTTAAATAAGGCGGGCGTGAGTTTTTGAAAAAGAGCATAACGATCCGCGTACGTCAAATGCTTAGGAGTGACTACAAACCGCTTTTTGTTTGGCAATTCGCAAACCCATAAAATGGCGCCTAAGTCCTTTCCCTTTTTTCCCTGAGTATAGTCCACTAAAATAAATTCAGCGTCTAGCAGCGGTTTCAGCTTGGCAAGATGCGCTGAGTGGTAGTTGTTGTATCCCGGCTCATAGGGCCCATTGGCATTGCGTACGATGGCTCCCTCGTAGCCCTCCTTAATAAACTGCGCCTTAAGCCTAAGGGCCTCATCCACATTCTTCACGCTAAAATTTTCAACTTGGTGGATAAAGGTAAGATCTTCCTTCTGTTTAAAAATATTTGTTAATAGCTGTTGTCTCTTGTTGGAAGGCATTTGAAGCTGATCACTCCAAAAACAGTCAAACACGTAAAAGTGCAGCTCGGAGGAATCTGTCTTCGCATTCGCCTGCCCCGCGATCCATTGCAGAGGTTTGCGGTGTAAATAAAGCTCACCATCCAAATATACTCTCACGTCTATAAATAAATAAAGCTGTTTGAGCTCTTTTTTAATATTGTCAAGACCTAAAAATTCCTTTTTCGTGCGCGAATACAAGAGAATGCTACCATCGCCCTGCTGGCAGGCCACAGCTCGAACGCCATTACGCTTGCGCTGCACGATGGGATCTGTTTCTTCTTCAAAAAATGTCTTAGGAATTATATTAAAATATTTTACCAGCATAGGGGGGATAATTCCTCTATTTGTGTGGGCTCCCCGCTTTTGTCTGGCATGGCGATTATATTTACTAAGGGCGTCCTTGAATGCCTGATGGACTACCGTTGTGGCATTTTTTTTACCCAAGTTTTTTCCCTCGGTAACACGTGTCATTTTTGATATCCGCACCGCCCCTTCTTCCACAAAAAATTTTGTGAAAATTTCAGCAACGGCGTCTTTTACATCTGTGGAAAACATCTCATCTGTGATGGGAATGATCGTGTTGTGCTGCACCACTTGCACACAAATAATCCATGAGGCCTTTTTTCCGCTTTTCGTTTCAGACTCAATCGGAGGAAAACAAAAAATGTTGTTTGAATATTGCCCAGGAAATTGATTTAGCATGGTTTTAACAATAAAATAAGCCTATCAATTTTTTTATAATTTGAATAGTTATTCCAAATTCAATATGGCTTCTTTAGATAATTTAGTGGCACGATATCAGAGGTGCTTTAATGACCAGTCTCTTAAAAATAGTACTATTGAACTTGAAATACGTTTTCAACAGATAAATTTTTTATTATTCAAAACCGTATATGAGGCACTTGTGGCACAAGAGATCCCTAGCACCATCTCCCACAGCATCCGCTGCATCAAAAAAGTTCACCATGAAAACCACTGCCGGGAAAAAATTTTGCCGTCGGAAAATCTTTACTTCAAAAAACAGCCTCTCATGTTTTTTAAGTTTTCAGAGCCTGCATCTCTGGGCTGTAAGGTCTCGCTGGCCATCGAGCAGCCCATTCGTAAATTTATCTTGGACTCCTCCATTCTCGTTCGGCTCAAAAATCGTACGACCTTTCGGGTATCTGAACTTTGGAAAATAGAGCTTACCATTGTAAAGCAGCTGATGGGAAGCGAGGTCTCTGCAAAACTTGCCGCTTTCAAAACGCTTCTGTTTGACACCCCAGAGCAACAAACGACAAAAAATATGATGACGTTAATAAACCCAGATGACGAATATCTTTACGAAATAGAAATAGAGTATACAGGAAAGCCCGAATCCCTAACGGCGGCAGATGTTATAAAAATTAAAAACACGGTGTTGACACTTATTTCTCCAAACCATTTAATGCTAACAGCCTACCACCAGGCCATTGAATTCATTGCCTCCCATATACTGTCCTCAGAAATCCTTCTTGCTCGTATTAAGAGCGGGAAGTGGGGGCTTAAACGCCTCCTCCCCCAGGTGAAATCCATGACCAAAGCGGATTACATGAAATTTTATCCGCCCGTTGGCTACTATGTAACGGACAAAGCAGATGGAATTAGAGGCATCGCCGTCATTCAGGACACGCAAATTTATGTGGTTGCAGACCAGTTATACAGCCTAGGTACCACCGGCATTGAACCCCTTAAACCAACCATTTTGGACGGTGAATTTATGCCTGAAAAAAAAGAATTTTATGGGTTTGACGTCATCATGTATGAGGGCAATCTATTGACGCAACAGGGGTTTGAAACAAGAATTGAGTCTTTAAGCAAGGGCATTAAAGTCTTACAAGCGTTTAACATAAAAGCAGAAATGAAGCCCTTTATTTCGCTAACAAGTGCAGATCCCAACGTGCTCCTCAAAAACTTTGAAAGCATTTTTAAGAAAAAAACTCGCCCATATTCTATTGATGGCATCATTTTAGTAGAACCTGGCAATTCTTATCTAAATACAAACACCTTTAAGTGGAAGCCCACCTGGGATAACACATTAGACTTTTTGGTGCGAAAATGTCCGGAGAGTTTAAACGTACCAGAGTACGCGCCCAAAAAAGGGTTTTCCCTGCATCTACTATTTGTAGGCATCTCCGGAGAGCTTTTTAAAAAATTAGCGCTAAATTGGTGTCCAGGATATACGAAACTATTCCCCGTTACACAGCGCAACCAAAACTACTTTCCAGTACAGTTCCAGCCATCGGATTTTCCATTGGCATTTCTTTATTACCACCCAGATACCTCGTCATTTTCTAATATAGATGGAAAGGTCCTTGAAATGCGTTGTCTTAAGAGAGAAATCAATCACGTCAGCTGGGAAATTGTAAAAATCCGGGAGGATAGGCAGCAGGATCTTAAAACCGGCGGGTATTTTGGCAATGATTTCAAAACAGCCGAACTCACATGGCTTAACTATATGGATCCCTTTTCCTTTGAGGAGCTGGCAAAGGGCCCTTCTGGAATGTACTTCGCCGGTGCCAAAACCGGCATATACCGCGCTCAAACAGCACTTATTTCCTTTATTAAACAAGAAATCATCCAAAAAATAAGTCACCAATCCTGGGTTATCGATCTTGGAATAGGAAAAGGGCAGGACCTAGGACGTTACCTGGACGCAGGGATAAGGCATCTTGTTGGGATCGATAAGGATCAAACCGCGCTTGCGGAGCTTGTTTATCGAAAATTTTCGCATGCTACGACCCGACAGCACAAGCACGCTACCAACATTTACGTGTTGCATCAAGACCTCGCAGAGCCTGCGAAAGAAATCAGCGAAAAGGTACACCAAATTTACGGGTTTCCCAAGGAGGGAGCTTCTTCCATTGTTAGCAACCTGTTTATTCACTATCTTATGAAAAACACGCAGCAGGTGGAAAACCTGGCCGTTCTGTGCCATAAGCTTCTTCAGCCGGGGGGAATGGTGTGGTTTACCACCATGTTGGGAGAACAGGTCTTAGAATTACTTCATGAAAATAGAATAGAGCTCAATGAAGTATGGGAGGCTCGTGAAAACGAAGTGGTCAAATTTGCTATTAAACGTCTCTTTAAAGAGGATATATTACAGGAAACTGGGCAAGAAATTGGAGTCCTGTTACCCTTCAGCAATGGCGACTTCTACAATGAATATCTTGTGAACACAGCGTTTTTAATTAAAATATTTAAACATCACGGCTTTTCCCTAGTTCAAAAGCAGTCCTTTAAGGACTGGATTCCAGAATTTCAAAACTTTAGTAAAAGTTTGTATAAAATTCTTACAGAAGCCGATAAAACTTGGACAAGCCTTTTTGGGTTTATTTGTCTGCGCAAAAATTAAATATTTTTTCATAAGAAGTACTACCCAGGTTTTAAAGAAATAGCTAAAAATATCATATGGATACTGCCATGCAGCTTAAAACGTCTATTGGTTTAATTACATGTCGTATGAACACCCAAAATAACCAAATAGAAACTATTCTGGTTCAAAAACGTTACAGCCTTGCTTTTTCAGAATTTATTCATTGTCATTACTCTATAAATGCTAATCAAGGTCATCTGATTAAAATGTTTAATAACATGACAATTAATGAACGACTGCTTGTCAAAACACTGGATTTTGACCGCATGTGGTATCATATTTGGATTGAAACTCCAGTCTACGAACTATACCACAAAAAATACCAAAAATTTAGGAAAAATTGGCTTCTCCCGGATAATGGGAAAAAGCTTATTTCATTAATCAACCAAGCAAAGGGCTCAGGAACACTTCTATGGGAAATCCCTAAGGGTAAGCCGAAGGAAGACGAGTCGGACCTTACCTGTGCCATACGGGAGTTTGAAGAAGAAACCGGGATTACCCGCGAATATTACCAGATTCTCCCAGAGTTTAAAAAATCTATGTCATACTTTGACGGTAAAACAGAATATAAGCATATCTACTTCCTTGCAATGTTATGTAAGTCGTTGGAGGAACCCAATATGAATCTTTCTTTACAATACGAAAACCGAATTGCCGAAATTTCTAAAATTTCTTGGCAAAATATGGAGGCTGTACGTTTTATTAGCAAACGCCAGTCATTAAACCTGGAGCCTATCATCGGGCCTGCATTTAATTTTATTAAAAACTATTTACGATACAAGCACTAGGATGCCGCATTAAAATGCCACATAAGGTAATACACTAGGAATGTCGCACACGCACAAGAATACAACGTCGCCGGAGATTTATTATCTAGTACACGTTTTATGTATGTACAATCCGCCTTCATTTAATATATTGAGCGGATGTACTATGTATTTATTTTAACAAAAAACATTATTTTTTTTTAATCTTCATCATCTGTTTTTATAAACTCAGTAATATCAAAAGTAGCTTGTGGGGTTTCAGAGGGTTCACCTTGGTTATCCTCCGTGAGGATAACATGTTCTTCAGGTTCGTCGTCACTGGAGAACCCATCATTTAATTCCTCTTCACTCAACATCTGTAAAAAATCTTCCAAGCTTTCGCTATCGTTAAAATCCTCATCATCCATAAGAATAATGGTACCTTCCTCATCGTTTCCTCCTTGTTTCGTGTCTAAATAGGCCTGCATGGCATTTGCAAAAGTATCAAAATAGGCTGAGTCAGATTGCTGTTCCAAAATATGGCCTTGCGTATTAAATGTGGTTGCATCGTTGTTAAATGCTTGCAAATACAGTAAGGGATTTATATCCATTATTATTAAGCAAAAAAAATTTAAATTATTTTTCGACCGATGTTAGGTAAAATTAAACAATTGCTATAGGTGTTAAGCAATGTTTATTGATTTTAAGTACTCAACAACCATGATGTAAATACTATACAGCACTTTTGGATTTTTAATCAAATCCAGATTAATACTAACTTCTTTTGTGATACAGTTCGTAATAATAGTATCCTGCTCATCGTTTTGTAAGATTTCTTTTAATATATTTTTTTTTACCGGGATACTAAGCAATTGATTATTTTCTTTTAAAAACTCCTTTTGATATTCAATCGTCTTATTCATTGAATATTTGTATATAACTATAATTACAAATGTTCAATGAATTGTTATTCATGTCGGGAGATGGCTATTTAAAAATCATGTCCTATTTTTCTTTGCTCAATAAGCATCCAAATATTTTCATGGCGTTTTATTAATTGTTCATTATTGAACGTATCACAAAGATCATTTATAAATTGCAGATAGTTTATTATTTCTTTCAAGAGAGTAACAAACATTACTTCAGCAGAACATATAATAGGTAATTCAGTGGCGTTAAAAGAATTTTGATCTTGTTGATACGCCAATGGCGAGGACTTAAGGAGATTTGGGGGTCTTGCCCAAAACCCTAGGCTGCTGTTCTTGTTTTTTAGGGCGTCATAAAGAAATGAAAGCACATTGCAAGGCTTAAGCCGCGACATCTCCTTCCCCTTGGGCCCTTTCCATATTTTTAGATCTAAGATCTCATCCGAGCTTATAGAGTAGGTATAGTAAAGTTTTTCAAAAAAGCATATCTGCTTGAAGTCTTTTTTAGAACGACTTTCAAGAAGCATTTCTATAATGTTAACAAGTTTTGTTAGGTTTAAGGCCTGTTCCTGTGTAAGCTCCTCTTGCACGTGATAGACTGAAAAAGTGTGCTTAGGAATGAAAATACTCCCCGTGGCACTGGCCTGTTGTCTGCCAGGTATATAGTACACGCTGCTGTTAGCAAGCTGTACCGGCACAATTTGCCCCACTTCTGCAACATTATTTTGCGATTCGGACGAGGGTATGACAATAGTTACGGGTTCAGTCAATAGGCTTTCGCCGAGAATAATATTACTGTCATTTTTAATAATTTTAACGGCCGCTATTAAATCAAAGGCATTTAAGTAAGAAACAACAGCAGAAAATCTTACATGCATATATCCTCTTCCGCTATTATTCGTACGCATAATAAAACAAGGGGAGCGTTGTATAACGCCAGTAATATTAAGAATAAAACTGTTTTTGAAACACTTACCCACATAAATGTTTTCAAGCTCCTTCAAAAGATGAGCCTCCACATTTGTACAAAAATTGGTAGGATCATCAATATTCAACGTTGTCTCAAAAATTTTTTGGTCGATCATATCTATAATATATTCTGTCTATTTCAATTTAAATAATATACGAATAAATAACGAGATTATTTTATTAAATAAGCAATGGTGTATACACTTTGTATTTACTTTGAGATATACTTTGTGTATCACAACGTGCCCTAAGATGTGTGCACAAGTGACGGCATTTTGTCGTTAAAAAGGTAAAACCAGCGGATTCCATCCTGCATTCCATTTGGTTGATTACGAGCCTCCATTTCTTTTTGCAAAAGGTTATTGCGAATGAGTAAGCAGAGCTTGATGGCACTAATCTTTGTAAGGTTTAAACTTATGCCCAATTGGTCAGCAATTTTTTGTTGCTCCTCCCGTCCGCGTGTTTCGCATACGGCTCCCCGGTTTAGCATGCGAATATCAGTAATCTCATTCTTTTTTAAAACCTGGATAGGTGGGCGGATTTTAAATTTAAGGGCCTTTCCCTTGCTTTCCATATAGCCTATGACGATGTCGTTTTCTTTTCGTTTAACATTAATATTAAGCATATAAAGCGGAATTTCATGCCAGGTTTTATCTTCTCGCGAGGTAATAAGTCGCACGGAGTCCTCCGTGGCATAGCCCACTAGAGTGTTGTCATCCCCAGGCACGTGGCTTATAATTTTAAAAATGTCCGGAAATGGCTGAATATCTTTTTTTGAAAAAGCGATGAAAAACTTTTTATAAACCTCGACAAGGGCCCCCATACCTGCAAGATTATCTATAATAAGTGCTTCTAGCATCGTATAGTGAAATGAAGCGGGGTAGTGGATGAGTACCTGCTCCATTGGCTCATCCTGAAAATCCTTCTGAAACTTTTCATACAATACTTGAAAGGGTTCTTTGGTCTGCGAGTGTTCGAGGTATTTGGTAATACGGATGCTGTGCATCGCGGGAGGCTGAAAATCCCGAATATATGTTTCAATATCTAATACCGGTTCCTTTTTATGGTTAAGCACCGCAGCGACGTACAAATGCTCAGGCTTTGCCGGCACATGCATAATGGTGCAAAGACGATTCTGTATCCATAATTCCTTGCACTGGTTTTTTGAGTAGCATAGAGAAATGAGCGCCAGCGCGAAGTTGTCCTCTGAGAAGAGTTTATTATCGATGGTAATTCCCTGTATGAGCTTGGGAGTGGAAACAGCCTTCCATAGCTCGGAGTACGTCCACACGGGGCGTGCCATAAACAAAGATATAATAATATTAGAAATTGTTTTTACCTCTTGCTCCCCGTATCCATAGGCCTCAAAGGTATTGAGGACGGTGGCTCCGACGTTTGCCGGCGTGATGGATGGACTAAGGGGCAGACTTTCCAACATAGGCTTATCAATCTTAATCTGGTTGGTGAACCCATCAATGGCGTGCTTTCGCAGCGCCTTATCCCCCTCCTGTATTAAAATGTATTCTTTTAATTTTTGTGCGTACTTAGCGAGCTCTGGCCCTCCATCGGGTGTTGTCGATACGTACAAATAAATTGTCACGTTGCGCTCACTGGGGGGGAGCTCCATGTGTGAATTTTTTCGCACCACCCTCCCAAATACCTGAATAAGCCGGGGAATATCAAGGGGCAATGACATAATCATCTCGTACCGCACGGCCTGAAAGTTCAAACCCTCCACAATCACCTTGGACCCGATGAGAATACGCAGCTGGTGGCCTTCCAGGTTGGACGAGGCGTTAAAAAGAGCCAGGCTTCGTTCGCGTACAGCGGGCTCTATTTCGCTGTGCAGAATGGTGAACCGTACTGGAATAAACTGATGGTCGCTATGTGTGTGCTCATCGCGAATCGCGGCGCAGATGGAGCAGCGGGTCGTTCCCACAGGGGACGAAACTTCATTTAAAATGCCATTACTTTGTAAAATTTCTTGCAAGATAAGAACCCCCGACATGCGGACCCGATTGTGGTAAATTAAAATTTTCCCCCGGCCTTGCCGAATAATGGAAAGAATGTCTTTCATCATTTGAGTGTATTTTCCGCTATAAAAGGCCAATCCCGAGATGTGCGTTGGTGGCTGCAGCGACAAAAAGCTGCCACTCACATTAAAGGGGGCTCTACGCGAAGGCTCAATAATCTGTACCCCGTTTTCCAGAAGCCAGTCTGTGCTTGCCATAGAAAGGGCGGTGGGGGTTTCCGTCGAGTTAAACAGGCCGTAAGCCTTGGGTTCCGTTTGTTTTGAAAATTTTGGGTTGGGAAACACCATGTCATAAATGCTGTACGCATTACTCGAGATTTTAGGGTCAGGGCCCAGCTGTTTAAGCGTTTCAAGCTGATACTCAGACATGGGGCATTCGATGAAATGTAAGTACGGCAATGTTTCGTCTTTATAGGACAACATCTTTCCGGCAAATATTCTTTCGGGGTAAAAATTGGTGTTGGTATCCAACAAAAAAGATACCCTTCCGGTGCTCAGTCTTTCCACAAGAGCTAGGGCGTCCTTTTTCCATTTAACGGAATGCCCACTGCTGTCAAACAGTTGCTGGCGCTGGAGGGGCTGGCCGTTGGGCAGCTCATGCCGCGGAACCAAAAGGTTTAACAGGTCGACGTATTCCATGACACTCCCGGTTACGGGCGTTGCCGACATGAAGACGGCCCTGGGGGCCTGGTGAGGTGGAAAGGCATCCAGGACATACTGTAAAGCGATGCCATAATTATTTCGTTCCTGGATATTGTACACGTTGTGTATTTCATCCGCAATGAGCAGTCCTCCCCTAAGTTGCTCCATGATTTTTTGATTCACCCGGATGAGGCCGTTTGTCTCGGCCTCGCTAATTTTTTGCACGAACTGAGATATATCGTTCTCATTCAATGTATCTTCTGCTTCGTCAGAACGATGAAACAGAGAAAGCACATCAAAGTTTTTCTCTTCACCCTTACTCGTAATATTGAAAAGCTTGGATGCAAATTCCTTATAGCCGTAAAACTGAAAAAAGCCTCCGCGGTTTCTATCGGTTAAACGGCGCTTTAACGTACTAACGAACCCATTTAGATGCCGTGATTCGACCGACGTGGTGCTGCCAGACTGCTTTGCAATGTGAAGAAGCCGGTGTAGCTCAGCGACCTCCTTGTAAGAAACAAATCCCAGCTCAGGACGTCTTAGCATTTCTGTTTGAATGATGGCGCGTGTAAAGCCTACCACAAAAATCCAGGGCGCATTTTCAATAAAATTCATGTAGTGGTTCATAAATTGACGCGCGATGGCAATCGCGGCAATGCTTTTTCCCGTCCCGGTCTGCCAGTTTAATAAAAGACGCGAGTAGGGCGTGTTGGGATTTTGAAAGTTTTGGACGAAAAGCTGGGCATTATGCAATTGGAGACCCTTGATGGAAGGAAAGGGCGACGCGTAGGGGTCACACGGAAAAAACGCTCGCCCCCCCTTCTCGCAGCCAGGCCCACCGATCTGGACAAAATGAGCCCGCAGATCACGAATGAGCTCTTTTTGGTCGACAGGAGGGGAAATCAACGATTTAAACTCCTTTCTTCGCGCCAACTGCTGCAAAAAGTCTGCGGCATCCAATTCGGGATACGCCATATTATCATAAAAAAAATAAACCTTTTTATGAAAACTTTTATGTGATTCTGTATTGCAATTGTTTTTTATGAATACTGTAAATAAGCGTATCAACTTGTTTTTCTAACGAAGAGGCGTTATTCTTTTTTTCTGGATATAAAATAATAATAAGTATAATAATTAAGACTAAACAGCAGGCAATCACTATCAAACTCATATTATACTTACTTTTTTATAAAAAGTATTATATCTTATGAATGCGCAAGTTCAGCTAATTGTTCGTCGCTTGGAATGTGGGACTGCAGGGAGGTGGAGTTTTTCCTTTTTCTAAAGAATACCGGGAAATGGTGGTGAGGCTCAGGTTGTTGTACATAGTAGCTAGGAGGAGGTTTAGGTATGCTCGACTTGCAGTCAATAGTCCGGTTATAGTAAACGATGGCAACGATGATAAGAATAATAATGAGCAAAATCAAAATGCCCAGGAGAATCGCAGTTGTTCCGGGATATTTGGCGATTGTATGGGCTAAAAGGCCTTGGGTGCTTTGTTTAATTCCCTCGCGGGTTGACAGGTTATGAGAAAGCAGTGGAGACGTTTCAGTGTCCATTTATTACAATTGAACAGTTATATTAATCTCAAATAAAATATAACACAAAATTAATTATGGCCATGCAAAAGTTATTTACGTATATTTACGAGTTTATTGAATATCGTAAGATGGTGCTGTTGGAAGAAAAGGTACCATATGATAAGTTTGTTCAAATGGTACTTAATACAGGATTTTTTCGTATTAACGCGGAGACGCTGAATCACGGAATCGTATCCGTGTTTATCTTTGGAGCAAATGGCAAGTACGTTCACCACGGAGGCGACATGAGAACGCTTTTAACGAATACGCTTAATGAAAAAAAACATTATGAAGAATTAATTTTAATCGTTGATAAGCCCGTTTTAAGCAAAAAAAATATTTTAGATATAATCGTCGAGCAGCGCGCTGCAAATCCCACGATTGTAATAAACATATATCCCTACCACCTGTTCTGCATTAACATTCCCAAGGTGAGTGCCATTCCTAAACATAAACTAATTACTCAGGAGGAGGCGCAGGAGTTTTTAGGTCGCGAATATCTGCAACCGCAGGACCTCATGCAAATTAGCGCGTCAGACCCCCCGGTGGTCTGGCTGGGAGGAAGACCGGGAGACTTTGTGCAAATTGAGCGGCCCTCAGAGACAGCTATGCACGCTGTTGTTATCCGCTTTATCACCAAGTCCAAAATTTGAGTCCCGTGTTTAAAGATGACAGACAGCTAAGTAAGCATATCTGTAAAATTGTCGATGTCCTCTGTGGATAGAGCGCTTTCCTCTGAGCAGCAAATTTTTTCATACATCTCCATGGGGGATGGCGAGGCTTTAATAGTATGTAGGTCACGTAAGAACTGTTGTATGATGGGATATTTGTCTTTTAAAAACTGGGGATGTTTCATAACTGGAATTATTTGAAAGATAAAGACCTTCCATCCAAAGTAGCCAACCACATTTGGCATTTCGGGACACGCGGTTTCATAAGGCATAGAATAGTGAATAGTGTACTGATCTTTTTGATACAGCGTTTCAAGTAGTTGGCGAAATGTTTCCGCGTCGAGCGTGCCAAAATCTTGAGGAGCCTCGGTGTGCTCCTGTGTAGAGCAGATCGTGATGATTCCCCAGGCAAGCGGGAGCATGGACTCTGGAGGGTGGATATCCGTATTGGTCTCATTATTCGATCCCAGCTGATGAATGCCGCACACGCGAAACATGGCCTCGACGTAGATGCCCATAGAGATAGGCGGCGAAAGGGCAAGACCGGATTGTATTTGCGGCATATAGTAGGAGGGCACCGAGTTTTTTATTTTTCGGTTGAATGGGGACTTTATTTCTACCAGCACGGGGATGCGTTTCGTGGCCTCATAGCGTACGTTGTTAAAAATTGTTTTGATTTCCCAGGACTGTTGAGTGTATCCCAGCGTTAGGTGACAAAACCCATCGGGGCTATTACTATGTCCGGGGTATCCCAAATAGGTCCCATCAATATGAATATTGTCACCTATGACGGTGGTTTGGCAGAACAACTCAAGCAGATCTTTACTAACACGCTCAAAAAGGGTTCCCCAGCTACAAGCAGCGCGGTTCAAATTCTTCTTAAAAAGATTTGCTTTTTCCGCCAAGGTTATATAATAGCTTTTGTAAGGGTTTAAACCTAAAACGCTGGCAAGGTCAGAGCCACCCACCTGAGTGCGACGAATAGCATGCCAGGCATCGGAGCGCTGCTGAGGAGAGTCTTTAAACAGGCGTACAAAGGTTTCCATTATACTTGTTTTAACAGGAATTCAATATAAAAAGTCAACACAGTTTGCAATTTTTCCAATCTCAAGATATAGCCATACATTTTTTTTTCCAATTGGCGAATATGTTTAAGCTCATGTGTTTCAATATTAGCATCCGGAAATTTAAATGCATAAAGATGTTCAAAGGCCTGATTTATACACGTATCAAAGGATCTGTGGTATGTTATTAGCTTCAGCATGTGTGCCAGATCTTCAAGATGGTCTAAATTTATACGGTTTTCCACGTGGTGGATCATGTCTGCCACATCTTGAGCCCCCATCCAGGGGATCACAAGGTACTCCCCCTTAAAGATGATTCGTCGTTTTTTTAAAAAATCATGAAAACGTTTTAAAGCTTCAAGAAAGGGGCAGTTGGGCTTTGACCCCAAAATGCTGACGACGATATCCTCGGGCATGATGTATTCGCAGTGAGGATAGTAGTTTACGGACTCTAATTCAGCGGCCCGCCGTTTTATTTCGTATCTTGCCCAGTTATTCAGAGAGTACTCCACGCCTCCGACCACAACAGACATCCTATCTATTAAAAAATAACAATAAAAACCTTATGAAATCTATGTATAGTGGCCGCTAAAATGTCTATATTAGAAAAAATTACGTCAAGTCCCTCTGAATGCGCAGAGCATCTTACAAACAAAGATAGCTGTTTAAGTAAAAAAATACAAAAAGAGCTCACCTCTTTTTTGGAAAAAAAAGAGACACTCGGTTGCGATTCGGAGTCCTGCGTAATTACCCACCCCGCCGTGAAGGCCTATGCGCAACAAAAGGGACTGGACCTCTCCAAAGAACTGGAGACTCGGTTTAAAGCGCCAGGACCCAGAAACAACACGGGTCTTCTTACAAACTTCAATATTGATGAAACGCTGCAGAGGTGGGCCATAAAATACACCAAGTTTTTCAACTGTCCTTTTTCCATGATGGACTTTGAGAGGGTCCATTATAAATTTAATCAAGTGGATATGGTAAAGGTATATAAGGGAGAAGAGCTACAATATGTAGAAGGCAAAGTGGTCAAGCGTCCTTGTAACACCTTCGGATGCGTTTTAAACACGGACTTTTCAACGGGCACTGGAAAACACTGGGTAGCCATCTTTGTGGATATGCGGGGCGACTGCTGGAGCATCGAATATTTTAATTCGACGGGAAATTCTCCTCCAGGTCCCGTTATTCGTTGGATGGAACGGGTCAAACAGCAGCTATTAAAAATACACCACACCGTGAAAACGCTTGCAGTTACCAACATTCGTCACCAACGGTCGCAGACCGAGTGCGGCCCCTACAGCCTGTTTTACATCAGGGCACGCCTCGACAACGTGTCATACGCCCATTTTATATCCGCTAGGATTACCGACGAAGACATGTATAAGTTTAGAACCCATCTGTTTCGCATCGCATAAACTAATAAAGTTTGAATTCTTTATAGGAATAAAAATGGAAGCGTTTGAAATCAGCGATTTCAAAGAGCATGCGAAGAAAAAAAGCATGTGGGCTGGCGCCCTCAACAAAGTCACTATTTCGGGTCTTATGGGGGTCTTTACCGAAGATGAGGACCTTATGGCGTTACCCATTCACAGAGACCACTGCCCCGCTTTGTTAAAAATTTTTGACGAGATCATCGTAAATGCCACGGATCATGAAAGAGCTTGCCATAACAAAACAAAAAAGGTAACTTACATTAAAATTTCGTTTGATAAAGGTGTGTTTTCTTGCGAAAACGATGGCCCGGGAATCCCCATTGCAAAGCATGAGCAAGCCAGTCTTATCGCCAAGCGCGATGTGTATGTTCCCGAGGTGGCTTCATGTCACTTTTTAGCCGGAACGAACATCAATAAGGCCAAGGACTGTATCAAGGGGGGAACCAACGGCGTCGGGCTGAAGCTCGCCATGGTGCATTCGCAGTGGGCCATTCTTACCACCGCCGACGGCGCGCAAAAGTATGTTCAACATATCAACCAACGCCTAGATATCATTGAGCCTCCTACCATTACACCCTCCAGGGAAATGTTTACACGTATCGAGCTCATGCCCGTATACCAGGAACTAGGGTACGCGGAGCCTCTGTCTGAAACAGAGCAGGCGGATCTTTCCGCCTGGATTTACCTTCGCGCCTGCCAATGCGCGGCCTACGTGGGAAAAGGCACCACCATTTATTACAATGATAAGCCTTGCCGCACGGGCTCTGTGATGGCGCTAGCCAAAATGTACACCCTGTTGAGCGCGCCTAATAGCACGATACATACGGCGACCATTAAGGCCGACGCAAAGCCCTATAGCCTGCACCCCCTGCAGGTTGCGGCGGTCGTGTCCCCCAAGTTTAAAAAATTTGAACACGTGTCCGTTATCAACGGGGTAAATTGCGTAAAAGGAGAACATGTCACCTTTTTGAAAAAGACTATTAATGAAATGGTCGTTAAAAAATTTCAACAAACGATTAAAGATAAAAACCGCAAAACAACATTACGAGACAGCTGTTCAAACATCTTTATCGTTATAGTGGGTTCCATTCCAGGAATAGAATGGACCGGCCAGCGGAAGGATGAACTTAGCATCGCGGAAAATGTTTTTAAAACGCATTACTCCATTCCTTCTAGTTTTTTAACAAGTATGACAAAGTCTATCGTGGATATTCTTCTGCAATCCATTTCTAAAAAAGATAACCATAAACAGGTCGACGTAGACAAATATACGCGTGCCCGCAATGCGGGAGGAAAAAGGGCGCAGGACTGCATGCTACTCGCGGCGGAAGGGGATAGCGCACTTTCCCTGCTGCGCACGGGACTAACCCTGGGAAAGTCCAACCCAAGCGGGCCCTCCTTTGACTTCTGCGGCATGATCTCCCTGGGAGGAGTCATCATGAATGCCTGCAAAAAGGTGACAAACATTACAACGGACTCTGGAGAAACCATTATGGTGCGCAACGAACAGCTTACCAATAATAAAGTGTTGCAGGGAATCGTGCAGGTATTGGGTCTAGACTTCAACTGCCATTACAAAACACAGGAAGAGCGAGCAAAGCTGAGATACGGCTGCATTGTTGCGTGCGTTGATCAAGATCTGGATGGGTGTGGAAAAATCCTTGGACTGCTGCTGGCCTACTTTCACCTGTTTTGGCCTCAGCTTATTATCCATGGTTTCGTAAAACGACTGCTTACCCCGCTGATACGTGTGTATGAAAAGGGTAAGACCATGCCCGTGGAATTTTACTATGAACAAGAGTTTGATGCCTGGGCAAAAAAGCAGACCAGCTTAGCCAACCATACCGTAAAATATTACAAGGGATTGGCGGCGCATGACACCCATGAAGTAAAAAGCATGTTCAAACATTTTGACAACATGGTGTACACGTTTACCCTGGATGACTCAGCAAAGGAGTTGTTTCATATTTATTTTGGCGGGGAGTCGGAGTTGCGAAAAAGAGAGCTTTGCACCGGCGTGGTGCCGCTCACCGAAACCCAGACGCAGTCCATTCATAGTGTCCGACGAATTCCTTGCAGCCTGCATCTGCAAGTAGATACCAAGGCTTACAAGCTGGATGCCATCGAGCGGCAGATTCCCAACTTCTTAGACGGGATGACGCGGGCGCGGCGCAAAATTTTAGCCGGGGGGGTGAAATGCTTCGCCTCCAACAACCGTGAACGAAAGGTTTTTCAGTTCGGGGGCTACGTTGCAGATCACATGTTTTATCACCATGGCGACATGTCGTTAAACACAAGTATTATAAAAGCCGCCCAGTATTACCCAGGCTCCTCCCACCTCTATCCGGTATTCATAGGCATAGGAAGTTTTGGCTCCAGGCACCTGGGAGGAAAGGATGCAGGATCCCCAAGATACATCAGTGTGCAGCTTGCGTCTGAATTTATTAAAACAATGTTCCCCGCGGAGGACTCATGGCTTCTCCCCTACGTCTTTGAGGACGGCCAGCGGGCGGAACCAGAGTACTACGTGCCTGTGTTGCCGCTTGCTATTATGGAGTACGGCGCCAACCCATCGGAGGGCTGGAAGTACACCACTTGGGCCCGGCAACTGGAAGACATTTTGGCCTTGGTGAGGGCCTACGTCGACAAAGACAACCCAAAACACGAGCTACTGCACTATGCAATAAAACATAAGATTACTATACTCCCGCTGCGGCCCTCCAATTACAATTTCAAGGGCCATTTGAAGCGGTTTGGCCAATACTACTACAGCTACGGCACGTACGTCATCTCAGAGCAGCGAAATATAATTACTATTACGGAGCTTCCTCTGCGTGTTCCTACGGTTGCATACATCGAAAGTATAAAAAAATCGAGTAACCGCATGACATTTATTGAAGAAATCATCGACTACAGTAGTTCAGAAACTATTGAAATTCTGGTGAAATTAAAGCCAAATAGTCTTAACCGTATCGTGGAAGAATTTAAGGAGACTGAAGAGCAAGATTCCATAGAAAATTTTCTGCGCCTGCGCAATTGTTTACATTCACATCTAAACTTTGTAAAACCTAAAGGTGGCATTATCGAGTTTAACACGTATTATGAAATTTTGTATGCGTGGCTACCTTACAGGCGTGAGCTTTACCAAAAGCGTCTTATGCGTGAGCACGCGGTGCTTAAGCTGCGCATTATCATGGAAACTGCTATTGTACGCTACATCAATGAGTCTGCAGAGCTAAATCTTTCCCATTATGAGGATGAAAAGGAGGCAAGCCGCATTCTAAGCGAGCATGGATTTCCCCCGCTGAACCACACGCTGATCATTTCCCCTGAGTTTGCCTCTATAGAGGAACTCAATCAAAAAGCACTGCAGGGCTGTTATACCTATATACTATCTTTGCAGGCTCGAGAATTGCTTATCGCAGCCAAAACTCGTCGGGTGGAAAAAATAAAAAAAATGCAAGCTCGTCTTGATAAGGTTGAGCAGCTTTTGCAAGAGTCTCCCTTTCCCGGCGCCAGCGTATGGCTGGAGGAAATTGATGCGGTGGAAAAGGCTATTATAAAAGGAAGAAATACTCAGTGGAAATTTCATTAAACGCTACCGGTTTTATGATGTCCAATAGGTGTTAAGCAATCAGTTCATCAACATTTTTTTCAAGAATTTGAAAAGTTTGGATAATGTTCTGAATACTTTTTTCTAAAAGAGTTATCAAATCTTCTTGTGAGGCCTTATGAATAATTGTTAATACCATTTCTTGCTTATGGGGAACACACTGATACCCCACAAAGCTAATATCAGGAATCATTTCATAAATATATGTTTTTAGCAGATTTCCGATGGTATGGGTTTCATCTTTTATCGTGATAATGGCCTTTGTTTTTTCCTCATCCATGGAAAACAGCACAAGTTCCGGCTGCGGCTCTTCAAAGTTTTCATAAATTTTTTGAATGCTTTGGATTCGGCCAATAATGATCCGGCAGGCGTTTTTTAAATACGTGCGAACGGCCTGGTTGATATGTGGCAGCGGCACCGCTGGAAAGCAAAGCCCCAGGCGGTGGTGACGCGGGTCTGAGGTCATAGAGCTTTGCTTGTAACCGCTAAGCGCCATATATTCTTTTTTATCCGTTGGGTACTGTTCAATGTCAAGGTGGGAAAAATGTGTTTTAACGGCAAGATTAAAGGCGGCATGCTTTCGTCCTATGCCCTTTTTAATATAGATATCCTCTATAATCAACGATTTTCCGGGTTGTAGGAAGCCAATCTCAAAGGTAGGATTAAAAATCGGGTATTTAAGCTTAGGGCCTGCCACCTGGATGAGATCGCGGCTATAGATGGTTTTAACCTCACAGCTATTGTTTAAACTCCGCAGAGCAAATACCAGTGTCTCGTTTTTCGCATAAATCGGAATGAAATTAATGCGGTTTCTAATAAATTGTTCCGTCATAAACAGGTCCGTGGAATCCTCGATCTTATACCCACCGGGCTTAATATCTAGCATATAATTGGGAATTTCATCTTGCAAGACCCGCGACAGGCCGTGGACCGCGGCTCTGCTAATGCCCTTAAAGTCCATAACAACATTGACCGGGACGAGGGGCAACTGCTCCTCGAGCTGAAATAGTTTTTTGGCCGCATTTTTAATAAAGAGGTTGGAAAAGTCTATCAAAAACGGTTTGATTTCCACGTTTTGGAAAATTTTTTCCATTTGTATTATAAATATATCTATATATATTCAAATTATGGTAGTTTATGACTTGCTCGTTTCTTTAAGTAAGGAATCCATAGATGTGCTACGGTTTGTAGAGGCAAACCTTGCGGCGTTTAACCAGCAGTATATTTTTTTCAATATCCAAAGAAAAAACTCGATCACGACACCCCTTCTCATTACGCCGCAGCAGGAAAAAATTTCGCAAATTGTTGAGTTTTTAATGGATGAATATAATAAGAACAATAGAAGGCCCTCCGGGCCGCCGCGTGAGCAGCCCATGCACCCATTATTGCCGTATCAACAATCCTCGGACGAACAGCCCATGATGCCGTATCAACAGCCCCCGGGGAATGATGATCAGCCATATGAGCAAATATACCATAAAAAACACGCGTCGCAGCAAGTAAATACTGAACTGAACGATTATTATCAACATATTCTTGCATTAGGCGATGAAGACAAAGGTATGGACAGCATGTTAAAACTTCCAGAAAAGGCAAAAAGGGATAGCGATGATGAGGACGACATGTTTTCTATAAAAAACTAACGACGTAACAATTAAACAAAAAATAAAAATCATTATAAAATGAATCTTGAATACGTCCAAGTTGTTCAAAAATTTAATCAAGTACTCCTAGAACTTACCAAAAAAGTATGTACCGTTGTGGGCGGGAGCAAACCCACCTATTGGTATCACCACATTAGAAGGGTTTGCTCAGAATGTCCATCCATGCCGATGAGTATGATAGGTCCGTATCTGAATGTCTATAAAGCCCAAATTCTAACAAGGGACAAGAATTTTTTTATGAATTTCGATCCCGCGCATAATGAGTACACCTTTATCATTCAAAAACTAAAAGAAGCAGCCCGAAATATGCCGGAAGACGAATTAGAACAGTACTGGGTAAAACTTTTATTTTTACTTAAAAGCTACATAAAATGTAAGCCCTTTATTAATTAAAGAATTGATGCATAACTAATAAATGGCCGGTCGTGTTAAAATAAAACAGAAAGAGCTCATAGACTCTACTGTAAAAAACAAAAATGTGATGAATCTGTTCCATGAAATTATAGGCTCAAAAGGCAATATTAATTTTAGCGTTGTCTGGCCCAAGTTTAAAAAAATCAAACAGAGCGTTTATGACTACATTTCCACTCTTTCTGTGCTGGAAAAAGCAAACGTTATGCAAAACTTTGAAGCTGATAAGAAACTGTTGGAACTTTTTGTACAAAAGCTGTGGGCTGCCTATGAAGGCTATTTCAAATATCCCGAGATTGAAAAATATGAGGTGGAAGGCCAGGTAAATTTCAATCTCGTACCTCAGTGCGTCCTCGAAAAGTTTAGCCAGTTGTATAGGATAAGAATCAATTCAGAGCTTGTCACACTCATCCTAAACAGCTGTGCCTTTATGAGTAAATATAACGATTATATTCTCAAAAAAGATCCCTACATACTAACCATAACCCCCGGCCTATGCTTTTCCCCCATTCCCAACTTCGAGGACCTAAATTTTAAACATCTTTACAACAGTGATAAAAATTCTCAGCATGACAAAGAGTTTATCATGTTTATATTATATAAGCTTTATACGGCTGCCCTAGGAGTGTACAATGCCATCTCGATTCCAGACATCGACGTAGAAGACCTTGAAAATATCATCCTATCCTCGGTGAGCCAGATTAAAAAACAAATTCCGCGCTGCAAAGACGCCTTCAACAAAATTGAATCTTCGGTACACCTGTTGCGCAAAAATTTTAACACATATTACAGTGACTATGTGGGCTCAGGCTACAACCCAACCATCATTATGGAACAGTACATTAAAGACATATCACAGGATTCCAAGAACATATCACCACGCATTTCCTACCAGTTTAGAACCATCATCAAGTATTACCGCGACATGATTGCCACCAGGCATCAAACGATGGACCCCCAGGTATTAAACCTCGTAAAGCACGTCGAAAAGAAATTAGATATGCTTGATAGAGAAAAAAATTAGTATATATAGTTATGGTGAATCTTTTTCCTGTTTTTACCTTAATTGTGATTATTACAATTTTAATTACGACTCGAGAACTATCCACCACGATGCTTATTGTTTCTCTTGTAACAGATTATATTATTATTAATACACAGTATACGGAACAGCAGCATGAAAACAATACATTTTTCATGCCGCAAAAAAATTCTTTTAACGAATCTTATAATAAAGACAAAAAATCTAATATACATATTCCCTACCAGTGGCTGGCGCCTGAACTGAAGGAAGCTGAGAGCAAGTACTGGTGGGGCAATTATGATCCTCATAGCGAGCCCGTTCTCGCTGGCGCATCTTGAATATCTTCATACGTGGCACGTCACCATCAAAAACATTGCCCAACAGCACGGGCTTGATATAAAGGTGGCCATTGTGGTCTCAACATCGCATTTAAATAATTTTTTGCCAATTTCCGGGGCGCTTAACATCGAATGTATAACCTTCCCCAGTTGCGGCATCAAGGAGATAGACCTCCTATGGGCGCGCATTAAACTATTTCAACATTACTGCGCCATCGGTGCCCGTCTTTTATGGCTGGTAAGTGCTGACATCAGGCCCCCTGTTTCAGCGTGGCCAGCCATCGCCGACAGTCTAAAAAAGGGAGCAGATGCGGTCGTTATTCCCTACCCCTCCCGATGGAACAATCTTATACCTACCGTCATCAAAGAAATAGTTGTCCACCAAAAAAAATGCCTTGTGGCGGTGGATGCACGCCACCTTGATACAGATACCCAGATTGTAGGGGCCGGGATGGGCTGCATCGTCCTAACCCTAAAGGCCCTTATGGTGCGCCTAAGTATTGGCAAACAGCCCGTTAAGATACTGTGGCCCGACCTTCACGGCACTGCCGAGGGCATTCCTCTGGAGGGGGTGGAGGTTGGCTGGTTTTTAAACGCTTATGCGCATAAATTAAATATACGCTGCCTAGGGGCTGATCATATTGCGCAGCACTTAACTTAATTCTTTATTTAAAAAGTCCACGCATCCAGTGGCGGCCTACATTAAGGGCCTACGCACATAAATATACACTGGCTAGAAGTACGCCTTCATTTAAACCATTGAATTATTTATATAATGGCTGCAAACATTATTGCAACAAGAGCCGTGCCAAAGATGGCCAGCAAAAAAGAGCATCAATACTGTCTGCTAGACTCCCAGGAAAAGCGTCATGGGCATTATCCCTTTTCATTTGAATTAAAGCCTTATGGGCAAACAGGCGCAAATATCATAGGAGTACAGGGCTCACTTACCCATGTTATCAAAATGACAGTATTTCCATTTATGATTCCTTTTCCTTTACAAAAAACTCATATAGATGATTTTATTGGTGGACGCATTTATTTATTTTTTAAGGAACTGGACATGCAAGCAGTTTCTGATGTAAATGGAATGCAATACCACTTCGAGTTCAAGGTTGTTCCTGTAAGCCCCAACCAAGTAGAGCTTCTTCCTGTGAATAATAAATATAAATTTACATATGCTATACCGGTAGTGCAATACCTTACCCCAATCTTTTATGATCTTTCGGGACCGCTAGATTTCCCATTAGATACTCTTTCGGTCCATGTGGATATCCTCTCCAATCATATACAGCTTCCTATCCAAAACCATAACCTAACAACGGGTGATCGTGTTTTTATTTCTGGATATAAACACCTGCAAACGATTGAATTATGTAAAAATAACAAGATTTTTATCAAAAATATACCGCCGCTTTCATCCGAAAAAATAAAACTATATATACTAAAAAATCGAATCAGAATTCCGCTATACTTTAAATCTTTAAAAACGTCTAAGTAATAACATTTTTATAGTCTACTCCTAGTTCCGAAATAGGCTGAATTTCTTTTTTAAGTCCTTTAAACCAAGGATGTGATACAAGACTCTTAAAGGAAAGCCGCTTATTTTCATTAATTGTTAAACATTCCGTGATAAACTGTTTTCCCGTCTCTGAAATGTTCTCGGGAATATAATTTTCCCGTTTCAGGATATCATTTAAATAAAAATTTTCTGCACGAAATCTAAAAAGATTAACCGCGACCATACCTATCGTCCACACGGTTAAAGGAAGCTGGTAGTAATAACCATAATAATAAAATTCTGGACACACGTATTCCCATGTTCCAAACATATTATATTGGGGACGGGTTTCGTCTAATCTAACAGCGCTTCCAAAGTCAATGACCTTAATGATCTTTTGATTTATGTCTATAATAAGGTTCTCATCCTTAATATCCCCATGGATAAAGCCCTTCTCATAAATGTTTTGTATAATAAGAATAAGCTGGAATATTATTTTTTTGGCTTCGGTTTCCTCAAGTTTTTTAAAGTAATGATAATGAAGTAGATCAACACTATTTGGAATATATTCTATGATTAGTATATGATACATAGCATTTTCGGTATATTCGATAAGCTTAATAACACCGGGAGTATCTTGCAGGGCTTTCAACACGATGACTTCATTTCCTGGAATTTCTTTTTTAGAAACGTACTTAAATATAATGGGTTGCCCTACTTGATGACCCAAAAAGACGTTATTTCTGCCACCCTCAAACATGGGTCTCGTCGCAATGAAATACATGTGCTGCGTTGTGGAGATCCTTTCCACCTTTGCTGTAGGATAAAACGCATATTGTGCCTGGGGATTTTTTAACATTTTTTTAAGCTGTTGTTCCGGCCTGGACATGTTTTATTAGCTTTATATATAAAGGGTTAGAAGGTTTAATTTCAATATATGCCTTAATGATGGGATTATATTCGTAAAAGGTATAGCCTAATCCTACGTCTTTGTTTTTTTGGTAAAAAAACTGTTTGCCCTCGTAGGATATGCTATAGGCTTTTACTTCGGCTTTTACAAGCGGTTGGCAGGGATTGGGCAAACGTAAATCGCGTTCAAAGTTTTCATGAAAAAGCAAAGCATTTGTGGGCTGACACATCAGACAGCCGCTTTCGCCATTGAAGGCACATTCAATGGCCGCCCTTTTTAGTAAATCGCGGAAAGCAGAATTAAGATGGCTCTTTTCAAGCCCCCTTTCGTGAAAACGCTCATCAATCGTTTTTTGTTCCTGACTGCCTTCGGGAATACTATAAAACATTTTTTGATTAGCCACCGCGATGTACAAAAAAGGCTGTACGGTTTTCTCCTCGGGCGGTAGCGCATCGTGGCTACCAATGCGTATAATGCGCGCCTTCACTTGATCCTCTCGGGCCTTATCCCAGTACGGCTCTAGGATATGAACCTGCCGCCCGTATTTGAGATCCAATCCCTCAGCTCCTGTTTTAGAGACGAGTAAAATTTTAATAACCTCTCCGTGTATATTCAGCGGCGAATTCCAAAGCTGCTGGATCATGTCGCGCTCTTTAGATAAAATTTTCCCTGTAATAAGCGTAAATCGTGTTATTTTGGAGGACAGGACTAACGTATGGGTCGGCCCATCTTCCGCAAAGTTTTTCACCATAAGATCTTTCCCATCCTTATGAAGGAGGATGGTGTTGTGCCCTTCTTCCAATACTTTTAGGGGCTGAAGGCACTGGTAGCCCTCTATTTCTAAAAAGCGGGCCACGACGTGAAGGCCCAATTCCACAAACTGTGAGTAAATGAGCACAGGGCCCGGAGACGTTTTAATATTTTTTAGCATGCGTACTATTTTGGGACTAGAATTTTCTGTGAAGGCCTCTTTGGGCAGCTGCTGAACAGCCTCTGATAATTTTTCATCCTCCTTTACTGTTAGCATTTCGGACGCGAAGATGCTGATCATACGGGAACGCACATAGTAGGAGGAGCCTGACTCTTGCTCCGATCCTGGCAGGCAGAGGGCGGCGGCATTTATTTTTTCATACATTCCTGAGCTGGCGTGCTTTTCCGCGTTTTCAACGTCTCGGGCCAGCAGATATTGCCTATACTGCTCGGGTGACATTTCAACCTTTTCTATAATAAGAGGAAGCTCTGTGGGGAATAGCTTGTTGAGCTCATTCTGGTTTCCAGCGTAGCTTATCATACCCACTAGGCGGTTTAGTAGTTTGTCCGCGTTTAAAGGGCTATTCGTTGTTTTATTGACATAAGCGGTGTAGAATCTTTCATAGTGAAGAGGTAATAAGATTCGCCCGCTTAGCATATTAAAACAGGGCACCATTTCAAAGGGGTCCTTCGAACACGGGGTGCCTGTTAAAAACAGAATACGAATATTTTTAGCTTGCATAATATTATTGTACAGCTGGCGGGCATTTGTTTTATCATTGGCGCTATTGATAATTCCTCTAAAGAGGTTGTGTGCCTCGTCAACGATGAGCAGGCATCCATTTAGGGACCCTCCCGCCTTTATGATCTGCTGCCCCATGTTGTAAGCGTCTAGGGACACAAACCTGAAGCGCCGCGAGATTTTTTGTAGCTCTTTGGAGTGATCCGTCGTTTCCGGATATAAAAGTTTAATAAGCTTTAACAAAGACTGTTGGAAGTTTGAGTGCAACGACTTGGGTGCGATCAGAATCGGGTTGTAAATATGTGAAAGTGAGATGGCAAGCGACAGGCTCAAAATGGTTTTCCCCATGCCCATCTGGTGATAGATGAGGAGGCCCCGTGTGTTTTCCCCCTGGCCTATCCCAAATTTAGGATCCGAAAAGGCGGTGTAAATTAAAAACTGGTAGTATTTCAGGGCTCGTGCAAAGCGGGCAGTGAGTGAGGTGTCTTTGCTTTCCTGAAGCTCTTTATATTTTTCATATACCTCTTTTAGGTATGCTTCTATTTGGACGGGGAAGGAGGTGTTGTTGTGCACGCAAGACATGACTCGTTATAAGGATCCCATATTAAAACTTCATTAGAAGAATAGGGCTGCTGATAGCTAGCGCTGCACTTAAAAATGGGGTAGCCCTTTTTCTTGTAAATCCGGTGCCTGTCGTAGACCTGGCTAGAAAGCGGGCTTAGTGTATCTTTAATGTCCACAACGATGCGTACCTTTTTTTCATCCGATCCCTGCCGGGTAATACGTCCCAAGATTTGCTCCATGTTGTTTCTGCGGGGCGTTGCCATGATGATCGATGTCATATGCTTGAAGGAAATGCCTCTACGCCCGTAGCCATAGGTCAGCAAGATAATGGAAGCGCTGTGTGCCTGAGAAAGAGCGGTATTTGAAACCCCGCCGCATAGGAGCGCCACCTCCGGAACGATAATTTGAACATCTTTGAATTCTTTGGAAAGCGCCTGATAAAAAATTTCTAAAAGTTTGCGAAATTCCACGAAAATGATGATGCCATACGGCTCATCGGTCCCCCATTTGTGAGGCTCAGCGGTATGCAGGGAGTAAAGCCGCTTTGCCTCATTTACGACAAGTTGTATACGCGAAGGATCTTGAAGTAGTTTATCAATGGTGGCAATGGCCGATACCTTTTCATTAATATACACAGGGCTAACGAAGTCAGGATGTCCCTGATATTCGATTTCCCTCACGTACCCGGAAAAGGTTGTGGTGGGACTTACAGTCCTCTGGGGCTGTCCTAGATGGTGAATAATAATCTTGTCCATACCATCGGGCCGGTCCAGGGGTGTAGCGGACAGTCCTAATATCCGACTAAGTTGTATTTTCCAAAAAATTTTGTAATTCTCCGGCGAGTGTAATTCATGTGCCTCATCTAACACGACTAGACCAAAGGGCTCAAAGAACTGCTCAGGCTTCTTGCGCAGGGTATTAATGATTCCCACGATGACGTCGTACTCTTTGCTCGTCATGTCCTTTTTCTTGCACGCTGCATTATTGTAAGCAGCTACACGTAGGTGGGGCAGGAGCAATGTTAGCTCGTCGATCCACTGTATTTGAATCGCCTTGGTGGGCACGATGACCAGGGTAGGGTACAAAAGTTTTTGAATAATGCTGATCGCAATACGCGTTTTCCCCAAACCGGTATTTAGATGTAGGTAAAAGCGCCCATAGGGGGACAGGAGCTTTTTATGAATCTTATCGACCATTTCTTGCTGGTAGTTAAATAGTGGAAATTCTGTTTCAACGCATGGGAGGGCCCGCAGCGACACGGGGCGCGTCGTGTAAACCATGTTAAACATTTCAAACTGCTTTTGCAGCAATATGGGAAAATAAATGTATTCCCCCTGCAGCGTGAAGGCAGTTTCCTGTCTTATGGCTATGTGCTTTGGCTGCCCGGGTAATGCCCGCGCCGTAACGGTGAGCGCCTTAAGAACGCGCCCGAAATCATGTTGTAATTTACTTTGTAGCTTCTTATAATTTATTCCTATTCCAGCAAAGGATATAATGGCCTCCATTCTCACGCTGGACGGGTTATATGCAGAGGTTCCAAAATTCTTACCAGAGGCGTTACGAGAGGGCTGTGCTGGCAAGAATCCTCTAAGCTTTTATATTCAACAAATTTTAAATTTAATGGGATGTGACGGTAACGAGTACCATGTTCTTTTTACCAGCAGCTCCGAGGAAGCAAATACTCATATGATCATGGCCGCCGTGCGTCGCCATTTGCTGCGGACGCAGCAAAGGCCTCATGTCATTATCGGAGCAGCCGAGCCCCCTAGCGTCACCGAATGTGTGAAGGCATTGGCGCAGGAAAAACGCTGCGTATACACCATCATCCCCCTAAAAAATTTTGAAATAGATCCTGTTGCGGTATACGATGCCATACAAAGCAATACCTGCTTAGCGTGCATTTCAGGCACTAATGCTGTTGTCAAAACGTTCAACAAACTCCAGGACATCAGCAACGTGTTAAAAGGTATTCCCCTGCACTCAGAAGTGAGTGATCTTGTTTATCAAGGATGTATTCAACAAAATCCGCCCGCTGATAGTTTTTCAATAAATAGTCTCTACGGCTTCCTGGGAGTCGGTGTTTTGGGAATGAAGAAAAAGGTCATGCAAGGATTGGGGCCGCTCATTTTTGGAGGAGGGCTGAGAGGCGGAAGCCCTAATATACCCGGAATTCATGCCATGTATAAAACGCTAACCCAGCAAAGGCCTTCTATGAAAAAAATAAATACAATACATACGCTGTTCATGAAAACTTTAAAAAAACATCAGCATGTATATCTACCCATAGGGGGCGTGTCTGCAGAGGACACGTCTGCAGAAAACATATCTACAAAAGACATGCCTGTTGAAGGCCCGAAGGGACTCCCGGGCTATATTTTATTTAGCGTTGGCCGTCGCGCCGAGGAGCTACAAAAAAAAATTTTCACTAAATTTAATATAAAGGTTGGCCGTGTTGTTGACTTACAAGAGATACTGTTTCGTATCAAAATACCCCAAAAATACTGGGAGACATTATTGTTCATCCAATTAAGAGATAATTTGACCAAAGAGGACATAAAAAGAGTTATGGTTGTTTTGATGCATTTAGATACCATCACTCCTCGTGGCTCTCTTCCTCCTCCGAGCCACTCTTCTTCTTTTTCTTAATCGTTTTTGTTTGTTCTATAATAAGGGAAAAGAACTCCGTGGGATCTTGTTCCCCGTACAGGTTATCTGCGACCATAAGGATGCTTAGAATGGTAAACAGGTGAGAATACATAAGGGTTTGCGTTTTAAGAAAACCCTGACGTTGAATCATAATTGAAAACACCTTGCAAAGCCGACTCATCAGTTGTTCTGTAATGGCGTTAAGCATTTTCTGGAATTTTTCTTGGTTTTCGGGTGTGATTTTATATTCATGTAGAAAGTGTTTCACACCTGAGGAGAAGAATCTTTCCTCCTTCGAGAGCCCATCTTTGATGATGGGAAGTTCCTTGATCAGGGCAAACCATTCCTCCTCTTGGGCTTGCGGATTCTGAAGATACTGATGGCAGATATGGTTTAGAATGGTGCACACGTAGCTAATAAGCTCTGAGCTGATTCTTTGGTTGGTTTTCAAATGTTGGCGAAAGTAGTTTTTCACCGAAGTGCATGTAATAAACGTCTTCATTTTCTTATAATATACAACAGTATGTTGAGTCTTTAATTTAAAATTACAAGGAGTTTTCTAGGTCTTTATGCGTATAGGTGTTTCTTTGTCGTAAATTTTCAATAGCCGACATTGTTTGTGAAGCAGTGTTCTGAGTAGTGACTGTCGTGTAAGGCTCAGCCGGATGAGCAGGAGCACTCGCGGCCGCAGGTGCGGCCGCCGGCCCGCCAGTTGCCATGACTAGTCTGTCCGTAACTGGGTTGTCCGTAACTGGTTTGTTTGTTGCTGGTCTGTTTGTTGCCGGTCTGCCCGTGACTGGCTTGCCTACACTTGCTGTAGTCGCTCCAGCTGGTTTAGAGGTACCTGGTTGTGGAGTGACTTCTACCCACTGCTGATCTTGATAAGGATTTATAAACTGTATATCTTCCTCCTCAATAGCAGCAGCTTTTTTCTTTCTTGAAGAGAATAGATAGATTAGAACGATGATAATGATGACTAAGACCACGATAGCAATGAGAATAGTATACATATGTGTGGAGAAGAAGCTTGGTGTAGTGACTGGTGACAAACACTCACCATAATGCCGCGGATAAACCGGTTGAAAAAATTCAGAATCCATTTAAGATACTATTATAAATAATATATAAAAATGTTGTGGCGCAATGAAATTACAGAATTTATGGACCAACTTTCCAAGTATTCTCAAGAAATCTTAAAAACGTTTAAGCAATTGCGTCCTAGTGAATATAAACAATACAATGAATTTTTAACACAAGTTACACCGTTGCTGCAAAAAACCCCTGAAAAAATTCCAGAGTTGGTTGACCATATATTCAATTACCTAGACAACGTTGAAAAAATTTGTGAGCTCCTCGTGAATGCTAGCTCAATTATTATTAGTTCAAAAATACGAGAACAAGTAAAACACGGAATGAGCTTCAGCTATAAAGCCGACCTCGACTCCTTGGCGGACATTCTCTCTCAAAAACAGTACGTGCTTATGCATCTTTCAAAAAATATTGCGGCCGAGTATTTTAATACGTGTTTAAACCAAGGGAAATCCAAGTTAGATCTCAAAGCTGCCTCTGTATTTTATAGTAGTCGTTCCCGAACGGCAAGCTCAGCAGAACTCTATAGAAAAATGCTATACGCCTATGGTTCACCGCAGGAAATTAATTATTATACTGAAAAAGCCCGAAATAAGACGTTGGATGTGGAGGAGAGCGACAGCATGGCCATCATCGAACGAACGGCCCGACACAACCTTTCCCTTATGCACCCGCTAGAAGCCATGGGGCTTACCTTTGGGGCAACCAACACGGACGCCGACCCGGAGGATCTGAAGGACAAAACGGTGATAAATTTAACGCTCCCGCAGGCAACAGAAAGCATCACCTACCATCTTAAATCCCTAATGCAGCTAAAAAAAGTAAGTACGGCTTCAGGACTAAATACAAACATTTTGAAAGCATTTGATAATATTATTTCCACCCCTGTGAAAAAAAATAAAATGGCCTCCAAGTTGGCGCCCGGGATGGATGTCGTGTTCACTAGCGATAACGGAAAAACATTTTTTACTAAAAACATTTTAAGCAAAAACATGCTAGCGGGGCCCAAAGAGCGGGTGTTTGCATATAATAATCTCATTAGTAATTTAAATAACTCCTGTTTCATACAAAATCACAACGATTTTTTAAGACAGCAGGACTCTTGGCCCTTCTATGACGCGCACAATTTTACCAACAAGTTTTTAATGCAGCCTATTTTTTCGGGGCAGACCCGTCCTCGGCTTCAGGGAGCCATGGAGGCGGCGCATGTGGAAACGCATCTCACGGCATTTTTACAAAGTATTCAGCCCTCTAGGCCACAAGATCCCTCTGTTTTGGCTTCCCCCAAGTTATCTGCTCTAATCTTGAACTAAAAACAGCCTTTCTTGGACTTAAATGATGGTCTACCAGTTTTTGAAATAACTTAGAGAACTATGAAGATTTTCATGAAATTTAAATTAGAGATTTGCAAAGGTTACTTGCGGTCATTTTCTGTTGAATTAAATAATTATTCGAATAGTATAATGTCTGAAGATATTCGTCGTGGTCCTGGCAGACCGCCAAAGAAAAGGGTTGTTCCCAACTTTGAGCGCAAGGGCATTCTGGAAAAACCAGTTCGGCCACAAAGCCGTCTCGAGTTTTCCTATGATAACCCGCTGATATTTAAAAATCTTTTTATTTACTTTAAAAACCTTAAAAGTAAAAATATTTTGGTGCGATGTACCCCCACCGAGATTACCTTTTTTTCACGTGACCAGTCGCAGGCAAGCTTTGTTATTGCCACCATCGACGGAAAAAACGTGAACCATTATTACGCCAGTGATGTCTTTTGGCTAGGCATCAACAGAGAGCTCGTTGAAAAAATGTTTAACAGCATTGATCGCTCTTTTTTAAAAATTACCATCGTTCACCGCTATGACAAGCCTGAAACCCTGTTTTTTATCTTTACGGATTTTGACATTGACAAGGAGTGCACGTATCAGATTACGGTCTCGGAGCCCGAGCTCGATATGGACCTTATCGAAATGGAAAAAAGCATCAGTGAAGAAAGACTCAAGAACTATCCTCTGCGCTGGGAGTTTACCTCCAAGCAGCTCAAGAAAACATTTAGCGACTTATCAAACTACACCGAGCTCGTGACCATTGAAAAACTCGGCGGCGATACGCCGCTGCACCTGTATTTCCAAAAGTTTAACTCCATCTCATACCACGAGATGTATAAATCTTCCAACAAGATCAACCTGACCTCGACCATTCCTAAGTCGCAGGTGTTCCAGATAAATGTTAAAATTGCTCACATCAAGTCGCTGGCCTCGGCTATGGTCACCGACAAGATCCGCATTCTGTGCGAAGAAAATGGGAACCTAATCTTTCAATCGGAAATGGATGCCCTTATGTTAAATACGATTACCTTGAACACCACGATATAGTTCGGTAACATTAGATGTTCTAATATTTAGCATCTAAATAATACGCTGTAGTCCGGTCAGGGTTGCGTCACAGTTTTCCCATTTTTTTGCCTCGTCGGCGGTGGCCACCGTTGCCCTATCATTTACGCCCGGTAAGACAAAGCTAAAGGCGTTCAGCGGGGCTTGGCAATGCCCGCCCAGCGTGAAGGAGCTCGGAGGATTTTGCGCATCCCGAAATCCCTTAGCCATGTTGTTTAACACTTCGGTTACGTCAATCGAGTGAAGGGATCCCTTGGGATCCGTGAATGTAAAGACGCAGTTTCTAAAGCGCATGTATGCGATGGACGATTCATCGGGGGTTTTGAAGGTAACAGTGTTCCCCTTGCTGTACTTAAAGGGGGACCATCCGGTAAAATTATACCAAATGAAAGCAATAATAATTAAAATAACCAACACAATAGTTATAGACAACACAAAGTCTGTAGTGCCGCCCATTATTAAATAAAAATATTTTAGACCGCCGGCTTAAAATTTACTTATTGCTCATAGCTTAAGTCTATTTTATTCATAGCTTAAGTTTATTGCTCATGGCTTAAGTCTATTGCTTATAGCTTAAGTCTATTTTATTCATAGCTTAAGTCTATTGTTCATGGCTTAAGTTTGTTGCTCATAGCTTAACTCCATTACTGATAGCTTACTGATCATGACTTAAATAAAAATATTTTGCCCGCTTAAAAATTGTTTAGGTTTGAAAAAATAAGAGATGGAGGGGGCAACTTATCGTCATTGTGTTTACCCCCACTGGAAGACATCAAACGGTAAATAATTATAAGAATCAAAATGATTAATATAAGGGTTAAAAAAGGATGATTCATCACATTAATTAAAAACGTATTTATAACGCTGTTGCAGTTGAAATTTTGGTATAGGTCGGAAATATTGCCCGAGCCTCCGTATTCTGCAATGTTCTGACATATGGTGAGTCCGGAGGGGCACTGCTTGTTGGTCAAAATATTTCTTTGCTCCGTTGTTTTATAGGCATTTTTATTTCCATTACACGGAGCAAACGCACATTCAGGCCATAGGGTGCCGGAGTTCACACAGGCACAATACTGGCTATACGCATACTCATCCTTTGAGCACAATCCCTGTTTATCGCATATGCTCCCAATAATATTGTCATCCTCCGCCGTTTGTTGATTTGTATGCGAGCGTAAAATAGCGGCCCAGGCCTTGGGCTCCTTTTTTTGCAGCTCGGAAATCGAAGGGCCTGTACAGCTAAAGTCGACCCAAATATCATTGCATTTCGTGGAAACTGGCATGCAAGACATAATTGAAATAATTAATAAGTATATATCATGGCAACAAATTTTTTTATTCAACCTATCACCGAAGAAGCTGAAGCATACTACCCACCTTCCGTGATAACGAATAAACGGAAGGACCTGGGGGTAGACGTATACTGTTGCTCCGACCTAGTGCTTCAACCTGGACTAAATATTGTTCGCCTGCATATTAAAGTAGCATGCGAACACATGGGCAAAAAATGCGGTTTTAAAATCATGGCGAGAAGCAGTATGTGCACCCATGAACGGCTGCTCATCCTTGCAAACGGAATTGGTTTAATAGACCCGGGTTATGTGGGCGAGCTCATGCTCAAGATCATTAATCTTGGCGACACCCCGGTCCAAATATGGGCCAAAGAATGTTTGGTGCAGTTGGTGGCCCAAGGTGACCATGTGCCTGACCATATCAACATCCTAAAAAGAAACCAAATATTTCCGCTGTTTGCGCCTACCCCAAGAGGCGAGGGTAGATTTGGGAGCACGGGCGAGGCCGGGATTATGAGAACTTAATTTTATTTTTTTTCTTAACATAATGGGAGGCTCTACAAGCAAAAATTCCTTTAAAAATACGACCAACATTATCAGCAATTCCATTTTCAATCAGATGCAAAGTTGTATTTCCATGTTGGATGGCAAAAATTACATAGGCGTATTCGGTGATGGAAATATTTTAAACCACGTTTTCCAGGATTTAAACTTATCATTAAACACAAGTTGCGTGCAAAAGCACGTAAACGAGGAAAATTTCATTACAAATCTTTCGAACCAAATTACTCAAAATTTAAAAGACCAAGAAGTTGCGTTAACCCAATGGATGGACGCAGGAACTCACGATCAGAAAACGGATATAGAAGAAAATATAAAGGTAAACTTAACAACCACACTTATTCAAAACTGCGTTTCATCCCTGTCGGGTATGAACGTGCTGGTGGTGAAGGGGAATGGCAACATTGTTGAAAACGCAACTCAGAAGCAGTCGCAGCAAATCATCTCTAACTGCTTGCAGGGGAGCAAGCAGGCCATAGACACCACAACCGGCATCACTAACACGGTAAATCAGTACTCACACTACACCTCAAAAAACTTTTTTGACTTCATTGCAGACGCAATTTCGGCTGTTTTTAAAAACATCATGGTCGCGGCTGTAGTTATCGTTCTAATCATCGTAGGGTTTATAGCCGTCTTTTACTTTTTGCATTCACGGCACCGCCATGAGGAGGAAGAAGAAGCTGAACCACTCATAAGCAACAAGGTATTAAAAAATGCTGCCGTTTCGTAATAATTTAATTAAAAGTAAAAAAAAAAGGTATTGTTATAGTGATGGCAGATTTTAATTCTCCAATCCAGTATTTGAAAGAAGATTCGAGGGACCGGACCTCTATAGGTTCTCTAGAATACGATGAAAATGCCGACACGATGATACCGAGCTTCGCAGCAGGCTTGGAAGAGTTTGAACCCATTCCCGACTATGACCCTACCACATCAACTTCCCTGTATTCACAATTGACCCACAACATGGAAAAAATCGCAGAGGAAGAGGATAGTAATTTTCTACACGATACTAGGGAGTTTACTTCACTGGTCCCCGATGAGGCAGACAATAAACCGGAAGATGACGAAGAAAGCGGTGCAAAACCTAAAAAGAAAAAACATTTGTTTCCAAAATTAAGCTCGCATAAATCGAAGTAAAAATTGAAGCGAAAAAAAGTAGAAAAAAAATGTTTGGAGCTTTTGTAAGCCACCGTTTGTGGTCAGATAGTGGTTGTACGACCACCTGCATCACAAACAGCATTGCTAATTATGTAGCCTTCGGCGAACAAATTGGATTTCCCTTTAAATCAGCTCAGGTATTTATTGCCGGCCCTAGAAAGGCTGTGATAAATATTCAGGAAGATGATAAAGTTGAGCTTTTAAAGATGATTGTTAAGCACAATCTTTGGGTTGTTGCTCATGGAACCTACTTAGATGTGCCCTGGTCCCGTAAGAGTGCGTTTGTTACACATTTTATACAACAAGAACTACTTATATGCAAGGAAGTCGGTATTAAAGGGTTAGTTTTACACCTAGGCGCTGTGGAGCCTGAACTTATTATGGAAGGACTAAAAAAAATTAAGCCGGTTGAGGGGGTTGTCATTTACCTGGAAACCCCGCATAACAAACATCATACATATAAATACAGTACAATTGAGCAGATCAAAGAATTGTTTTTACGGATACGAAATACCAGGTTGAAACAGATTGGTTTATGCATTGATACGGCTCACATCTGGTCTTCCGGTGTCAACATCTCCAGCTATAATGACGCGGGGCAATGGCTGCGCTCGCTGGAAAACATTCATTCCGTGATCCCACCAAGCCACATTATGTTCCACCTAAATGATGCCGCCACAGAATGCGGAAGCGGTATAGACCGACATGCAAGTCTTTTTGAAGGAATGATTTGGAAATCATATAGCCATAAAATAAAGCAAAGCGGTTTATATTGTTTTGTTGAATACGTTACGCGACACCAGTGTCCGGCTATATTGGAGAGAAACCTCGGGTCTTCCATGCAATTACAAACCGCTTTAACCGCAGAATTTACTACATTAAAATCGTTATTAAAATAAGGATGAGTTTTAGCGAATGTCCCTTAGTTATTAGTGCATGCAAAAAATTTCTACAAAAGCGTATTACAATAGAGAATGAAGCACTTATAAATGCCTTAATAACCGCTTTAGCGCAGACCAGCACGTTGAATGATCTTTGTTTATTACCTATTCAAACCTATTTGCTTAGTTATAAAAATGCTTTTGAGTGGATACACTTCGTATGTATTGCAATCACCACTATTTTGGATAATAAGTATAACTGGAAGGACTGTACGGTAGATATTAATTATATTTTTCTCCATGTAACCTATATTTACAATATTAAAACCAAGGAATACCTAGACTACTGTTCTTAAACTTTATTTTTTCTATATTTACGCCAAAGAGAATATTTAAAGTTTTTTTTGAAAAAAAATAATATATGTAGATAAAATTCAGTTACATGATATATGTGTAAACATGTGTGGTAAACAACATATGGTTATGCTTTATAAGATAAATGCGCATAATATATGTAAACAAAATATGGTTATGTGTTAAATGCATATAAATGTATTTTAACGTATATCTTGTGATAATGGATATATGCATTTATTAAAAGAGGCTGTATTTATTATAAATCTTGCTAAGGATGCCATTGTCAACATATATCCCATGTTGGACAAATTGCGTTGCGATCCAGTTCTTTTTTTTTTGATTTTGTTTAATGCTATCCTTTTTGAAGGGATGGTTGTCCACCATATTTATTCGATGTTCAATGAATAGGTCTGCTTTTTCGTAAGGCAGTGAAGGTCGTTCCAAGACTCCTTGAACGAAGGACGTGTTTTCTTGGATCCACTTAAAAAGCACGTGGCATTCAAAAACAGGACAGTGATTGGATCCTTGGATATGCTTTGGACAGCCAATGCTTGAAGAGATGTAGTCCCTTTTCTTTAGGACAAGCTTCTCCACGCTGGGGCAACAGAGATCGTTCAAGTTCTGGACGGTCGCATTTGGAATGTTGAAACTTCGTATCCATTCACCCTCGGGTCCTCCCTTATGAAGAAGGAGTATTTGCTCATGGTCCTTAGTAATCTTAACCAAATGTTGGAAGATCATTTTTTTACCTGCTTTAAAGGCCTGAAGGGTGTCAGTTGGCAAAGCTATTGAATTCGGGAGTGGGCTTTCATCAAGCGTGAAATGGTGAATGTGACGCGACTGGAAAGAAAACGACCGTTGATTTATTTTTTCAAAGATTGGGTCGATTCCGCCATGAAAGAACAGCTGCAAGATTTTAGAAGGCGTATTTTTTTCCCAATAAAAAATGACCACTTCTCGTGGGATTAAAATCGTCTGTGTCCCATTTTCATTATATAATTGGCCCATAAAGCCATCAACGTCAATCAACACCAAAAGCATGGTATAGAGAGCTTTTAGAACCGGAGTTCGTTAAAAAAATACAAAGTTCGTTTAAAACGTGTAATGTTACTAAAAAAATGTAATGTTTAAATGATAATGATACCACATGCATTAATGAAAAAAACTTTTAAATTTTTGTTTTAATATTTGCATGAAAATGGAAACATTTTTAGTCTGTTTATTTCACAATGCAGATGGTTTACATCAACAGATTCAGGAAATTTTGTATTTATTGCGGATGCATATTTACGAAACAAATCTTTACTTAAAGCAGGAACTATCACGGCTTATATATCCAAATAGGCAACTTTCTTTTGTGTTACTTATGCCCCTTTCCCTTCTAAGAAACTGGGATGACATTGAATATTTAACGGACGTTGTAGATGATAAGCAGACTCTACATTACGCGGCAAATTTGCTGACAAACTACGTTCTACATCTATCCATGTTTCAAAAGCTGACAAAACCATACTTCCTTTTAGCGGTCAAGCGGGTCAGCGAAAAACTCAACAAAAAGCAGCGACATTCATTTTACGAGGTATTGGTAACCTCCGAAACCTTGAATAATTATGAAAACCTATCTAAAAACATTTTAAATACGTTGATGTTTGCCGTGCGCTACGTATTTAAACCTACGCCGAACTATTCAGAAATTCTCGCAGAGTTGGAAAAAAAAAATAAAATTCACCATATTATTTTTAATATGGTAATTACGGATTTTGCGCAAATCCGTGAACAACAAATGGATAAACATCTGTGTGAAACAAATAATGAGCTTCGTCAGGAATGTAAAGAAACTATTTTTGATTTAAAGGTGGTAGGAAATGTTTAGCCAATAAACTCATGCCCGCATTTTTTACAGGTACAAAATATCGTGGATGGCTCATCGAGGGCGCGTGTTTGTACTTCTCTGTAGGTACACATACGCTGCTTGCAGTTGGGACACTTATAAAGTTGTGACGTCTTTTCGGCGACCTTTTGCTGCGAACGTAGAGTAATTTCTGTCTTCTCCTTTAAGGCGGCAGAGGGGCAAAGCTCGGCGAACGTCATGCTACCAATTGCCTCCGGTTTTAGCTCGCCAGAAATTAGCTTATTAAGGGCATCGTTATCCTGTTGTTGGTGACTTTTTTTTTCGCAGTTAATAATATGATTGATCGTCCCACAACGGGTTGAATATTCTTCTAAAAAGGTTTTTTCTTGTTGCTGGTACGTATAATGATAACACGAGGCCTCGATTTTTTGCGCGTATTCGGTGCATAAATCAGTATGTTCCTTAAAAAACATATGTTTTTGAAGCGTTCTAAAAAACATCATTTGGATGATATCACGCATTTCCAAAATAATATAGGGTTCTAGTCTTTTGGAATCTTTCATAACTAGATCGGTGGTAATATTCTTAGTCATACAATTTATTAAAAATGGTTTAATATATTGTAAATATTTTTTAGGCGTGTCAGCCTGTAAAAAACATTCTTGTTCAATCTTATTTGTAAGGATAGTATTTTGCAAATACTTATTTAGCAAAAATACGATAGAATCGCGGGCTATATGCATTTTCATATAATTTTTTTTTTAAAATTTAATACAAAAAAAAGAAGTATAGACTCTTCTTCTAGTCCGGTTAGTTCGTTGGTTGCCTCAACATGGAGACTCAGAAGTTGATTTCCATGGTTAAGGAAGCCTTAGAAAAATATCAATACCCTCTTACTGCTAAAAATATTAAAGTAGTGATACAAAAAGAGCACAATGTCGTCTTACCTACAGGATCTATAAATAGCATACTGTACAGTAACTCAGAACTTTTTGAGAAGATTGATAAGACAAATACCATTTATCCCCCGCTTTGGATACGGAAAAACTAATTGTAACCAGTAGTACATTTAAGGATAGTTTAAGCAGTAAATGTAGAATAACACAGTTAAGCAATAAATAACAAGTATATAGGAATATATAGGAATATATAGGAATATATAGAAATATATAGAAATAGCTAAGCTTAATACTAATTCAGCTTTTTTTTTAACTAAAACCTGAATAGATGCGAAGTAGCGGACATATACATACTAAAATAAGCCATACATTTACTTTCTTCTTGAACATGAAACCTTTTTTTCTTCTGTTGTTGGTATATAAACAATAGGACTGTTTGCTGAGGTTGTATGATCTTCTACAACTGCTGTCTCAGGATGACGATGTTTTTTTAAACTAAAAGTGTAGGATGGAATGAGTGGAATATAGTTATGGCTCGACTTATCCTGTTTCGTACAGGAATATTTTTTACAAATAGAACGCAACAAGCATATGAATAAAAACAGAAATGATATACAGGAGCATAAAATAGATATGAACACTAAGGGGTAGCAGCTTTTATAACGTTCCGTATTTTTCTTAGCTATCAATTGATTTACCGTAATATTTATCTCGGGAAACTTTGTTCTACAATATTTTGTTTGGTATTCCAGAAACTCATGTCCTGGCTTATTCCCGCAGCTTAAAAAATGATACAAAAATGTGTTATTGTTACTAAAATTAATTCTTCTTAAGAAAAACTGCGGAAGACGCTTTAGGTACGTCTGTTCCTGTTTTAGTAGGAAGTAGTATAAGGGACAATTTCTTTTTCCACACATTAGATTATTGTAATATAGGTAGGTTGGGGTGTTGGAGCGAATAAGTTTTCTGAGTATGTTATAATCTATGACTTGTAAATCGTTATACCTTAGGTCCAAAAACTTGAGTTCTTTACCAAAGCCACCTGCAATTTCAGAAATATTTTTCATCCCGCAGCGGATAATACGGATGTCCTGAAACGTCTTTAAAATACTTGTATTGTAGTGAATACTTATGTTATTTTTTTGTAAATAATCTATGTCATGACAAGTGCATGAAATGCCAGCAGCATTGCTTGGTATAGTATTATATGCAGGAAGAACTATACTACTATTGAGAATAGTCACATTGTACTTATACCATGTATTATTTTCTGATATAAAGTATTTGCAGGTGACCTGTGGTTTAATCCTACCTGTTAAGCCACTTCCTAAAAAAACAAAAAATATGAAAACCCTTAGCATCCTGTATATACTATTAAAAATTTATAAAATTTTCTGTTTAAATTTCATTTAGACAAAAAAAATAATATATATACATCAGCAAGAAATTATATACAGATTATATAATTTTCTGATTTTTTTTTGCCACAATAAGCATCATTATATGCATTAAAATCTCAATACTAAACACTAAAATCTAAATTCTAAGCATTAAATTCTAAGCATTAAATTCTATGCACTAAACTGTAAGCACTAAAATCTAAGTAACTAAAATCAACACTAAATGTATGCAACCTAAAATGTAAAGCATTACTCATCATCCTCCTCTTCTTCATCCTCATCATCATAGGTTAAGATATATGTGTCATCCTCCATTTCTTCACATTCATCTTCATAAGCATCACTGGGTATTGGTGGAACATTGGATGCAGCATTTTTAAAATATTCTATGTCTTCTGGTGAACACTCATCTAATGATTTTTTGACAGTCCTTTTAACTTCCATGGGATATGATTCCAAATCCTCTTTATATAAGAGTTTACGGTAGCTTTTAGCTGCATCCACATTTGCTGGAGAATCTGGATTTGGCTCATTGAGCAGTGAAATTACACTAAGAAGAATGGTATCAATCTTTTGAGCCGGAGACCAAGTCATTCCCTGTTCTTCAGCATTGTCTCCGTGTAAGATAGAGATACATAGTTTTCCATCAGAGTAAATATTAGGATGCCACATTTCAGAGGTGAATGTTAATCTGGGTGGTGCATATGGGTATTCTGGAGGAAAGGCGATTTTTGCCTTGAATAAGCCTCCCTCATAAAAAGTGTCAGGTGGGCCCCTTAAGATCACATCCCATTCAGTCATATCCTTCTCATTCACCGAAATTTTGAAATTCTCAGAGGGATTCTCTATCAGGTGTCTGTACTCTGCTATTAAAAACCTGGAAACCATGGTTATTTAATATTAATTAAATTCCCTGGTTTATTCCTCCTTACTTGTACAGCTCGTCCATGCCGAGAGTGATCCCGGCGGCGGTCACGAACTCCAGCAGGACCATGTGATCGCGCTTCTCGTTGGGGTCTTTGCTCAGGGCGGACTGGGTGCTCAGGTAGTGGTTGTCGGGCAGCAGCACGGGGCCGTCGCCGATGGGGGTGTTCTGCTGGTAGTGGTCGGCGAGCTGCACGCTGCCGTCCTCGATGTTGTGGCGGATCTTGAAGTTCACCTTGATGCCGTTCTTCTGCTTGTCGGCCATGATATAGACGTTGTGGCTGTTGTAGTTGTACTCCAGCTTGTGCCCCAGGATGTTGCCGTCCTCCTTGAAGTCGATGCCCTTCAGCTCGATGCGGTTCACCAGGGTGTCGCCCTCGAACTTCACCTCGGCGCGGGTCTTGTAGTTGCCGTCGTCCTTGAAGAAGATGGTGCGCTCCTGGACGTAGCCTTCGGGCATGGCGGACTTGAAGAAGTCGTGCTGCTTCATGTGGTCGGGGTAGCGGCTGAAGCACTGCACGCCGTAGGTCAGGGTGGTCACGAGGGTGGGCCAGGGCACGGGCAGCTTGCCGGTGGTGCAGATGAACTTCAGGGTCAGCTTGCCGTAGGTGGCATCGCCCTCGCCCTCGCCGGACACGCTGAACTTGTGGCCGTTTACGTCGCCGTCCAGCTCGACCAGGATGGGCACCACCCCGGTGAACAGCTCCTCGCCCTTGCTCACTGCGGCCGCGGATCCAAAGTAGATGAACCTCTTTTGTTTTTTATTGGGTTCATTTTTACTAAATTTATGAACTGGAAAAAACTTTAACGGCATAATTATCAAATGCGAAGGGGGATCCGTATAAAATCCTAGCTTGCCGGTAATGGCTATTAAGTTAAATTTGGTACCAGTAACACTAATATTTAAAAAGCCCTGATCATTAACTTTCCACATTAAAAGATTATTATATTCGAATGTTTGTCCAATATGGACAACTTTGTCACCAGATGTTACATTTGATTTGGTTGTTAGTGGCTGAAGCTTGGCACAATCAAAAATAAGCCCATTAACACTAAGATATAGAGGAGTGGGTTGATCTATTTTCTCATAGTTTAATATTCCATCTTTCCACGTAATAGCTTGATAATTATCCGCAGCAATGAGTTGAAATTTTATAAATAGTACAGGGGTTTTAGTTGTCGTTATACATTTAAAGGGTGTTTTATAAAAATAAAAAATAATAATTGTTAAAAGTATGATAATAATCGCCAAAATAATTTCATACATTTTTTATAAGAATTATACATAGTATGGTATTTAAAATATTAGCTAAATTTAAAAAAACTTCATGATTTTTAAAACAGGGAAAAAGGGGATTAGGTTGAATAAAAAAGGTAAGCACTTGTCTATATATTTTTTTTACAATGTTGCCTTGAGTCGCATTTTTAACTGGCTGGGGAGTATCAGAGTGGAATATCACTGTAGTAGGTCTATAAGGTCTTGTTAAAATATGATCGGTCATTGTTTTCGTACTAGTGTCATTTAGGGTCGACCTGATAGCTCGATATAAAGTTATAGGGGATAACCTATCAAATACAGTCTTATCTGTGCTGAAATGTATATCGTCTTCTTTATCACTAATAATATTAGGAATGGCTGTCATTAAATAATTACTACTTGTTGTTGTGGGTGAAATAGTTGTACTGGTATTATTGGAAATGGCTGTCATTAAATAATTACTACTTGTTGTTGTGGGTGAAATAGTTGTACTAGTATTATTAGAAATGGCTGTCGTTAAATAATTACTACCTATTACAAGTAAACTAATGCTAACTACATTTTTAACCTCAATAAACCTAAAAAGCCATACTAAATACCTAAACAACATCCTGTTATAATATGAGCAGAAAAAAAAAATAAGTATAATTAGGGAATTATTCTTATTCGCTTACTATTAAGAATAATTCAGAATCTTATTTAGTTAGAAACTATCATAAAGTGAATAGGACTCATCGTCGGATGAAGATTCCGTTTCAGAGATAGTTTCTTTTTCTTCCTCAGAATAATCTGTTCCTACAATAGAATCGGTGTCATCCTCAGAAAGAGAAGTATTTAAATATGGACTATCTATAGCAATATCCTCTTCTATCTCGCAATCCTCCTCCTCCATTTCCATAGTGTGTAGGAGAATATTTTTATCATCATGCTCACTTCTTTTTTTGTTGAAAGATGAACCGTCCTCAATACGGTTCATGTTAAGTTCCTTCATCTTATGTATAATTTCCGTAATCCGTGATGTTTTTGACATGTAAGATGGTTTTAAGGTTATATCCACAATAACAGGAGAATCTCTATCATTTTCATTTGATAAACTTTGATCTTTGATTTCTTCGTCTAAAATTCTTGTCTTTTTTTGGGTACTAGATGAAATAGAGGAATTCATATTCTGAAACGATATATCAAGGGGAGCTGGACGCTTTTTTCCAATTAAACCGTTTTTCGAGATACTATGATTAGATGAATGATCTTTAGCCAAGCTGTCCTTGGATATACTATAGTTAGATATTTTACCTTTAAATAATATTCTTCTATACAAGTTATTCTTAGGTAAAGAATTAGTATGGATTCCTATATTTTTATCTGAAGGAGTGTCCATATCGGAGAACGTCCTCTTACGAATATTTTGACCACGAGCCATTTCATCCACTATAGGCAGTATTTTGGCTGGCTATGGTTCTTTGTTGTGACAATTCTATGAGATTTGATTGCAAATCAATTTTTAGTTTTAAATATATTGGTACCTAGGACAAAGAAAGTATATATAGCCAATAATTATTCCACTAAATTGATTTCCAGACTGATGGGTATGGAGCCATGTTGTCTCTGCAGACGATCGCAAAAATGGCCGTAGCAACAAACACCTACTCCAAGTATCACTATCCAATACTGAAGGTCTTTGGGCTGTGGTGGAAAAACAATACGCTAAATGGCCCTATTAAAATATGTAACCATTGCAACAACATAATGGTAGGAGAATATCCTATGTGTTACAATCATGGAATGAGTCTGGATATAGCTTTGATTCGGGCAGTAAAGGAGCGTAATATATCCTTAGTCCAGCTTTTCACCGAATGGGGGGGAAATATTGACTATGGGGCACTTTGTGCTAACACTCCATCTATGCAAAGATTATGTAAAAGTTTGGGAGCCAAACCACCAAAGGGCCGAATGTATATGGATGCTCTTATACATCTTTCAGATACCTTGAATGATAATGATCTGATTAGGGGGTATGAGATTTTTGATGATAATAGCGTGTTGGATTGTGTCAATCTCATACGACTCAAAATAATGCTTACCTTGAAGGCCCGTATACCTCTCATGGAACAACTAGACCAAATTGCCTTAAAACAACTTCTGCAGCGATACTGGTATGCCATGGCTGTACAACACAACTTAACAATCGCTATCCACTATTTTGATAATCATATTCCTAATATAAAGCCATTTAGTCTGCGCTGTGCTTTGTATTTTAATGATCCCTTTAAAATCCATGATGCTTGCAGAACTGTAAATATGGATCCTAATGAGATGATGAACATTGCTTGTCAACAGGATTTAAACTTTCAAAGCATTTACTATTGTTATCTTTTAGGGGCTGATATTAATCAGGCTATGCTAATGTCTTTAAAGTATGGTCATCTTTCTAATATGTGGTTTTGCATAGATTTGGGGGCGGATGCCTTTAAAGAGGCAGGGGCGCTTGCTGAGAAAAAAAATAAAAGAGTGTTACAACACATATTAGGTCTTAATATCTTTAAGCGAGAGTTGATTCCCCCCTGTAAAGATCCTGATCCTTATCAAATCCAAATTCTGTTAAAAAACTACATTCTAAAAAATGTCTCAACTGTTTTTACATATTATTGCCAGTAGCCATTGTTTATATCAGAAAATAACCCATTTGTTTATCTTTTTTTGTGGGGCAACCATTAAGACCCGACGCAAAAAAAGATTAATCTTTTATCAGATACCTAAAACGTTCTATAAGGGAGTCTATGAGATGGATCATATTTTGATGGTCATAGTAAGAAGCAAGCTTTTTGGCGAAAACAACGGAGTTAAAGAATTTAACCCGCTCATGTTTGGATAGGACTTTTAACAGCGAGCCAAAACAGTATTTAAAAATTTGGCAATAGTTTTTTTGGGATGCAATAAACAAACACTTGATCAGTGCCCGCTTCACTTTCTGATCAGACATGTTTGCCGCATAACAGGCCTTTTTAAACTTAGTAATATAATTATGTTCCGCAAGCACCATTAACAAGGGAACGATGGGAAGCTGCTTTTCTTGGTGAAATTTACGTAAATATTCGATGGCCACCGCTTGGACGACTGTGTAATTTACTAAGTTAGAAATGATAGCTTTCATGGTTGTAAAAATATACATAGGATTTTCTTTTTCTGTATACAGTTTGAAAAGCTTATGATTACGTGAAATGATGGCCATTTTTAATACAAGATGGTATAGTGTATCTTTAGGTAAAAATGCCTTGCAAGCCGCGATGATGTCGATGTTGTCTCCATGAACAGCGATAGAAACTAATGTTTCCAATCTAAATGTTTTTATCTGCATTAATAGAAGAATGCAGTCAATGTTATTATACTTAATAATACTGTAATACACCGAATCAATGACCGTCATCTGAGAATCAAGCTGACTTATTAGTAAATTTAACGTTTTTTTGGAGGCATGACCTTTGATCGCGGCACTAAGTGCACACAGTATAGCAAAATTGTTAAATACATTTTGATTTAGGAGAAGGAGTAATATTTTCCTTCGGTTATAGTACGCAGCATCTGTGATGATTATTGGCCGATAAATGTTAAAATGTGTTAACAGCTTTTTAAAAAAACGGAAGTAATTTTTTTGGATCGCTGTTTGCATCATCGAAATAATGAGATAATCAGGGTATATAATGGGTAGGTCACATGCTACCTCTAACAAAGAATAGTCGCCCAATCTAAAGGCTGTGTTGAAAAGCGTACTATCATCATACGTATCGAGTACCCCTGCTGTTACAAACCAAGCGATAAGATGAATGTGCCGTTCCTTGCAAGCTATCGCAAATAGGGAGTTTCCTATGGAATGTCGAATAATGTACTCCCTATTTTTTTCCAAAATGTTTGGAAAATTGTATAGCGTTGCGGCATACAGTAGACACTCCATTCTGGCGTTATAATTTTTACTTTTACATATGAATAGGTGGAAGAACTCGAATAATTCTTGAGAACTTGTTAAATGCATAATATGGTGATATTTTGGTGTCGTTAAATGGTATGAGAAAATGCATTCTAATACATCTTTTCGGTTATGCTTTAGCGCCTGAGCTAAGGCATATTCAGGCTCGACCCATAGGACTAGTGTTTCTATAATTGAGATATTCGCCTGCTTTGCCAGGGCATACTTTAAGACGCTCCGGTTAGAAAAAATGTTGTTATGAAGATGGATAACCGTATCCATTTTTACGATGGGACCATTCCAGTATAGTCCTAAATGCTGTAGCAGATCTTTTGTTAGTTGTGAAGCGTTCTCGGGTGTCATATAAATATGTTGCAGGGCTTTTTTCTGTAAGGAGAACATTTCGTCGTAATCGTACAAAAAAAAATTAAAATTTGGGCATGGATGATTCAAACATAACAAAATCAAGATTTTATAACAGTTTGCATTAACCTATACATATATGCAAGTAAATGAGATATTATCTATCATAACGAATCAAGGGATATTTGTATATATCAGGAGTTTCTGAAATAAAGATATGAAGATTATCATAGTAGTATCCATCAATCACAATGCAACTTCCTTTAAGGCATAATTTAGTAAACTCAGCACTCCCATCTTCTGGATGCTTTACAACTAACATTAAAAACTCCTCAGTCATATTATCTGTAATAAAATAAGATCCTCCTGGAGCCATTTGTAGCATGTCTCTTATTCCTACAAAATCTTTTTTGGGATGGTAAAAACTCAGCAGTTTCAAACTCTTTTTTAGTTTTTTTTCCTGGTATTTAAGCCATTTGTTATAAAACAGTTTTCTTATGAAAATGCATTTGAAAATATTGGGAATGTTTAACCATGCTTCTTCCGAGCACATCTCCAGATACTTACTTTCTTTGTTTCCCATGTCTAATTTATTGCTCACTAAGTTAGTAATGAATCTATTTTAATAATCTACTTTACTAATCTATCTTAATAACCTATCTTATAATCTATCTTAATAACCTAATTATAACCTATTTATAATTGGCTAATGCTGCCGGCATTTCATGCCTATCTAAACAACTCCTACTAAGCAATCTACTATTACATATATAGATTCACTTTTTATATTTGTAAATCATGAGAATTATAAAATCATTACTCATTTTTATTGTAAATTAGTGGGTATTTGTAAAAATCTTCAAACGTTTTAAGATAGTTTTCTAGAGAGAAGTAATCTTTGCCATCAATATATAATGCTTTTCCTTTAAACTCCAGTTTTGCTATGTTTAGTGAGCCGTTTCTAGATCTTTTTGGGCAATAAATAGATTTTCATTGGTTGCATCGTCCGTAAGCAGAAAGGTACCACTAGGCACGTTAAAAAACATACGTTCTATTTCATGGTCGGATTTTTGAGAATAGAAAAAATCTAATTTTTTAATCCGCGTTAACTCTTTTTTATCAATCTTTCCAGACTGTTTTATATATACTTTATTGCAAATCTTACAATCCTCTATGGCTTCATTATACTTATTTTGCTTATCCTCTATTGACATGTCCGTATTTGATAGGTAACTTCCGTTAAGGCGGTTCCCCATGGTTTTAGATAGATTTTTAATTCAGTTGTATACTTTTATTATGAGGCTAAAATATAGAAGTTTGATCCTAAAAAAATAAAAAGATTTTGTACATTTATTTATGGTTTATAGCGGTATAGAGGCCGATAAAAGGTATCCGGGTAGTCTCCTATGATATCGTCAATTTTGGTATAATAACAGTTGTTATGGTAGTATTGTCCAAACCGAGTATGTATGCGCCGGTGAAGCGTCCGCCCGCTAATGGTACAGTTCCAGGTTAAGACAATCATATCACACCCAAAAAGAGAGGAAACAGCATAGGTGCCCAAAGGTTCATTATATAACATACGCCGCATATATTTTAGTTTTTTTTCTCCATGGTAATAATCACAGGTTTTCATGTCCTGCTTAATAGGATGATTCCCCATGTATGATAATATATAATAAATTTAGTTTTTAGCTTTTTCAAAAAATTGGGCGCTCGAAACTAAATTTTCCTTATCACAGCGTTTGGAGAAAGCGTATTTAAAGATATATCTTCTTCTAACAAGACTGCAAAAAAAATCTTACCCCTTATTTTTATAATGTTCATCATAGCGTTTGAAGATATCAGAAGGTGCCAGGTTTTATAAAAATATCCTTTAGGATTTATAACGATACAAGGGTCTATAAAATATATGCGGGTATAATCTTATAAAATCATCGATTTTTTCATAATATTCTCCGTTTATACAATAAAGATCATAACAGATATTGATGCGTAGATGCATTATTCGCGTGTTCGTTGGGCAGCTAAAGGATATCACAACGTAGTTTTTTTTAAGAAAAGACGAAACTACATAAGTCCCTAAGGGTTCATTGAATAGTAAACGCCATATTTGTTTTAAATTTTGTTGTTCACCATAGTAGTATTCGCACTTTTTCAAGTCTTTTTTAATAAGCCTATTCCCCATGTATGCTTATAAATAAAAATTTAGAAATGTGCTATATTATTTGTTGATGAATCATGAACACGTCTTATATGTTGATATGTTACTTTAAAAACATTTGTATTTTCAACAGACGCGTTCTATTCTTATTAAGAATGATGCCGTCTTTATTTTAAACCTTGGTTTAAAATTTAAAGAAGTATTTATAAACTATAATCATGGGAACTTTTTCAGTAACTGCCTCTGCAAAAAGTGACGATGCTGTTTGTAAGTATTTAGAAGAACCAATAGATGAAAATTACAGAAACATATTAAGAAATGAGCATGTTAAAAAAAATTTAAATGAGGCTCTGAATCGACATATTACTACCTATAATCCAGTAGTTGATTGGTGTAATAACTATTCAACATTTTCATCTCAGGATTTCGATGAATATAAAATTTATATACATAGCGATCTTATGGATGGACGACCTCGTCCAAAAAAAACATGGTGTGTCATCATGTAATGTTTGTTAGTTTTATATAAACGCAAAAATATTCTTCTAGGAGATGTTGATATACTACCTATTGAATTCAATATATTAAAGTACATTTCTGGCTATTCCCATTACGGTATTATTATTACTATTTTTAAGAGCTAGATGTGGATTTAAGTAATAATAACATTCTCCCGTTCCTCCTAGAGACACCTCATCAAATTCCCATCCTATGCAACCTTTATGTTGTAAACATAATGATTGACAGCATTCATCTTCTTTTGACCAAGTCGTCCAAATCCTACCAAGATCTATACGTGTTTTTCCAAATGGAGATTGAAGATCAGCAGTAGTGGCATTAAACCTATAAAAACCAGGTGCATAATCACATGAACGGATCGTAGGATCTAATTTAATATCTTTTATATCTTGTTTTACTGCTTCTAGACAACTTTTATCAGTACATGTTCCACGTACACAGTGGTGTCCTTTATCCTTACAATCCGTATCTGTCTTACATTTTTTTTTCGGCGGTTTATGTTTCAGATGGTAAAAACCCAGTATTAAAATAATCACAAGAATAATTCCTATAAGTACTTGAACAACAGGATAAAACATTTTAATATTAAATATATTTTTTAATTAAATGAATAGATTTAATCCAAGTAGTATTAAAATTTTTTAGAAATAGTGTTCTACAAATAATGAAATGAATGGTCCAAAAAAAATAAGGTGTACAATAATGTAATATATTGTTAGGCTAAGTAAATTTAATATTTTAAAGTATTTGGAAAAATATTTTTTAACATATGATGTCTAGGAATATTTTTTAGACATTTAAAACCATATAGTTACTTTATTTATTACACTGAACTTGAAAAGACTTATTACCTAAAATATTAATAGATGAAGTAATATTGTGTAATTGAGTCCATAACATGGGTGGGAAACAAAAATCTCGTAATATGAAAAATAAACATCCTAAAAAGAGTGCAATTGTTATAAGTTTATGTAACTTTATTTTAAAGTAAGAATATAAAAATATGAGTACAAGAGGAATAGGGGCCATTACTAACATTGGCTCCAACATCCTGTTGTCTACAAAAAAAAATATTTTTTTTAGCAAAAAAAAATCCATGGAAGGATATTAATACACATAATTATTTGACATCACATTAGTGTACTTACCAAATAGTAATATACAACCATCCTAATATTCACCTTTATGAAATGATCCCAACCTATACGGTAAAATAGTATAGGTTTTAATAAAGAAAAAAGATATTCTGTGGTTTTTATTTTTGTATAGTGTGTGAATACAAAATAAAATCCCAAATTTTAACCTTTTCTTTTTTTTTCTATACAGGATGTTAGAAATAGTATTGGCAACGCTGCTAGGCGACCTGCAGCGGCTCCGGGTTCTTACCCCTCAGCAGCGGGCAGTTGCCTTCTTTCGAGCCAATACTAAGGAGCTAGAGGACTTCTTATGCTCAGATGGGCAGTCTGAGGAGGTACTGTCTGGCCCCCTTCTTAACCGTCTACTAGAACCCTCAGGCCCTCTTGATATTTTAACCGGATATCACCTATTTCGTCAGAATCCCAAGGCAGGTCAGTTGCGCGGCCTTGAGGTCAAGATGCTTGAACGGTTATACGATGCTAATATTTACAATATACTGTCTCGGCTGCGGCCTGAAAAAGTTCGCAACAAGGCTATTGAGCTATACTGGGTTTTCCGAGCTATCCATATTTGTCATGCTCCTTTAGTTTTAGATATTGTACGATATGAGGAACCGGACTTTGCTGAACTGGCCTTTATTTGTGCTGCTTACTTTGGTGAACCTCAGGTAATGTATTTGCTCTACAAATATATGCCTCTGACCCGCGCAGTTCTTACGGATGCCATCCGGATAAGTCTTGAGAGCAACAACCAGGTAGGGATTTGCTATGCTTACTTGATGGGAGGCAGCCTCAAGGGACTAGTCTCCGCCCCACTGCGTAAACGTCTGCGCGCCAAACTACGCTCGCAGCGCAAAAAGAAGGACGTTCTTTCACCCCACGACTTCTTACTGCTGCTCCAGTAGCTTTTTTTGCCGCAGGAGCACCGCGGATAGGAGCTCCTCCACGCTCGCGATCCGGCGCTGGAAGCGGAACCGATCGACCGCCACCTGCTCCCAGGGACCCTTGCGCTCGATGTCGTCGGCTTCCCACACCTCGACGGCTGTGGCAAAATGGACATGCTTCGCGTCGTTCGTCCGTTTTTTGCGCCGCCTCCCCATTATTCTTCCTGTAAGATTAGTGTTTAATACCTATAATAACATAATTTTAAGATTTAATATACCAAAACTTAAACTATTTTTGTATAGTAACTATTAGCATGTCTACACATGATTGTTCTCTAAAAGAGAAACCGGTTGATATGAACGATATATCTGAGAAATCAGTTGTCGTGGATAATGCACCCGAGAAACCAGCTGGAGCGAATCATATACCTGAGAAGTCGGCCCGCGAAATGACATCATCAGAATGGATTGCTGAATATTGGAAAGGTATAAAACGTGGAAATGACGTGCCATGTTGTTGTCCAAGAAAAATGACCAGTGCAGACAAAAAGTTTTCAGTATTTGGTAAGGGATCCCTAATGCGCTCCATCCAGAAGAATAATTAAAAAAAATATTTTTTTTAGCAAGTTTTTAAACTATTTAAATAAATGTGGTAAAAAAATTCACATAATAATTAAAGTGAACGTGTTAGAATTAATATTTTTTTATAATCGGATATAATATCCATTAAATCAATAAATGATAGTGTTGCTACCACACTAAACAATAACAAACAGAAACGCACGATACCTTTCCTCATGATTTATAATAGCGTGTTATCTAAAGATTTTTTTGAAAAAAATATTAAATTTTAGTTGATTATTTTTTTCAGTTACAACATTGCTTTAGAAAAAATACCTAATTACTACATAGCAAATAAAGCGAGCGCATTGTTACAAACAACATTTTTTTGCGCCTGGATACTCCTATATATGAGAACTATAATACGGTATATTAATCCTATTACCAACATTGTCAATAATAGTATGTAGGCAATGACATACTTTAAATACCAAATATCCATGGTTATTTCTAAAAATCTTGAAAAAACGTTAAATTTTAGATCGGTCACCTACGACAGTAATACTAATTTTAATAATTGATGACTGAAATCATAATATAATGCCGTGCGAAAAATAATTATTTTTCGGTTAAAGATACCATTACATAAAAAATATGCCATCTACTCTACAAGTGCTTGCTAAAAAGGTATTGGCCTTAGGGGAGCATAAAGAAAATGAACATATATCTAGAGAATATTATTATCATATATTAAAGTGTTGCGGTTTATGGTGGCATGAAGCTCCGATTATACTTTGTTATGATGGGAGTGAGCAAATGATGATAAAGACTCCAATCTTTGAAGAAGGCATATTACTTAATACTGCATTAATGAAAGCTGTACAGGAGAATAATTATGAATTAATAAAGTTGTTTACTGAATGGGGAGCAAACATCAATTATGGATTAATTTCCATTAATACCGAGCATGCCCGGGATCTATGTCGAAAATTAGGAGCTAAAGAAATGCTTGAAGGAAATGAATTTATACAAATTATATTCAAAACATTAGATGATACCACCAGTAGTAATATAATTTTATGTCATGAATTATTCACCAACAATCCTCTTTTAGAGAATGTAAATATGGGGGAAATGAGGATGATAATTTATTGGAGGATGAAAAATTTAACGAACCTATTATTAAATAATGACTCTATTAGTGAAATATTAACTAAATTCTGGTATGGTATAGCAGTAAAATATAATCTTAAGGATGCGATCCAATATTTTTACCAGAGATTCATGGACTTCAACGAGTGGCGAGTAACATGTGCTCTTTCTTTTAATAATGTGAATGATCTTCATAAGATGTATATAACAGAGAAGGTTCATATGAATAATGACGAAATGATGAATCTAGCCTGCAGCATTCAAGACAGAAATTTATCAACCATTTACTATTGTTTTCTATTGGGGGGCTAACATCAATCAAGCAATGTTAACCTCAGTATTAAATTATAATATTTTTAACTTATTCTTTTGTATAGACTTAGGGGCTGATGCCTTTGAAGAGGGTAAGACCCTGGCGAAACAAAAGGGGTATAATGAAATAGTGGAAATCTTATCATTAGATATCATTTATAGTCCAAATACTGACTTCTCATCAAAAATAGAACCTGAACATATTAGTTCTTTGTTAAAAAACTTTTATCCAAAAAATCTGTTCGCTTTTGATCGTTGCAACCCCGGTTTATATTATTCTTAGAGGACCGCTACAAAAATTATTTTTTTTTCTTGATCAAAGCTCCAAAATAATTATTAGATTAAAGTCGCCTATAGCAGCAGCCCACTCCAAAAAAAGTATTTTATAGTACAAAAAACACGAAAAATAGTTTGCGGCCGGCGGCAAACTATTTGTTGTTGTCTAAAACTTAATGTTTTTTTAATATTTTTAAATGCAACCATGGATTGTTGGACTATCAGGGAGAAGAACTATAGCTACATCATATTGTCAATACTGGTAATACTATTAATATGGTATCTTATACTTAACTATTGTCGATCGAAAAAAAATGCAGTTACAAACAACATGCCGCCACCATACACGGTGTCAAGTAGCTGTTCTCAATAATAGGGTTGATTGACGCTCTTCGTAATAATATGTTGATTGACGCATCATAAAATGCTGTGGTTGATTAATATGTTGATTGTCGCCTACTTTATTATATAAGTAATGATTTTTGTATAAAATACGGGTTTGTGAGGGCTTTATTTTTTCTTATTAGAACAAAGCATGCAATTTAAGGCCTACAGCAAGAGTAATTTAACACCTACAACAGTAATTTTAAGGTCAGTAATAATGTTTAATTAAGGCCTGACCACTAAAACTTAAACGATTTTGTAAAAAAAAATGTCTACTCCACTTTCTCTACAGACTCTTGTTAAAAAAGTGCTGGCCACACAGCACATATCTAAAGAACACTACTTTATTTTGAAATATTGTGGTTTATGGTGGCATGAAGCGCCGATTACGATTTGCATTGATGAGGATAGCCAAATATTGATAAAATCGGCAAGCTTCAAAGAAGGCTTATCTTTAGATATCGCATTAATGAAAGTCGTGCAAGAAAATAACCATGATTTAATAGAGTTGTTTACCAAGTGGGGTGCAGATATCAACTCTAGCTTAGTTACTGTTAATACGGAGTATACCCGGAACCTTTGTCAGAAATTAGGCGCAAAGGAAGCTTTGAATGAAAGGGATATTTTACAAATATTTTATAAAACACGTCATCTTAAAACTAGCAGTAATATTATTTTATATAATGAATTGTTTTCTAATAATCTCCTTTTCCAAAATATAGAGAGATTGAGTTTAATAGTTTATAGGGGCTTGAAAAACTTATCAATCAACTTTATATTGGATGATATTTCATTTAGCGAAATGTTAACTAGATACTGGTATAGTATGGCGATATTATATAACCTTACTGAAGCCATCCAATATTTTTATCAACGATATAGGCATTTTAAAGATTGGCGGCTTATATGTGGGCTTTCTTTTAACAATTTGTCTGACCTTCATGAAGTATATAACTTAGAGAAGACGGATATAGACATTGATGAAATGATGAAGTTGACCTGTAGTACGTATGATGGTAATTATTCGACTATTTATTATTGTTTTATGTTGGGGGCTGACATCAATCGGGCAATGTTAACCTCGGTAATAAACTTTCATATTGGTAACTTGTTCCTTTGTATAGATTTAGGAGCTGATGCTTTCGAAGACAGCATGGAACTAGCAAAACAAAAGAATAATAATATATTAGTAGAAATATTATCATTTAAAAATTATTATAGTTCAAATACCTCTCTTTTATCAATAAAAACGACAGATCCGGAAAAAATTAATGCCTTATTAGATGAAGAAAAGTATGAGTCAAAAAATATGTTAATGTATGAAGAATTATCTCATTGATACAAAATTATTTTTTATAACAGAACTCTCTGATGGTGACAAATCTCCGATAGGAATATATGACGTAACATAATTATTTTTTTCGCCCAGAAAAAAATTATAAATGTTATTATTGCCAGCACTTTTATCAACTATACGTACAAAAAGGTGTTGACCAAAAAAATAATTTTTTTTCTTGATCAAAGTATGTAAACGCCCGCTTACAGCAAGGATCT
