# Supplementary material for: African swine fever virus I177L induces host inflammatory responses by facilitating the TRAF6-TAK1 axis and NLRP3 inflammasome assembly
Source: J Virol. 2025 Mar 26;99(4):e02080-24. doi: 10.1128/jvi.02080-24 (PMC11998506; doi:10.1128/jvi.02080-24)
Supplement: Supplemental material — Figures S1 to S6; Tables S1 to S5. [file jvi.02080-24-s0003.pdf]

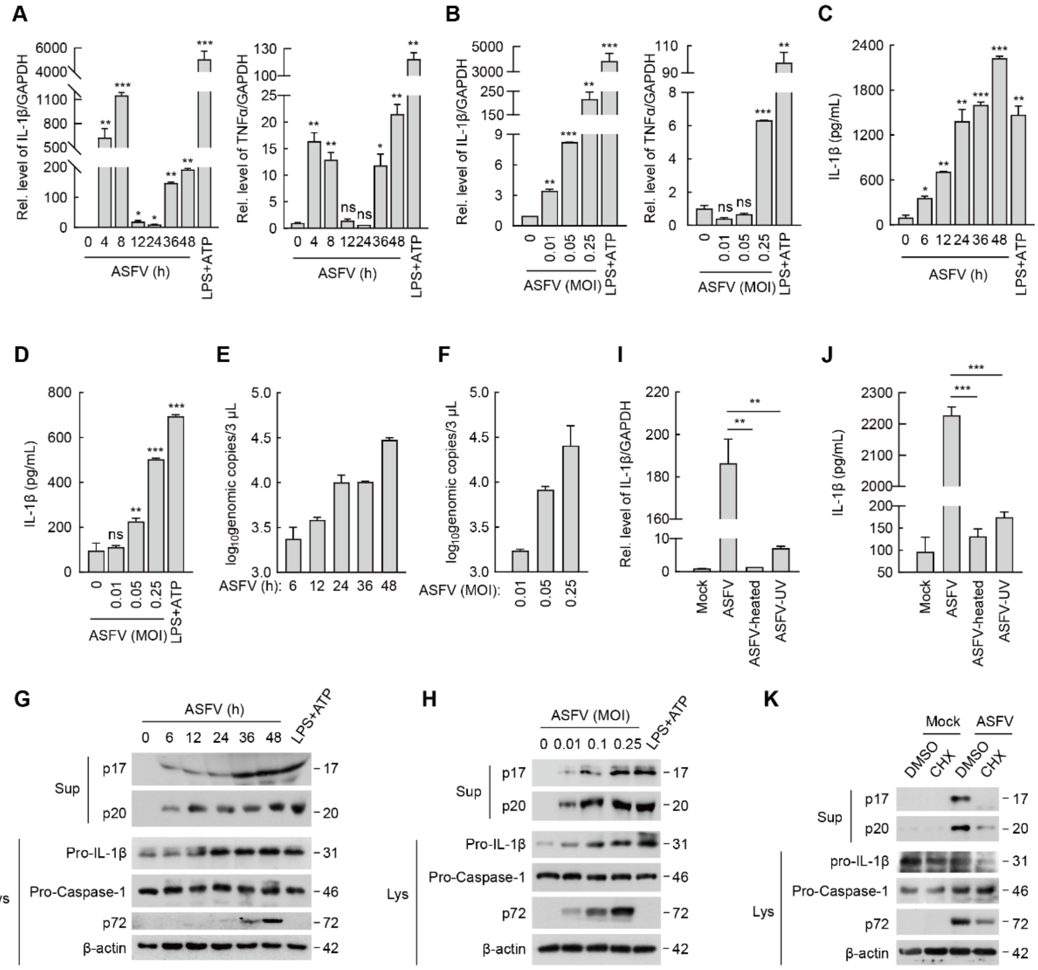

Fig. S1. ASFV infection induces inflammatory responses and IL-1β production in BMDMs. (A-H) BMDMs were treated with LPS (0.1 μg/mL) for 3 h and ATP (5 mM) for 1 h or infected with ASFV at 0.05 MOI for indicated time or for 24 h at indicated dose. (A, B) IL-1β and TNF-α mRNAs were determined by qPCR. (C, D) IL-1β levels were detected by ELISA. (E, F) ASFV genomic copies were determined by absolute qPCR. (G, H) Mature IL-1β (p17) and cleaved Caspase-1 (p20) in the supernatants and pro-IL-1β and Caspase-1 in the lysates were evaluated using Western blot. (I, J) BMDMs were infected with ASFV, heat- or UV-treated ASFV. IL-1β mRNA and secretion were determined by qPCR and ELISA, respectively. (K) Western blot of BMDMs treated with DMSO or CHX (1 μg/mL) for 2 h, followed by ASFV infection

at 0.1 MOI for 24 h. Data are mean (n=3) ± s.e.m. ns, $p > 0.05$, *$p < 0.05$, **$p < 0.01$, ***$p < 0.001$.

A

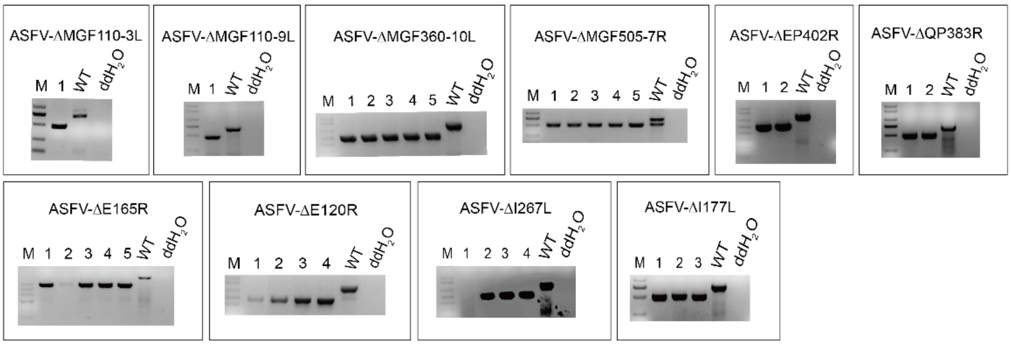

B

No.	Start	End	Gene	SNV	Deletion	Insertion	Amino acid change
1	701	701	MGF-110-3L	A>T			Ser>Ser
2	10157	10158	Non-coding			G	
3	12312	12313	Non-coding		GGGG		
4	69548	69548	M1249L	G>A			Gly>Asp
5	76549	76549	C717R	T>C			Phe>Glu
6	111415	111415	CP2475L	T>C			Gly>Gly
7	159493	159493	E199L	G>C			Arg>Ser

Fig. S2. PCR of ASFV single-gene-deleted recombinant viruses and accuracy of ASFV-ΔI177L by next-generation sequencing. (A) Identification of ASFV single-gene-deleted recombinant virus using Genotyping PCR. (B) Summary of other variations of ASFV-ΔI177L genome compared to parental ASFV genome. Five single nucleotide variations (SNVs), one deletion, and one insertion in non-coding regions were detected in the genome sequence of ASFV-ΔI177L obtained by next-generation sequencing.

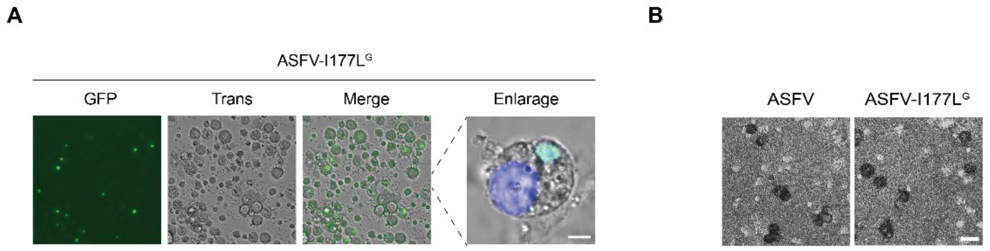

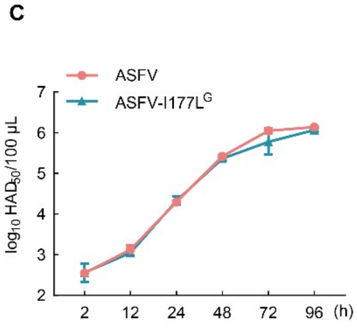

D

No.	Start	End	Gene	SNV	Deletion	Insertion	Amino acid change
1	10157	10158	Non-coding			G	
2	12312	12313	Non-coding		GGGG		
3	69548	69548 •	M1249L	G>A			Gly>Asp
4	76549	76549	C717R	T>C			Phe>Glu
5	111415	111415	CP2475L	T>C			Gly>Gly
6	121723	121723	O174L	C>T			Pro>Ser
7	121827	121827	O174L	C>T			Ser>Phe
8	121852	121852	O174L	C>T			Pro>Ser
9	159493	159493	E199L	G>C			Arg>Ser

Fig. S3. The identification and characteristics of ASFV-I177L^G and accuracy of ASFV-I177L^G by next-generation sequencing. (A-C) The growth characteristics, hemadsorption capability, and replication of ASFV-I177L^G compared to those of ASFV. (D) Summary of other variations of ASFV-I177L^G genome compared to parental ASFV genome. Seven single nucleotide variations (SNVs), one deletion, and one insertion in non-coding regions were detected in the genome sequence of ASFV-I177L^G obtained by next-generation sequencing. Scale bar, 50 μm.

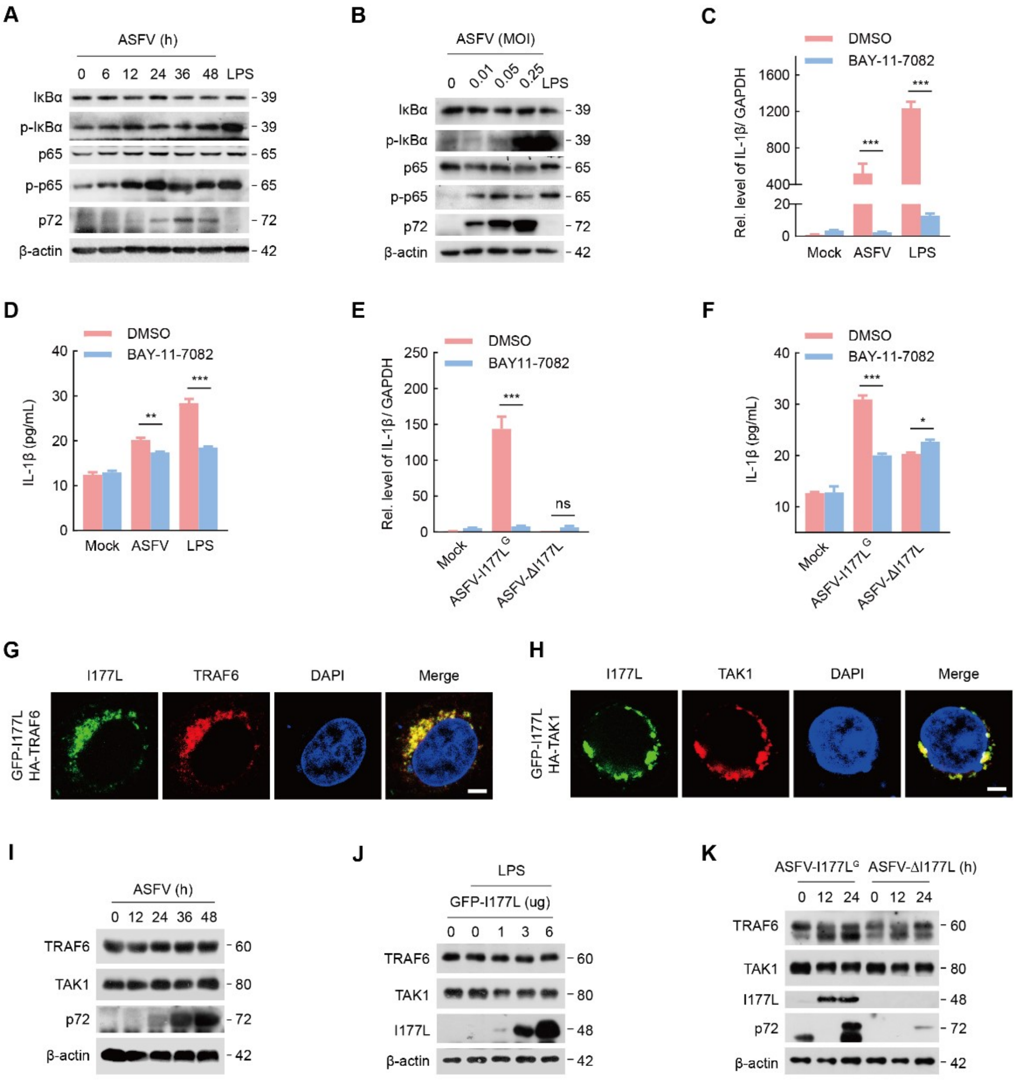

Fig. S4. ASFV infection and viral I177L activates the NF-κB signaling pathway. (A, B) Western blot of extracts of PAMs stimulated by LPS (0.1 μg/mL, 3 h) or infected with ASFV at 0.1 MOI for indicated time or for 24 h at indicated dose. (C, D) IL-1β mRNA and IL-1β levels in PAMs treated with DMSO or BAY11-7082 (an inhibitor of NF-κB, 10 μM) for 1 h and then infected with ASFV at 0.1 MOI for 24 h or stimulated by LPS (0.1 μg/mL) for 3 h. (E, F) IL-1β mRNA and IL-1β levels in PAMs treated with BAY11-7082 and then infected with ASFV-I177L^{G} or ASFV-ΔI177L. (G, H) Confocal microscopy of Hela cells transfected with indicated plasmids for 36 h. Scale bar, 10 μm. (I-K) Western blot of extracts of PAMs infected with ASFV (I) or infected with ASFV-

I177L^G or ASFV-ΔI177L (K) and of LPS-stimulated PK15 cells transfected with indicated plasmid (J). Data are mean (n=3) ± s.e.m. ns, $p > 0.05$, *$p < 0.05$, **$p < 0.01$, ***$p < 0.001$.

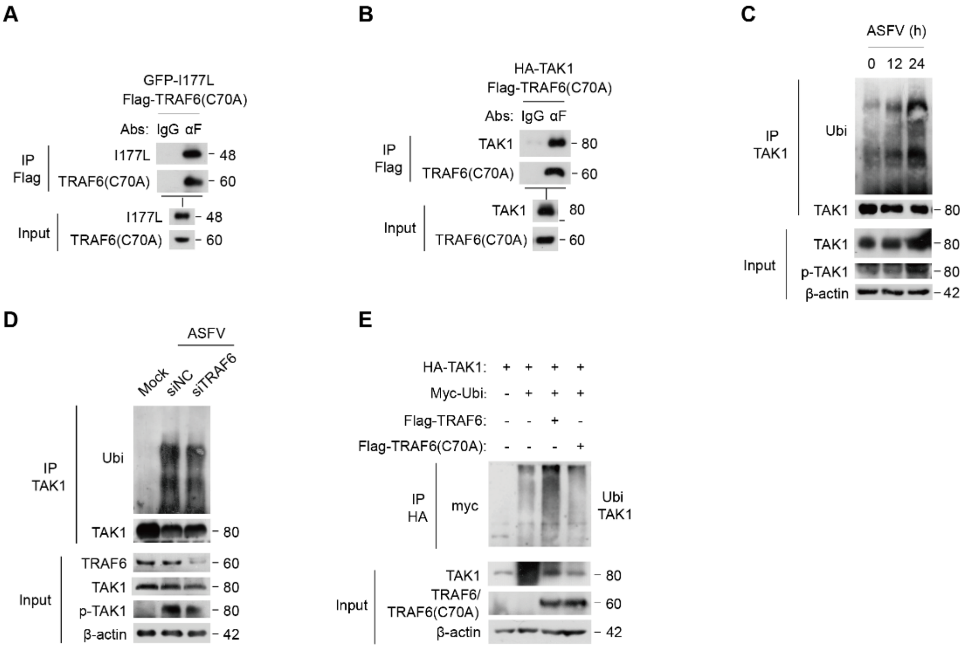

Fig. S5. TAK1 could be ubiquitinated by TRAF6. (A, B) HEK293T cells were transfected with Flag-TRAF6(C70A) and GFP-I177L or HA-TAK1 for 36 h and then immunoprecipitated with anti-Flag antibody. (D, E) Immunoprecipitation of PAMs infected with ASFV (D), transfected with siTRAF6 and then infected with ASFV (E) using anti-TAK1 antibody and Western blot using anti-Ubi antibody. (F) Immunoprecipitation and Western blot of HEK293T cells transfected with HA-TAK1 and Myc-Ubi, with Flag-TRAF6 or Flag-TRAF6(C70A) for 36 h.

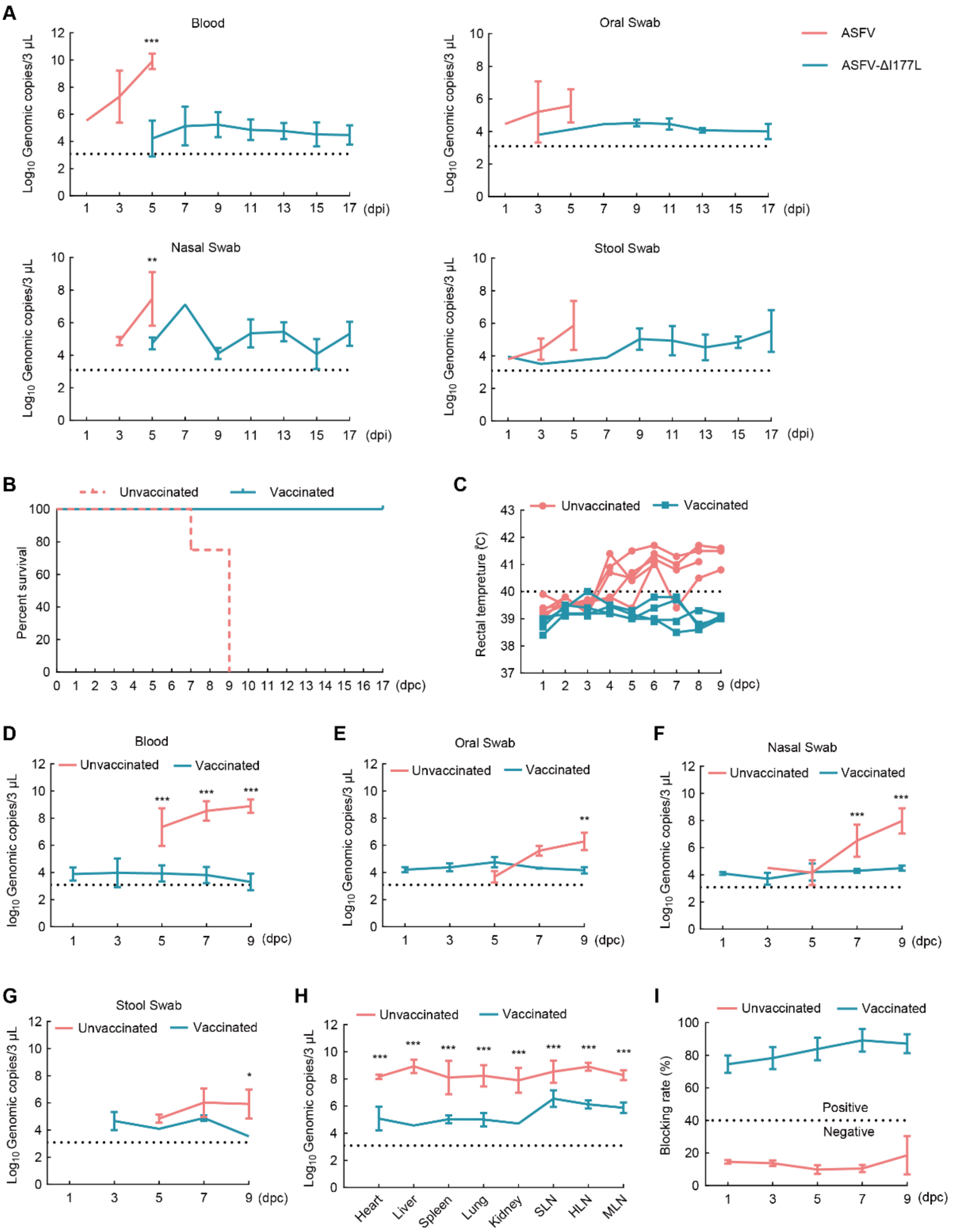

Fig. S6. ASFV-ΔI177L virulence and its protective ability against ASFV challenge. (A) Absolute qPCR of viral titers in bloods, oral swabs, nasal swabs, and stool swabs of pigs infected with ASFV or ASFV-ΔI177L at a dose of 10^4 HAD_{50}. Values were represented as log_{10} genome copies per 3 μL. (B-I) ASFV-ΔI177L vaccinated pigs (Vaccinated, n=4, cyan) and healthy pigs (Unvaccinated, n=5, red) were injected with parental ASFV with 10^2 HAD_{50} and observed the characteristics 17 days post-challenge

(dpc). Survival rates (B). Rectal temperatures during the periods when the unvaccinated group remained alive (C). Absolute qPCR of viral titers in bloods, oral swabs, nasal swabs, stool swabs and tissues samples of the two groups (D-H). ELISA of ASFV p30 antibody blocking rates (I). Data are presented as mean ± s.d. $*p < 0.05$, $**p < 0.01$, $***p < 0.001$.

Table S1. Summarized primers used for plasmids construction.

Plasmids and genes	Sense primers	Anti-sense primers
pEF1α-HApuro	CTAGCTACCCATACGACGTCCCAGACTACGCTTAAG	TCGACTTAAGCGTAGTCTGGGACGTCGTATGGGTAG
pEF1α-pNLRP3-HApuro	CCATTTCAGGTGTCGTGAAGCTAGCGCCACCATGAGCATGGCAAGCGTCCGCTG	TCTGGGACGTCGTATGGGTACTGGGAAGGCTCAAAGACAATGGT
pEF1α-pASC-HApuro	TTCAGGTGTCGTGAAGCTAGCGATCGCCACCATGGGGTGCACGCGTGACGC	TACCGTCGACACTAAGCGTAGTCTGGGACGTCGTATGGGTAGCTCTGCTCCAGGTCGGC
pEF1α-pCaspase-1-HApuro	CCATTTCAGGTGTCGTGAAGCTAGCGCCACCATGGCTGACAAGATCCTGAAG	TCTGGGACGTCGTATGGGTAATATCCTGGAAAGAGGTAGAAAG
pEF1α-pIL-1β-HApuro	CCATTTCAGGTGTCGTGAAGCTAGCGCCACCATGGCCACGGTACCTGAACCTGC	TCTGGGACGTCGTATGGGTAGGGAGAGAGGACTTCCATGGTGA
PRK-I177L-GFP	CTGCACCTCGGTTCTATCGATTGAATTCGCCACCatgtatgaaattattttggcgat	TCCTCGCCCTTGCTCACCATTCTAGAAAAGTAGATGAACCTCTTTTGT
PRK-I177L-CFP	AAAGAGGTTCATCTACTTTTCTAGAATGCTGTGCTGCATCAGAAG	TGGACGAGCTGTACAAGTAACTCGAGGGGGGGCCCGAGCTT
PRK-CFP	CTGCACCTCGGTTCTATCGATTGAATTCGCCACCATGCTGTGCTGCATCAGAAGAAC	GTTCTTCTGATGCAGCACAGCATGGTGGCGAATTCAATCGATAGAACCGAGGTGCAG

Table S2. Primers for recombinant virus identification and its transfer plasmids construction.

Plasmids	Domains	Sense primers	Anti-sense primers
ASFV I177L-p72GFP	LA	CAGATTGTACTGAGAGTGCACCATATGTCGCGAACTAAAATCTAAATTCTAAGCAT	TCCAAACTCATCAATGTATCTTAAGGGAGGAATAAACCAGGGAATTTA
	p72GFP pA	CTTAAGATACATTGATGAGTTTGGACAAACCACAACTAGAATGCAG	GCTAGCGGTCGCCGGAGGAAAAGTCAAAAG
	RA	ACTTTTCCTCCGGCGACCGCTAGCATCGCCAAAATAATTTCATACAT	CACTATAGGGAGACCGGCAGATCTATTCTGAGGAAGAAAAAGAAACT
ASFV-I177LGFP	LA	CAGATTGTACTGAGAGTGCACCATATGTCGCGATTTCCAATAATACCAGTACAAC	ACAGCTCCTCGCCCTTGCTCACTGCGGCCGCGGATCCAAAGTAGATGAACCTCTTTTGT
	GFP	GTGAGCAAGGGCGAGGAGCTGTTC	CTTGTACAGCTCGTCCATGCCGAG
	RA	CGGCATGGACGAGCTGTACAAGTAAGGAGGAATAAACCAGGGAAT	CACTATAGGGAGACCGGCAGATCTACCCTTAGCATCCTGTATATACT

Table S3. Genotyping PCR primers for identification of ASFV recombinant viruses.

ASFV recombinant virus	F1	F2	R
ASFV-ΔMGF110-3L	TGCTTTAAAAAA CCTCCCACACCT	tgccattgattgttgag aacag	agaccatcttgacaagcccag
ASFV-ΔMGF110-9L	TGCTTTAAAAAA CCTCCCACACCT	acagataatccgatgc aaatgcgt	TGCTAGAAAATGTT CCTGGAGGAA
ASFV-ΔMGF360-10L	TGCTTTAAAAAA CCTCCCACACCT	acgtggtggttaatctg tgctctt	TGATACATGATAGG GCCATTATGC
ASFV-ΔMGF505-7R	TGCTTTAAAAAA CCTCCCACACCT	tttgggaaaatcccgc ggaaagaa	TCCTGTAGGGAGA ACATTTTCTCT
ASFV-ΔEP402R	ATGGCGGTTTAT GCGAAGGATCTT	tttgggggagtagcag gtgttaat	TGCCCTAAAGATTG GGTTGGATAT
ASFV-ΔQP383R	TGCTTTAAAAAA CCTCCCACACCT	aattctgtttcaacgcat gggagg	TGATGTCCTGGAGT TTGTTGAACG
ASFV-ΔE165R	TGCTTTAAAAAA CCTCCCACACCT	tcctttgagcacaatcc ctgttta	TTAAGTTCTCATAA TCCCGGCCTC
ASFV-ΔE120R	TGCTTTAAAAAA CCTCCCACACCT	ccggaagatgacgaa gaaagcg	ctagggccggcaataaatacct
ASFV-ΔI267L	TGCTTTAAAAAA CCTCCCACACCT	actggatcgcaacgca atttg	atatgtgtaaacatgtgtgg
ASFV-ΔI177L	cttgccggtaatggctatta agt	GGTGGTGCAG ATGAACTTCA	taatcccctttttccctgtttta
ASFV-ΔDP71L	TGCTTTAAAAAA CCTCCCACACCT	aaactacgctcgcagc gcaaaaag	TTCTTCTGGATGGA GCGCATTAGG

Table S4. SiRNA sequences for the interference of TRAF6/NLRP3/ASC/ Caspase-1 expression.

Genes	Sense primers	Anti-sense primers
siTRAF6	GCAUCUUGAGGAUCAUCAA	UUGAUGAUCCUCAAGAUGC
siNLRP3	GGACUGGGCAUCAGAGAAATT	UUUCUCUGAUGCCCAGUCCTT
siASC	CCAUCGACCUCACUGACAATT	UUGUCAGUGAGGUCGAUGGTT
siCaspase-1	GCUUUGAUUGACUCUGUUATT	UAACAGAGUCAAUCAAAGCTT
siNC	UUCUCCGAACGUGUCACGUTT	ACGUGACACGUUCGGAGAATT

Table S5. Primers used in this study for Quantitative PCR.

Genes	Forward sequence	Reverse sequence
IL-1β	CCTCTCCAGCCAGTCTTCATTG	GGTCATTATTGTTGTCACCGTAGT
TNFα	TCGCCCACGTTGTAGCCAAT	TCTTTCAGCTTCACGCCGT
TRAF6	CTCATCAGAGAACAGATGCCCA	CTCTGCATCTTTTCATGGCAGC
p72	CCGGGTACAATGGGTCTTCC	CGCAACGGATATGACTGGGA
p30	CTCCGATGAGGGCTCTTGCT	AGACGGAATCCTCAGCATCTTC
GAPDH	TCGGAGTGAACGGATTTGGC	TGCCGTGGGTGGAATCATAC
